# A Continuum of Cell States Spans Pluripotency and Lineage Commitment in Human Embryonic Stem Cells

**DOI:** 10.1371/journal.pone.0007708

**Published:** 2009-11-05

**Authors:** Shelley R. Hough, Andrew L. Laslett, Sean B. Grimmond, Gabriel Kolle, Martin F. Pera

**Affiliations:** 1 Eli and Edythe Broad Center for Regenerative Medicine and Stem Cell Research, Keck School of Medicine, University of Southern California, Los Angeles, California, United States of America; 2 The Australian Stem Cell Centre and Department of Anatomy and Developmental Biology, Monash University, Clayton, Victoria, Australia; 3 The Institute for Molecular Biosciences, University of Queensland, St. Lucia, Queensland, Australia; University of Washington, United States of America

## Abstract

**Background:**

Commitment in embryonic stem cells is often depicted as a binary choice between alternate cell states, pluripotency and specification to a particular germ layer or extraembryonic lineage. However, close examination of human ES cell cultures has revealed significant heterogeneity in the stem cell compartment.

**Methodology/Principal Findings:**

We isolated subpopulations of embryonic stem cells using surface markers, then examined their expression of pluripotency genes and lineage specific transcription factors at the single cell level, and tested their ability to regenerate colonies of stem cells. Transcript analysis of single embryonic stem cells showed that there is a gradient and a hierarchy of expression of pluripotency genes in the population. Even cells at the top of the hierarchy generally express only a subset of the stem cell genes studied. Many cells co-express pluripotency and lineage specific genes. Cells along the continuum show a progressively decreasing likelihood of self renewal as their expression of stem cell surface markers and pluripotency genes wanes. Most cells that are positive for stem cell surface markers express Oct-4, but only those towards the top of the hierarchy express the nodal receptor TDGF-1 and the growth factor GDF3.

**Significance:**

These findings on gene expression in single embryonic stem cells are in concert with recent studies of early mammalian development, which reveal molecular heterogeneity and a stochasticity of gene expression in blastomeres. Our work indicates that only a small fraction of the population resides at the top of the hierarchy, that lineage priming (co-expression of stem cell and lineage specific genes) characterizes pluripotent stem cell populations, and that extrinsic signaling pathways are upstream of transcription factor networks that control pluripotency.

## Introduction

Lineage commitment in the mammalian embryo is most often depicted as a series of binary choices between alternate cell states, and increasing evidence supports the hypothesis that fate decisions in embryonic stem (ES) cell cultures reflect these developmental processes [1]. Recent studies of the ES cell transcriptome and epigenome have revealed networks of co-regulated transcription factors that maintain pluripotency and suppress the expression of genes associated with particular differentiation lineages [2]. The pluripotent population is characterized by a high degree of plasticity in chromatin structure [3], and lineage specific transcription factors show bivalent chromatin epigenetic marks characteristic of both suppression and inactivation [4]. These bivalent epigenetic marks are thought to prepare their cognate loci for transcription, in a cell that is poised to embark on lineage commitment. As the pluripotency network is extinguished, stem cell genes shut down, and lineage specific factors are turned on. This models depicts the ES cell as a highly plastic but nevertheless discrete and stable cellular entity, one that in turn gives rise through a massive switch in gene expression to discrete progenitor populations with more limited developmental potential.

However, much evidence indicates that the pluripotent cell populations in the embryo or in ES cell cultures are not comprised of a single cellular entity, but instead display significant heterogeneity at the molecular level, heterogeneity that is associated with an apparent probabilistic element of fate determination[5]. Thus, although the cells of the inner cell mass of the mouse embryo all express the pluripotency factor Oct-4, neither the inner cell mass nor cultures of mouse ES cells show uniform expression of the pluripotency factor nanog [6], [7]. Nanog, and the transcription factor GATA-6, which is a marker for the primitive endoderm lineage, are expressed in mutually exclusive fashion in the E3.5 mouse embryo, and lineage studies have shown that cells at this stage are already committed to either epiblast or primitive endoderm states [6]. However, mouse ES cells lacking nanog can participate extensively in chimera formation, and at least in vitro, nanog positive and negative ES cells can interconvert. ES cells that are nanog−/− are pluripotent but show a greater propensity for differentiation into primitive ectoderm [7]. A more recent study showed overlapping expression of nanog with GATA-6 and a Pdgfra reporter, markers of the primitive endoderm lineage, from the morula to the 64 cell stages [8], suggesting a gradual transition from a pluripotency program to a committed state. Likewise, the expression pattern of cdx-2, a key transcription factor in the trophoblast lineage, overlaps with that of Oct-4, but within any blastomere, cdx-2 expression bears no consistent relationship to that of Oct-4 or nanog until after the trophectoderm lineage has been sorted [9]. These observations have lead to the conclusion that at early stages of development, expression of cdx-2 is stochastic. In mouse ES cultures, a subset of cells is positive for both Oct-4 and the transcription factor Rex1 and these cell types can interconvert [10]. Similar observations have been made with respect to expression of stella in mouse ES cells, with stella positive cells, which resemble the inner cell mass, reversibly converting into stella negative cells which are more akin to epiblast [11].

Heterogeneity in human ES cultures is reflected by the variability in expression of cell surface antigens seen under culture conditions that promote stem cell renewal. Andrews and coworkers [12] showed that ES cell cultures were comprised of populations that were positive or negative for cells expressing SSEA-3. SSEA-3 positive cells accounted for most of the colony forming ability, and they expressed higher levels of pluripotency markers than did the SSEA-3 negative compartment. Similar results were reported by Bhatia and colleagues [13]. Our laboratory used two monoclonal antibodies, GCTM-2 and TG30, recognizing the cell surface proteoglycan characteristic of primate ES cells and CD9 respectively, to fractionate human ES cell populations into four compartments according to their relative levels of expression of both markers [14]. The flow cytometry results showed a quantitative continuous gradient of stem cell antigen expression in ES cell cultures, a gradient that reflected the position of cells in a colony relative to the feeder cell layer. This gradient of surface antigen expression was paralleled by a gradient in expression of pluripotency genes, with highest levels of genes like Oct-4 found in the population of cells with the highest level of antigen expression. Finally, within the fractionated cell populations, there was evidence for coexpression of lineage specific markers alongside of pluripotency genes, similar to lineage priming described first in hematopoietic stem cells [15].

These studies, carried out on bulk populations, did not provide critical information on the patterns of gene expression in single cells; thus, the apparent co-expression of pluripotency and lineage specific genes could have simply reflected the presence of different cell types within the sorted subpopulations. Moreover, these studies did not reveal anything about the developmental potential of the cell subpopulations. In this study, we address both these questions.

## Results

### Immunotranscriptional Profiling Confirms a Continuous Gradient of Stem Cell Surface Marker and Pluripotency Gene Expression in Human ES Cell Cultures

**Figure 1:**
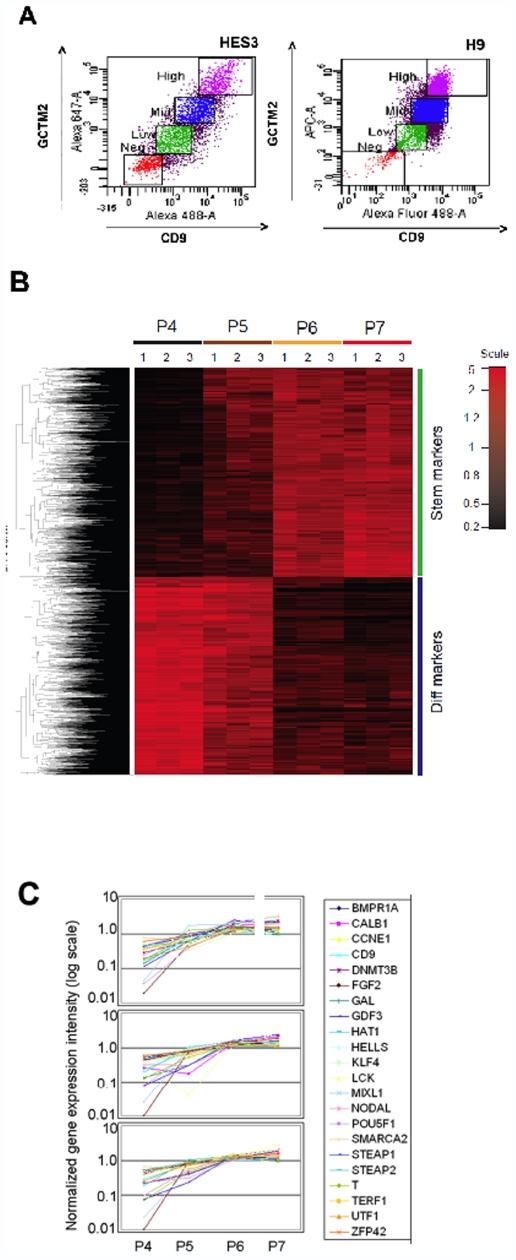
Gene expression in immunologically defined subsets of human embryonic stem cells. A. Fractionation of HES 3 or H9 cells by flow cytometry according to the levels of expression of cell surface markers (GCTM-2, pericellular matrix proteoglycan, and CD9). Cells were separated into High, Mid, Low, and Negative subpopulations as shown. B. Heat map showing gene expression in the four subpopulations isolated as shown in A above. Data are for cell line HES2 at passages 48, 49 and 50 (1,2 and 3) respectively. Subpopulations labeled as follows: P7, High; P6, Mid; P5, Low; P4, Negative. Results for 3752 genes showing a B-statistic greater than zero between P4 and P7 in all experiments are depicted. C. Patterns of expression of selected pluripotency genes in subpopulations isolated as shown in A above. Results are shown for HES-2 at passage 48 (top), passage 49 (middle) and passage 50 (bottom).

We first analyzed several human ES cell lines cultured under different conditions to determine whether the same gradient of cell surface marker and gene expression we observed previously [14] was a general feature of human ES cultures. Using antibodies against the tetraspannin CD9 and the pericellular matrix proteoglycan recognized by monoclonal antibody GCTM-2 [14], we fractionated HES-2, HES-3 and H9 cells grown on serum-containing medium or in the presence of Knockout serum replacer and FGF-2 respectively. For all cell lines, we observed a gradient of expression of the two antigens in the cell population (Figure 1a), as we reported previously, and confirmed in an independent analysis of the secretome of human ES cells [16]. Figure S1 shows QRT-PCR data for expression of the TGF-beta superfamily member GDF3, a gene which is strongly downregulated across the various subpopulations, for four ES cell lines subjected to separation by flow cytometry. The data indicate that although the proportions of cells within the immunologically defined subpopulations can vary from one cell line to another, all cell lines show a gradient of antigen expression that is reflected in the levels of pluripotency genes. Immunotranscriptional profiling was carried out as described on the four cell populations of HES-2 shown in Figure 1a. A global analysis is shown in Figure 1b, and the results for a subset of stem cell genes are displayed in Figure 1c. All genes showing a two fold or greater change across the various fractions are listed in Table S1 and S2. In the present study, we again observed a continuous gradient in the expression of pluripotency genes across the cell populations that paralleled the gradient in cell surface marker expression.

### Transcript Analysis of Cells Isolated by Dissection from Specific Zones of Stem Cell Colonies Shows a Positional Gradient of Expression of Pluripotency and Lineage Specific Genes in Single ES Cells

We sought first to examine gene expression in single ES cells isolated directly from specific zones within colonies, with two objectives. First, we wanted to determine whether the observed regional gradation of expression of cell surface markers of pluripotency was reflected in patterns of pluripotency gene expression, and second, we wanted to compare these results, obtained from cells subjected to minimal manipulation, to those from cells that were separated by flow cytometry in subsequent experiments. For single cell QRT-PCR, we used the technique developed by Klein and coworkers [17]–[19]. To confirm the accuracy, fidelity, and sensitivity of the method, we carried out several tests including the dilution of a pool of cDNA to levels equivalent to single cell content prior to measurement of gene expression in ten replicates. The results confirmed the suitability of the assay for single cell transcript quantitation and show its applicability to both abundant and rare transcripts (Figure S2). Duplicate measurements of Ct values for individual cells were highly reproducible (Figure S3).

We studied the expression of a panel of five pluripotency genes, including the transcription factors Oct-4 and nanog, DNMT3b (a DNA methyltransferase required for de novo DNA methylation during development), GDF3 (a TGF beta superfamily member expressed specifically by ES cells) and TDGF-1 (Cripto, a co-receptor for the stem cell maintenance factor nodal), and two to three genes each characteristic of early stage ectoderm, mesoderm, and endoderm lineage commitment. Under the conditions of culture employed in this study (ES cells grown in serum supplemented medium in the presence of a mouse feeder cell layer) the highest expression of stem cell antigens is observed at the outer edges of the colony, with declining levels towards the middle and center. Isolation of cells from outer, middle and inner colony zones was performed under microscopic guidance (Figure S4), after which cells were immediately lysed and prepared for Q-RTPCR.

**Figure 2:**
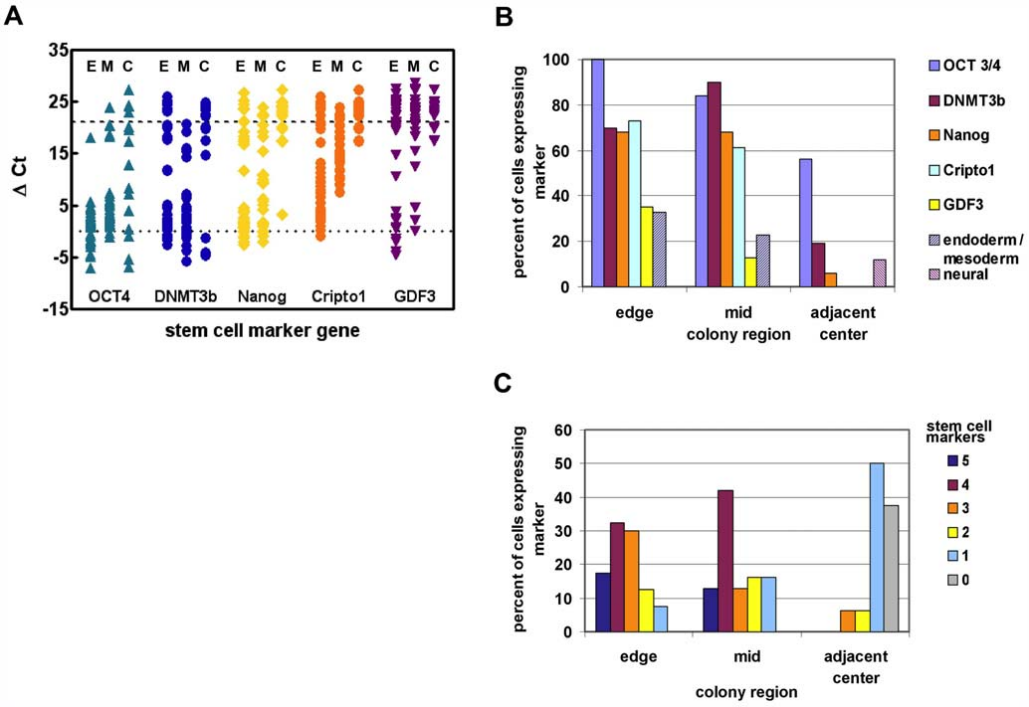
Patterns of gene expression in single cells vary according to position within a colony. Single cell Q-RTPCR for the genes indicated was carried out on 87 cells isolated from different regions of ES colonies. (A) Expression of 5 stem marker genes in single cells isolated from the edge (E), mid (M) or adjacent center (C) region of HES2 colonies. Data are normalized to cyclophilin and expressed as ΔCt (marker gene–cyclophilin). Marker genes expressed at very low or undetectable levels (Ct 35–40) fall above the upper dotted line. Each point represents a Q-RTPCR measurement on a single cell; eighty seven cells were analyzed. Cripto is TDGF-1. (B) Percent of isolated single cells expressing stem or lineage markers according to location with the colony. Endoderm markers were GATA-4, GATA-6, and SOX-17; Mesoderm markers were GSC, MIXL1, and T; Neural markers were PAX-6 and LHX-2. (C) Incidence of co-expression of 0–5 stem marker genes (identified in panel A) in single cells isolated from three colony regions.

The data are summarized in Figure 2a–c. Values for the housekeeping gene cyclophilin A were highly consistent across all cells analyzed. A cell was considered to be positive for a given marker if the value for ΔCt value was 21 cycles or less compared to cyclophilin levels within the same cell (individual data points, Figure 2a). Expression of pluripotency markers was highest and most consistent at the edge of the colony, where most cells expressed between 3–5 of the pluripotency markers shown (Figure 2b–c). However, even at the edge of the colony, only a minority (∼20%) of cells expressed all markers. In the middle of the colony, the majority of cells still showed strong expression of Oct-4, nanog, and DNMT3b, but fewer cells were found to contain transcripts for the growth factor GDF-3. Towards the center of the colony, ∼40% of the cells had lost expression of all pluripotency markers, and we found a number of cells that were null-expressing no markers of pluripotency or lineage commitment, only the housekeeping gene cyclophilin a. However, many cells in this region still expressed Oct-4.

In the edge and the middle zones, we observed a proportion of cells expressing a combination of pluripotency markers and lineage specific genes. In general, cells expressed either mesodermal or endodermal markers, but occasionally cells were found to express endo- and meso- dermal genes. We only found cells expressing neural markers within the central zone, where many cells were negative for both pluripotency and lineage specific markers. The nature of these marker negative cells is unknown. Overall, 15% of cells co-expressed lineage specific and stem cell markers.

### Gene Expression in Single Cells Isolated by Flow Cytometry on the Basis of Stem Cell Surface Antigen Expression

We then harvested single cells and labeled them with antibodies GCTM-2 and TG30 for flow cytometry. We use two stem cell markers to improve the resolution of the analysis, which sorted the population into GCTM-2^HIGH^CD9^HIGH^, GCTM-2^MID^CD9^MID^, GCTM-2^LOW^CD9^LOW^, and GCTM-2^−^CD9^−^ (HIGH, MID, LOW, and NEG).

**Figure 3:**
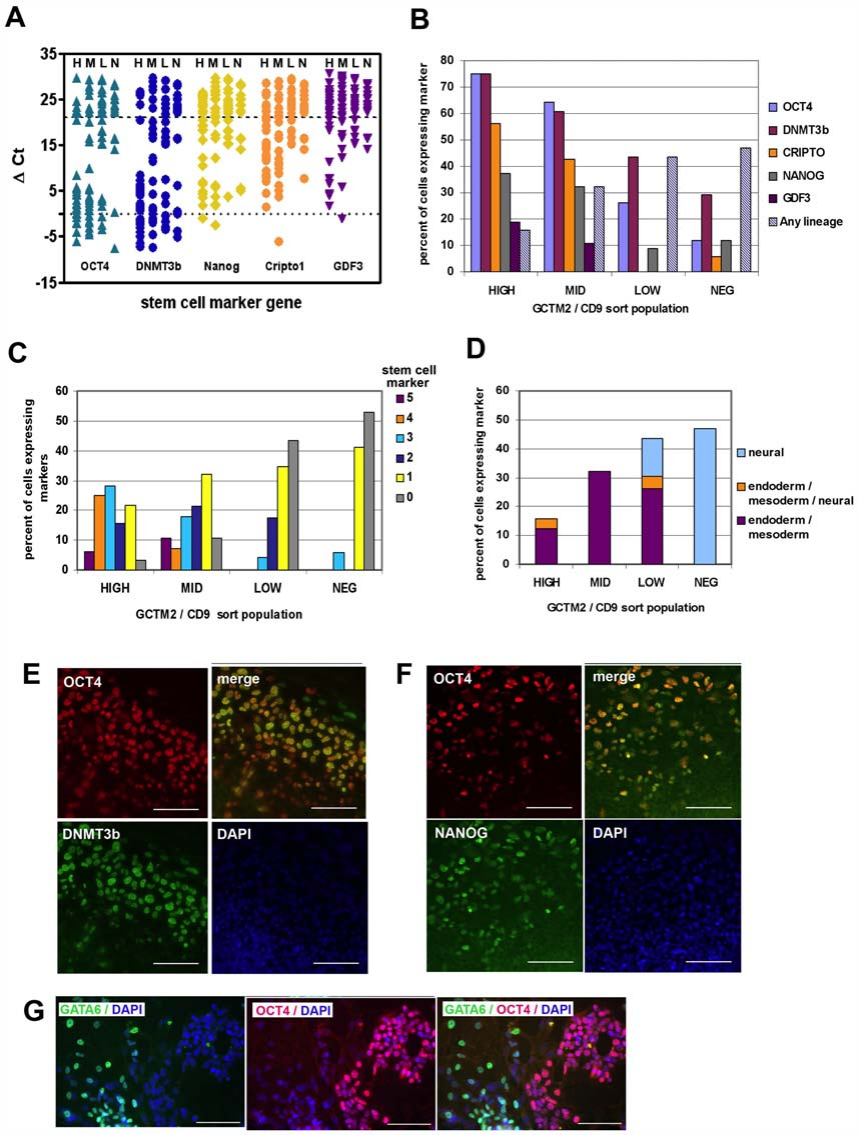
Heterogeneity in gene and protein expression in HES cells. Single cell Q-RTPCR for the genes indicated was carried out on cells separated by flow cytometry on the basis of cell surface marker expression. (A) Expression of 5 stem marker genes in single HES3 cells isolated by FACS into four populations according to levels of expression of GCTM2 and CD9 surface antigens (H = HIGH, M = MID, L = LOW, N = NEG). Data are normalized to cyclophilin and expressed as ΔCt (marker gene–cyclophilin). Marker genes expressed at very low or undetectable levels (Ct 35–40) fall above the upper dotted line. Each point represents a Q-RTPCR measurement on a single cell; one hundred cells were analyzed. Cripto is TDGF-1. (B) Percent of sorted single HES3 cells in (A) expressing stem or lineage markers according to level of surface marker expression. Endoderm markers were GATA-4, GATA-6, and SOX-17; Mesoderm markers were GSC, MIXL1, and T; Neural markers were PAX-6 and LHX-2. (C) Incidence of co-expression of 0–5 stem marker genes (identified in panel A) in sorted single HES3 cells. (D) Detection of lineage marker expression in single sorted HES3 cells. Lineage markers were the same as those in 3B. (E) Immunolocalization of OCT and DNMT3b, (F) OCT4 and Nanog, and (G) OCT4 and GATA6 in HES colonies in culture. Scale bars represent 100 µM.

C_T_ values for cyclophilin were somewhat more variable in cells isolated by flow cytometry and frozen prior to analysis, compared to freshly isolated and unmanipulated cells, but the gradients in patterns of gene expression were similar for cells isolated by the two techniques (Figure 3a–d). The HIGH population expressed pluripotency genes at a greater frequency than the other cell populations, and as with the previous analysis, Oct-4 and DNMT3b were in general more uniformly expressed than were nanog, TDGF-1 or GDF-3 (Figure 3a–b). In particular, expression of TDGF-1 and GDF-3 was observed only in cells expressing relatively high levels of surface antigens. Levels of expression of pluripotency genes declined in parallel with the levels of stem cell surface antigens. Cells negative for all markers studied (expressing cyclophilin only) were found primarily in the LOW and NEG populations (Figure 3c). Cells expressing lineage specific markers were most abundant in the MID, LOW, and NEG populations; only a small minority of HIGH cells expressed lineage specific genes (Figure 3b). As seen in cells dissected from colonies, significant numbers of cells coexpressed lineage specific markers along with pluripotency genes. Amongst the populations that expressed HIGH or MID levels of stem cell surface antigens, coexpression of transcripts for endoderm or mesoderm genes predominated, but in the LOW and NEG populations, there were significant numbers of cells expressing neural lineage markers, in particular Pax-6 (Figure 3d).

In accordance with previous findings in mouse ES cells, the cell populations showed heterogeneity in the expression of some markers of pluripotency at the protein level (Figure 3e–f). The expression of Oct-4 was not always coincident with that of Nanog or DNMT3b. Thus, as anticipated from earlier studies, heterogeneity of pluripotency gene expression in single cells is to a degree reflected at the protein level. By contrast, although cells clearly co-expressed transcripts for Oct-4 and GATA-6 (5% of total cells), we detected no overlap of these markers at the protein level following examination of 300–400 cells (Figure 3g).

### Stem Cell Surface Antigen Expression Reflects a Gradient in Self Renewal Capacity

**Figure 4:**
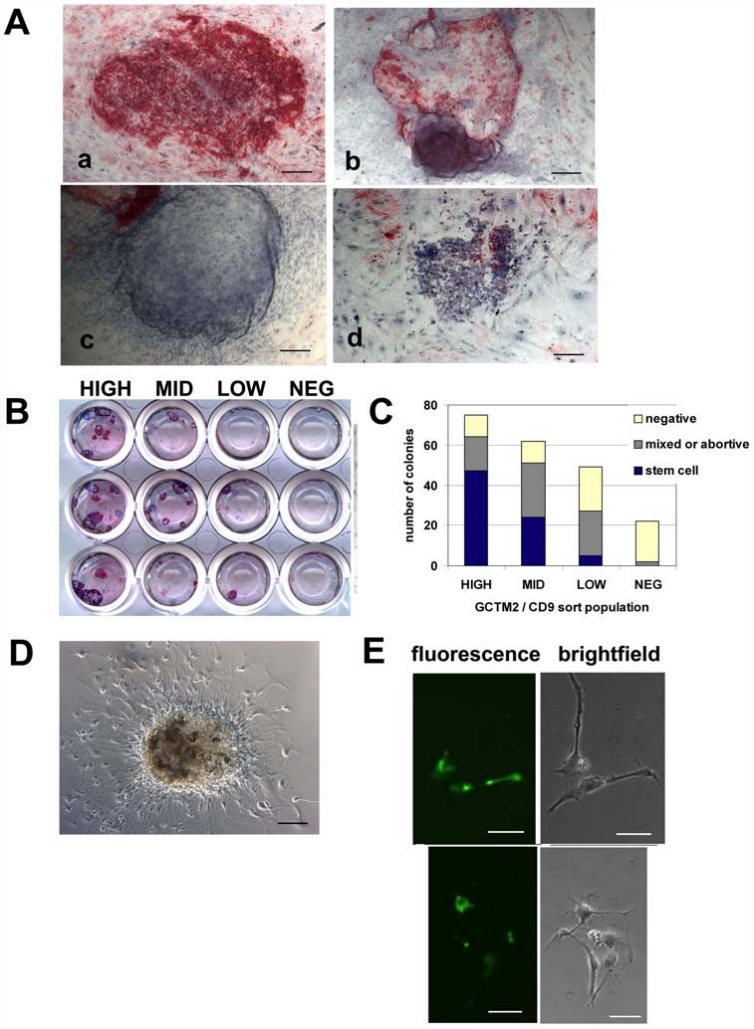
Biological fate of HES3 subpopulations isolated by FACS. (A) Representative images of cell types emerging following culture of sorted subpopulations. After 17 days, cells were fixed and stained for expression of GCTM2 and counterstained with hematoxylin (a) stem colony, (b) mixed or abortive colony, (c) cystic colony, (d) GCTM2-negative pebble-like cells. Scale bars equal 100 µM. (B) Image of cell culture wells following staining for GCTM2 at day 17. (C) Incidence of stem colony formation generated from sorted subpopulations in culture. Colony phenotypes were tabulated across three replicate experiments. (D) Neuronal-like outgrowths emerge within 72 hr following transfer of clusters of small GCTM-2 negative pebble-like cells (panel A–d above), to conditions supporting neural development. Scale bar equals 100 µM. (E) Immunocytochemical localization of β-III tubulin on neuronal outgrowths. Scale bars equal 50 µM.

Having established that a gradient of stem cell antigen expression was paralleled by a gradient of pluripotency gene expression, we wished to ask whether the subpopulations identified by flow cytometry in fact differed in their potential for self-renewal. The addition of the Rho-associated kinase inhibitor [20] prior to and during the sorting procedure enable the analysis of the fate of single cells by a colony formation assay. We replated cells on to a feeder cell layer and analyzed the formation of stem cell colonies fourteen days later by staining for expression of GCTM-2 (Figure 4a, colony morphologies). This assay detects cells that are able to give rise to new colonies containing a majority of cells that express stem cell markers. Overall stem cell colony formation efficiency following single cell isolation and flow cytometry was still quite low (∼0.5%) despite the use of the Rho-associated kinase inhibitor but consistent results were obtained. In the HIGH and MID populations, we observed more large (containing >200 cell) colonies that were predominantly GCTM-2 positive (Figure 4b–c). The MID and LOW populations gave rise to some of these stem cell colonies, but formed more mixed or abortive colonies that were partially positive for GCTM-2 than did the HIGH or NEG fractions. In the LOW and NEG populations, very few cells were capable of forming stem cell colonies; most formed small colonies that were negative for GCTM-2. These small colonies contained round cells with a pebble-like appearance (Figure 4a panel d), and when they were transferred to a medium that supports neural stem cell growth, they gave rise to beta-tubulin positive cells (Figure 4d–e). Large, cystic colonies were found predominantly in the HIGH and MID fractions Figure 4A panel c). These colonies consisted of flat epithelial cells and had a morphology typical of extraembryonic endoderm cells previously characterized in our laboratory [21].

## Discussion

Recent studies of the human ES cell transcriptome and epigenome have highlighted the plasticity of the pluripotent state. ES cell chromatin is open at a global level [3], and the stem cells express high levels of chromatin remodeling factors. Bivalent marking of loci encoding lineage specific transcription factors in ES cells is thought to prepare these genes for rapid activation of transcription once the pluripotency network is extinguished [4]. In fact, most loci in ES cells appear to be poised for transcription or actually undergoing transcription at a low level. Mosher and colleagues observed low level transcription of tissue specific genes as well as LINE elements and other noncoding regions of the genome, indicative of a high level of transcription throughout the ES genome [22]. Similarly Guenther et al. reported that ES cells initiated transcription at many loci throughout the genome, though only a small proportion of these transcripts underwent elongation [23].

These observations, which have provided striking insights into the epigenetic regulation of the molecular blueprint for pluripotency within ES cells, have been made at the population level, and thus reflect the summation of activity within all cells in the population. The view of ES cell transcriptional regulation that emerges from these studies encompasses plasticity but nevertheless envisions lineage commitment and loss of pluripotency as an all or nothing switch. Broadly speaking, however, analysis of transcriptional regulation at the single cell level has provided evidence for a strong stochastic element: genetically identical cells within the same environment show significant variation in molecular phenotype, in a fluctuating manner [24]. This type of regulation poises the cell between adaptability and stability, likely to be a defining state for pluripotent stem cells. For many years, commitment and differentiation processes have been seen as conversions within a bistable system: a cell exists in one of two stable states, and the conversion between the two is abrupt and discontinuous. However, more recent analysis by Chang et al. [25], [26], Mansonn and colleagues [27], and Huang et al. [28] have studied multipotent hematopoietic progenitor cells and characterized metastable states within the population that fluctuated in their transcriptional program. These workers considered that this fluctuation might account for reversible lineage priming towards specific cell fates.

Previously we showed that in cultures of human ES cells grown under conditions that provide for stem cell renewal, there was a continuous gradient of expression of genes associated with pluripotency [14]. The present study shows that such a gradient is also seen at the level of single cell analysis, but reveals that there is considerable cell-to-cell variation within this gradient in the expression of pluripotency genes. Oct-4 is most consistently expressed of the pluripotency genes that we have studied, and it is switched off only in populations that have lost other measurable features of pluripotency, such as stem cell surface marker expression and the capacity for self-renewal. However, the expression of transcripts for TDGF-1, a nodal co-receptor, and particularly.

GDF3, a growth factor implicated in cell renewal (as either a nodal agonist [29] or a BMP antagonist [30]), is mainly limited to cells at the top of the hierarchy. Peerani et al. [31] suggested that there is a minimal colony size effect the maintenance of human ES cell pluripotency, and provided evidence that autocrine regulators of BMP and nodal signaling are key components of this regulatory pathway. This finding suggests that extrinsic signaling pathways may lie upstream of the network of known transcription factors that regulate pluripotency. It is of interest that during reprogramming of mouse fibroblasts to induce pluripotency, the cell surface marker SSEA-1 is upregulated very early on in the process [32]. The pathways that connect expression of stem cell surface glycoconjugates such as the TRA-1-60/GCTM-2 antigen, receptors, and growth factors in human and even mouse ES cells with the transcriptional networks that regulate pluripotency remain unclear.

Under these culture conditions, which provide for stem cell maintenance and inhibition of differentiation, as stem cell marker expression begins to drop, cells switch on the expression of lineage specific transcription factors at the RNA level. Endodermal and mesodermal genes are most often co-expressed alongside of pluripotency genes; the neural genes Pax-6 and LHX-2 are activated in cells that have exited or are near to exiting the pluripotent compartment. It is important to distinguish lineage specific gene expression shown here, which occurs at a high level and with a degree of specificity in the different population compartments, to the low level of genome wide transcription observed in ES cells by others [22], or with transcript initiation at many loci noted previously in ES cells [23]. Lineage specific transcripts in our study were generally found at fairly high levels, and the PCR probes that we used would not have detected the 5′ most ends of the transcripts that are the hallmark of initiation complexes described previously. The lack of co-expression of pluripotency markers and lineage specific markers at the protein level despite robust co-expression of transcripts of both classes of gene suggests a role for post-translational regulation of expression, perhaps by small RNAs.

Differentiation into extraembryonic endoderm [21] or neural progenitors [33] are frequent early outcomes of spontaneous differentiation when human ES cells are cultured in the presence of a feeder cell layer, and it is interesting to speculate that the cells are being primed for these fates. In the HIGH and MID fractions, we observed numerous cystic areas of flat epithelial cells that were negative for GCTM-2. It is probable that these cells represent extraembryonic endoderm, and that they arise directly from stem cells primed to differentiation into this lineage. The small colonies of round, pebbly cells seen in the low and negative fractions were capable of neural differentiation under the appropriate conditions. Such colonies did not form from the HIGH fraction.

The pattern of distribution of cell populations within the colonies under these conditions of culture may be a consequence of localized paracrine and autocrine signaling. Cells towards the edge of the colony are in intimate contact with the feeder cell layer, which produces stem cell maintenance factors such as the BMP antagonist gremlin [21], GDF-11, and GDF-8 [34]. These factors, along with stem cell autocrine factors such as nodal and GDF-3, might maintain a high local ratio of SMAD2/3 to SMAD1 signaling to drive stem cell maintenance. Further into the colony, away from these signals, cells embark on differentiation. Extraembryonic endoderm cells might secrete BMP and Wnt antagonists that enable neighboring cells in the center of the colony, well away from stem cell maintenance signals, to undergo anteriorization and neural commitment.

In summary, human ES cell cultures contain a heterogeneous population of cells with continuously variable expression of pluripotency genes and co-expression of lineage specific genes. These compartments of cells can be resolved on the basis of their expression of cell surface antigens. At one end of the hierarchy are stem cells expressing most pluripotency genes studied and few lineage specific genes; at the other end are fully committed cells that have extinguished the pluripotency program, and in between there is a continuous spectrum. This spectrum in gene expression is reflected in the ability of cells to undergo self-renewal or to form specific differentiated cell types. The overall picture is compatible with a model of multiple states of pluripotency within a highly dynamic cell population. Further prospective, real time studies of hESC lines bearing multiple reporters for pluripotency and lineage specific genes will enable monitoring of the transit of cells through the various compartments. Identification and isolation of these subcompartments in ES cultures, and understanding the factors that provide for their maintenance and differentiation, will yield a more refined understanding of the pluripotent state and will also aid in propagation and directed differentiation of ES cells.

## Materials and Methods

### Human ES Cell Culture

Human ES cells (HES2 and HES3 lines, passage 30–50) were maintained as described previously [35]. Briefly, ES colonies were maintained on mitotically inactivated mouse embryonic fibroblasts (density of 60,000 cells/cm^2^) in media consisting of DMEM (Invitrogen cat. no. 11960-044, L-glutamine 1% v/v (Invitrogen cat. no. 25030-081), penicillin/streptomycin 0.5% v/v (Invitrogen cat. no. 15070-063), non-essential amino acids 1% v/v (Invitrogen cat. no. 11140-050), insulin-transferrin-selenium 1% v/v (Invitrogen cat. no. 41400-045), β-mercaptoethanol 0.18% v/v (Invitrogen cat. no. 21985-023), with 20% (v/v) fetal bovine serum. Colonies were passaged weekly using mechanical dissection. Cell line H9 [36] was maintained in DMEM-F12 (Invitrogen cat. No. 11330) supplemented with 20% Knockout Serum Replacer (Invitrogen cat. No 10828) and FGF-2 (10 ng/ml, Peprotech cat. No. 100-18b) plus antibiotics and L-glutamine as described above[37].

### Microarray Expression Profiling

Total RNA was hybridized to Illumina Sentrix Human 6 V2 and V3 BeadChip arrays and analyzed using BeadStudio v2.3.41. The data from these arrays has been submitted to the GEO repository under accession number GSE13201. Normalisation and statistical analysis of microarray data was performed in R using the Bioconductor packages Limma, Affy and BeadExplorer. RMA background subtraction and Quantile normalization was used on sample data. Differential expression was determined by fitting a linear model to the normalised data using Limma and B statistics were calculated using empirical Bayes methods [38] with B statistics >0 considered significant. Visualisation of data was performed in GeneSpring GX 7.3 (Agilent Technologies). All data is MIAME compliant.

### Immunostaining

HES2 and HES3 colonies grown in 4-well chamber slides (BD Falcon cat. no. 354104) containing MEFs were washed twice with PBS prior to fixing with 2% paraformaldehyde for 30 minutes at room temperature. Samples were permeabilized with 0.1% Triton X-100 and blocked with 1% IgG-free BSA prior to staining with anti-OCT4 at 1∶100 (C-10, sc-5279, Santa Cruz Biotechnology, Inc.), anti-DNMT3b at 1∶200 (sc-10235, Santa Cruz Biotech.) anti-Nanog at 1∶200 (ab21624, Abcam, Inc.) or anti-GATA6 at 10 µg/ml (MAB 1700, R&D Systems, Inc). Secondary labeling was carried out using isotype matched Alexa-fluor labeled secondary antibodies (AF488, 568 and 594, all from Invitrogen Corp). Samples were then fixed and nuclei counterstained using Prolong Gold with DAPI (Invitrogen, cat. no. P36931) prior to imaging. Neuronal outgrowths in 24-well tissue culture dishes were fixed, permeabilized and blocked as above prior to staining with anti-β-III tubulin, 1∶200 (Chemicon mab1637 followed by secondary labeling with AF488 goat anti-mouse IgG1 (A21121, Invitrogen). Images were acquired using Zeiss Axioimager.Z1 and Axiovert 200 microscopes, both with epifluoresence capability and fitted with an Axiocam MRm camera and Axiovision 4.6 software (Carl Zeiss, Inc.).

### FACS Analysis, Cell Sorting and Culture

Cultures of unfixed ES cells were sorted into four populations for parallel analysis of gene expression and development in cell culture. HES3 cultures were treated with 10 µM Y-27632 (ROCK inhibitor, Axxora LLC, San Diego, CA) for 1 hour prior to dissociation to single cells using TrypLE™ (Invitrogen, cat. no. 12605). Cells were stained in solution using a mixture of GCTM-2 (mouse IgM), TG30 (anti-CD9, mouse IgG2a) and Thy1.2-PE (to gate out any mouse embryonic fibroblasts, BD Bioscience, cat. no. 553006). Primary antibodies against GCTM-2 and TG30 were detected using goat anti-mouse IgM-AF647 and goat anti-mouse IgG2a-AF488, respectively (Invitrogen, Carlsbad, CA). For FACS analysis of H9 cells, anti-IgM-APC secondary was used to detect GCTM2.

Control samples included unlabeled cells, cells labeled with secondary antibody only and single fluorochrome labeled cells. Cells were sorted using a FACSAria (BD Biosciences) with a 100 µM nozzle and low pressure conditions. Cells were first gated based on forward and side scatter properties and then Thy1.2 negative cells were analyzed for levels of GCTM2 and TG30 labeling. Cells were sorted into four populations; those negative for both GCTM2 and TG30, and cells that exhibited low level, mid-level or high level expression for both TG30 and GCTM2. Resulting cell populations were sorted into sterile polypropylene tubes containing HES media supplemented with 10 µM ROCK inhibitor.

Sorted cells were plated at 10,000 cells per well in triplicate in 48-well plates containing inactivated MEFs at a density of 60,000 cells/cm^2^. Cultures were fed daily for 17 days with HES media containing 10 µM ROCK inhibitor prior to fixing and staining for GCTM2. Additional wells containing cells from the negative sort population were cultured as above and after 17 days, pebble-like clusters of small cells were excised and transferred to ENStem-A neural progenitor media (cat. no. SCR055, Millipore, Inc) in 24-well culture dishes coated with poly-L-ornithine (20 µg/ml) and laminin (5 µg/ml). Cultures were fed daily prior to fixing and staining for β-III tubulin. In parallel with cell culture, individual cells from each sort population were isolated for RT-PCR analysis. Single cells were washed once in PBS immediately following sorting and picked into PCR tubes containing lysis buffer with tRNA and flash frozen on dry ice prior to storing at −80 °C.

### Immunostaining of Sorted Cells

Sorted cells cultured in 48-well plates for 17 days were washed once with PBS and fixed for 5 min. at room temperature with absolute ethanol. Cells were incubated with GCTM2 hybridoma supernatant (mouse IgM) for 30 min at room temperature following by three washes with 100 mM Tris-HCl, pH 8.5. Secondary antibody (goat anti-mouse IgM- alkaline phosphatase conjugate, Sigma cat. no. A9688, 1∶100 in 100 mM Tris-HCl, pH 8.5) was applied for 30 min at room temp followed by three washes in Tris-HCl as before. Alkaline phosphatase color development was carried out using Vector Lab AP kit II (cat. no. sk-5100) per the manufacturer's instructions. Cells were then washed twice with deionized water and counterstained with Mayer's hematoxylin (Sigma, Saint Louis, MO, cat. no. MHS-1). Cell types present in each well were then categorized and quantified based on phenotype and GCTM2 immunostaining (indicated by AP positive red color). Cell types were categorized as stem colonies (stem colony phenotype containing GCTM2 positive cells), mixed or abortive colonies, or GCTM2-negative cells.

### Single Cell QRT-PCR

Global amplification of cDNA from single ES cells was carried out according to a modified method of Klein et al. [18] as described by Hartmann et al. [19] and Bahar et al. [17]. Single cells were isolated by manual dissection (HES2 at p36–42, eleven separate isolations) and also following separation by FACS (HES3 at p24–41, six separate isolations). For isolation by manual dissection, small pieces were excised from mature (day 6) HES2 colonies from three regions (edge, mid, and adjacent to the center region). Excised samples were digested using TrypLE™ (Invitrogen cat. no. 12605) to isolate single cells. Each single cell was carried through two rinses of PBS using a finely drawn glass capillary pipette under a dissecting microscope and then transferred in a minimal volume (0.5 µL) to a PCR tube containing lysis buffer, protease, tRNA and poly-T gripNA™ probe (mTRAP™ midi-kit, Active Motif, cat. no. 23024). Following lysis and solid-phase capturing of mRNAs with streptavidin magnetic beads, samples were reverse transcribed using random primers. The resulting first strand cDNA products were poly G-tailed followed by 30 rounds of PCR amplification using a single primer. The final volume of cDNA (45 µL) was diluted three-fold with molecular biology grade water prior to quantitative PCR (qPCR) analysis. Gene specific qPCR primer/probe sets (TaqMan Gene Expression Assays, Applied Biosystems) are listed in Table S3. Equivalent amounts of cDNA generated from single cell RT-PCR reactions were used as a template for qPCR. Reactions were performed in duplicate for each sample using TaqMan Universal PCR Master Mix (cat. no. 4304437) with an ABI Prism 7900HT sequence detection system (Applied Biosystems). The thermal profile for PCR consisted of activation steps (50°C for 2 minutes, 95°C for 10 minutes), then 50 cycles of denaturation at 95°C for 15 seconds, followed by annealing and extension at 60°C for 1 minute. For each sample, expression of marker genes was normalized to cyclophilin. Data are expressed as delta Ct (ΔCt) marker vs. cyclophilin.

## Supporting Information

Table S1 (0.79 MB PDF)

Table S2 (0.05 MB DOC)

Table S3 (0.05 MB DOC)

Figure S1 QRT-PCR analysis of GDF-3 expression in immunologically defined subpopulations of four different human ES cell lines. Table below the Figure indicates the proportion of cells in each subpopulation for the different cell lines.
(0.05 MB TIF)

Figure S2 Reproducibility of unbiased global amplification of human embryonic stem cells. Expression of four stem cell marker genes normalized to cyclophilin in nine single cell equivalents prepared from a pool of nine lysed HES3 cells. Each single cell equivalent was separately subjected to mRNA isolation, reverse transcription and global cDNA amplification. Boxes in the box plots indicate the interquartile range (IQR) with the median; the whiskers indicate highest and lowest points.
(1.59 MB TIF)

Figure S3 Reproducibility of single cell Ct measurements. Duplicate values for Ct measurements for four genes on forty single cells isolated by manual dissection are shown.
(0.60 MB TIF)

Figure S4 Isolation of single ES cells from three colony regions Small sections were excised from the edge (A), mid (B), and adjacent center (C) regions of HES2 colonies. Single cells were isolated for global RT-PCR analysis from each section as described in the materials and methods. Scale bars equal 100 µM.
(8.71 MB TIF)
