# Supplementary material for: A Continuum of Cell States Spans Pluripotency and Lineage Commitment in Human Embryonic Stem Cells
Source: PLoS One. 2009 Nov 5;4(11):e7708. doi: 10.1371/journal.pone.0007708 (PMC2768791; doi:10.1371/journal.pone.0007708)
Supplement: Table S1 — (0.79 MB PDF) [file pone.0007708.s001.pdf]

Illumina ID	Gene Name	F-stat	Genbank	Description	fold (p4...p5)	Bstat (p4..p5)	fold (p4...p6)	Bstat (p4..p6)	fold (p4...p7)	Bstat (p4..p7)	fold (p5...p6)	Bstat (p5..p6)	fold (p5...p7)	Bstat (p5..p7)	fold (p6...p7)	Bstat (p6..p7)	AveExpr	P.Value	adj.P.Val
2360296	0	47.40196	CD174408	AGENCOURT	6.421886	0.047056	51.46607	7.124601	49.72504	7.009672	8.014168	1.068551	7.74306	0.788373	-1.035013	-5.661268	386.4219	7.31E-07	6.20E-05
2320411	0	53.95808	CX782759	HESC3 16 C	9.747944	2.707308	44.60349	7.813342	49.48667	8.093707	4.575681	-0.627403	5.076627	-0.293141	1.10948	-5.62319	878.9548	3.61E-07	3.93E-05
270025	0	124.4595	CN264801	17000424505	2.281253	-1.873592	20.83148	10.28882	40.28386	12.42787	9.131599	6.732797	17.65866	9.538094	1.933797	-2.650337	177.9133	3.33E-09	2.57E-06
5570239	MIXL1	120.7673	NM 03194	Homo sapiens	32.71392	10.68463	51.82603	12.40719	35.86225	11.3992	1.584219	-4.792487	1.096238	-6.705477	-1.445141	-4.631193	352.5141	3.95E-09	2.81E-06
1850348	0	144.9664	AK123661	Homo sapiens	2.901359	1.014121	23.23956	11.94308	34.17441	13.21165	8.009888	7.284234	11.77876	9.209511	1.470527	-4.117299	169.4983	1.39E-09	1.47E-06
3060446	0	165.3449	DA737172	DA737172 NT	17.1327	10.74362	45.94304	14.25953	34.07653	13.50134	2.681599	0.688791	1.988976	-2.174225	-1.348231	-4.626466	786.2157	6.53E-10	9.63E-07
1990504	TCP1	197.1743	NM 03075	Homo sapiens	27.34475	12.89765	40.41068	14.81029	30.66359	14.11746	1.477822	-4.336491	1.12137	-6.561849	-1.317872	-4.606933	392.1166	2.36E-10	5.00E-07
5960670	LOC645682	81.53126	XM 93050	PREDICTED:	8.264787	5.39738	25.49613	9.908969	30.66004	10.52159	3.084911	-0.226869	3.709719	0.859561	1.202537	-5.40371	2746.523	3.65E-08	9.35E-06
2060671	GDF3	85.53594	NM 02063	Homo sapiens	4.607427	2.467366	23.62599	9.872844	29.33597	10.60313	5.127806	3.129287	6.367104	4.28272	1.241682	-5.294131	811.145	2.79E-08	8.14E-06
380520	MGAT4C	101.4406	NM 01324	Homo sapiens	7.26175	5.831619	27.87068	11.34837	28.27431	11.42255	3.838011	2.145055	3.893595	2.143716	1.014482	-5.663017	207.4356	1.07E-08	4.89E-06
6280044	POU5F1	84.25567	NM 00270	Homo sapiens	8.326107	5.920045	24.14668	10.25516	26.3871	10.5671	2.900116	-0.283705	3.1692	0.190051	1.092784	-5.597024	5045.864	3.04E-08	8.40E-06
3190561	0	61.82741	BX094358	BX094358 NC	5.70466	2.780196	18.86134	8.019323	26.3716	9.189043	3.306304	-0.358448	4.622816	1.55184	1.398182	-4.957777	1362.664	1.71E-07	2.39E-05
5310494	HHLA2	78.13967	NM 00707	Homo sapiens	4.240652	2.097098	18.69945	9.210083	25.03724	10.24901	4.40957	2.390082	5.904101	4.025015	1.338929	-4.990601	454.2632	4.64E-08	1.06E-05
2100433	LOC168474	109.265	XR 000552	PREDICTED:	6.235233	6.191951	20.34841	11.52718	23.24148	12.0228	3.263456	2.035031	3.727444	2.929158	1.142177	-5.451958	341.0375	6.99E-09	3.84E-06
3140148	CHST4	124.5028	NM 00576	Homo sapiens	4.648152	4.94982	21.01148	12.16888	22.89644	12.50739	4.520395	4.741491	4.925923	5.316763	1.089711	-5.565556	388.454	3.32E-09	2.57E-06
6770482	POU5F1P1	68.54927	NR 00230	Homo sapiens	7.608227	5.19875	18.38305	8.972643	21.73794	9.582582	2.416208	-1.825109	2.857163	-0.791242	1.182499	-5.439559	6367.381	9.63E-08	1.72E-05
6620292	HNRPA1	74.96548	NM 00213	Homo sapiens	13.19117	7.709894	20.32721	9.467543	21.65256	9.697188	1.540971	-4.981685	1.641444	-4.820815	1.065201	-5.631627	490.5864	5.85E-08	1.22E-05
7320215	0	53.77418	DA570727	DA570727 HE	3.222405	-0.706439	13.24626	6.535576	21.46843	8.373277	4.110674	0.904347	6.662238	3.47032	1.620717	-4.31871	171.1589	3.68E-07	3.96E-05
4560170	LOC643272	111.3277	XM 92663	PREDICTED:	6.357824	6.472555	21.68986	11.9352	20.92253	11.84543	3.411522	2.520815	3.290832	2.157666	-1.036675	-5.648516	5145.599	6.28E-09	3.67E-06
5090538	0	39.52848	CX762712	AGENCOURT	5.084159	0.911604	15.61	5.812991	19.57375	6.632639	3.070321	-1.796847	3.849949	-0.682865	1.253924	-5.409493	524.9479	1.94E-06	0.000117
3180674	DCAMKL1	81.87402	NM 00473	Homo sapiens	6.799457	5.604632	19.52407	10.25221	19.50711	10.26105	2.871416	0.181215	2.868921	0.013429	-1.00087	-5.665027	224.5609	3.57E-08	9.28E-06
7320274	NALP12	68.10092	NM 03329	Homo sapiens	3.163142	0.369601	13.1845	8.146547	18.52728	9.491255	4.168165	2.302185	5.857238	4.266025	1.405232	-4.721036	176.8764	9.99E-08	1.77E-05
4560092	PPP2R2C	35.32725	NM 18187	Homo sapiens	4.686109	0.45937	12.0047	4.73827	18.22103	6.348809	2.561762	-2.864287	3.888307	-0.641574	1.517825	-4.847696	171.505	3.52E-06	0.000177
4010369	LOC642464	144.2164	XM 93110	PREDICTED:	3.006191	3.130706	14.88736	12.44181	18.10593	13.22966	4.952234	6.690269	6.02288	8.017002	1.216195	-5.011239	119.0792	1.43E-09	1.48E-06
6040102	0	37.52705	AI810049	wf79g05.x1 S	7.834534	2.554038	20.67701	6.290194	17.8891	5.740089	2.639214	-2.969032	2.283364	-3.997751	-1.155844	-5.569264	509.1816	2.56E-06	0.000143
2710220	MDN1	87.22493	NM 01461	Homo sapiens	7.188556	6.993884	13.64941	10.05959	17.36687	11.03009	1.89877	-2.508099	2.415906	-0.526716	1.272353	-4.990337	245.0465	2.50E-08	7.75E-06
2350482	CXCL12	82.28253	NM 00103	Homo sapiens	3.185875	0.823308	18.57336	10.01346	17.1775	9.726026	5.829909	4.698743	5.391769	4.263332	-1.081261	-5.605102	92.31545	3.47E-08	9.09E-06
7400600	KRT18	84.76014	NM 00022	Homo sapiens	8.230405	7.550721	13.08893	9.77028	17.16663	10.87051	1.590315	-4.136092	2.085757	-1.929413	1.311538	-4.840537	212.6289	2.94E-08	8.22E-06
7100358	TACSTD1	75.76509	NM 00235	Homo sapiens	4.338517	3.409073	13.35925	9.238053	16.84789	10.17583	3.079219	1.059184	3.883328	2.628218	1.261141	-5.111464	4418.919	5.51E-08	1.20E-05
4540347	LAD1	38.06646	NM 00555	Homo sapiens	1.439981	-5.976781	7.402335	2.548093	16.78837	6.03018	5.140578	1.024234	11.65874	4.713663	2.267983	-3.079247	171.1136	2.37E-06	0.000135
4060209	CHGA	23.23	NM 00127	Homo sapiens	4.251533	-1.59648	12.72605	2.90729	16.75045	3.895202	2.993286	-3.134513	3.939862	-2.037321	1.316233	-5.415269	357.3829	3.03E-05	0.000838
2360221	RIT2	84.68362	NM 00293	Homo sapiens	2.750034	0.136627	14.70831	9.692592	16.45091	10.1439	5.348411	4.730975	5.982076	5.39906	1.118477	-5.529062	131.0661	2.95E-08	8.22E-06
2370019	LOC653738	63.58383	XM 92934	PREDICTED:	7.00655	5.478115	13.1796	8.411749	16.23197	9.2534	1.88104	-3.335435	2.316685	-1.893254	1.231598	-5.271548	201.2637	1.46E-07	2.17E-05
6980544	NR5A2	50.95585	NM 20586	Homo sapiens	3.479548	0.616914	9.46815	6.132355	16.17765	8.423754	2.721086	-0.995297	4.649354	2.430343	1.708639	-3.756368	105.9209	4.93E-07	4.74E-05
6400358	C9orf61	162.7377	NM 00481	Homo sapiens	5.574081	9.085111	12.12759	13.26539	16.03743	14.42866	2.17571	1.462796	2.877143	4.245203	1.322392	-4.018941	195.5063	7.15E-10	1.00E-06
7040450	CLDN7	35.7795	NM 00130	Homo sapiens	1.418923	-6.015681	5.996601	1.600138	15.74085	5.906908	4.226165	0.077489	11.09352	4.625071	2.624963	-2.312714	547.8473	3.29E-06	0.000169
6660377	C7orf20	45.24444	NM 01594	Homo sapiens	6.258599	3.363834	12.88461	6.628275	15.60315	7.386848	2.058705	-3.447871	2.493074	-2.368407	1.210991	-5.418443	145.8108	9.40E-07	7.30E-05
4250373	LOC652826	31.35427	XM 94250	PREDICTED:	8.164885	2.616272	14.93754	4.952573	15.59663	5.084625	1.829486	-4.924093	1.910208	-4.971843	1.044123	-5.656672	291.6916	6.58E-06	0.000275
4230358	SLC7A14	11.53092	NM 02094	Homo sapiens	4.535799	-3.160014	10.99293	-0.347861	15.11275	0.666321	2.423592	-5.075966	3.331883	-4.319571	1.374771	-5.479438	280.2681	0.000797	0.009892
770554	0	67.78138	CD250323	AGENCOURT	2.322504	-1.586451	10.34855	7.681176	15.06183	9.294154	4.455771	3.222983	6.485166	5.383815	1.455453	-4.421078	91.34143	1.03E-07	1.79E-05
7330070	HERC6	62.5773	NM 00101	Homo sapiens	2.474565	-1.30212	10.39101	7.354114	15.0259	8.933439	4.199126	2.560167	6.072137	4.70102	1.446048	-4.525706	134.6404	1.60E-07	2.31E-05
7320592	SPIB	64.67939	NM 00312	Homo sapiens	2.01865	-2.695665	8.37779	6.750705	14.25787	9.161476	4.150194	2.85236	7.063072	5.920852	1.701866	-3.397378	100.5294	1.33E-07	2.07E-05
3170338	LECT1	64.68934	NM 00701	Homo sapiens	7.660578	5.721817	17.51159	9.324076	14.24488	8.505608	2.285935	-1.925786	1.859505	-3.757851	-1.229325	-5.293086	121.6768	1.33E-07	2.07E-05
4060367	0	166.1367	CN288134	17000532821	3.789147	6.650778	13.24595	13.71575	14.04695	14.02051	3.495761	5.947635	3.707153	6.507843	1.060471	-5.577947	2147.636	6.35E-10	9.63E-07
6510255	0	112.3637	DA728582	DA728582 NT	3.053529	2.884059	12.91347	11.44476	14.03193	11.81874	4.229031	5.318174	4.595316	5.93812	1.086612	-5.550291	1244.384	5.96E-09	3.63E-06
7380192	GAL	51.66513	NM 01597	Homo sapiens	2.985942	-0.262174	10.03184	6.652142	13.97233	8.087149	3.359691	0.661286	4.679371	2.689019	1.392798	-4.80845	7761.318	4.58E-07	4.51E-05
3440392	FLJ20273	54.72431	NM 01902	Homo sapiens	2.58231	-1.262255	10.36774	6.909243	13.82972	8.151523	4.014911	1.915989	5.355562	3.588977	1.333918	-4.991775	681.8678	3.34E-07	3.76E-05
770369	LOC643300	49.77934	XM 93198	PREDICTED:	8.316211	4.875078	17.12912	7.887549	13.77642	7.006645	2.059727	-3.389614	1.656574	-5.041927	-1.243365	-5.341	351.2853	5.60E-07	5.18E-05
3060202	GRM4	73.22702	NM 00084	Homo sapiens	2.048923	-1.923627	7.009355	6.913581	13.76424	10.14034	3.420995	2.461568	6.717791	6.704442	1.963695	-1.913424	441.4821	6.67E-08	1.34E-05
1430309	NOLA1	36.20025	NM 01898	Homo sapiens	7.833522	3.449448	13.82878	5.784628	13.75867	5.734591	1.765334	-4.78715	1.756384	-5.066638	-1.005096	-5.664891	117.9672	3.10E-06	0.000162
830537	CHODL	41.86758	NM 02494	Homo sapiens	4.999767	2.173373	11.90987	6.331216	13.59837	6.867565	2.382085	-2.443792	2.7198	-1.746036	1.141773	-5.544428	287.0638	1.43E-06	9.56E-05
2570114	TMEM125	24.30188	NM 14462	Homo sapiens	1.675609	-5.462754	4.137517	-0.962769	13.12579	4.489148	2.469262	-3.360879	7.833446	2.463906	3.172384	-1.751096	62.36002	2.42E-05	0.000708
4210398	KLKB1	61.41882	NM 00089	Homo sapiens	4.188514	3.16819	10.2716	8.069195	13.08838	9.140011	2.452325	-0.741318	3.124827	1.027721	1.274231	-5.065165	376.8255	1.77E-07	2.45E-05
2450440	AER61	54.68619	NM 17365	Homo sapiens	8.969914	5.772717	16.9207	8.440207	12.80937	7.3022	1.886384	-3.736385	1.428037	-5.755283	-1.320963	-5.088812	339.8565	3.35E-07	3.76E-05
1240367	LOC96610	193.1791	NM 08092	Homo sapiens	9.62324	12.36258	14.49964	14.73296	12.7737	14.33487	1.506732	-2.635201	1.32738	-4.601001	-1.135117	-5.217453	201.9518	2.66E-10	5.25E-07
5890470	MARVELD3	98.30728	NM 05285	Homo sapiens	1.102138	-6.534556	5.890923	6.82066	12.72187	10.73335	5.344997	6.158131	11.5429	10.10341	2.159571	-0.728624	91.82675	1.27E-08	4.92E-06
5340209	FGF2	70.66139	NM 00200	Homo sapiens	6.652514	5.956967	15.04856	9.767722	12.69159	9.063413	2.262085	-1.377151	1.907788	-3.052337	-1.185711	-5.356654	86.45775	8.13E-08	1.52E-05
1770477	SLC43A1	110.2049	NM 00362	Homo sapiens	3.016649	3.756079	8.160154	10.42341	12.68411	12.49904	2.705039	2.803593	4.204703	6.370078	1.554396	-2.7406	201.8972	6.66E-09	3.72E-06
2850349	LOC138255	62.30628	NM 00101	Homo sapiens	2.76931	0.047802	10.20291	7.916357	12.55602	8.838667	3.684278	2.239656	4.533987	3.547967	1.230631	-5.227737	3533.301	1.64E-07	2.33E-05
5910053	0	47.8255	BX089348	BX089348 NC	1.209782	-6.410625	7.519597	4.25209	12.45535	6.574271	6.215664	3.446151	10.29553	5.829012	1.656385	-4.134512	53.79734	6.96E-07	6.02E-05
7560598	LOC648081	43.687	XM 93713	PREDICTED:	6.487479	4.027741	11.50955	6.675018	12.23926	6.920279	1.774118	-4.227193	1.886597	-4.051014	1.0634	-5.636346	1018.94	1.14E-06	8.24E-05
3450370	MOSC2	258.2582	NM 01789	Homo sapiens	4.39714	10.24573	13.18081	16.22397	12.1329	16.05122	2.997586	7.167893	2.759271	6.550554	-1.086369	-5.379407	243.6638	4.95E-11	2.44E-07
1410736	0	59.43281	AW972815	EST384910 M	4.414667	3.864838	8.563009	7.585906	12.11485	9.206682	1.939673	-2.504694	2.744227	0.305256	1.414789	-4.427231	1795.652	2.12E-07	2.76E-05
6270164	MARVELD3	42.44434	NM 00101	Homo sapiens	1.543269	-5.30294	6.359241	3.617094	12.09558	6.710532	4.120631	1.379082	7.837637	4.818458	1.902048	-3.291352	92.38839	1.33E-06	9.12E-05
1980192	CTSC	81.82102	NM 00181	Homo sapiens	4.948211	5.718661	11.1241	10.06754	12.04704	10.43266	2.248106	-0.374119	2.434625	0.206496	1.082967	-5.572663	393.8394	3.58E-08	9.28E-06
3190044	GABRB3	83.05137	XM 93725	PREDICTED:	3.506091	3.609919	9.476437	9.624295	12.00897	10.72228	2.70285	1.525851	3.425174	3.400633	1.267245	-4.862673	1383.031	3.29E-08	8.81E-06
7610671	EBI3	10.2532	NM 00575	Homo sapiens	2.653419	-4.794447	6.551405	-1.790256	11.9925	0.371946	2.469042	-4.868164	4.519641	-2.947548	1.830524	-4.95538	92.33909	0.00131	0.014423
5270347	ZC3HAV1	91.39142	NM 02462	Homo sapiens	4.015554	5.508851	7.763545	9.604882	11.9516	11.68866	1.933368	-1.018179	2.976326	3.110177	1.539451	-3.048955	97.44686	1.92E-08	6.37E-06
1510220	ZC3HAV1	46.56865	NM 02462	Homo sapiens	3.565662	1.678269	6.538454	5.314078	11.83059	8.241506	1.833728	-3.399968	3.317923	1.108895	1.809387	-3.024042	92.54438	8.05E-07	6.55E-05
4220687	FLJ90231	143.5139	NM 17358	Homo sapiens	1.041424	-6.650151	6.643288	9.502149	11.72073	12.3516	6.379041	8.806972	11.25452	11.77682	1.764296	-1.415974	87.18095	1.47E-09	1.48E-06
6560497	GRPR	82.89579	NM 00531	Homo sapiens	2.13333	-1.293046	10.56764	9.380578	11.68762	9.839942	4.953591	5.259044	5.47858	5.926262	1.105982	-5.529958	80.13035	3.33E-08	8.86E-06
2680672	CDC14B	34.66682	NM 03333	Homo sapiens	6.420174	2.770696	12.78927	5.782651	11.64913	5.3569	1.992044	-3.997102	1.814457	-4.790552	-1.097873	-5.613701	565.4826	3.89E-06	0.00019
2070678	C20orf54	65.97357	NM 03340	Homo sapiens	7.256674	6.79546	10.78999	8.773333	11.61615	9.102726	1.486905	-4.73328	1.600755	-4.380146	1.076568	-5.600939	185.3117	1.19E-07	1.94E-05
3370326	HM13	52.57221	NM 03078	Homo sapiens	6.682671	5.160411	11.61922	7.789501	11.6021	7.768605	1.738709	-3.973082	1.736146	-4.226138	-1.001476	-5.665015	175.1784	4.16E-07	4.26E-05
5910037	IBRDC2	42.06256	NM 18275	Homo sapiens	1.598446	-5.10426	7.056715	4.147451	11.58966	6.496785	4.414736	1.769444	7.250579	4.408412	1.642358	-4.121893	114.2395	1.39E-06	9.41E-05

6940286	CXCL12		111.3932	NM	00060	Homo sapiens	5.572306	7.849663	12.32197	12.02816	11.55944	11.78947	2.211287	0.584998	2.074445	-0.254844	-1.065966	-5.586142	282.1332	6.26E-09	3.67E-06
3190592	NLN		80.63045	NM	02072	Homo sapiens	6.015341	6.86252	11.09134	10.04668	11.55292	10.23969	1.843842	-2.301958	1.920577	-2.10774	1.041617	-5.640732	245.0707	3.89E-08	9.67E-06
1690025	SMPDL3B		46.51713	NM	01447	Homo sapiens	4.503401	2.555576	10.65211	7.000528	11.54445	7.342462	2.365347	-1.820841	2.563496	-1.412146	1.083772	-5.609502	376.6433	8.09E-07	6.58E-05
1850204	TUBG1		71.34931	XM	94451	PREDICTED:	6.931176	6.780204	12.18878	9.551792	11.50314	9.298799	1.758544	-3.218542	1.659624	-3.972632	-1.059604	-5.623705	134.3354	7.71E-08	1.45E-05
3990338	LSM12		155.0455	NM	15234	Homo sapiens	5.801628	9.956563	10.79002	13.46038	11.49028	13.79866	1.859827	0.147503	1.980527	0.796398	1.064899	-5.553208	119.6976	9.45E-10	1.19E-06
2940332	MGC15416		38.55116	NM	13841	Homo sapiens	3.684707	0.791345	8.501944	5.355719	11.45659	6.719021	2.30736	-2.343915	3.109226	-0.417727	1.347525	-5.019921	248.3223	2.22E-06	0.000129
6840519	DTWD2		78.66726	NM	17366	Homo sapiens	3.537647	3.757337	8.506519	9.180271	11.39316	10.57827	2.40457	0.541154	3.220548	2.985166	1.339345	-4.473494	154.8687	4.47E-08	1.06E-05
6520497	ACTG1		44.82927	NM	00161	Homo sapiens	7.10981	4.62821	12.14484	7.054692	11.26473	6.703366	1.70818	-4.428021	1.584393	-5.161622	-1.078129	-5.62095	6035.493	9.88E-07	7.53E-05
2480041	DIABLO		104.9554	NM	13893	Homo sapiens	7.936843	9.660837	10.12541	11.13212	10.96541	11.523	1.275748	-5.357067	1.381584	-4.853071	1.08296	-5.543464	95.85684	8.78E-09	4.41E-06
870358	PYCARD		31.53341	NM	14518	Homo sapiens	2.10261	-3.613802	6.497342	3.062105	10.89317	5.532295	3.090131	-0.956589	5.180782	2.001528	1.676557	-4.183842	845.9753	6.38E-06	0.00027
4060475	SPINT1		61.15745	NM	18164	Homo sapiens	2.600289	0.034317	8.389829	7.637108	10.87197	8.877552	3.226499	1.839801	4.181063	3.616694	1.295851	-4.916947	154.1139	1.81E-07	2.48E-05
6100390	FLJ25801		53.11471	NM	17355	Homo sapiens	2.344257	-1.092307	6.428835	5.867641	10.85856	8.527765	2.742377	0.314688	4.631983	3.986212	1.68904	-3.265176	749.9677	3.93E-07	4.15E-05
3460504	TM4SF18		20.18875	NM	13878	Homo sapiens	1.828761	-5.321366	7.577289	1.234861	10.76811	2.70652	4.1434	-1.236574	5.888198	0.28188	1.421103	-5.220461	103.3542	6.06E-05	0.00141
3940639	LOC652097		30.77905	XM	94142	PREDICTED:	1.549609	-5.410148	3.952448	0.308607	10.71848	5.597621	2.550609	-2.122108	6.916889	3.6625	2.711858	-1.532644	711.5806	7.24E-06	0.000294
6760435	FLJ11286		61.66624	NM	01838	Homo sapiens	2.603883	0.277593	7.642457	7.459104	10.68221	9.106971	2.935023	1.354282	4.102416	3.763264	1.397746	-4.411593	80.03069	1.73E-07	2.42E-05
450039	GYLTL1B		15.82191	NM	15231	Homo sapiens	2.795507	-3.841788	9.208418	1.138958	10.65213	1.676926	3.294007	-2.958863	3.810446	-2.516464	1.156781	-5.600408	106.4872	0.000193	0.003361
1510390	SLC9A9		26.80287	NM	17365	Homo sapiens	4.225103	-0.217309	11.56884	4.440795	10.59885	4.027999	2.73812	-2.55666	2.508543	-3.287485	-1.091518	-5.627135	107.7264	1.47E-05	0.000491
2000369	SC4MOL		110.9114	NM	00674	Homo sapiens	8.168848	9.737674	12.4858	11.99124	10.59672	11.30398	1.528465	-3.548378	1.297211	-5.48057	-1.17827	-5.174515	483.1202	6.42E-09	3.68E-06
3610767	WDR1		33.66377	NM	01749	Homo sapiens	4.711093	1.680964	9.862363	5.31538	10.55038	5.590779	2.093434	-3.401171	2.239477	-3.180835	1.069762	-5.634587	308.848	4.54E-06	0.000213
3450626	DBC1		130.3747	NM	01461	Homo sapiens	3.01634	4.807162	9.33712	12.23538	10.54362	12.84215	3.095513	4.998197	3.4955	6.065375	1.129215	-5.30036	1391.465	2.55E-09	2.19E-06
1850291	MGC26856		29.45624	NM	15277	Homo sapiens	2.744572	-2.154852	8.611274	4.004237	10.53342	4.892305	3.137566	-1.192002	3.837909	-0.14717	1.223212	-5.431387	11126.39	9.09E-06	0.000347
130328	DHX9		43.95585	NM	00135	Homo sapiens	7.181731	4.822322	11.30496	6.898721	10.52458	6.553821	1.574128	-4.906779	1.465466	-5.590314	-1.074149	-5.623985	653.4332	1.10E-06	8.07E-05
5420164	UGT8		46.79268	NM	00336	Homo sapiens	3.138334	0.669805	8.244362	6.442841	10.50226	7.594254	2.626987	-0.578492	3.346444	1.084175	1.273872	-5.12246	141.7327	7.84E-07	6.47E-05
2680692	HTR7		24.03413	NM	00087	Homo sapiens	4.423014	-0.560969	12.84003	4.096193	10.48335	3.217561	2.903006	-2.66474	2.370182	-3.972078	-1.224803	-5.492534	117.8723	2.56E-05	0.000741
4890082	C21orf59		21.70135	NM	01783	Homo sapiens	6.228377	1.366795	8.519066	2.602455	10.48313	3.475792	1.367783	-6.044261	1.683124	-5.552336	1.23055	-5.475286	180.3683	4.25E-05	0.001086
4610376		0	68.6608	CA392028		cs21c02.x1 Hu	5.267302	5.592004	10.77681	9.372177	10.4095	9.212708	2.045983	-1.623632	1.976249	-2.15667	-1.035286	-5.649127	1480.281	9.54E-08	1.72E-05
1400369	SYT1		49.21665	NM	00563	Homo sapiens	3.434886	1.617052	8.281695	6.818079	10.4003	7.907612	2.411054	-1.018372	3.027846	0.620385	1.255818	-5.150409	126.7771	5.96E-07	5.40E-05
6040201	CTNNB1		99.59341	NM	00190	Homo sapiens	8.834803	9.757362	10.94292	11.05242	10.34397	10.81667	1.238615	-5.66836	1.170821	-6.306533	-1.057903	-5.60874	760.5757	1.18E-08	4.92E-06
5080554	SCNN1A		62.01699	NM	00103	Homo sapiens	2.686052	0.859655	6.922619	7.324091	10.27231	9.333441	2.577247	0.587749	3.824317	3.629681	1.483877	-3.891826	3094.843	1.68E-07	2.37E-05
1660086	PIK3CB		43.05548	NM	00621	Homo sapiens	6.722683	4.609996	10.5186	6.709112	10.25485	6.569019	1.564643	-4.909347	1.52541	-5.322539	-1.025719	-5.659711	209.3019	1.23E-06	8.67E-05
4900754	DONSON		106.0201	NM	01761	Homo sapiens	8.590565	9.916583	11.36067	11.53317	10.23594	11.08853	1.322458	-5.060137	1.191532	-6.165441	-1.109881	-5.463138	130.3662	8.29E-09	4.24E-06
6590561	CCR7		14.13364	NM	00183	Homo sapiens	2.622655	-3.982359	6.082051	-0.369424	10.19439	1.808029	2.319043	-4.370902	3.887051	-2.217738	1.676144	-4.860195	95.4529	0.000325	0.005054
1660546	EPHA1		97.77428	NM	00523	Homo sapiens	3.539466	4.834857	9.723179	11.01931	10.16743	11.25298	2.747075	2.718901	2.872589	3.053051	1.04569	-5.625965	2840.751	1.31E-08	4.95E-06
5820403	ANXA2		32.75395	NM	00100	Homo sapiens	5.13664	2.623542	7.305833	4.361204	10.15331	5.935765	1.422298	-5.571959	1.976644	-3.790197	1.389754	-4.921117	180.3512	5.24E-06	0.000233
4150661	RBM35A		91.96656	NM	01769	Homo sapiens	1.502235	-4.19108	7.436309	8.772344	10.13837	10.33898	4.950163	6.257676	6.748858	8.182506	1.363361	-4.27837	57.21131	1.86E-08	6.20E-06
1010739	SCG3		178.0726	NM	01324	Homo sapiens	2.164386	2.90832	8.095155	13.22473	10.08549	14.32481	3.740162	7.989269	4.659746	9.806027	1.245867	-4.197613	246.8256	4.26E-10	7.40E-07
3610164	THOC4		109.1973	NM	00578	Homo sapiens	8.154576	9.745778	12.35666	11.96532	10.07338	11.08685	1.515304	-3.636031	1.235304	-5.892023	-1.226665	-4.921291	1047.323	7.01E-09	3.84E-06
6620435	ECT2		53.98187	NM	01809	Homo sapiens	9.716899	6.679521	13.87819	8.248839	9.983594	6.783569	1.428253	-5.385817	1.027447	-6.777819	-1.3901	-4.79168	163.243	3.60E-07	3.93E-05
4670202	CXCL12		115.4675	NM	19916	Homo sapiens	4.662027	7.322284	11.74733	12.42305	9.976354	11.73192	2.519791	2.386146	2.139918	0.557864	-1.177518	-5.113672	960.7581	5.10E-09	3.27E-06
6180427	GPR160		66.46851	NM	01437	Homo sapiens	2.302652	-0.680565	8.593614	8.245129	9.975278	8.968083	3.73205	3.30369	4.332082	4.300602	1.160778	-5.381025	102.468	1.14E-07	1.91E-05
7160039	LOC652615		33.51762	XM	94215	PREDICTED:	6.277307	3.244958	10.01365	5.3761	9.882502	5.283501	1.595214	-5.076233	1.574322	-5.404523	-1.013271	-5.66387	214.8521	4.64E-06	0.000215
7550280	LOC653631		31.88516	XM	93047	PREDICTED:	9.489551	4.339246	11.50378	5.050211	9.869843	4.340825	1.212257	-6.288387	1.040075	-6.775356	-1.165548	-5.53519	532.574	6.03E-06	0.000259
7100653	LOC648996		19.6508	XM	93807	PREDICTED:	1.113692	-6.641678	4.939288	-0.862721	9.86148	2.254418	4.435057	-0.966679	8.854763	2.027461	1.996539	-4.153035	30.59629	6.91E-05	0.00157
3360392	SHC1		28.84831	NM	18300	Homo sapiens	4.24476	0.845394	8.284397	4.224798	9.861076	5.010839	1.951676	-3.966856	2.323118	-3.0777	1.190319	-5.475064	105.6701	1.01E-05	0.000375
4860630	CDCP1		52.50789	NM	02284	Homo sapiens	1.939818	-2.684528	6.172241	5.739578	9.755876	8.135337	3.181866	1.548719	5.029274	4.604298	1.580605	-3.702222	175.763	4.19E-07	4.26E-05
380376	SLC25A24		55.9887	NM	01338	Homo sapiens	5.650338	5.011913	11.27793	8.495581	9.683139	7.777954	1.995974	-2.471155	1.713728	-3.9932	-1.164698	-5.418923	88.23021	2.95E-07	3.42E-05
3890646	MTAC2D1		35.74316	NM	15233	Homo sapiens	2.242139	-2.775174	7.68015	4.693245	9.666865	5.785437	3.425368	0.282564	4.311448	1.606633	1.258682	-5.282299	70.81	3.31E-06	0.000169
2230661	LCK		52.6513	NM	00535	Homo sapiens	1.181599	-6.358081	4.053093	2.793934	9.649655	7.810177	3.430176	1.879847	8.166605	7.0122	2.380813	-0.919528	59.81885	4.13E-07	4.24E-05
3140403	PCNA		117.8767	NM	18264	Homo sapiens	9.521289	10.86393	11.0932	11.99556	9.638664	11.38428	1.165094	-5.991302	1.012328	-6.78149	-1.150906	-5.264738	95.70468	4.54E-09	3.04E-06
4280088	LOC654037		101.1513	XM	93819	PREDICTED:	4.112806	6.602855	8.477903	11.05473	9.637133	11.70821	2.061343	0.397937	2.343202	1.661953	1.136735	-5.309969	411.8824	1.08E-08	4.90E-06
3440072		0	52.47823	Z39772		HSC1JB062 n	4.949486	3.226883	15.43972	8.688295	9.555106	6.584822	3.11946	0.287834	1.930525	-3.536657	-1.615861	-3.965244	82.79546	4.20E-07	4.26E-05
5700278	NPFFR2		27.16862	NM	05303	Homo sapiens	1.331579	-6.188532	5.00523	1.037631	9.421861	4.214718	3.758868	-0.232938	7.075705	3.039269	1.882403	-3.791257	50.95928	1.38E-05	0.000466
2850377	ITPR2		120.2854	NM	00222	Homo sapiens	1.742528	-2.048888	10.82281	11.63716	9.356741	10.98604	6.210983	8.394297	5.369637	7.846842	-1.156686	-5.255796	66.48926	4.04E-09	2.81E-06
4540327	PRO1853		85.56806	NM	14473	Homo sapiens	5.184854	7.212993	8.967371	10.35943	9.32157	10.5638	1.729532	-2.235034	1.797846	-2.010071	1.039499	-5.637074	193.3492	2.79E-08	8.14E-06
6180192	PSMC4		57.47004	NM	15300	Homo sapiens	5.417412	5.245793	9.999144	8.466185	9.310519	8.114033	1.845742	-2.878792	1.718629	-3.744398	-1.073962	-5.604557	700.8961	2.55E-07	3.16E-05
6480364	ENPP1		54.55079	NM	00620	Homo sapiens	3.423615	2.339989	8.284724	7.706332	9.256418	8.249162	2.419876	-0.379517	2.703697	0.404254	1.117287	-5.515816	560.6672	3.40E-07	3.80E-05
2650754	CAV1		16.19533	NM	00175	Homo sapiens	1.646213	-5.618303	3.184672	-2.680211	9.194536	2.587751	1.934544	-4.757658	5.585266	0.504076	2.887122	-2.367471	113.3552	0.000174	0.003101
2760100	FLJ16231		67.57237	NM	00100	Homo sapiens	6.047411	6.512303	10.68663	9.467752	9.175435	8.741955	1.767142	-2.931193	1.51725	-4.582952	-1.1647	-5.358836	146.8255	1.04E-07	1.81E-05
5570445	MCRS1		23.58923	NM	00633	Homo sapiens	3.405868	-1.202315	8.509528	3.40351	9.146398	3.696686	2.49849	-2.91152	2.685482	-2.702128	1.074842	-5.637558	79.6961	2.81E-05	0.000797
2360278	MCM7		27.6035	NM	00591	Homo sapiens	3.432187	-0.909874	10.27078	4.604365	9.129044	4.031499	2.992488	-1.620336	2.659833	-2.557879	-1.125066	-5.58708	108.5942	1.27E-05	0.000441
2340402	CYP2S1		25.20836	NM	03062	Homo sapiens	3.496577	-0.69852	8.093976	3.635939	9.117211	4.16064	2.314828	-3.125059	2.607467	-2.602892	1.126419	-5.583392	646.6323	2.01E-05	0.000623
1430021	AMD1		30.76575	NM	00163	Homo sapiens	5.121502	2.246827	8.790364	4.87925	9.104939	5.012776	1.716364	-4.623318	1.777787	-4.656361	1.035786	-5.656574	357.3246	7.26E-06	0.000295
7150494	PCDH10		77.8106	NM	02081	Homo sapiens	9.371596	8.451666	14.07338	10.40405	9.099504	8.446591	1.501706	-4.507004	-1.029902	-6.77245	-1.54661	-3.666966	194.6492	4.75E-08	1.08E-05
580537	CARD11		35.11074	NM	03241	Homo sapiens	2.155403	-2.740351	6.049874	3.990109	9.078399	6.084918	2.806841	-0.648327	4.211926	1.971042	1.500593	-4.443555	259.9135	3.64E-06	0.000181
1340707	TMTC3		18.02307	NM	18178	Homo sapiens	4.069594	-0.543589	5.623034	0.903595	9.027183	3.158937	1.381719	-5.980666	2.218202	-4.06138	1.605394	-4.689015	75.80274	0.000105	0.002138
290706	LOC388755		32.23434	XM	37389	PREDICTED:	2.149158	-3.291698	7.769035	4.337619	9.006576	5.025791	3.614921	0.304705	4.190747	1.075028	1.159291	-5.51576	411.2071	5.69E-06	0.000247
940735	RABGAP1L		31.00225	NM	01485	Homo sapiens	3.029831	-0.625777	6.995239	4.233999	9.002186	5.471554	2.308788	-2.413518	2.971184	-0.830433	1.286902	-5.206321	232.7176	6.97E-06	0.000286
6550204	KCNK1		154.9437	NM	00224	Homo sapiens	4.803315	9.554561	9.900165	13.81759	8.999179	13.44815	2.061111	2.046079	1.873535	0.685689	-1.100119	-5.373025	255.2526	9.48E-10	1.19E-06
5720767	KCND2		67.91738	NM	01228	Homo sapiens	2.907388	1.936555	8.553543	8.933309	8.988044	9.17984	2.942003	2.092693	3.09145	2.407921	1.050798	-5.628365	472.7935	1.01E-07	1.78E-05
4250386	LOC641860		28.96946	XM	93576	PREDICTED:	3.529099	-0.29522	9.165603	4.685099	8.97235	4.548602	2.597151	-2.138732	2.542391	-2.492451	-1.021539	-5.662151	428.3383	9.90E-06	0.00037
6520577	CPNE1		73.74642	NM	00391	Homo sapiens	6.401158	7.306073	10.48411	9.902446	8.953864	9.149375	1.637845	-3.417258	1.398788	-5.137688	-1.170903	-5.305797	178.9225	6.41E-08	1.29E-05
7650615	LOC391045		52.5541	XM	37278	PREDICTED:	8.250722	6.756539	9.870599	7.639055	8.95328	7.150181	1.196332	-6.155574	1.085151	-6.707996	-1.102456	-5.567312	191.7313	4.17E-07	4.26E-05
7040020	PSMB8		78.6171	NM	00415	Homo sapiens	1.209971	-6.039123	4.565001	5.856926	8.936208	9.809481	3.772818	4.559249	7.385472	8.729108	1.957548	-1.137487	71.77865	4.48E-08	1.06E-05
6270553	NODAL		22.00067	NM	01805	Homo sapiens	1.726331	-5.151617	5.23493	0.912039	8.923852	3.540975	3.032403	-1.79041	5.169259	1.049352	1.704675	-4.360211	1812.907	3.97E-05	0.001029
2490564	KIAA1160		42.59266	NM	02070	Homo sapiens	5.525811	4.247957	8.717178	6.584969	8.907987	6.668342	1.577538	-4.638036	1.612069	-4.73161	1.021889	-5.660568	102.7118	1.30E-06	9.00E-05
2940411	COL6A2		99.73514	NM	05817	Homo sapiens	3.687311	6.060314	7.916135	10.98491	8.898825	11.60313	2.146858	1.065986	2.413364	2.213767	1.124138	-5.351865	356.0425	1.17E-08	4.92E-06
650753	FASTK		20.92104	NM	00671	Homo sapiens	2.973192	-2.127675	7.047231	2.319559	8.894477	3.384826	2.370258	-3.313354	2.991559	-2.168991	1.262124	-5.395918	97.79231	5.09E-05	0.001244

7400739	CRSP8		43.8396	XM 93359	PREDICTED:	6.289062	4.979005	8.914245	6.710667	8.862199	6.658707	1.41742	-5.34387	1.409145	-5.638587	-1.005873	-5.664707	260.422	1.11E-06	8.16E-05
380731	TUBA1		54.22067	NM 00600	Homo sapiens	1.678747	-3.729303	5.445156	5.496184	8.852025	8.186447	3.243584	2.117149	5.272996	5.379711	1.62567	-3.329997	462.0007	3.51E-07	3.87E-05
6400066	CTNNB1		107.2558	XM 94565	PREDICTED:	7.645528	10.03334	9.814675	11.59509	8.814154	11.10425	1.283714	-5.171508	1.152851	-6.311857	-1.113513	-5.420104	883.3326	7.76E-09	4.15E-06
4230475	LOC651143		57.22482	XM 94253	PREDICTED:	4.990537	5.471198	7.565281	7.853332	8.750144	8.598185	1.515925	-4.312705	1.753347	-3.186758	1.156618	-5.380755	177.3586	2.61E-07	3.20E-05
7330292	RTP1		42.74125	NM 15370	Homo sapiens	2.149024	-1.848829	5.087785	4.455561	8.692434	7.453086	2.367487	-0.895677	4.044829	3.080432	1.708491	-3.184994	119.2611	1.28E-06	8.89E-05
4490358	ITGB1BP3		53.03733	NM 17067	Homo sapiens	1.402979	-5.331003	5.254359	4.927716	8.657998	7.715298	3.745144	2.858925	6.171152	5.962146	1.647774	-3.350364	314.6535	3.97E-07	4.18E-05
6130494	DBR1		45.11137	NM 01621	Homo sapiens	8.315021	5.95001	10.53531	7.0322	8.654022	6.071705	1.267021	-5.993099	1.04077	-6.769396	-1.217388	-5.336974	121.1171	9.55E-07	7.38E-05
20577		0	40.1986	AL833138	Homo sapiens	2.646031	-1.242106	9.773358	6.393805	8.631506	5.768473	3.693591	1.184612	3.262057	0.199869	-1.132289	-5.538558	96.9294	1.77E-06	0.00011
20279	C1orf172		76.12579	NM 15236	Homo sapiens	1.437733	-4.592037	5.49211	7.10372	8.621385	9.648313	3.81998	4.664286	5.996514	7.62545	1.569777	-3.057177	75.42419	5.37E-08	1.19E-05
2900612		0	43.98036	BF942123	nae87b02.x1 I	1.777607	-3.996742	7.734589	5.753786	8.620885	6.276872	4.351123	2.647134	4.849712	3.245519	1.114589	-5.558655	53.64055	1.10E-06	8.06E-05
3890561	NTS		23.3873	NM 00618	Homo sapiens	5.235149	1.579356	7.245359	3.045968	8.592288	3.83452	1.383983	-5.875085	1.641269	-5.375949	1.185902	-5.500453	4163.815	2.93E-05	0.000818
730021	TES		27.83339	NM 15282	Homo sapiens	2.698293	-1.836187	7.271994	3.840826	8.585471	4.629475	2.695035	-1.715963	3.181816	-0.809763	1.180621	-5.483131	287.7151	1.22E-05	0.000428
620520	PRKAR1B		86.72641	NM 00273	Homo sapiens	1.905761	-0.787214	5.64422	8.523434	8.579503	10.88453	2.961662	3.769283	4.501877	7.022839	1.520051	-2.887709	307.7613	2.58E-08	7.89E-06
1510341	LOC554223		25.64767	XR 00111	PREDICTED:	4.445751	0.928322	8.172923	3.936128	8.573751	4.127626	1.838367	-4.4119	1.928527	-4.372177	1.049043	-5.651058	165.8688	1.84E-05	0.000584
1990347	DPAGT1		47.47578	NM 00138	Homo sapiens	4.780673	3.732713	10.07764	7.663395	8.567465	6.856939	2.107996	-2.190268	1.792104	-3.759432	-1.176269	-5.402327	72.44869	7.25E-07	6.19E-05
4900348	UBL7		16.81323	NM 03290	Homo sapiens	3.29684	-2.314568	7.972049	1.759829	8.554043	2.028073	2.418088	-3.769135	2.594619	-3.647506	1.073004	-5.645157	60.68995	0.000146	0.002728
6060739	T		40.26744	NM 00318	Homo sapiens	5.099274	3.216369	10.59561	6.878755	8.49154	5.798928	2.077866	-2.852595	1.665245	-4.707461	-1.247784	-5.266078	102.5332	1.76E-06	0.000109
3390678	NFE2L3		131.7453	NM 00428	Homo sapiens	3.385572	6.525355	9.116284	12.88881	8.459341	12.57739	2.692686	4.44456	2.498644	3.672424	-1.077659	-5.501836	1929.814	2.40E-09	2.13E-06
4560315	HMBS		63.70411	NM 00019	Homo sapiens	4.779087	5.764375	7.836029	8.654222	8.45653	9.045875	1.63965	-3.323225	1.769486	-2.772799	1.079186	-5.575955	480.8166	1.45E-07	2.16E-05
5870398	MT1M		40.12265	NM 17687	Homo sapiens	3.702177	2.486907	5.602112	5.038229	8.441223	7.289601	1.513194	-4.681212	2.28007	-1.418114	1.506793	-4.065274	179.2949	1.79E-06	0.000111
5420156	GABRA5		74.75426	NM 00081	Homo sapiens	2.897207	3.175716	6.291314	8.740803	8.438468	10.35598	2.17151	0.441708	2.912622	3.191228	1.341289	-4.204317	125.8884	5.94E-08	1.22E-05
1470369	CCL26		40.81738	NM 00607	Homo sapiens	1.526763	-5.077202	5.878047	4.29461	8.422253	6.205976	3.850005	1.908601	5.51641	4.052579	1.432832	-4.596472	178.4352	1.64E-06	0.000104
3170255	AK3L2		83.30297	NM 00100	Homo sapiens	3.941088	5.873556	7.714013	10.11333	8.411027	10.57621	1.957331	-0.513771	2.134189	0.280296	1.090357	-5.513354	1572.081	3.24E-08	8.76E-06
1030424	SNX5		124.6728	NM 15222	Homo sapiens	4.770226	9.201558	7.479412	12.13612	8.39893	12.77454	1.567937	-1.88644	1.760699	-0.43502	1.12294	-5.264879	505.2584	3.30E-09	2.57E-06
5570687		0	41.12643	BX537622	Homo sapiens	6.782203	4.970177	9.481905	6.568271	8.370337	5.932116	1.398057	-5.505954	1.234162	-6.363406	-1.132798	-5.529962	72.79267	1.57E-06	0.000102
5810731	OACT1		104.4209	XM 37180	PREDICTED:	2.804423	4.596351	5.99704	10.37985	8.356992	12.23378	2.138422	1.776764	2.979933	5.157717	1.39352	-3.266547	173.6822	9.04E-09	4.45E-06
60068	LOC441969		37.93144	XM 49778	PREDICTED:	4.949154	2.76793	10.741	6.629643	8.325406	5.392245	2.17027	-2.692239	1.682188	-4.740215	-1.290148	-5.171207	225.2668	2.42E-06	0.000137
430408	KITLG		28.27955	NM 00089	Homo sapiens	7.255431	3.643442	8.401315	4.197065	8.28863	4.09319	1.157935	-6.366955	1.142404	-6.662064	-1.013595	-5.663893	92.02775	1.12E-05	0.000402
2470019	psiTPTE22		39.73626	NR 00159	Homo sapiens	2.715856	-0.763249	8.719063	6.257214	8.237832	5.946395	3.210429	0.559243	3.033236	-0.005174	-1.058417	-5.636228	1023.337	1.89E-06	0.000114
4150224	MMP9		21.23234	NM 00499	Homo sapiens	1.702373	-5.101416	4.371457	0.285803	8.197832	3.587203	2.567861	-2.521093	4.815533	1.049351	1.875309	-3.801496	137.3715	4.73E-05	0.001175
3460026	PPP2R2D		79.85564	NM 01846	Homo sapiens	6.569391	7.996808	10.31278	10.42457	8.195654	9.305299	1.569822	-3.580771	1.247552	-5.931159	-1.258323	-4.852349	108.7073	4.11E-08	9.95E-06
4230014	TGIF		40.18448	NM 00324	Homo sapiens	6.045924	4.418026	9.372052	6.574498	8.159542	5.864821	1.550144	-4.861609	1.349594	-5.947806	-1.1486	-5.4969	124.1529	1.78E-06	0.00011
5550196	RYR2		44.87505	NM 00103	Homo sapiens	1.674637	-4.098495	5.788054	5.04908	8.119976	6.898322	3.456303	1.892224	4.848797	4.082208	1.402885	-4.555409	85.832	9.83E-07	7.51E-05
4780523	LOC649561		46.26851	XM 93863	PREDICTED:	6.867597	5.695099	9.345541	7.213262	8.064407	6.456271	1.360817	-5.537935	1.174269	-6.50108	-1.158863	-5.450994	177.5346	8.33E-07	6.66E-05
2940672	POU5F1		62.82877	NM 20328	Homo sapiens	3.365763	3.610821	6.943004	8.371401	8.044933	9.164253	2.062832	-0.757454	2.390226	0.543442	1.158711	-5.317548	26214.22	1.56E-07	2.26E-05
2850059	PMAIP1		64.47523	NM 02112	Homo sapiens	3.128734	2.309766	9.725829	9.28511	7.9907	8.296835	3.108551	2.307839	2.553972	0.520287	-1.217143	-5.143203	962.3673	1.35E-07	2.08E-05
5080719	LOC441244		42.98787	XM 94365	PREDICTED:	6.481829	4.735591	11.04334	7.274098	7.989891	5.697739	1.703738	-4.228678	1.23266	-6.367984	-1.382164	-4.818344	184.4773	1.24E-06	8.73E-05
4640368	CYP26A1		46.37797	NM 00078	Homo sapiens	8.567202	6.134051	11.40308	7.435893	7.980035	5.710083	1.331016	-5.761296	-1.07358	-6.735368	-1.428952	-4.649653	513.4547	8.23E-07	6.63E-05
360170	DHX37		30.43842	NM 03265	Homo sapiens	3.129155	-0.123176	6.793682	4.444254	7.969937	5.244809	2.171092	-2.650541	2.546993	-1.680413	1.173139	-5.462467	104.8277	7.67E-06	0.000307
3370279	PRDM14		24.82837	NM 02450	Homo sapiens	3.40663	-0.689629	8.215405	3.932864	7.964798	3.742008	2.411593	-2.748711	2.338029	-3.171853	-1.031464	-5.659211	393.7642	2.17E-05	0.000658
1190196	NFKBIB		12.04596	NM 00250	Homo sapiens	2.095862	-4.760832	3.897469	-2.046228	7.926311	1.311276	1.859602	-5.10906	3.781886	-1.947328	2.033707	-4.082063	57.57835	0.00066	0.00859
1820273	LOC647195		41.06021	XM 93023	PREDICTED:	2.814088	-0.165906	8.338448	6.48384	7.904617	6.184978	2.963109	0.322613	2.808945	-0.245292	-1.054883	-5.63728	43.37954	1.58E-06	0.000103
3310168	LRAT		32.57927	NM 00474	Homo sapiens	1.801367	-4.279514	6.82486	4.152113	7.884344	4.865786	3.788712	0.988379	4.376868	1.764409	1.155239	-5.508769	81.31855	5.39E-06	0.000238
670148	MAD2L1BP		28.26337	NM 01462	Homo sapiens	6.421593	3.334741	8.064458	4.319751	7.883976	4.169511	1.255835	-6.142378	1.227729	-6.478657	-1.022892	-5.661621	243.0038	1.12E-05	0.000403
7100181	LOC643224		18.08101	XM 92823	PREDICTED:	3.759739	-1.66281	10.26851	2.818573	7.879918	1.612975	2.731175	-3.176637	2.095868	-4.730111	-1.303123	-5.393966	144.5016	0.000103	0.002114
4490192	LOC642829		41.03079	XM 92623	PREDICTED:	4.690022	3.355617	8.977674	6.820285	7.843783	6.114633	1.914207	-3.12793	1.67244	-4.425165	-1.144559	-5.491331	504.6256	1.59E-06	0.000103
2100458		0	43.63665	CR600638	full-length cDN	1.674161	-4.11911	5.847411	5.064289	7.83408	6.664874	3.49274	1.928181	4.679405	3.815393	1.339752	-4.820732	36.0464	1.14E-06	8.27E-05
6550520	LYSMD2		95.09204	NM 15337	Homo sapiens	2.040787	0.550375	6.218274	9.836981	7.816221	11.12947	3.046999	4.641649	3.830004	6.544525	1.256976	-4.527352	241.214	1.54E-08	5.50E-06
2470066	KIF23		48.87406	NM 13855	Homo sapiens	6.810811	6.423159	7.453531	6.896623	7.782624	7.105482	1.094368	-6.409492	1.142687	-6.555565	1.044153	-5.643168	67.69169	6.19E-07	5.57E-05
3520605	SLC39A3		36.43571	NM 14456	Homo sapiens	4.161404	2.866666	6.214905	5.180499	7.782441	6.384267	1.493463	-4.911688	1.870148	-3.39709	1.252222	-5.17055	142.131	2.99E-06	0.000158
1510132		0	33.87047	BX108554	BX108554 So	2.131786	-2.683867	6.304327	4.469457	7.733172	5.537081	2.957298	-0.08855	3.627555	1.199335	1.226645	-5.30978	161.2299	4.39E-06	0.000207
2750397	LOC647415		20.40448	XM 93648	PREDICTED:	2.7349	-1.758926	3.992746	0.409289	7.713478	4.080048	1.459924	-5.534725	2.820387	-1.627216	1.931873	-3.386673	99.44573	5.75E-05	0.001361
3710017	LRFN5		13.16788	NM 15244	Homo sapiens	1.402187	-6.206944	2.524088	-4.107544	7.686894	1.453499	1.800107	-5.1671	5.482075	0.123546	3.045415	-2.299025	61.60359	0.000446	0.006359
7040367	KIAA1161		21.99402	NM 02070	Homo sapiens	2.367329	-3.311208	7.457109	2.897456	7.68266	2.999126	3.150009	-1.453203	3.245285	-1.472339	1.030246	-5.66022	95.04374	3.98E-05	0.001029
5690201	F2RL1		56.16314	NM 00524	Homo sapiens	4.826347	5.124769	8.864483	8.519044	7.681017	7.769579	1.836686	-2.595484	1.591476	-4.172496	-1.154077	-5.39642	1262.593	2.90E-07	3.38E-05
4830086	SLC38A5		204.9824	NM 03351	Homo sapiens	1.426089	-1.877548	4.96768	12.45782	7.593998	14.94662	3.483428	9.239076	5.325051	12.46187	1.528681	-0.552386	250.9697	1.89E-10	5.00E-07
3940681	BANF1		44.02852	NM 00386	Homo sapiens	5.028295	4.690782	7.06096	6.580542	7.572768	6.936542	1.404245	-5.177824	1.506031	-4.905956	1.072484	-5.608122	336.7069	1.09E-06	8.06E-05
5810176	CRK		91.053	NM 01682	Homo sapiens	5.74489	8.581501	8.522236	10.9369	7.568375	10.33737	1.483446	-3.586089	1.31741	-5.161692	-1.126033	-5.366826	122.402	1.96E-08	6.46E-06
6270181	PIK3C2A		41.12027	NM 00264	Homo sapiens	6.377217	4.817227	9.69688	6.858789	7.566263	5.59635	1.52055	-4.956282	1.186452	-6.49382	-1.281594	-5.133261	149.1899	1.57E-06	0.000102
3370470	KIAA0319L		22.55559	NM 02487	Homo sapiens	4.51224	0.886969	7.459132	3.339018	7.56597	3.367515	1.653088	-5.045029	1.676766	-5.225075	1.014323	-5.663836	214.7325	3.51E-05	0.000937
70605	IDH1		65.96403	NM 00589	Homo sapiens	6.780404	7.801712	8.10454	8.854012	7.564239	8.482937	1.195289	-5.989102	1.115603	-6.580247	-1.071428	-5.591585	1126.465	1.19E-07	1.94E-05
4220014	STEAP1		64.40507	NM 01244	Homo sapiens	5.611561	6.984042	7.63388	8.779766	7.558089	8.720959	1.360384	-5.009269	1.346878	-5.350894	-1.010028	-5.663417	243.8281	1.36E-07	2.08E-05
4540243	GABRB3		44.81412	NM 00081	Homo sapiens	2.476805	-0.058857	5.598424	5.813621	7.550898	7.51142	2.26034	-0.768357	3.048644	1.648388	1.348754	-4.616923	2289.913	9.90E-07	7.53E-05
6660470	MAEA		32.05402	NM 00101	Homo sapiens	3.832563	1.574827	7.059572	5.033559	7.545205	5.352547	1.841997	-3.678902	1.96871	-3.41074	1.068791	-5.626555	163.8453	5.86E-06	0.000253
2760128	C1orf108		78.20431	XM 94112	PREDICTED:	4.884252	7.25915	7.550592	9.92734	7.53482	9.923961	1.545905	-3.278241	1.542676	-3.534391	-1.002093	-5.664946	1253.232	4.62E-08	1.06E-05
4040538	PPAP2C		62.41909	NM 17754	Homo sapiens	2.047467	-0.658881	5.196238	7.038447	7.531423	9.234349	2.537887	1.580553	3.678411	4.662333	1.449399	-3.665898	157.9685	1.62E-07	2.33E-05
6620609	CD1D		129.823	NM 00176	Homo sapiens	6.061976	10.39261	9.199604	12.96291	7.502516	11.99046	1.517592	-2.466783	1.237635	-5.426708	-1.226203	-4.557276	744.4527	2.61E-09	2.19E-06
2030072	GABRB3		87.40811	XM 93725	PREDICTED:	3.064628	4.371073	7.560299	10.55302	7.495967	10.52288	2.466955	2.368946	2.445963	2.18925	-1.008582	-5.663372	1098.61	2.47E-08	7.71E-06
4810139	ILF3		57.21908	NM 15346	Homo sapiens	7.533767	7.139886	9.710374	8.45254	7.495116	7.126333	1.288914	-5.697856	-1.005157	-6.784671	-1.29556	-4.905687	292.538	2.62E-07	3.20E-05
3930037	ZNF488		35.9787	NM 15303	Homo sapiens	1.808958	-3.594692	4.879752	3.70324	7.469482	6.152562	2.697548	-0.195841	4.129162	2.765458	1.530709	-4.088503	285.0122	3.20E-06	0.000166
2690110	NR3C1		33.67628	NM 00102	Homo sapiens	3.524181	1.511546	6.205911	4.920197	7.465432	5.906269	1.760951	-3.764592	2.118345	-2.50097	1.202955	-5.341453	86.26362	4.53E-06	0.000213
6100615	HMGN2		58.49609	NM 00551	Homo sapiens	6.474546	7.029127	8.169514	8.321061	7.445901	7.818494	1.26179	-5.7288	1.150027	-6.486562	-1.097183	-5.546192	121.4368	2.32E-07	2.91E-05
2640630		0	16.01303	U56251	Human HeLa	5.356411	0.017326	8.652222	1.965409	7.428549	1.232239	1.615302	-5.597681	1.386852	-6.33725	-1.164726	-5.575651	456.5281	0.000183	0.003222
4070072	TNMD		51.6924	NM 02214	Homo sapiens	3.374632	2.517751	8.185074	7.979285	7.416871	7.443501	2.425472	-0.120776	2.197831	-1.192797	-1.103575	-5.539366	666.0405	4.56E-07	4.51E-05
7050609	PLEKHG6		49.53813	NM 01817	Homo sapiens	2.672568	0.956125	6.175753	6.845795	7.388191	7.843893	2.310793	-0.256376	2.764453	1.19636	1.196322	-5.229634	211.2251	5.75E-07	5.27E-05
4640239	LOC642954		25.86598	XM 92710	PREDICTED:	4.432925	1.131875	8.591165	4.437618	7.38055	3.649055	1.938035	-3.995411	1.664939	-5.150709	-1.164028	-5.518954	81.68474	1.77E-05	0.000567
5550747	LOC442425		58.01373	NM 00101	Homo sapiens	2.684974	1.128363	7.881968	8.366623	7.374464	7.999606	2.935585	1.955513	2.746568	1.274294	-1.068819	-5.600811	113.92	2.42E-07	3.03E-05

7330408	GULP1		117.0804	NM	01631	Homo sapiens	5.694125	9.946014	8.035791	12.18503	7.336313	11.74588	1.411242	-3.539652	1.2884	-4.979026	-1.095345	-5.428877	339.0198	4.72E-09	3.06E-06
3180452	PARP12		43.67273	NM	02275	Homo sapiens	2.172343	-1.011808	4.461256	4.6721	7.297758	7.679904	2.053661	-1.425136	3.359395	2.723737	1.635808	-3.105001	157.8175	1.14E-06	8.24E-05
1230072	RNU30		66.92977	NR	00256	Homo sapiens	4.54726	6.257864	7.167758	9.060419	7.282852	9.146609	1.576281	-3.329706	1.601591	-3.385901	1.016057	-5.660412	370.6702	1.10E-07	1.86E-05
1260041	CDC2L1		43.20054	NM	03349	Homo sapiens	4.549662	4.203532	6.829757	6.548528	7.276685	6.874763	1.501157	-4.635156	1.59939	-4.358239	1.065438	-5.616953	165.2843	1.21E-06	8.57E-05
10138	CPXM2		124.7537	NM	19814	Homo sapiens	3.239489	6.96733	6.915412	12.34026	7.250472	12.64215	2.134723	2.715914	2.238153	3.215818	1.048451	-5.587572	334.0208	3.28E-09	2.57E-06
1710180	FAM7A1		63.97162	XM	93125	PREDICTED:	2.699735	2.025469	6.806379	8.599564	7.227022	8.929774	2.521129	1.456755	2.676938	1.901096	1.061801	-5.601951	103.2153	1.41E-07	2.14E-05
2120379	ECD		37.88753	NM	00726	Homo sapiens	4.932032	3.714266	7.895435	6.232491	7.22514	5.736862	1.600849	-4.486271	1.464942	-5.361912	-1.092773	-5.588495	276.9397	2.43E-06	0.000138
5270376	EFCBP1		31.42517	NM	02235	Homo sapiens	2.667737	-1.012883	6.761301	4.778737	7.218011	5.096064	2.534471	-1.27765	2.705668	-0.977533	1.067548	-5.628041	74.09729	6.50E-06	0.000273
7610546	HMFN0839		35.71726	NM	03271	Homo sapiens	2.028597	-2.455174	4.834573	3.959805	7.207877	6.295116	2.38321	-0.959519	3.553134	2.009525	1.490902	-4.174504	63.63093	3.32E-06	0.00017
1980719	PTPN6		26.7382	NM	08054	Homo sapiens	3.081859	-0.303573	6.093843	3.752154	7.207229	4.626037	1.977327	-3.379734	2.338598	-2.375106	1.182707	-5.445829	245.4368	1.49E-05	0.000496
2470131	ABI3		16.67421	NM	01642	Homo sapiens	2.490646	-2.995773	4.641008	0.406885	7.205752	2.698212	1.863375	-4.535461	2.893126	-2.160609	1.552627	-4.709441	100.6195	0.000151	0.002803
1190278	FKSG30		75.60598	NM	00101	Homo sapiens	5.105405	7.477663	7.38923	9.750165	7.203473	9.615762	1.447335	-4.045855	1.41095	-4.568178	-1.025787	-5.652038	8634.756	5.58E-08	1.21E-05
6560438	USP22		49.34012	XM	94226	PREDICTED:	4.408029	4.704187	6.797728	7.297537	7.192179	7.594472	1.542124	-4.148424	1.631609	-3.865238	1.058027	-5.621027	619.7542	5.88E-07	5.35E-05
1850685	hCAP-H2		13.80871	NM	01455	Homo sapiens	2.68028	-2.882714	3.850648	-1.123974	7.182858	2.127032	1.436659	-5.845776	2.679891	-2.965259	1.865363	-4.066956	59.44343	0.000361	0.005463
1570358	CALB1		36.90126	NM	00492	Homo sapiens	1.357461	-5.759133	4.876554	3.386579	7.141063	5.57963	3.592408	1.638991	5.260602	3.990948	1.464367	-4.431096	62.00811	2.80E-06	0.00015
5490189	MAL2		46.45526	NM	05288	Homo sapiens	2.179342	-0.876123	5.054565	5.660031	7.13205	7.707526	2.319308	-0.181693	3.272571	2.650976	1.411012	-4.206473	634.2412	8.15E-07	6.61E-05
3130367	CRB3		17.23833	NM	13916	Homo sapiens	1.070565	-6.665735	3.458094	-1.960329	7.093772	1.77165	3.230159	-1.883898	6.626195	1.682113	2.051353	-3.740566	131.4386	0.000129	0.002509
70347	CCT6A		67.11389	NM	00176	Homo sapiens	5.600115	7.025798	8.640822	9.478719	7.081724	8.421696	1.542972	-3.796278	1.264568	-5.846591	-1.220158	-5.053623	1037.719	1.08E-07	1.84E-05
3830072	CBLC		43.76031	NM	01211	Homo sapiens	1.716365	-3.353976	4.191962	4.111769	7.076107	7.380301	2.442349	0.081167	4.12273	4.12148	1.688018	-2.902296	59.08501	1.13E-06	8.22E-05
1660575	NLGN4X		133.9095	NM	02074	Homo sapiens	3.714361	8.380747	7.267609	12.93756	7.068003	12.84304	1.956624	1.908666	1.902885	1.417811	-1.028241	-5.636293	2097.875	2.19E-09	1.97E-06
5670343	SKIL		32.40899	NM	00541	Homo sapiens	2.717187	-0.45966	6.002747	4.677931	7.063201	5.556483	2.209176	-1.978253	2.599453	-0.875808	1.176662	-5.414834	268.3312	5.53E-06	0.000242
7210167	APP		45.33744	NM	00048	Homo sapiens	2.631638	0.945808	5.107704	5.826872	7.057007	7.750421	1.940884	-1.813581	2.681603	1.057655	1.38164	-4.336551	224.0656	9.30E-07	7.26E-05
3930497	LOC644739		31.37588	XM	93367	PREDICTED:	5.601959	3.436728	8.100873	5.245307	7.032179	4.479496	1.446079	-5.421438	1.255307	-6.342175	-1.151972	-5.508729	392.6139	6.55E-06	0.000274
1070138	HNRPA3		37.46537	NM	19424	Homo sapiens	4.821721	3.954354	6.706857	5.774243	7.028434	6.004306	1.390968	-5.352523	1.457661	-5.294451	1.047948	-5.641848	1141.756	2.58E-06	0.000143
5360347	QSCN6		20.85667	NM	00282	Homo sapiens	2.826691	-1.511123	5.199668	2.03558	7.015486	3.633704	1.83949	-4.225294	2.481873	-2.469233	1.349218	-5.093833	131.748	5.17E-05	0.001255
2490390	EPB41L4B		23.10067	NM	01842	Homo sapiens	2.11009	-3.352438	5.113864	2.19201	6.999544	3.892137	2.423528	-2.254379	3.317177	-0.312938	1.368739	-5.009247	57.94497	3.12E-05	0.000855
1780152	CEACAM1		47.86924	NM	00102	Homo sapiens	1.738141	-3.143858	4.967958	5.44983	6.964875	7.475067	2.858201	1.568437	4.007082	4.087459	1.401959	-4.275768	55.24037	6.93E-07	6.01E-05
4200605	MAP2K6		15.97395	NM	00275	Homo sapiens	6.989437	0.840747	9.865202	2.068345	6.952928	0.496897	1.411444	-6.067315	-1.005251	-6.784924	-1.418856	-5.251437	83.70734	0.000185	0.003251
270167	TBC1D23		44.62691	NM	01830	Homo sapiens	4.223936	3.775559	7.564246	7.170584	6.952633	6.690453	1.790805	-3.098939	1.646008	-4.083172	-1.087968	-5.579232	97.00417	1.01E-06	7.66E-05
5960168	LOC652183		28.68954	XM	94154	PREDICTED:	1.31606	-5.992271	3.286719	0.265307	6.936009	4.92564	2.497392	-1.359498	5.270282	3.541472	2.110314	-2.294379	62.11379	1.04E-05	0.000381
6860039	AGPAT2		23.14732	NM	00641	Homo sapiens	3.080678	-0.819816	6.252546	3.223389	6.935204	3.735378	2.029601	-3.520142	2.251194	-3.045569	1.109181	-5.590833	234.8993	3.09E-05	0.00085
6980541	GRB7		41.72548	NM	00531	Homo sapiens	1.901693	-2.59257	5.04293	4.985494	6.922489	6.859476	2.651811	0.493886	3.640172	2.871052	1.372712	-4.536866	45.7937	1.45E-06	9.68E-05
2120626	SERPINB9		19.15157	NM	00415	Homo sapiens	2.215739	-3.426768	5.016145	1.348777	6.913288	3.041126	2.26387	-3.14374	3.120082	-1.304756	1.378207	-5.075013	80.36169	7.83E-05	0.001719
4590474	FUT1		31.11809	NM	00014	Homo sapiens	2.21894	-1.661389	3.882069	2.499813	6.905216	6.094188	1.749515	-3.557431	3.111944	1.045451	1.778746	-2.94879	43.42626	6.84E-06	0.000281
5420358	LOC654127		12.18021	XM	93972	PREDICTED:	3.703089	-2.662564	9.340179	1.052388	6.902956	-0.264977	2.522267	-4.196959	1.864108	-5.613077	-1.353069	-5.400528	54.31268	0.000629	0.00828
770717	SLCO4C1		38.67155	NM	18099	Homo sapiens	2.432071	-0.441346	5.243776	5.109988	6.892971	6.706579	2.156095	-1.391755	2.834198	0.801107	1.314505	-4.819492	71.26613	2.18E-06	0.000127
2320653		0	11.59669	DB369750		DB369750 NT	1.993477	-4.876776	3.996285	-1.675761	6.853828	0.957906	2.00468	-4.690014	3.438127	-2.211447	1.71505	-4.627405	55.79446	0.000778	0.009688
7210408	COBL		57.93144	NM	01519	Homo sapiens	2.405041	0.994446	5.602464	7.523248	6.83729	8.700883	2.329467	0.763998	2.842899	2.521522	1.220408	-5.000165	987.308	2.44E-07	3.04E-05
6510544		0	36.92105	BX089938		BX089938 So	2.791129	-0.655742	9.386771	6.477933	6.833421	4.828369	3.363073	0.775366	2.448264	-1.720537	-1.373656	-4.854018	163.6879	2.79E-06	0.00015
3850349	TBRG4		24.52128	NM	00474	Homo sapiens	3.62593	0.390418	6.736167	3.801191	6.814362	3.82239	1.857776	-4.029322	1.879342	-4.194705	1.011608	-5.664074	85.70069	2.31E-05	0.000688
3420372	FAF1		22.20152	NM	00705	Homo sapiens	4.687366	1.658939	6.013018	2.84029	6.77424	3.437617	1.282814	-6.068393	1.445213	-5.839566	1.126596	-5.570355	318.9336	3.80E-05	0.00099
540142		0	47.14236	CX165253		HESC2 26 E	5.435588	5.376083	8.019797	7.497741	6.753947	6.54811	1.475424	-4.746851	1.242542	-6.17132	-1.187424	-5.316774	88.06459	7.53E-07	6.31E-05
1230196	SLC25A3		52.75717	NM	21361	Homo sapiens	4.574092	5.460235	6.632544	7.717074	6.753914	7.806074	1.450024	-4.519612	1.476558	-4.598849	1.018299	-5.65997	108.9958	4.08E-07	4.22E-05
2570014	LOC651106		48.0605	XM	94023	PREDICTED:	7.920026	7.087084	7.417822	6.771117	6.734289	6.21611	-1.067702	-6.459858	-1.176074	-6.454727	-1.1015	-5.55848	72.38142	6.78E-07	5.95E-05
1990646	CGI-96		46.01418	NM	01570	Homo sapiens	2.530731	1.094412	4.302395	5.270767	6.729801	8.11969	1.70006	-2.803521	2.659232	1.500751	1.564199	-3.157567	183.6166	8.58E-07	6.77E-05
1190053	GIYD2		24.35314	NM	17804	Homo sapiens	3.452742	0.434247	5.67719	3.298788	6.694374	4.178283	1.644255	-4.665223	1.938857	-3.780071	1.17917	-5.455533	200.6356	2.39E-05	0.000703
4490477	PCGF6		35.53251	NM	00101	Homo sapiens	7.138339	5.018162	8.428757	5.766821	6.672904	4.515273	1.180773	-6.26149	-1.06975	-6.742269	-1.263132	-5.224694	166.3201	3.42E-06	0.000173
6450296	MYRIP		17.62196	NM	01546	Homo sapiens	5.468765	0.237251	10.47397	2.928247	6.663548	0.859305	1.915234	-4.882616	1.218474	-6.612889	-1.57183	-4.908326	57.21493	0.000117	0.002319
6980301	FLJ20674		73.1404	NM	01908	Homo sapiens	2.079025	0.58115	5.067191	8.31214	6.653034	10.00003	2.437292	2.366049	3.200074	4.864909	1.312963	-4.178579	538.0429	6.71E-08	1.34E-05
2000136	TAGLN2		28.25138	NM	00356	Homo sapiens	2.930727	0.03631	5.24742	3.771636	6.631864	5.100063	1.790484	-3.703691	2.262874	-2.060832	1.263833	-5.169801	526.4025	1.13E-05	0.000403
3420735	PHF15		35.79606	NM	01528	Homo sapiens	2.834649	0.741239	5.273997	5.001478	6.617115	6.327065	1.860547	-2.799975	2.334369	-0.976695	1.254668	-5.083332	838.9473	3.28E-06	0.000169
4570706		0	23.90562	CA433634		UI-H-CO0-atb	2.38536	-2.665328	6.869085	3.554569	6.614905	3.314017	2.879684	-1.271997	2.773126	-1.721622	-1.038425	-5.655476	71.73647	2.63E-05	0.000759
2970520	ZNF278		48.16796	NM	03205	Homo sapiens	2.961098	2.000064	6.390746	7.246638	6.596529	7.411802	2.158236	-0.756114	2.227731	-0.632104	1.0322	-5.650298	82.79797	6.70E-07	5.90E-05
20397	RPL8		56.77088	NM	03330	Homo sapiens	2.592248	2.099337	4.920568	7.135923	6.57795	8.942096	1.898186	-1.052818	2.537547	1.846523	1.336828	-4.245369	1048.202	2.73E-07	3.27E-05
6100170	KIAA1906		26.55404	NM	05290	Homo sapiens	2.895915	-0.201384	5.07744	3.383435	6.532649	4.822051	1.753311	-3.94321	2.255815	-2.206425	1.286603	-5.115276	90.70619	1.55E-05	0.000511
6060390	CDA		16.44672	NM	00178	Homo sapiens	2.851953	-2.47489	6.5991	1.835894	6.52909	1.734169	2.313888	-3.530283	2.28934	-3.826481	-1.010723	-5.664473	480.7675	0.000161	0.002938
4220056	KIAA1804		58.19982	NM	03243	Homo sapiens	2.029752	-0.519949	4.920801	7.002935	6.521615	8.756291	2.424337	1.406956	3.213011	3.875962	1.325316	-4.347094	41.24498	2.38E-07	2.99E-05
6590221	TMPO		70.43946	NM	00327	Homo sapiens	6.143785	8.069806	7.747914	9.483375	6.521351	8.523506	1.261098	-5.512804	1.061455	-6.71286	-1.188084	-5.150681	552.3816	8.28E-08	1.54E-05
4780754	DUSP6		21.7124	NM	00194	Homo sapiens	5.625419	2.039129	7.449554	3.269089	6.520038	2.546913	1.324267	-6.022671	1.159032	-6.643947	-1.142563	-5.562121	124.4547	4.24E-05	0.001085
1440427	LOC643319		31.15587	XM	92798	PREDICTED:	2.821794	0.009297	5.727022	4.631644	6.489425	5.321379	2.029568	-2.532298	2.299752	-1.709155	1.133124	-5.509224	635.6204	6.80E-06	0.000281
2600709	OLFML3		56.50097	NM	02019	Homo sapiens	3.409935	3.590551	7.401981	8.584843	6.485155	7.8313	2.17071	-0.343349	1.901841	-1.882886	-1.141373	-5.390107	2669.047	2.80E-07	3.32E-05
2450546	LOC401321		45.77013	XM	37948	PREDICTED:	1.657469	-3.664063	5.123604	5.518575	6.470463	6.91932	3.091221	2.090092	3.903821	3.77745	1.262873	-4.969552	1456.426	8.83E-07	6.95E-05
7200189	RNF5P1		28.55332	XR	00052	PREDICTED:	5.566837	3.613475	6.384863	4.220946	6.433167	4.224515	1.146946	-6.344037	1.155623	-6.593894	1.007565	-5.664564	530.772	1.07E-05	0.000388
7000170	SLC44A1		24.2887	NM	02210	Homo sapiens	4.650123	1.721692	7.326058	4.008638	6.427375	3.279925	1.575455	-5.109745	1.382195	-6.02514	-1.139821	-5.548349	299.837	2.43E-05	0.00071
5260131	LAD1		26.3385	XM	94266	PREDICTED:	1.40914	-5.554552	2.589171	-1.228494	6.419275	4.912608	1.837412	-3.505228	4.555455	3.037	2.479278	-1.067993	44.55279	1.61E-05	0.000526
4810333	FLJ14712		21.74103	XM	37187	PREDICTED:	2.506698	-2.26004	5.910032	2.855464	6.407589	3.253654	2.357696	-2.541143	2.556187	-2.203958	1.084188	-5.620288	465.8312	4.21E-05	0.001079
6760328	MYLC2PL		9.698425	NM	13840	Homo sapiens	1.381954	-6.289459	2.032802	-5.338397	6.404794	0.138291	1.470962	-5.956588	4.634594	-1.106529	3.150723	-2.407872	55.17437	0.001647	0.017307
4490215	WDR72		28.29412	NM	18275	Homo sapiens	2.648259	-0.697767	5.287017	3.872743	6.404684	4.961793	1.996412	-2.819256	2.41845	-1.488083	1.211398	-5.323904	126.6507	1.12E-05	0.000402
2120278	SMN1		29.32565	NM	02287	Homo sapiens	4.43409	2.190826	7.765555	5.166969	6.389	4.092176	1.75133	-4.186593	1.440882	-5.674699	-1.215457	-5.364308	180.8609	9.30E-06	0.000354
1740240		0	37.96918	DR423590		nav28b10.y1 H	1.851673	-2.93777	4.954299	4.691119	6.386292	6.214713	2.67558	0.427482	3.448931	2.305883	1.289041	-4.935182	37.92305	2.40E-06	0.000137
4610402	JUP		26.76578	NM	00223	Homo sapiens	3.321567	0.809611	5.353967	3.720172	6.382766	4.706079	1.61188	-4.549157	1.921613	-3.470072	1.192156	-5.389332	76.27804	1.48E-05	0.000494
5860717	BTBD1		52.36725	NM	02523	Homo sapiens	5.811734	6.673834	6.8111	7.601554	6.379916	7.208567	1.171957	-6.118002	1.097765	-6.643815	-1.067585	-5.602686	112.8033	4.25E-07	4.29E-05
2680156	ATP5G1		54.00761	NM	00517	Homo sapiens	2.115555	-0.149748	4.693177	6.575659	6.355566	8.498962	2.218414	0.442212	3.004208	3.204954	1.354214	-4.191617	619.8437	3.59E-07	3.93E-05
5340246	CD9		83.67679	NM	00176	Homo sapiens	3.389707	5.869947	6.533182	10.44994	6.352293	10.30007	1.927359	0.206196	1.873995	-0.307736	-1.028476	-5.644615	8164.943	3.16E-08	8.64E-06
4490053	SNAP23		50.25925	NM	00382	Homo sapiens	5.685962	6.471407	6.559247	7.304225	6.320839	7.070078	1.153586	-6.195915	1.111657	-6.606498	-1.037718	-5.645276	116.9513	5.32E-07	5.00E-05
1400296	MFAP3L		31.14583	NM	02164	Homo sapiens	1.481494	-5.220099	4.532785	2.911652	6.317095	4.893778	3.059604	0.502277	4.264003	2.677881	1.393645	-4.704344	92.80859	6.81E-06	0.000281
870392	LOC653450		28.52166	XM	37055	PREDICTED:	5.842927	3.754029	6.679219	4.33193	6.31029	3.981561	1.143129	-6.356296	1.079988	-6.731758	-1.058465	-5.639037	106.7352	1.07E-05	0.000389

1780307	EPB41L4B		46.32303	NM	01842	Homo sapiens	1.514397	-4.146762	3.405678	3.468561	6.301173	7.728607	2.248868	0.050565	4.160847	5.115336	1.850196	-1.702535	69.31824	8.28E-07	6.64E-05
5890707	ITGB1		53.17002	NM	13337	Homo sapiens	5.776748	6.565839	7.396756	7.976878	6.299579	7.054286	1.280436	-5.605859	1.090506	-6.664767	-1.174167	-5.305581	952.6441	3.91E-07	4.15E-05
5900056	TP53		27.1816	NM	00054	Homo sapiens	4.586004	2.418719	6.666167	4.387763	6.288703	4.028683	1.453589	-5.361395	1.371282	-5.933379	-1.060023	-5.637322	480.8645	1.37E-05	0.000465
6770500	RPS26		59.87481	NM	00102	Homo sapiens	3.539904	5.165788	5.180766	7.879443	6.271636	9.067523	1.463533	-3.826285	1.771697	-1.785723	1.210561	-4.951358	343.8349	2.04E-07	2.67E-05
6220242	PGAM1		33.28661	NM	00262	Homo sapiens	4.714971	3.381398	7.10881	5.615056	6.247187	4.865498	1.50771	-4.929269	1.324968	-5.981291	-1.137922	-5.505972	2100.6	4.81E-06	0.00022
5550082	CHST9		41.98329	NM	03142	Homo sapiens	1.574577	-4.143572	4.40977	4.511106	6.210536	6.677339	2.800606	1.290335	3.944256	3.84954	1.408358	-4.274156	421.8603	1.41E-06	9.46E-05
2190632	LOC650037		18.33735	XM	93912	PREDICTED:	1.9152	-3.949388	3.246025	-0.575595	6.185225	3.369501	1.694875	-4.592984	3.229544	-0.37988	1.905477	-3.300809	107.2563	9.64E-05	0.002013
7550736	HSPBP1		14.07645	XM	93800	PREDICTED:	2.266487	-3.778631	4.845093	0.258812	6.184216	1.497321	2.13771	-3.952501	2.728546	-2.788791	1.276388	-5.377139	161.0374	0.000331	0.005116
4810632	NOC4L		21.98251	NM	02407	Homo sapiens	2.555504	-1.450903	4.29613	1.918586	6.176218	4.073567	1.681129	-4.406327	2.41683	-1.946415	1.437624	-4.663821	297.9798	3.99E-05	0.001032
290201	PCDH1		24.83554	NM	00258	Homo sapiens	2.428453	-1.892183	5.575027	3.437406	6.172388	3.980262	2.295711	-2.179221	2.541695	-1.626824	1.107149	-5.580083	93.27649	2.17E-05	0.000658
870095	MAP4K3		43.26853	NM	00361	Homo sapiens	5.843641	5.612662	7.436839	6.905802	6.1542	5.819105	1.272638	-5.798318	1.053145	-6.749767	-1.208417	-5.25946	307.1672	1.20E-06	8.51E-05
3400228	SH2D3A		19.70774	NM	00549	Homo sapiens	1.21124	-6.404255	1.852809	-4.592727	6.132748	3.184729	1.529679	-5.232699	5.063196	2.316185	3.309973	-0.294006	37.60104	6.81E-05	0.001551
6370358	GPR176		27.51995	NM	00722	Homo sapiens	1.742497	-4.149211	4.733527	2.932825	6.127462	4.447469	2.716519	-0.581782	3.516483	1.133974	1.294481	-5.091155	46.31725	1.29E-05	0.000447
2000356	HAT1		106.7969	NM	00364	Homo sapiens	5.548683	9.746525	7.883646	12.02702	6.048772	10.57839	1.420814	-3.474935	1.090128	-6.549361	-1.303347	-3.969628	729.198	7.96E-09	4.21E-06
6290370		0	27.65485	AB167288		Homo sapiens	3.889445	2.306071	4.915137	3.645088	6.048657	4.867612	1.263712	-5.933295	1.555146	-4.908553	1.230618	-5.250476	118.2913	1.26E-05	0.000438
7160706	INDO		13.63638	NM	00216	Homo sapiens	4.346175	-1.202082	8.482104	1.631358	6.04854	0.040674	1.951625	-4.917884	1.391693	-6.350608	-1.402339	-5.260965	455.8396	0.000381	0.005694
7650433	GPR64		111.1864	NM	00575	Homo sapiens	4.178311	8.503827	7.618572	12.41654	6.034319	11.14824	1.823362	0.348585	1.444201	-3.199929	-1.26254	-4.170967	684.6438	6.33E-09	3.67E-06
1230386	FAM18B2		42.82676	XM	93692	PREDICTED:	5.000402	5.138785	6.657888	6.776626	6.032553	6.178889	1.331471	-5.442718	1.206414	-6.296758	-1.10366	-5.541196	151.2944	1.26E-06	8.84E-05
1940332	C1orf97		33.92171	NM	03270	Homo sapiens	3.656357	2.654139	5.45623	5.164869	6.022756	5.731205	1.492258	-4.702235	1.647201	-4.131182	1.103831	-5.550477	1379.1	4.36E-06	0.000206
3360592	CXorf15		75.85574	NM	01836	Homo sapiens	2.019752	1.107226	4.163597	8.018208	6.015395	10.51562	2.061439	1.429749	2.978283	5.248248	1.444759	-2.814523	53.6905	5.47E-08	1.20E-05
1240368	FBP1		101.6149	NM	00050	Homo sapiens	1.134769	-6.165434	3.846943	7.7619	6.014342	10.87774	3.390067	6.588653	5.300058	9.89614	1.563408	-1.734068	49.54172	1.06E-08	4.89E-06
4200193	MARVELD3		34.6894	NM	05285	Homo sapiens	-1.181748	-6.387261	2.698664	-0.712049	5.980492	4.754782	3.18914	0.942943	7.067435	5.794614	2.216094	-1.658598	70.10038	3.88E-06	0.000189
450463	DSG2		47.39439	NM	00194	Homo sapiens	3.559263	3.577259	6.615672	7.573758	5.980184	6.961132	1.858719	-2.141049	1.680175	-3.380478	-1.106265	-5.51387	449.9002	7.31E-07	6.20E-05
6520468	ANK3		30.29948	NM	00114	Homo sapiens	2.683766	0.356535	4.15021	3.488798	5.9595	5.803424	1.546413	-4.372175	2.220573	-1.363585	1.435951	-4.282508	102.03	7.85E-06	0.000311
1010358	ITGB1BP3		57.06721	NM	01444	Homo sapiens	1.295981	-5.405992	3.702224	4.910857	5.958461	8.209935	2.856697	2.912916	4.597647	6.563649	1.609428	-2.588352	1269.097	2.65E-07	3.24E-05
4590561	SCGB3A2		21.48696	NM	05402	Homo sapiens	3.791748	0.796957	5.646845	2.972014	5.946192	3.221016	1.489246	-5.331488	1.568193	-5.29684	1.053011	-5.645278	2848.998	4.46E-05	0.001127
1070707	LOC644122		27.19391	XM	93473	PREDICTED:	2.623757	-0.49944	4.736842	3.541079	5.925984	4.885481	1.805366	-3.447848	2.258587	-1.802122	1.251041	-5.173111	202.7307	1.37E-05	0.000465
2900424	UTF1		13.92012	NM	00357	Homo sapiens	2.409253	-3.465412	5.292284	0.671876	5.915524	1.203555	2.196649	-3.833011	2.455335	-3.441089	1.117764	-5.604602	211.3233	0.000348	0.005311
3840500	PIM2		47.76749	NM	00687	Homo sapiens	2.828329	2.477062	4.986411	6.72577	5.91481	7.799135	1.763024	-2.177259	2.091274	-0.53148	1.186186	-5.171924	4060.528	7.01E-07	6.05E-05
3460307	RIF1		49.95297	NM	01815	Homo sapiens	5.176842	6.186228	6.45117	7.499095	5.910385	6.960168	1.246159	-5.746103	1.141697	-6.492432	-1.091497	-5.54953	77.71578	5.50E-07	5.11E-05
4570520	RPL6		53.88804	NM	00102	Homo sapiens	3.144862	3.784257	5.422542	7.71264	5.902653	8.231964	1.724254	-2.172285	1.87692	-1.412773	1.08854	-5.528117	3069.712	3.63E-07	3.93E-05
4640020	ASB3		24.85333	NM	01611	Homo sapiens	5.626077	2.816849	7.454274	4.12119	5.89824	2.83261	1.324951	-5.93478	1.048375	-6.767761	-1.263813	-5.299072	164.9679	2.16E-05	0.000656
4150072		0	68.60094	AW075801		xa85f07.x1 NC	2.618556	3.077965	5.704373	9.118475	5.878223	9.304261	2.178443	1.221456	2.244834	1.426038	1.030477	-5.643837	461.3429	9.59E-08	1.72E-05
6220537	CAPG		43.82534	NM	00174	Homo sapiens	2.657443	1.920583	4.15347	5.453584	5.872125	7.766569	1.562957	-3.476869	2.20969	0.05414	1.413788	-3.890511	125.9931	1.12E-06	8.16E-05
1660746	QKI		16.08176	XM	94222	PREDICTED:	6.73046	1.372458	7.876362	1.853196	5.860527	0.39659	1.170256	-6.402	-1.148439	-6.696902	-1.343968	-5.31563	387.005	0.000179	0.003179
3400360	SHMT1		36.55671	NM	00416	Homo sapiens	4.089669	3.414406	6.386485	6.076095	5.858915	5.540949	1.561614	-4.3464	1.432614	-5.274671	-1.090046	-5.577952	92.49862	2.94E-06	0.000156
3310241	LOC115749		11.24191	XM	05668	PREDICTED:	2.387153	-3.923648	4.96948	-0.391635	5.854143	0.37815	2.08176	-4.446935	2.452353	-3.870941	1.178019	-5.555439	66.28572	0.000889	0.010716
1300204	ZNF165		45.62832	NM	00344	Homo sapiens	1.521918	-4.092555	3.88151	4.512158	5.848293	7.289823	2.550407	1.245502	3.842712	4.550406	1.506706	-3.479228	350.2977	8.98E-07	7.03E-05
3290136	DNAJC11		64.79493	NM	01819	Homo sapiens	2.70197	3.069471	5.888691	8.957544	5.821551	8.883409	2.179406	0.947708	2.154558	0.680774	-1.011533	-5.662134	144.1244	1.32E-07	2.07E-05
3360465	UNC5D		40.7919	NM	08087	Homo sapiens	2.378478	0.216934	4.82908	5.707225	5.813931	6.873543	2.030323	-1.209259	2.444391	0.413782	1.203942	-5.16439	212.1647	1.64E-06	0.000104
2650079	ACTB		65.2452	NM	00110	Homo sapiens	4.254475	6.716086	5.940925	8.95867	5.803936	8.811312	1.396394	-4.277895	1.364196	-4.78479	-1.023603	-5.653157	13358.71	1.27E-07	2.02E-05
3400091	HHLA3		66.32342	NM	00103	Homo sapiens	6.027613	8.455708	5.877478	8.449612	5.803021	8.362374	-1.025544	-6.500263	-1.038703	-6.752626	-1.012831	-5.661763	95.11005	1.16E-07	1.91E-05
2350646	PDE7A		31.49481	NM	00260	Homo sapiens	3.938689	3.33485	4.348159	3.851018	5.799127	5.678303	1.103961	-6.382799	1.472349	-5.009259	1.333697	-4.739218	82.29265	6.42E-06	0.000271
4060605	CD44		13.61423	NM	00100	Homo sapiens	1.230019	-6.46473	3.224288	-2.198198	5.794844	0.980743	2.621332	-2.908124	4.711182	0.159394	1.797248	-4.245642	336.4058	0.000384	0.00572
3990615	LAMC2		28.26078	NM	00556	Homo sapiens	1.060831	-6.652432	2.830398	-0.512246	5.790652	4.336273	2.668094	-0.560911	5.458598	4.151347	2.045879	-2.299854	59.17666	1.12E-05	0.000403
2340703	MTMR12		28.79967	NM	01906	Homo sapiens	2.852575	0.353474	5.101476	4.251328	5.753584	4.954441	1.788375	-3.413222	2.016979	-2.615037	1.127827	-5.511213	125.2188	1.02E-05	0.000378
4810246	C1orf80		42.6716	NM	02283	Homo sapiens	4.222035	3.869441	7.728756	7.392508	5.749698	5.692237	1.830576	-2.847298	1.361831	-5.582968	-1.344202	-4.681834	70.5251	1.29E-06	8.93E-05
5220240	HRASLS3		47.16973	NM	00706	Homo sapiens	1.978105	-1.020184	4.379178	5.878979	5.746261	7.673435	2.213824	0.26331	2.904932	2.721409	1.312178	-4.490506	4848.39	7.51E-07	6.30E-05
4920551	LOC399942		18.7835	XM	93447	PREDICTED:	3.7559	-0.065097	6.539171	2.818518	5.724244	2.059642	1.74104	-4.721564	1.524067	-5.675356	-1.142364	-5.557908	1606.949	8.59E-05	0.001849
2680255	HSD11B2		66.84852	NM	00019	Homo sapiens	1.536703	-3.551258	5.667466	8.147425	5.723681	8.198293	3.688068	5.156488	3.72465	5.274749	1.009919	-5.663102	386.6364	1.11E-07	1.87E-05
290687	TAF1L		35.55837	NM	15380	Homo sapiens	7.732718	5.114872	9.777929	6.162004	5.7162	3.337357	1.264488	-6.047735	-1.352772	-6.022231	-1.710565	-3.785573	114.621	3.40E-06	0.000173
60255	MAP7		37.27008	NM	00398	Homo sapiens	1.661841	-3.72666	4.501781	4.465666	5.688516	5.95221	2.708912	0.858843	3.42302	2.623213	1.263614	-4.989904	1378.666	2.65E-06	0.000146
4480193	FAM46B		70.86219	NM	05294	Homo sapiens	2.543035	3.482726	4.871003	8.883225	5.645679	9.854941	1.915429	0.331886	2.220055	1.966176	1.159038	-5.103151	3100.231	8.01E-08	1.51E-05
2690458	LOC653337		26.38781	XM	93282	PREDICTED:	3.512283	1.686068	4.833049	3.636856	5.640476	4.556776	1.376042	-5.455245	1.605929	-4.633515	1.167064	-5.426517	74.56918	1.60E-05	0.000524
840717	ARL5		25.5422	NM	01209	Homo sapiens	4.219548	2.356955	5.502795	3.8049	5.629442	3.899906	1.30412	-5.846192	1.334134	-5.997031	1.023015	-5.660361	154.4128	1.88E-05	0.000593
3890008	IL23A		24.43297	NM	01658	Homo sapiens	2.981596	-0.447669	6.425288	4.162337	5.587975	3.330085	2.154982	-2.693442	1.874156	-3.948146	-1.149842	-5.508792	128.1647	2.36E-05	0.000695
3060100	CLDN10		11.69784	NM	00698	Homo sapiens	2.474076	-3.610752	5.188802	0.055453	5.586281	0.379759	2.097268	-4.313404	2.257926	-4.180412	1.076603	-5.641353	222.5481	0.000749	0.009413
4280445	FOXH1		34.10408	NM	00392	Homo sapiens	2.920841	0.87128	6.153486	5.796585	5.57819	5.178485	2.106751	-1.777252	1.909789	-2.86341	-1.103133	-5.553809	65.07725	4.24E-06	0.000202
3450484	MOCS2		82.51108	NM	17680	Homo sapiens	6.279151	9.366073	7.045711	10.26479	5.576208	8.880757	1.12208	-6.182634	-1.126061	-6.429755	-1.263531	-4.535458	86.49648	3.42E-08	9.03E-06
770221	MT1G		36.07436	NM	00595	Homo sapiens	1.311639	-5.648842	3.133556	1.948107	5.5711	6.057182	2.389039	-0.054483	4.247436	4.404951	1.777884	-2.453428	50.81212	3.15E-06	0.000165
2030195	CAV2		9.807868	NM	00123	Homo sapiens	1.323862	-6.335894	1.826087	-5.580789	5.569516	0.143422	1.379364	-6.057033	4.207022	-1.005209	3.049974	-2.169344	25.76822	0.001573	0.016699
6020279	PLCB3		84.96667	NM	00093	Homo sapiens	3.043343	6.394573	4.532919	9.676933	5.564224	11.06944	1.489454	-2.291104	1.828327	0.55663	1.227515	-4.386966	446.4604	2.90E-08	8.22E-06
2060255	PAGE5		19.1414	NM	13046	Homo sapiens	2.00602	-3.725862	4.908263	1.895502	5.562967	2.577301	2.446767	-2.223773	2.773136	-1.56959	1.133388	-5.556003	58.63524	7.85E-05	0.001721
1740246	MORC3		35.68991	NM	01535	Homo sapiens	7.312035	5.412989	8.096	5.857982	5.54335	3.742252	1.107216	-6.412758	-1.319064	-6.063384	-1.460489	-4.526772	124.3065	3.34E-06	0.00017
5130162	MCOLN2		25.58099	NM	15325	Homo sapiens	1.246808	-6.179878	2.955073	-0.065667	5.536386	4.207965	2.37011	-1.387826	4.440447	3.019499	1.873519	-2.829432	82.87526	1.87E-05	0.000591
5960390	LOC644715		22.60547	XM	93232	PREDICTED:	1.401291	-5.576471	2.561938	-1.269558	5.530559	4.104567	1.828269	-3.516955	3.94676	2.154333	2.158741	-1.924707	43.18273	3.47E-05	0.00093
1070056	LOC649169		34.12928	XM	93974	PREDICTED:	2.424611	-0.02253	4.53547	4.742313	5.529691	6.009567	1.870597	-2.344766	2.280651	-0.65413	1.21921	-5.150591	289.7085	4.22E-06	0.000201
2370180	FLJ12505		43.53515	NM	02474	Homo sapiens	2.546066	1.136296	5.227599	6.581994	5.52948	6.91858	2.053206	-0.860814	2.171774	-0.472478	1.057748	-5.613833	2690.797	1.16E-06	8.34E-05
6220593	OR52A1		27.18465	NM	01237	Homo sapiens	5.611758	3.255384	7.657242	4.751526	5.521732	2.931452	1.3645	-5.748204	-1.016304	-6.782802	-1.386746	-4.910264	150.6883	1.37E-05	0.000465
2370537	MRPL45		36.60841	NM	03235	Homo sapiens	3.999697	3.859408	5.367239	5.69136	5.520663	5.838589	1.341912	-5.349352	1.380271	-5.396449	1.028585	-5.654417	763.5547	2.92E-06	0.000155
2320066	LOC653650		78.33742	XM	93534	PREDICTED:	5.230336	8.661091	6.288869	9.971391	5.518313	9.172891	1.202383	-5.646836	1.055059	-6.705986	-1.139636	-5.25912	128.8224	4.57E-08	1.06E-05
1030274	USP44		21.93478	NM	03214	Homo sapiens	3.488082	0.187317	6.542303	3.704833	5.502584	2.697771	1.875616	-3.941596	1.577538	-5.28396	-1.188951	-5.451051	2455.295	4.03E-05	0.001041
1110685	ENSA		61.89383	NM	20716	Homo sapiens	3.0728	4.509628	5.363459	8.669825	5.481111	8.804247	1.745463	-1.409414	1.783751	-1.336418	1.021936	-5.654068	1437.768	1.70E-07	2.39E-05
7150671	C10orf26		47.38378	NM	01778	Homo sapiens	4.179295	5.442301	5.283998	6.977922	5.479916	7.191423	1.264328	-5.538056	1.311206	-5.501075	1.037078	-5.642087	229.0631	7.32E-07	6.20E-05
540537	C17orf81		17.05939	NM	20341	Homo sapiens	3.374044	-0.069199	4.183499	1.045998	5.479857	2.62787	1.239906	-6.154511	1.624121	-5.12442	1.309874	-5.164108	119.4828	0.000136	0.002595
3140307	LOC653906		13.25746	XM	93657	PREDICTED:	3.41489	-1.615282	5.959995	1.082071	5.475768	0.595125	1.745296	-5.0844	1.603498	-5.713727	-1.088431	-5.631513	102.7097	0.000433	0.006226
130370	HIF1A		52.67041	NM	18105	Homo sapiens	6.118821	6.748085	8.059665	8.27933	5.466521	6.038779	1.317193	-5.433008	-1.119326	-6.588304	-1.474368	-3.902717	562.8666	4.12E-07	4.24E-05

4060195	SNRPN	31.59744	NM	00309	Homo sapiens	3.825718	2.758129	5.710915	5.199724	5.456248	4.88788	1.492769	-4.771132	1.426202	-5.361798	-1.046674	-5.641502	5868.357	6.32E-06	0.000268
2680403	LOC648643	54.26513	XM	93771	PREDICTED:	4.311913	6.284053	5.33524	7.717282	5.454772	7.845624	1.237326	-5.597203	1.265047	-5.673076	1.022404	-5.655384	1191.114	3.50E-07	3.86E-05
4060088	CTH	22.76245	NM	00190	Homo sapiens	2.857384	-0.165576	4.500732	2.789666	5.452405	3.951186	1.575123	-4.65957	1.908181	-3.444156	1.211449	-5.328186	57.33188	3.36E-05	0.000905
4590671	ALDOA	21.93931	NM	18404	Homo sapiens	4.217218	1.519743	6.073914	3.444497	5.410959	2.743046	1.440265	-5.495374	1.283064	-6.283416	-1.122521	-5.56567	4807.042	4.03E-05	0.001041
1470044	CRTAP	48.17173	NM	00637	Homo sapiens	3.62914	4.401077	5.817076	7.56524	5.405281	7.08627	1.60288	-3.279839	1.489411	-4.287984	-1.076184	-5.572641	844.7669	6.70E-07	5.90E-05
610564	CDC14C	24.20144	XM	93712	PREDICTED:	2.716873	-0.65433	5.291352	3.672919	5.40433	3.760122	1.947589	-3.122666	1.989173	-3.184729	1.021351	-5.660923	56.74759	2.47E-05	0.00072
6480091	GSTM1	17.15242	NM	00056	Homo sapiens	2.492199	-1.749169	3.347511	0.084206	5.394403	3.12681	1.343196	-5.774522	2.164515	-2.880403	1.611467	-4.089906	45.57406	0.000132	0.002549
3610041	SBNO1	45.74258	NM	01818	Homo sapiens	3.96408	4.579001	6.174104	7.405338	5.392513	6.551189	1.557512	-3.799601	1.360344	-5.309205	-1.14494	-5.385126	134.9205	8.86E-07	6.96E-05
4560196	LOC648024	40.67113	XM	94335	PREDICTED:	3.563741	3.557595	5.617761	6.567995	5.385845	6.277861	1.576366	-3.790298	1.51129	-4.435617	-1.04306	-5.638524	2403.794	1.67E-06	0.000105
3890754	C1orf43	72.54025	NM	01544	Homo sapiens	4.023177	7.083927	5.934394	9.776444	5.378383	9.160485	1.475052	-3.283549	1.33685	-4.754716	-1.103379	-5.424582	586.2984	7.03E-08	1.37E-05
5130433	TIMP4	74.98212	NM	00325	Homo sapiens	1.64733	-1.335415	3.74814	7.564979	5.358899	10.16675	2.275282	2.874028	3.253081	6.38937	1.429749	-2.789688	365.9011	5.84E-08	1.22E-05
2900192	PCDH10	40.32016	NM	03296	Homo sapiens	5.259373	4.873491	7.792565	7.012942	5.354536	4.838658	1.481653	-4.808314	1.018094	-6.780902	-1.45532	-4.26573	373.3823	1.75E-06	0.000109
670164	CUL4B	26.60208	NM	00358	Homo sapiens	4.492187	3.240471	4.877235	3.585297	5.351467	4.124801	1.085715	-6.439804	1.191283	-6.450811	1.097234	-5.579542	60.89577	1.53E-05	0.000507
1470132	CBR3	37.24538	NM	00123	Homo sapiens	1.014376	-6.686526	3.256774	1.790831	5.341511	5.262779	3.210618	1.971635	5.265809	5.293604	1.640123	-3.29164	87.09707	2.66E-06	0.000146
3710575	LOC648039	44.31904	XM	94371	PREDICTED:	3.789088	4.415547	5.662052	7.060025	5.291178	6.608723	1.494305	-4.137942	1.396425	-5.030151	-1.070093	-5.591031	92.55361	1.05E-06	7.86E-05
3390725	LOC652458	68.62328	XM	94190	PREDICTED:	9.454755	8.962892	9.895772	9.378544	5.286677	5.908168	1.046645	-6.481647	-1.788412	-2.901315	-1.871832	-1.957179	96.65914	9.57E-08	1.72E-05
2640743	ZNF3	19.47228	NM	01771	Homo sapiens	3.733996	0.63343	5.464028	2.707872	5.277162	2.460488	1.463319	-5.440204	1.413275	-5.876204	-1.03541	-5.656196	167.4146	7.22E-05	0.001624
770338	ETV4	32.53111	NM	00198	Homo sapiens	1.507793	-4.565704	3.489323	2.696735	5.275093	5.582645	2.314192	-0.435178	3.498552	2.881995	1.511781	-3.778337	2596.083	5.43E-06	0.000239
2970474	HNRPK	43.6868	NM	03126	Homo sapiens	4.741563	5.413452	6.181274	7.005376	5.271408	5.999626	1.303636	-5.472573	1.111745	-6.604716	-1.172604	-5.308627	445.2113	1.14E-06	8.24E-05
6510470	TJP2	43.98438	NM	00481	Homo sapiens	1.370526	-5.273282	4.875443	5.437779	5.270413	5.916834	3.557351	3.365566	3.84554	3.895979	1.081012	-5.57872	44.60661	1.09E-06	8.06E-05
6450128	SON	53.47058	NM	03219	Homo sapiens	5.789486	6.747457	7.735859	8.399656	5.262573	6.127872	1.336191	-5.259744	-1.100125	-6.634502	-1.469977	-3.833024	109.9225	3.79E-07	4.03E-05
2710593	DNAJB14	46.68498	NM	00103	Homo sapiens	2.353939	0.970396	4.848663	6.81738	5.261436	7.342037	2.059809	-0.315681	2.235163	0.381139	1.085131	-5.541561	64.68596	7.94E-07	6.53E-05
4200703	CNIH3	30.97265	NM	15249	Homo sapiens	1.728705	-3.327573	3.641887	2.991662	5.260301	5.525915	2.106714	-1.322805	3.042914	1.746807	1.444389	-4.12501	74.80188	7.01E-06	0.000286
2760685	RSU1	37.38262	NM	01242	Homo sapiens	2.513085	0.797985	4.667094	5.558837	5.251785	6.316618	1.857118	-2.052005	2.089776	-1.054627	1.125279	-5.453268	101.6842	2.61E-06	0.000144
270754	FOXO1A	93.53374	NM	00201	Homo sapiens	2.052812	2.578656	4.524996	10.15217	5.249762	11.18525	2.204292	3.486724	2.557352	5.148213	1.160169	-4.890333	1384.585	1.69E-08	5.78E-06
4060136	HPS3	93.71137	NM	03238	Homo sapiens	4.708358	8.720473	7.309406	11.58364	5.24929	9.632561	1.552432	-2.280599	1.114888	-6.418016	-1.392456	-3.246442	257.1573	1.67E-08	5.76E-06
160671	ARPC2	21.80707	NM	15286	Homo sapiens	4.805867	3.051019	3.66467	1.063073	5.242435	3.348743	-1.311405	-5.835333	1.090841	-6.710917	1.430534	-4.646753	71.11134	4.15E-05	0.001068
5490403	ATP1B3	67.183	NM	00167	Homo sapiens	4.31451	7.319772	5.62227	9.168161	5.239585	8.711009	1.303107	-4.875272	1.21441	-5.839029	-1.073037	-5.546251	1210.233	1.08E-07	1.84E-05
4830255	CRYBB1	45.16518	NM	00188	Homo sapiens	1.51805	-4.290964	5.149788	6.055181	5.219598	6.115157	3.392372	3.238449	3.438358	3.288217	1.013556	-5.662304	70.23247	9.49E-07	7.35E-05
5340670	LRRC8E	37.22227	NM	02506	Homo sapiens	1.99089	-1.270235	3.875212	4.514688	5.219469	6.584829	1.946472	-1.382077	2.621676	1.385309	1.346886	-4.386609	52.85834	2.67E-06	0.000146
2570711	FLJ30834	55.04439	NM	15239	Homo sapiens	3.137049	4.465591	5.001168	7.962968	5.203508	8.215969	1.594227	-2.665398	1.658727	-2.395434	1.040458	-5.63011	93.87145	3.24E-07	3.68E-05
70148	PDCD10	49.32061	NM	14585	Homo sapiens	3.391877	4.047529	5.942923	7.88617	5.202635	7.029055	1.752104	-2.219898	1.533851	-3.900798	-1.142291	-5.357497	193.6869	5.89E-07	5.35E-05
4280458	SLC3A2	23.06151	NM	00101	Homo sapiens	3.098222	0.313711	5.150585	3.491652	5.19776	3.505955	1.662432	-4.326488	1.677659	-4.506061	1.009159	-5.664271	186.2231	3.15E-05	0.000861
6660711	ARNTL	18.60611	NM	00117	Homo sapiens	2.489596	-2.031434	4.988985	2.300752	5.159962	2.455329	2.003933	-3.429099	2.07261	-3.424419	1.034271	-5.656527	66.57423	8.99E-05	0.001912
4060465	APPBP1	16.60303	NM	00101	Homo sapiens	5.570587	0.993797	7.858542	2.425865	5.152223	0.269806	1.410721	-5.929089	-1.081201	-6.753203	-1.525272	-4.893879	80.87056	0.000154	0.002845
520386	LOC388610	75.38929	NM	00101	Homo sapiens	2.372908	3.556908	4.484037	9.220341	5.133751	10.16469	1.88968	0.854864	2.163485	2.448242	1.144895	-5.105311	1446.899	5.67E-08	1.21E-05
7320564	SMPDL3B	37.48814	NM	00100	Homo sapiens	1.927176	-1.285024	3.310617	3.724783	5.119572	6.958247	1.717859	-2.416267	2.656515	1.927838	1.54641	-3.070299	43.36293	2.57E-06	0.000143
1940594	TOP1MT	13.55763	NM	05296	Homo sapiens	2.179752	-3.392818	3.652346	-0.324092	5.113899	1.665561	1.675579	-4.860652	2.346092	-2.990413	1.400168	-4.985036	77.15999	0.000392	0.005799
3890360	IPPK	35.77121	NM	02275	Homo sapiens	2.719243	1.660357	4.271188	5.110121	5.106527	6.311227	1.570727	-3.668298	1.877922	-2.039246	1.195575	-5.177454	86.80994	3.30E-06	0.000169
1940528	VGF	12.4514	NM	00337	Homo sapiens	1.118769	-6.623759	3.174113	-2.352427	5.104043	0.232448	2.837147	-2.51762	4.562196	-0.090131	1.608022	-4.705059	1791.42	0.000571	0.007685
5340088	SLTM	24.92967	NM	02475	Homo sapiens	3.735134	1.620593	5.827732	4.217898	5.098532	3.376122	1.560247	-4.7758	1.36502	-5.863398	-1.143022	-5.503861	234.3956	2.13E-05	0.000649
3610671	ALG1	70.58699	NM	01910	Homo sapiens	2.897867	4.870483	5.305055	9.57709	5.097522	9.315862	1.830676	-0.14776	1.75906	-0.848346	-1.040713	-5.620748	211.7737	8.18E-08	1.53E-05
6250561	ECAT11	94.4981	NM	01907	Homo sapiens	2.634082	5.606289	4.900213	10.86498	5.096227	11.1502	1.860311	1.449063	1.934726	1.878603	1.040001	-5.605235	18802.02	1.59E-08	5.53E-06
6840209	ADK	23.6269	NM	00112	Homo sapiens	3.154404	1.021516	4.289869	2.9903	5.095897	4.075502	1.359962	-5.500205	1.615486	-4.535762	1.187891	-5.362353	92.88205	2.79E-05	0.000792
4250543	LOC650919	10.30362	XM	94441	PREDICTED:	1.942759	-4.456483	2.478637	-3.361622	5.088659	0.887705	1.275834	-6.165151	2.619295	-2.758566	2.053006	-3.43398	33.2488	0.001283	0.014189
4590554	CORO2A	80.07598	NM	05282	Homo sapiens	2.514423	4.918916	3.913955	9.001966	5.088654	10.92152	1.556602	-1.352754	2.023786	2.302026	1.300131	-3.586716	2637.972	4.04E-08	9.88E-06
2480152	LOC650020	61.20098	XM	93911	PREDICTED:	4.624398	7.278932	5.633807	8.63454	5.083355	7.958008	1.218279	-5.635415	1.099247	-6.569669	-1.108285	-5.439038	157.886	1.81E-07	2.48E-05
6770553	TUBG1	25.08368	NM	00107	Homo sapiens	3.555959	1.599346	5.306884	4.02418	5.068338	3.699679	1.492392	-4.97576	1.425309	-5.535592	-1.047066	-5.644258	350.8691	2.06E-05	0.000634
1580370	SNCB	10.79522	XM	93649	PREDICTED:	3.979556	-1.553448	6.27118	0.369528	5.063531	-0.719255	1.575849	-5.705446	1.272386	-6.544833	-1.238499	-5.495403	58.70565	0.001056	0.012197
1190079	LOC642817	35.1419	XM	92670	PREDICTED:	5.999985	5.279062	6.485575	5.636824	5.060962	4.116596	1.080932	-6.443019	-1.185542	-6.447503	-1.281491	-5.046693	11793.86	3.62E-06	0.00018
70070	PIGW	32.72753	NM	17851	Homo sapiens	3.987985	3.442575	5.515598	5.426594	5.057333	4.845959	1.383054	-5.223659	1.268143	-6.051634	-1.090614	-5.573162	771.359	5.26E-06	0.000234
290400	MCCC2	16.90553	NM	02213	Homo sapiens	2.804056	-1.007373	4.0413	1.216557	5.042239	2.560044	1.441233	-5.445712	1.798195	-4.311397	1.247678	-5.293151	35.27843	0.000142	0.002677
3780056	SEH1L	30.17918	NM	03121	Homo sapiens	4.196942	3.69129	4.786822	4.416032	5.040175	4.715758	1.14055	-6.287218	1.200916	-6.347906	1.052927	-5.633032	357.5174	8.02E-06	0.000315
2350112	CD2AP	28.79241	NM	01212	Homo sapiens	3.242767	1.712755	5.090389	4.670716	5.034541	4.563956	1.569767	-4.315702	1.552545	-4.653083	-1.011093	-5.663606	538.8211	1.02E-05	0.000378
1170471	APOBEC3B	20.9075	NM	00490	Homo sapiens	2.1463	-2.936984	5.345437	2.984821	5.032237	2.579593	2.490536	-1.71485	2.34461	-2.365213	-1.062239	-5.636195	49.10445	5.11E-05	0.001247
1010753	TERF1	62.20039	NM	00321	Homo sapiens	2.200343	1.578141	4.85898	8.466049	5.031974	8.69744	2.208283	1.684765	2.286905	1.962296	1.035603	-5.634105	1048.117	1.65E-07	2.35E-05
7150605	ORC5L	32.87207	NM	00255	Homo sapiens	3.512135	2.56709	5.710678	5.677292	5.028827	4.84727	1.625985	-3.908022	1.431843	-5.211576	-1.135589	-5.468257	85.59895	5.14E-06	0.00023
4220246	LOC643790	13.01231	XM	92707	PREDICTED:	4.293867	-0.470767	6.142934	1.100647	5.019073	0.010652	1.43063	-5.899555	1.168893	-6.662287	-1.223918	-5.482692	257.9884	0.00047	0.006617
6180605	WRNIP1	26.13786	NM	02013	Homo sapiens	3.500351	1.909873	4.897945	4.001958	5.018938	4.119095	1.399273	-5.291741	1.433839	-5.383515	1.024703	-5.658599	158.2905	1.68E-05	0.000542
4560414	LOC644615	44.907	XM	92773	PREDICTED:	2.412287	1.421954	4.490427	6.535509	5.018761	7.284199	1.861481	-1.248814	2.080499	-0.193337	1.117658	-5.42784	500.3752	9.79E-07	7.49E-05
50053	MAPKAP1	40.90312	NM	00100	Homo sapiens	2.911737	2.959351	4.244585	5.855375	5.01572	7.001815	1.45775	-4.116881	1.722587	-2.501264	1.181675	-5.170575	195.1826	1.62E-06	0.000104
4760328	STK17A	30.19167	NM	00476	Homo sapiens	2.927904	1.066798	5.242647	5.040588	5.013385	4.720114	1.79058	-3.102979	1.712278	-3.735439	-1.04573	-5.64078	93.50461	8.00E-06	0.000315
1340044	ETFB	34.96714	NM	00198	Homo sapiens	2.572904	1.390876	3.783411	4.479677	5.010679	6.471148	1.470483	-4.216889	1.94748	-1.472332	1.324381	-4.481843	1376.507	3.72E-06	0.000184
6220692	IFITM1	29.1629	NM	00364	Homo sapiens	2.31963	-0.69033	4.382866	4.155472	5.005549	5.012303	1.889468	-2.452592	2.157908	-1.423921	1.142072	-5.442666	15184.07	9.57E-06	0.000361
3460687	PEMT	19.03014	NM	14817	Homo sapiens	3.501821	0.175349	5.685889	2.884253	4.995249	2.086196	1.623695	-4.872391	1.426472	-5.839455	-1.13826	-5.545208	202.206	8.07E-05	0.001762
3370017	MFAP2	24.03743	NM	00240	Homo sapiens	4.13308	1.87828	6.293363	4.190756	4.98559	2.786427	1.522681	-5.060656	1.206265	-6.463972	-1.262311	-5.230168	71.29077	2.56E-05	0.00074
4570615	MGC34647	41.08247	NM	15245	Homo sapiens	1.228242	-6.018567	3.920563	4.137059	4.983726	5.794905	3.192012	2.755583	4.057609	4.508403	1.271176	-4.867576	55.43553	1.58E-06	0.000103
1300129	COL4A3BP	15.10203	NM	00571	Homo sapiens	5.261983	1.362679	5.050277	0.883935	4.978748	0.753396	-1.04192	-6.504275	-1.056889	-6.766401	-1.014367	-5.663927	102.3153	0.00024	0.003986
1690070	FBXO7	17.19939	NM	01217	Homo sapiens	2.436022	-2.163563	4.521222	1.735065	4.966751	2.262582	1.855985	-3.939819	2.038878	-3.522448	1.098542	-5.598744	72.59986	0.000131	0.002527
4760369	LOC643554	84.93856	XM	92685	PREDICTED:	2.657283	5.135865	5.093279	10.5172	4.95754	10.35019	1.916725	1.368236	1.865643	0.870213	-1.02738	-5.63961	310.8954	2.90E-08	8.22E-06
1740754	TMED10	58.73723	NM	00682	Homo sapiens	4.48469	6.945628	5.682768	8.546541	4.956173	7.643775	1.267148	-5.315771	1.105132	-6.551252	-1.146604	-5.281401	984.7012	2.26E-07	2.88E-05
2070270	IQSEC2	31.07448	NM	01507	Homo sapiens	1.272696	-5.849529	2.758298	0.878069	4.9556	5.330666	2.167288	-0.930184	3.893782	3.815818	1.796615	-2.358523	153.3356	6.89E-06	0.000283
2140731	LRRC2	13.26449	NM	02451	Homo sapiens	2.989156	-1.162163	3.446788	-0.529315	4.94095	1.659157	1.153097	-6.365522	1.652958	-5.131544	1.433494	-4.863977	54.89108	0.000432	0.00622
4070735	GFM2	28.28634	NM	17068	Homo sapiens	5.067176	3.388853	6.969815	5.039955	4.93489	3.000722	1.375483	-5.590002	-1.026806	-6.778103	-1.412355	-4.707623	66.80221	1.12E-05	0.000402
6200603	LACTB	67.11153	NM	17184	Homo sapiens	3.352411	5.784716	5.613403	9.545802	4.930238	8.693065	1.674438	-1.568008	1.470655	-3.501706	-1.138566	-5.245488	258.2725	1.08E-07	1.84E-05
5720605	SNURF	25.6757	NM	00567	Homo sapiens	3.073523	1.05826	4.73869	3.925208	4.926659	4.138831	1.541778	-4.555904	1.602935	-4.495006	1.039667	-5.648252	8794.862	1.83E-05	0.000582
840328	ADSS	63.83056	NM	00112	Homo sapiens	3.737298	6.645786	5.041613	8.85145	4.908526	8.664731	1.348999	-4.360484	1.313389	-4.929278	-1.027113	-5.646467	661.4432	1.43E-07	2.15E-05

3520678	HSPD1	64.96935	NM	00215	Homo sapiens	3.807503	6.889291	4.978738	8.879033	4.905872	8.774103	1.307612	-4.697106	1.288475	-5.120729	-1.014853	-5.65927	16082.81	1.30E-07	2.05E-05
3060324	RTEL1	11.45757	NM	01643	Homo sapiens	2.684265	-2.878208	4.936643	0.247446	4.904859	0.159496	1.839104	-4.788013	1.827263	-5.072518	-1.00648	-5.664833	113.632	0.000819	0.010092
6980692	TRNT1	22.58682	NM	01600	Homo sapiens	3.850604	1.457533	5.792352	3.762971	4.902006	2.723138	1.504271	-5.124566	1.273049	-6.257419	-1.181629	-5.435249	124.7297	3.49E-05	0.000932
5310431	DDX47	39.9486	NM	01635	Homo sapiens	3.933691	4.609376	5.100541	6.309216	4.901397	6.018016	1.29663	-5.421755	1.246005	-5.974521	-1.04063	-5.639741	909.6222	1.83E-06	0.000112
4670097	LOC644544	52.74437	XM	92766	PREDICTED:	2.912656	4.776035	3.377451	6.021439	4.898503	8.887668	1.159578	-5.887821	1.6818	-1.559275	1.450355	-2.885554	126.5758	4.09E-07	4.22E-05
2490730	KIAA0888	33.93008	XM	03257	PREDICTED:	2.693261	0.810701	5.435574	5.806081	4.895956	5.10138	2.018213	-1.696663	1.817855	-2.907941	-1.110217	-5.519886	73.45854	4.35E-06	0.000206
3450167	SLC25A26	40.04204	NM	00100	Homo sapiens	3.33009	4.05644	4.148211	5.645303	4.889748	6.788251	1.245675	-5.596571	1.468353	-4.299013	1.178761	-5.187924	340.1044	1.81E-06	0.000112
4480494	GABBR2	22.30379	NM	00545	Homo sapiens	2.53057	-1.19747	5.090262	3.442039	4.874964	3.130198	2.011508	-2.867561	1.926429	-3.435175	-1.044164	-5.64783	183.4514	3.71E-05	0.000975
1450102	SBDSP	46.93312	NR	00158	Homo sapiens	4.279146	5.747096	5.399083	7.269668	4.87405	6.573669	1.26172	-5.528236	1.139024	-6.455297	-1.10772	-5.481543	138.431	7.71E-07	6.42E-05
7210471	DDX19B	21.517	NM	00101	Homo sapiens	2.806135	-0.988336	6.108861	3.737472	4.872486	2.36985	2.176967	-2.69955	1.736369	-4.545006	-1.253746	-5.275893	65.32051	4.43E-05	0.001121
2510246	JAK1	18.31518	NM	00222	Homo sapiens	2.700802	-1.114162	4.50774	2.130182	4.871226	2.569177	1.669037	-4.49098	1.803622	-4.196651	1.080636	-5.615691	59.99718	9.70E-05	0.002023
6060368	C1orf94	45.072	NM	03288	Homo sapiens	1.301118	-5.37584	3.373814	4.127194	4.871141	6.881966	2.593012	2.021015	3.743812	5.072263	1.443808	-3.609854	773.7874	9.60E-07	7.39E-05
6480725	PRKCH	15.73718	NM	00625	Homo sapiens	2.095174	-3.429743	4.301461	1.094651	4.869724	1.801459	2.053032	-3.419302	2.324257	-2.795211	1.13211	-5.557917	147.3222	0.000198	0.003425
1440408	PPM1K	51.50522	NM	15254	Homo sapiens	3.864834	5.899241	4.871515	7.538875	4.868855	7.518629	1.260472	-5.36467	1.259784	-5.628659	-1.000546	-5.665028	275.3598	4.65E-07	4.57E-05
6350768	C14orf126	113.1245	NM	08066	Homo sapiens	3.249173	8.494186	5.065815	12.08128	4.86782	11.85325	1.559109	-0.505943	1.498172	-1.384499	-1.040674	-5.590636	71.58354	5.74E-09	3.63E-06
1010719	LOC652634	81.7354	XM	94218	PREDICTED:	3.088307	6.620915	4.805524	10.18957	4.861885	10.28161	1.556038	-1.564564	1.574288	-1.585714	1.011728	-5.660231	250.5968	3.60E-08	9.28E-06
4890242	ANP32C	29.75092	NM	01240	Homo sapiens	3.320011	1.685672	5.913144	5.351788	4.861836	4.101888	1.781061	-3.354197	1.464404	-5.183343	-1.216237	-5.252612	576.381	8.63E-06	0.000334
5260348	RPL13	32.47824	NM	03325	Homo sapiens	1.81516	-2.259256	3.243662	3.060962	4.854706	6.073219	1.786984	-2.288637	2.674534	1.544261	1.496674	-3.540757	511.3344	5.47E-06	0.00024
4670692	OAT	89.97721	NM	00027	Homo sapiens	3.335371	7.409245	5.343103	11.03032	4.847316	10.40118	1.601951	-1.033767	1.453306	-2.755647	-1.102281	-5.332299	2354.774	2.10E-08	6.72E-06
6100240	PLEKHB2	37.11524	NM	00103	Homo sapiens	3.893536	4.257057	5.027295	5.911609	4.839624	5.628998	1.29119	-5.508063	1.242989	-6.032574	-1.038778	-5.643223	516.9133	2.71E-06	0.000147
670487	NUP43	18.52395	NM	19888	Homo sapiens	4.127752	1.491893	4.671129	2.022637	4.838789	2.191807	1.13164	-6.385348	1.172258	-6.571263	1.035893	-5.655478	407.5096	9.19E-05	0.00194
4540367	TBC1D2	33.10679	NM	01842	Homo sapiens	1.88811	-2.059334	3.914669	4.226072	4.832366	5.692667	2.073326	-0.995227	2.559366	0.850714	1.234425	-5.028049	300.3679	4.95E-06	0.000225
2940435	SON	50.53486	NM	13892	Homo sapiens	5.970911	7.244451	6.185702	7.497297	4.82227	5.895174	1.035973	-6.492401	-1.238195	-6.019233	-1.282737	-4.762837	76.06028	5.16E-07	4.90E-05
1070626	PPP1R3B	27.37212	NM	02460	Homo sapiens	2.806571	0.528147	5.017417	4.522661	4.814584	4.217698	1.78774	-3.234967	1.715469	-3.828364	-1.042129	-5.645297	62.12226	1.32E-05	0.000455
6270437	EDNRB	13.47547	NM	00399	Homo sapiens	2.456219	-2.246391	3.26103	-0.593906	4.809951	1.855882	1.327663	-5.902444	1.958275	-3.911741	1.474979	-4.678073	34.03642	0.000402	0.005911
6900097	MAGI2	32.78252	NM	01230	Homo sapiens	2.653758	0.506186	5.572132	5.737779	4.799486	4.748854	2.099714	-1.469857	1.808562	-3.075716	-1.160985	-5.385165	142.7975	5.21E-06	0.000233
620360	LOC654135	13.27777	XM	94593	PREDICTED:	1.473108	-5.885849	4.453229	-0.084535	4.780304	0.257296	3.023015	-1.885742	3.245046	-1.674615	1.073447	-5.639372	59.83347	0.00043	0.00621
620494	RRM2	83.3199	NM	00103	Homo sapiens	3.898367	8.361195	4.942601	10.23899	4.775403	10.01296	1.267865	-4.671799	1.224975	-5.371373	-1.035012	-5.624421	689.4882	3.23E-08	8.76E-06
6200747	LOC285216	16.71917	XM	49665	PREDICTED:	2.746972	-1.911292	5.927666	2.472208	4.771676	1.197196	2.157891	-3.34585	1.737068	-4.928439	-1.242261	-5.375264	72.07377	0.000149	0.002779
650280	JMJD2C	42.40995	NM	01506	Homo sapiens	2.887085	2.21781	5.914581	7.29832	4.770116	5.889953	2.048634	-0.921741	1.652226	-3.366249	-1.239924	-4.968624	68.88363	1.33E-06	9.12E-05
4060288	SMCX	12.5775	NM	00418	Homo sapiens	4.025207	-0.314712	4.893918	0.491044	4.767229	0.29341	1.215818	-6.298802	1.184344	-6.621166	-1.026575	-5.661471	166.5557	0.000546	0.00742
1980224	DKFZp434N035	14.10709	XM	94355	PREDICTED:	2.6145	-1.819035	3.704725	0.247224	4.766193	1.786429	1.416992	-5.61367	1.822985	-4.399669	1.286517	-5.227414	100.4972	0.000327	0.005082
4230343	DDX6	42.04991	NM	00439	Homo sapiens	2.281682	1.349026	3.504007	5.226912	4.74363	7.517449	1.535713	-3.152253	2.079006	0.26568	1.353773	-3.972727	224.2107	1.39E-06	9.42E-05
4610133	MAT2B	19.34417	NM	18279	Homo sapiens	2.326665	-2.127495	4.706781	2.545745	4.742466	2.547592	2.022974	-3.046522	2.038311	-3.217217	1.007581	-5.66455	216.2179	7.46E-05	0.001661
2970110	LOC653717	56.76523	XM	49603	PREDICTED:	6.586846	7.671666	7.605162	8.543342	4.728203	5.647157	1.154598	-6.166734	-1.393097	-5.111057	-1.608468	-3.0131	198.8496	2.73E-07	3.27E-05
2640220	DHODH	43.28056	NM	00136	Homo sapiens	3.072048	3.839126	4.3205	6.444273	4.714924	7.045555	1.406391	-4.333563	1.534782	-3.591367	1.091291	-5.512237	98.69021	1.19E-06	8.51E-05
5390719	LOC644037	24.43695	XM	93360	PREDICTED:	4.96204	3.310074	5.410564	3.652133	4.714188	2.759998	1.090391	-6.44067	-1.052576	-6.758824	-1.147719	-5.498159	16699.95	2.35E-05	0.000695
7100474	SNX7	22.09997	NM	15223	Homo sapiens	3.691963	1.561662	5.051449	3.383496	4.707983	2.897795	1.368228	-5.58514	1.275198	-6.204001	-1.072954	-5.61959	418.5906	3.89E-05	0.00101
6040196	PPP1R16B	14.1517	NM	01556	Homo sapiens	1.417328	-5.912789	3.715995	-0.435163	4.706047	0.936559	2.621832	-2.206266	3.320367	-0.906075	1.26643	-5.336088	252.9752	0.000323	0.00503
2470184	KCTD8	60.68532	NM	19835	Homo sapiens	3.106723	5.049879	5.183121	8.935712	4.697015	8.257185	1.668356	-1.675334	1.511887	-3.184024	-1.103493	-5.423434	177.2083	1.89E-07	2.55E-05
1340301	LOC647534	27.2798	XM	94288	PREDICTED:	2.708811	-1.268841	8.55191	5.461473	4.694763	2.104625	3.157071	-0.085252	1.733145	-4.571677	-1.821585	-3.43518	39.59043	1.35E-05	0.000461
1940731	MET	100.5795	NM	00024	Homo sapiens	1.574168	-0.788895	4.07762	10.07401	4.692854	11.12214	2.590333	5.849511	2.981164	7.341902	1.150881	-4.877932	355.4096	1.12E-08	4.90E-06
4560100	COL21A1	32.71298	NM	03082	Homo sapiens	2.013388	-1.621048	4.523399	4.950072	4.684185	5.156205	2.246661	-0.451164	2.326519	-0.295999	1.035546	-5.647495	71.26196	5.27E-06	0.000234
1090576	TNNC2	25.45447	NM	00327	Homo sapiens	1.805219	-3.040085	3.482943	2.430456	4.683447	4.529595	1.929374	-2.283206	2.594393	0.205603	1.344681	-4.653894	72.91948	1.92E-05	0.0006
6900195	RECQL	31.98744	NM	03294	Homo sapiens	4.172196	3.647824	5.720378	5.536026	4.676651	4.219946	1.371071	-5.309253	1.120909	-6.611614	-1.223178	-5.197969	190.5902	5.93E-06	0.000255
4490551	PPL	29.17795	NM	00270	Homo sapiens	2.218492	-0.574728	3.777627	3.784441	4.669422	5.280388	1.702791	-3.060203	2.104773	-1.158544	1.236073	-5.040545	342.2036	9.54E-06	0.00036
4180647	ARHGEF5	61.19585	NM	00100	Homo sapiens	1.688015	-0.827639	3.075601	6.059862	4.665828	9.463	1.822023	0.338532	2.764092	5.102447	1.517046	-1.973275	222.5808	1.81E-07	2.48E-05
4920315	DUSP10	18.83086	NM	14472	Homo sapiens	3.115425	-0.028025	4.786835	2.576793	4.665119	2.369877	1.536495	-5.005706	1.497426	-5.419938	-1.026091	-5.659489	220.4346	8.49E-05	0.00183
5860239	ARHGEF1	22.67924	NM	19900	Homo sapiens	2.977455	0.432123	4.762067	3.502132	4.65625	3.314445	1.599375	-4.431076	1.563836	-4.848101	-1.022726	-5.65991	241.7013	3.42E-05	0.000916
620014	TPST2	38.59289	NM	00359	Homo sapiens	1.537921	-3.754202	3.496076	4.215555	4.654047	6.34871	2.273249	0.578946	3.026194	3.139358	1.33122	-4.364161	51.166	2.20E-06	0.000129
3180270	SLC1A1	65.09769	NM	00417	Homo sapiens	1.772796	-0.821015	4.689339	8.536259	4.650584	8.469965	2.645165	3.811724	2.623305	3.698119	-1.008333	-5.663184	167.4554	1.28E-07	2.03E-05
5690239	BCL11A	22.52314	NM	13855	Homo sapiens	1.868459	-2.962425	3.370039	1.768926	4.649109	4.039709	1.803647	-3.130884	2.488206	-0.500219	1.379542	-4.577868	88.46292	3.54E-05	0.000943
2570717	LOC653276	24.86732	XM	93149	PREDICTED:	1.089484	-6.592057	1.824903	-3.469733	4.644419	4.131479	1.675016	-3.730306	4.262952	3.727729	2.545022	-0.223411	33.26714	2.16E-05	0.000655
4900593	PRKAR2A	30.11663	NM	00415	Homo sapiens	3.197946	2.718652	3.90706	4.096268	4.643362	5.301208	1.22174	-5.882229	1.451982	-4.805357	1.188454	-5.237426	297.0582	8.10E-06	0.000318
3990180	GPM6B	12.55612	NM	00100	Homo sapiens	2.368058	-3.430132	5.269521	0.887466	4.636394	0.132042	2.22525	-3.641044	1.957889	-4.602078	-1.136556	-5.580942	49.29258	0.000551	0.007456
3990196	UMPS	23.29139	NM	00037	Homo sapiens	2.35955	-0.878428	3.89963	2.875736	4.636125	4.037436	1.652701	-3.880194	1.964834	-2.582473	1.188863	-5.324483	76.67386	2.99E-05	0.000829
5260433	ALDH4A1	26.9335	NM	00374	Homo sapiens	2.989997	1.403962	4.435086	4.138181	4.634939	4.400535	1.483308	-4.65155	1.550149	-4.530191	1.045062	-5.640547	622.749	1.44E-05	0.000482
6100544	MYOZ3	55.62284	NM	13337	Homo sapiens	1.79749	-0.437163	3.459939	6.458681	4.625956	8.720263	1.924873	0.544139	2.573565	3.759648	1.337005	-3.717354	644.6919	3.06E-07	3.50E-05
6180400	WDR74	18.4545	NM	01809	Homo sapiens	2.222551	-2.277807	4.050417	1.876083	4.621011	2.697137	1.822418	-3.675043	2.079148	-2.883773	1.140873	-5.510133	86.86585	9.35E-05	0.001969
3170167	CLEC2D	19.25569	NM	01326	Homo sapiens	4.437142	2.114772	4.626467	2.148365	4.620711	2.093459	1.042668	-6.498987	1.041371	-6.77034	-1.001246	-5.665022	5823.22	7.62E-05	0.001687
450315	CDK2	23.68607	NM	00179	Homo sapiens	4.153054	3.077936	3.990372	2.605336	4.611997	3.550198	-1.040769	-6.495406	1.110508	-6.653958	1.155781	-5.444148	69.07338	2.75E-05	0.000786
1230309	PTCHD1	39.50997	NM	17349	Homo sapiens	2.255054	0.775995	3.936713	5.616059	4.608243	6.751873	1.745729	-1.942924	2.043518	-0.349784	1.170581	-5.195647	49.95496	1.95E-06	0.000117
3800279	ADAM22	13.35938	NM	00419	Homo sapiens	2.059021	-3.484319	3.371838	-0.372349	4.60146	1.580389	1.637593	-4.824514	2.23478	-2.995428	1.364674	-5.011912	65.16381	0.000418	0.006093
3840176	RFC2	46.98223	NM	18147	Homo sapiens	3.34922	4.742165	4.694542	7.233227	4.600894	7.071955	1.401682	-4.302363	1.373721	-4.769929	-1.020354	-5.656481	833.3812	7.67E-07	6.42E-05
380019	SEPHS1	86.47648	NM	01224	Homo sapiens	2.234398	3.784595	4.596144	10.43169	4.595695	10.44537	2.056995	2.788461	2.056794	2.712313	-1.000098	-5.665034	5339.242	2.62E-08	7.89E-06
4730091	ING5	39.71959	NM	03232	Homo sapiens	2.090412	0.06959	3.746283	5.363953	4.583137	6.852863	1.792126	-1.556589	2.192456	0.527923	1.223383	-4.899332	40.76225	1.89E-06	0.000114
4830414	FLJ43692	42.00964	NM	00100	Homo sapiens	1.975738	-0.537813	3.966762	5.841298	4.581125	6.880406	2.007737	-0.251003	2.31869	1.148408	1.154878	-5.257582	90.41977	1.40E-06	9.45E-05
7560575	INADL	25.31603	NM	17687	Homo sapiens	2.085397	-1.296811	3.114773	2.077538	4.579473	4.97235	1.493611	-4.379044	2.195972	-0.868238	1.470243	-3.895954	86.13683	1.97E-05	0.000613
5490608	DEPDC1B	24.97617	NM	01836	Homo sapiens	4.897709	3.471102	5.277588	3.755063	4.569764	2.806925	1.077563	-6.456573	-1.071764	-6.73472	-1.154893	-5.47383	479.5205	2.11E-05	0.000645
3990243	RQCD1	33.83185	NM	00544	Homo sapiens	2.318708	0.504307	3.870054	4.794648	4.559166	5.968474	1.669056	-2.834071	1.966253	-1.272425	1.178063	-5.218562	183.1346	4.42E-06	0.000208
1690326	LOC441032	13.356	XM	93349	PREDICTED:	4.188693	-0.156329	5.684435	1.22337	4.556899	-0.028356	1.35709	-6.008728	1.087905	-6.744656	-1.247435	-5.421773	58.83584	0.000419	0.006096
5090168	ILF3	69.56299	NM	00451	Homo sapiens	4.769974	8.414235	5.311894	9.278902	4.55676	8.225344	1.113611	-6.184554	-1.046791	-6.723564	-1.165717	-5.079927	2891.995	8.88E-08	1.63E-05
5290687	C9orf85	30.62162	NM	18250	Homo sapiens	2.750119	1.110529	4.800913	5.150725	4.550338	4.752736	1.745711	-2.994572	1.654596	-3.738884	-1.055067	-5.625389	342.5787	7.43E-06	0.000299
3400731	CENTB1	24.0981	NM	01471	Homo sapiens	1.450906	-5.00381	2.87152	0.775531	4.543729	4.221697	1.979122	-2.110543	3.13165	1.686013	1.582343	-3.553627	205.6932	2.52E-05	0.000733
5560040	MGC34829	22.0305	XM	20899	PREDICTED:	3.804242	2.136584	4.405019	2.913743	4.543515	3.074694	1.157923	-6.280682	1.194329	-6.441905	1.031441	-5.655467	64.93336	3.95E-05	0.001023
4040259	ITM2A	29.26933	NM	00486	Homo sapiens	1.654697	-3.52835	3.508636	3.133795	4.536405	5.000072	2.12041	-0.953537	2.741533	1.254593	1.292926	-4.784975	720.7138	9.39E-06	0.000355
6400025	LOC401305	12.87779	XM	37657	PREDICTED:	2.187714	-3.085273	3.353907	-0.442343	4.533818	1.450673	1.533064	-5.21419	2.072399	-3.544837	1.351802	-5.053595	83.38723	0.000492	0.006865

2100240	TMEPAI		13.39898	NM	02018	Homo sapiens	1.845837	-4.411085	3.854612	0.107552	4.533712	1.056083	2.088274	-3.470775	2.456183	-2.620364	1.176178	-5.49498	108.5155	0.000413	0.006027
4560753	LOC653468		15.56521	XM	93420	PREDICTED:	2.795337	-0.852328	3.701889	0.84648	4.530196	2.128124	1.324309	-5.83063	1.620626	-4.932768	1.223752	-5.338723	91.08712	0.000209	0.003572
4120017	ANTXR1		28.59375	NM	05303	Homo sapiens	2.283052	0.157867	3.118397	2.799002	4.515651	5.652233	1.36589	-4.945153	1.977901	-1.377973	1.448068	-3.79493	141.6703	1.06E-05	0.000386
4290364	LOC653542		37.27812	XM	92799	PREDICTED:	5.593732	6.117674	4.861825	5.181803	4.509516	4.637917	-1.150541	-6.222471	-1.240428	-6.11043	-1.078126	-5.58803	141.0573	2.65E-06	0.000146
6760075	EOMES		12.76325	NM	00544	Homo sapiens	3.821017	-0.150025	4.368622	0.358049	4.50789	0.489353	1.143314	-6.399548	1.179762	-6.609198	1.031879	-5.659296	5977.415	0.000512	0.007055
1300524	LOC647436		18.09587	XM	93711	PREDICTED:	3.565519	0.580102	5.067094	2.551103	4.50332	1.780728	1.421138	-5.535753	1.26302	-6.329304	-1.125191	-5.558379	1378.625	0.000103	0.002109
2120561	DUSP22		22.37514	NM	02018	Homo sapiens	3.659143	1.750832	4.887757	3.45829	4.501926	2.884911	1.335765	-5.670372	1.230322	-6.333564	-1.085704	-5.599623	69.88047	3.65E-05	0.000964
6860382	ITSN1		64.61045	NM	00100	Homo sapiens	3.084695	5.374417	5.242481	9.433297	4.50014	8.377165	1.699513	-1.165719	1.458861	-3.455919	-1.164959	-5.059826	116.8713	1.34E-07	2.07E-05
7380039	TCEA1		33.32463	XM	94311	PREDICTED:	4.7368	4.175143	6.518701	5.975795	4.494654	3.639253	1.376182	-5.350073	-1.053874	-6.750176	-1.450323	-4.302516	207.9579	4.78E-06	0.00022
1240088	OSBPL10		69.33727	NM	01778	Homo sapiens	1.504074	-2.537155	3.760911	7.79112	4.492041	9.152664	2.500482	4.051938	2.986582	5.810454	1.194402	-4.748757	1013.968	9.04E-08	1.65E-05
5220440	SMPDL3B		31.84709	NM	01447	Homo sapiens	1.827161	-2.279841	3.78409	4.157207	4.487544	5.377602	2.071021	-0.873	2.456019	0.623683	1.185898	-5.223142	575.758	6.06E-06	0.00026
1580703		0	18.17401	XM	49856	PREDICTED:	1.903188	-3.157114	3.104169	0.545355	4.485506	3.146352	1.631037	-4.25332	2.356839	-1.443142	1.444994	-4.433503	58.59887	0.000101	0.002074
3830040	NSF		20.5964	NM	00617	Homo sapiens	4.233139	2.452145	4.2773	2.303566	4.473052	2.551588	1.010432	-6.513356	1.056675	-6.753897	1.045765	-5.646691	75.97348	5.50E-05	0.001313
50719	LOC653930		22.26095	XM	94361	PREDICTED:	2.977024	0.870271	4.067428	2.959385	4.47101	3.569161	1.366273	-5.404611	1.501839	-4.986209	1.099223	-5.5652	99.10879	3.75E-05	0.000981
6100634	FGD5		16.48786	NM	15253	Homo sapiens	2.135659	-2.851579	4.026442	1.420469	4.467896	2.049038	1.88534	-3.627358	2.092046	-3.088755	1.109639	-5.576161	243.0495	0.00016	0.002912
1770703	PHKB		46.18059	NM	00103	Homo sapiens	4.289846	6.306888	4.553739	6.7101	4.461559	6.540569	1.061516	-6.436279	1.040028	-6.75081	-1.020661	-5.656717	56.61791	8.42E-07	6.69E-05
4760079	LOC642525		33.80889	XM	92601	PREDICTED:	3.196579	2.472908	5.373364	6.018377	4.461381	4.750371	1.680973	-3.290502	1.395674	-5.228702	-1.204417	-5.195933	216.8021	4.44E-06	0.000209
5720706	IRF6		15.1295	NM	00614	Homo sapiens	1.125039	-6.575563	2.396044	-2.677953	4.455207	1.506121	2.129743	-3.033875	3.960047	1.013807	1.859401	-3.417821	30.20609	0.000238	0.003965
1110598	CTAGE6		34.90044	XM	49846	PREDICTED:	1.192519	-6.180509	3.3001	2.885668	4.44531	5.120384	2.767335	1.67633	3.727663	3.990614	1.347023	-4.462711	134.2268	3.75E-06	0.000185
5130014	LRRC40		36.77401	NM	01776	Homo sapiens	4.334022	4.472188	6.006111	6.450479	4.443442	4.476919	1.385806	-5.092601	1.025247	-6.775606	-1.35168	-4.564692	384.7083	2.85E-06	0.000152
7550253	ABHD11		28.00683	NM	14891	Homo sapiens	2.723861	1.311086	3.868542	3.95621	4.443058	4.926637	1.420242	-4.771165	1.631162	-3.720828	1.14851	-5.389327	65.44024	1.18E-05	0.000418
7210164	LOC648057		17.58101	XM	93710	PREDICTED:	4.387522	1.48707	5.155209	2.206935	4.439388	1.260361	1.17497	-6.31411	1.011821	-6.783941	-1.161243	-5.509607	190.8563	0.000118	0.002338
3800608	OGG1		21.25719	NM	00254	Homo sapiens	2.543633	-0.803836	4.526424	3.172391	4.433345	2.991308	1.779511	-3.598481	1.742919	-4.003434	-1.020995	-5.660653	59.87123	4.71E-05	0.001172
7380577	ZA20D3		37.34899	NM	01900	Homo sapiens	3.458731	3.713385	5.072967	6.344556	4.430643	5.390084	1.466713	-4.323673	1.281003	-5.76181	-1.144973	-5.37653	1008.572	2.62E-06	0.000145
2340670	FOXD3		14.15233	NM	01218	Homo sapiens	1.247636	-6.364572	3.584506	-0.653835	4.429996	0.587596	2.873039	-1.620193	3.550713	-0.481188	1.235874	-5.39851	143.6272	0.000323	0.00503
5670372	ARL4		13.54161	NM	21246	Homo sapiens	1.811662	-4.088052	2.678514	-1.508263	4.425269	1.941869	1.478484	-5.260952	2.442657	-1.950753	1.652136	-3.937601	55.78203	0.000394	0.005823
940767	HSPD1		22.12347	NM	00215	Homo sapiens	2.940559	0.411634	4.689235	3.493161	4.416222	3.054605	1.594675	-4.421378	1.501831	-5.116217	-1.06182	-5.627972	77.36346	3.87E-05	0.001005
5290215	HSPB8		10.94113	NM	01436	Homo sapiens	1.504294	-5.621291	2.587664	-2.677164	4.415564	0.683351	1.720185	-4.751568	2.935306	-1.594415	1.70639	-4.138131	128.1465	0.000997	0.011673
4850176	LRBA		36.63183	NM	00672	Homo sapiens	3.683499	4.11017	4.959776	6.10462	4.413777	5.272784	1.346485	-5.122455	1.198257	-6.227583	-1.123703	-5.452917	247.0328	2.91E-06	0.000154
4050564	SPAG9		22.75582	NM	17234	Homo sapiens	4.65232	2.837694	5.355363	3.50173	4.40708	2.2407	1.151117	-6.324549	-1.055647	-6.756277	-1.215173	-5.342093	108.5503	3.36E-05	0.000905
4060255	C3orf63		44.08303	NM	01522	Homo sapiens	5.416759	7.048087	4.477021	5.796798	4.404546	5.650913	-1.209903	-5.860081	-1.229811	-6.013326	-1.016455	-5.660468	67.7245	1.08E-06	8.02E-05
3170064		0	31.39241	AK129542		Homo sapiens	1.731466	-2.793719	3.599358	3.849183	4.398922	5.322013	2.078792	-0.776223	2.540577	1.01348	1.222141	-5.053947	1526.134	6.53E-06	0.000274
240131	CYP2S1		42.27238	NM	03062	Homo sapiens	1.98536	-0.206938	3.806622	5.892447	4.396494	6.964668	1.917346	-0.503781	2.214457	0.963781	1.154959	-5.228725	3874.629	1.36E-06	9.24E-05
1030142	HCP5		30.37816	NM	00667	Homo sapiens	2.168754	-0.383691	3.676169	4.118558	4.393	5.419106	1.695061	-2.818116	2.025587	-1.154035	1.194994	-5.167579	177.8795	7.75E-06	0.000309
7150470	BEX1		44.08319	NM	01847	Homo sapiens	2.821415	3.661621	4.004932	6.521663	4.391557	7.192619	1.419476	-3.994049	1.556509	-3.102983	1.096537	-5.47388	1909.43	1.08E-06	8.02E-05
510142	CHMP6		18.60945	NM	02459	Homo sapiens	2.543639	-0.767615	3.664974	1.761861	4.390613	2.974224	1.440839	-5.159644	1.726115	-4.060274	1.197993	-5.341103	116.8294	8.99E-05	0.001911
3310026	PPIF		58.65872	NM	00572	Homo sapiens	1.463536	-2.902894	2.80542	5.192306	4.380784	9.040693	1.916878	1.034892	2.993286	5.898415	1.561543	-1.610194	103.3128	2.28E-07	2.88E-05
2570398	YWHAB		67.17953	NM	00340	Homo sapiens	2.699501	5.180016	4.190106	9.012269	4.380383	9.343979	1.552178	-1.718493	1.622664	-1.249427	1.045411	-5.598155	531.471	1.08E-07	1.84E-05
2940563	LOC644608		30.32871	XM	92772	PREDICTED:	3.200638	1.868195	5.637087	5.604708	4.378613	3.925363	1.761238	-3.195352	1.368044	-5.549989	-1.287414	-4.928777	510.2581	7.81E-06	0.000311
2970152	LOC150223		12.9332	NM	00101	Homo sapiens	1.836209	-4.619565	4.379284	0.469631	4.365247	0.398154	2.384959	-2.869034	2.377314	-3.114823	-1.003216	-5.664973	32.77435	0.000483	0.006759
6620386	CASP6		12.73456	NM	00122	Homo sapiens	5.958065	1.411673	4.269287	-0.614883	4.361141	-0.552171	-1.395565	-5.953648	-1.366171	-6.286129	1.021515	-5.662875	97.98481	0.000517	0.007111
70593	ZNF567		27.2577	NM	15260	Homo sapiens	5.647038	3.770082	7.007648	4.808999	4.35138	1.958202	1.240942	-6.09394	-1.297758	-6.175439	-1.610443	-4.048213	115.4475	1.35E-05	0.000462
3130040	PSMA1		50.6883	NM	00278	Homo sapiens	3.764536	6.119576	4.577853	7.5568	4.343187	7.157912	1.216047	-5.603882	1.153711	-6.276872	-1.054031	-5.601367	812.9861	5.08E-07	4.85E-05
2120619	C21orf33		49.61804	NM	00464	Homo sapiens	2.725693	4.094516	3.824474	7.001773	4.340588	7.961683	1.40312	-3.803299	1.592471	-2.337473	1.13495	-5.252292	1109.665	5.70E-07	5.25E-05
3120097	RGS7		12.248	NM	00292	Homo sapiens	2.365293	-3.020442	4.357541	0.510389	4.339487	0.433291	1.842284	-4.413033	1.834651	-4.685122	-1.004161	-5.66493	59.372	0.000614	0.008121
130463	B4GALT6		78.17975	NM	00477	Homo sapiens	2.539425	5.068342	4.527153	10.14632	4.335939	9.840019	1.782747	0.716034	1.707449	-0.075341	-1.0441	-5.594947	580.9383	4.62E-08	1.06E-05
3400307	VMD2L1		25.15708	NM	01768	Homo sapiens	1.680026	-3.661888	3.582607	2.732527	4.332656	4.084643	2.132471	-1.304809	2.578921	0.225552	1.209359	-5.215827	58.87111	2.03E-05	0.000627
10292	KHK		21.66352	NM	00022	Homo sapiens	1.343901	-5.849263	4.017426	1.908158	4.329357	2.362599	2.989377	0.166474	3.221486	0.555791	1.077645	-5.61371	53.91226	4.29E-05	0.001094
4490040	C14orf115		41.04169	NM	01822	Homo sapiens	2.183787	1.181479	3.413855	5.37919	4.328966	7.245198	1.563273	-2.742977	1.982321	0.003508	1.268058	-4.484671	1559.013	1.59E-06	0.000103
7150017	CCT7		46.17022	NM	00642	Homo sapiens	3.092886	4.479584	4.333979	7.107879	4.313639	7.053051	1.401274	-4.147778	1.394697	-4.447208	-1.004715	-5.664528	2835.159	8.43E-07	6.69E-05
870241	CNN2		11.31228	NM	00436	Homo sapiens	2.012707	-4.079339	3.864436	-0.293854	4.304388	0.302682	1.920019	-4.206026	2.138606	-3.791438	1.113846	-5.59638	198.7467	0.000865	0.010497
1690156	LOC642464		75.11324	XM	92624	PREDICTED:	1.53903	-1.440418	3.257538	7.77398	4.294333	10.05889	2.116618	3.237201	2.790286	6.310116	1.318276	-3.294076	1143.501	5.78E-08	1.22E-05
5820681	ZNF206		27.25192	NM	03280	Homo sapiens	1.72913	-3.083063	3.494378	3.120966	4.287331	4.622337	2.020888	-1.404501	2.479473	0.365441	1.226923	-5.087408	5266.589	1.35E-05	0.000462
4640491	LOC648695		52.89072	XM	94424	PREDICTED:	4.389974	7.091695	4.720803	7.638399	4.283141	6.919762	1.07536	-6.383733	-1.024943	-6.76972	-1.102183	-5.455802	2951.599	4.03E-07	4.21E-05
7330767	VASP		40.37939	NM	00100	Homo sapiens	1.807957	-0.987448	3.030053	4.403754	4.282917	7.257974	1.675954	-1.815345	2.368926	2.099197	1.413479	-3.40207	899.4067	1.73E-06	0.000108
5340129	NUP35		56.28297	NM	13828	Homo sapiens	3.165785	5.527031	4.525122	8.34551	4.282083	7.92937	1.429384	-3.532751	1.352613	-4.48982	-1.056757	-5.582859	820.6198	2.86E-07	3.36E-05
7160671	NFATC2IP		27.36628	NM	03281	Homo sapiens	2.282471	0.258247	3.02843	2.666729	4.279694	5.40081	1.326821	-5.165685	1.875027	-1.859322	1.413173	-3.960795	78.94801	1.33E-05	0.000456
5550521	GPD2		24.34804	NM	00040	Homo sapiens	3.630133	2.537726	4.319725	3.57057	4.27393	3.45536	1.189963	-6.133724	1.177348	-6.445138	-1.010715	-5.663705	139.2945	2.40E-05	0.000703
4640452	PARP9		28.77331	NM	03145	Homo sapiens	1.860968	-1.887338	3.169149	3.027173	4.272675	5.351922	1.702957	-2.698826	2.295943	0.22633	1.348209	-4.347747	47.02508	1.03E-05	0.000378
940039	RAB25		20.7152	NM	02038	Homo sapiens	1.878293	-3.001157	3.557482	2.035975	4.265843	3.298939	1.893997	-2.785956	2.271128	-1.406762	1.199119	-5.303097	1361.207	5.34E-05	0.001287
1110307	GRHL2		11.3314	NM	02491	Homo sapiens	1.359296	-5.997244	1.749703	-5.006429	4.263289	0.94308	1.287213	-6.041085	3.136395	-0.769488	2.436579	-1.99648	41.82485	0.000859	0.010442
6580491	PAPSS2		37.21965	NM	00101	Homo sapiens	2.29243	1.079655	3.806234	5.514885	4.256462	6.341709	1.660349	-2.429093	1.856746	-1.326068	1.118287	-5.415696	877.9976	2.67E-06	0.000146
1440605	LARP2		27.74344	NM	17804	Homo sapiens	1.609751	-3.458004	2.7262	1.648189	4.2522	5.280574	1.693554	-2.776688	2.641527	1.547818	1.559754	-3.135336	68.09906	1.24E-05	0.000434
510121	HBXIP		14.34945	NM	00640	Homo sapiens	1.824915	-4.113319	3.511257	0.291074	4.245535	1.504273	1.924066	-3.587731	2.326429	-2.431204	1.209121	-5.387513	41.15794	0.000303	0.00479
160278	ANKRD17		46.8688	NM	03221	Homo sapiens	4.327705	6.690866	4.258066	6.57193	4.24469	6.524458	-1.016355	-6.508257	-1.019557	-6.776122	-1.003151	-5.664825	101.5473	7.77E-07	6.42E-05
1230240	C7orf28B		94.60312	XM	94226	PREDICTED:	3.741597	8.764103	5.347513	11.52061	4.242686	9.934259	1.429206	-2.537832	1.133924	-6.119542	-1.260407	-3.896064	457.8033	1.58E-08	5.53E-06
3450349	SPTLC2		19.4305	NM	00486	Homo sapiens	2.777379	-0.168222	4.245064	2.666735	4.24212	2.616871	1.528443	-4.788014	1.527382	-5.045327	-1.000694	-5.66503	112.312	7.30E-05	0.001637
240035	CDH1		21.25404	NM	00436	Homo sapiens	1.330354	-5.74424	2.954798	0.526857	4.227979	3.168427	2.221061	-1.450332	3.178085	1.307926	1.430886	-4.399599	36.18289	4.71E-05	0.001172
5390315	LOC650230		14.09459	XM	94194	PREDICTED:	2.725767	-1.506138	4.271834	1.191443	4.220493	1.064826	1.567205	-5.074765	1.548369	-5.399743	-1.012165	-5.663975	224.4625	0.000329	0.005093
4610050	C15orf44		24.55222	XM	94054	PREDICTED:	4.702394	4.195581	3.429343	1.807021	4.203502	3.24802	-1.371223	-5.359736	-1.118685	-6.625231	1.225746	-5.209884	115.0589	2.30E-05	0.000686
160735	NSF		24.40228	XM	93819	PREDICTED:	2.775046	1.131076	3.751317	3.330626	4.19991	4.121609	1.351804	-5.27195	1.513456	-4.592922	1.119583	-5.493541	74.10275	2.37E-05	0.000698
4070504	TAF13		23.89916	NM	00564	Homo sapiens	2.023526	-1.405889	2.97471	1.9364	4.198398	4.634254	1.470062	-4.428754	2.074793	-1.228409	1.411364	-4.134879	54.20985	2.63E-05	0.000759
510767	MDK		13.86956	NM	00101	Homo sapiens	3.43319	-0.284675	4.52853	1.160409	4.196073	0.643536	1.319044	-5.973066	1.222208	-6.492058	-1.07923	-5.626483	197.0575	0.000353	0.005391
2970240	TAP2		13.72719	NM	00054	Homo sapiens	1.397912	-5.865265	3.307571	-0.604365	4.191947	0.885862	2.366079	-2.450598	2.99872	-1.031448	1.267379	-5.284697	48.66263	0.00037	0.005574
2450670	GRB2		38.56277	NM	00208	Homo sapiens	2.514887	2.387925	3.647327	5.595449	4.191469	6.658188	1.450295	-3.815391	1.666663	-2.353663	1.149189	-5.253869	479.6902	2.21E-06	0.000129
20095	LOC91614		26.87198	NM	13916	Homo sapiens	2.641449	0.17177	5.182391	4.910277	4.189523	3.450863	1.96195	-2.314607	1.58607	-4.429073	-1.236988	-5.148509	74.33938	1.46E-05	0.000486
5570152	SH3GL2		17.95231	NM	00302	Homo sapiens	-1.108633	-6.600844	2.758303	-1.617179	4.18221	1.166954	3.057945	-0.472542	4.636536	2.048096	1.516226	-4.509134	190.1218	0.000107	0.002166

4230433	LOC650111		18.26687	XM	93920	PREDICTED:	1.382305	-5.660523	3.178293	0.396973	4.181787	2.305858	2.299271	-1.702319	3.025228	0.243493	1.315734	-4.992095	117.8204	9.82E-05	0.002038
3870687	LOC653519		47.68127	XM	92786	PREDICTED:	3.966151	6.137919	4.67769	7.312208	4.181545	6.477628	1.179403	-5.897466	1.054308	-6.718102	-1.118651	-5.401768	221.6276	7.08E-07	6.09E-05
4780242	CHDH		25.1888	NM	01839	Homo sapiens	2.456901	-0.346646	4.601591	4.266506	4.171559	3.549422	1.872925	-2.658326	1.697895	-3.776337	-1.103087	-5.547644	103.4794	2.02E-05	0.000625
6770465	ZNF611		48.2922	NM	03097	Homo sapiens	4.212506	5.566749	6.368826	8.200694	4.157722	5.365743	1.511885	-3.855002	-1.013176	-6.781659	-1.531807	-3.204316	54.65448	6.61E-07	5.85E-05
2320026	PCDHAC2		20.21618	NM	03188	Homo sapiens	1.278061	-5.952075	2.445696	-0.911296	4.155389	3.234071	1.913599	-2.629407	3.251323	1.650324	1.699062	-3.160341	31.9408	6.02E-05	0.001404
3370202	EDIL3		20.28545	NM	00571	Homo sapiens	1.268901	-6.231859	4.82204	2.096126	4.149829	1.110838	3.80017	0.94	3.270411	-0.195105	-1.161985	-5.498764	31.6752	5.92E-05	0.001387
4050497	SMN1		48.71687	XM	93695	PREDICTED:	2.520536	2.428746	5.070799	8.047186	4.14368	6.592938	2.011794	0.096511	1.643968	-2.511021	-1.223743	-4.831596	568.221	6.30E-07	5.64E-05
2480543	ABCC1		36.19975	NM	01990	Homo sapiens	4.383148	5.146611	4.919108	5.840846	4.142333	4.607864	1.122277	-6.292543	-1.058135	-6.730551	-1.187521	-5.231946	176.0293	3.10E-06	0.000162
3170471	MCC		28.23545	NM	00238	Homo sapiens	5.006618	4.468676	4.920997	4.225671	4.139489	3.023383	-1.017399	-6.510747	-1.209477	-6.346174	-1.188793	-5.339871	113.3798	1.13E-05	0.000404
7160440	ELOVL7		38.9203	NM	02493	Homo sapiens	1.811986	-2.203021	4.981727	6.399481	4.125091	5.043879	2.749319	2.007472	2.276558	0.102647	-1.207665	-5.101837	52.66249	2.11E-06	0.000124
380458	LOC440132		15.52475	XM	49856	PREDICTED:	1.683101	-4.551216	3.564106	0.677018	4.123554	1.616405	2.117583	-2.721041	2.449974	-1.832491	1.156967	-5.488708	874.3133	0.000211	0.003602
2940326	BAT1		16.59614	NM	00464	Homo sapiens	2.655416	-1.367867	4.805493	2.355616	4.121539	1.335283	1.809695	-4.006479	1.552126	-5.266111	-1.165946	-5.48099	57.74154	0.000155	0.002849
270403	RPL29		44.9425	NM	00099	Homo sapiens	2.063293	1.378249	3.051696	5.401258	4.116934	7.931066	1.479042	-2.932547	1.995322	0.909487	1.349064	-3.594717	1857.8	9.75E-07	7.47E-05
6620521	PRCC		85.10633	NM	00597	Homo sapiens	2.965126	7.806773	3.702285	9.90693	4.115529	10.76308	1.24861	-4.322242	1.387978	-2.68792	1.111619	-5.151898	367.0005	2.87E-08	8.22E-06
2600520	MBP		54.12894	NM	00102	Homo sapiens	1.166779	-6.001396	2.878722	4.593403	4.113527	7.666425	2.467238	3.211143	3.525541	6.478545	1.428942	-3.033905	341.0749	3.55E-07	3.89E-05
5810709	DNAPTP6		19.66713	NM	01553	Homo sapiens	1.767648	-3.634723	3.574239	1.870569	4.113518	2.835355	2.022031	-2.344668	2.327114	-1.348634	1.150879	-5.454077	3185.781	6.88E-05	0.001565
160403	ELMO2		23.45389	NM	02208	Homo sapiens	2.866698	1.209859	3.85226	3.306474	4.113233	3.743525	1.343797	-5.363632	1.434833	-5.120838	1.067746	-5.609163	53.36464	2.89E-05	0.000812
7320445	ZWILCH		12.12782	NM	01797	Homo sapiens	4.197975	-0.745457	6.51272	1.204845	4.111205	-1.221002	1.551396	-5.645104	-1.021106	-6.782901	-1.584139	-4.816881	35.02332	0.000641	0.008401
6020475		0	42.90905	CR627403		Homo sapiens	-1.065204	-6.617775	3.585103	3.823454	4.10573	4.826324	3.818867	4.433848	4.373441	5.402705	1.14522	-5.376216	100.1143	1.25E-06	8.79E-05
3800600	C14orf24		51.20501	NM	17360	Homo sapiens	4.859229	7.02052	5.524174	7.886241	4.100949	5.803455	1.136842	-6.168971	-1.184904	-6.186973	-1.347048	-4.181772	100.4895	4.80E-07	4.68E-05
4730747	KCMF1		19.87892	NM	02012	Homo sapiens	3.624301	1.441271	4.754194	3.01078	4.100581	1.996211	1.311755	-5.804353	1.131413	-6.629689	-1.159395	-5.467296	126.8873	6.53E-05	0.001496
4560364	LOC645625		16.05174	XM	93051	PREDICTED:	3.735955	0.699209	4.716386	1.907225	4.098947	0.979651	1.262431	-6.083421	1.097162	-6.714087	-1.150634	-5.521129	82.531	0.000181	0.003199
7210121	CCNC		22.79589	NM	00519	Homo sapiens	2.949306	0.88019	4.481104	3.724176	4.094283	3.058909	1.519375	-4.614164	1.388219	-5.535585	-1.094478	-5.572262	412.6937	3.33E-05	0.000901
4610358	SRP9		27.46981	NM	00313	Homo sapiens	4.070947	3.52998	4.809504	4.4956	4.086015	3.338608	1.181421	-6.149954	1.003701	-6.784846	-1.177065	-5.351213	473.3795	1.30E-05	0.000451
6270301	AP1S1		15.12168	NM	00128	Homo sapiens	2.300047	-2.223144	3.887401	1.288558	4.084117	1.569706	1.69014	-4.35731	1.775667	-4.252305	1.050603	-5.644432	550.0708	0.000238	0.003969
2850739	C2orf15		18.67485	NM	14470	Homo sapiens	2.02599	-2.500654	3.619032	1.906169	4.082629	2.723923	1.786303	-3.425982	2.015128	-2.626828	1.1281	-5.510798	279.8561	8.84E-05	0.001887
510753	LOC647743		17.27132	XM	93680	PREDICTED:	3.286378	0.39684	4.555134	2.323406	4.079996	1.5598	1.386065	-5.587627	1.241487	-6.356729	-1.116455	-5.56333	213.9091	0.000128	0.002491
20224	MGC17624		15.411	NM	20696	Homo sapiens	1.082721	-6.627359	1.762978	-4.491328	4.079777	1.761574	1.628285	-4.541082	3.768078	1.467219	2.314139	-1.690273	64.46645	0.000219	0.003703
7050711	ZNF525		33.53787	XM	37564	PREDICTED:	1.760197	-2.190214	3.614723	4.601025	4.07685	5.505413	2.05359	-0.378382	2.316133	0.714761	1.127846	-5.403085	506.6514	4.63E-06	0.000215
4260538	SLC30A2		23.56293	NM	00100	Homo sapiens	1.399259	-5.183907	2.889213	1.173147	4.076624	3.852168	2.064817	-1.467989	2.913417	1.442597	1.410981	-4.275609	82.48016	2.83E-05	0.000799
1820053	TASP1		44.12583	NM	01771	Homo sapiens	2.610937	2.926862	4.348047	7.145201	4.074224	6.635793	1.66532	-2.038883	1.560445	-3.068164	-1.067209	-5.568791	138.8891	1.08E-06	8.00E-05
5420386	RAD51L1		23.69806	NM	13351	Homo sapiens	1.606841	-3.631943	2.472819	0.439243	4.073971	4.63434	1.538932	-3.911522	2.535391	0.887176	1.647501	-2.788879	53.32443	2.75E-05	0.000785
7650497	C3orf38		50.96215	NM	17382	Homo sapiens	3.643661	6.290999	4.303326	7.563124	4.067607	7.11913	1.181045	-5.786591	1.116352	-6.452771	-1.05795	-5.585783	316.5553	4.93E-07	4.74E-05
6020161	NDUFV3		38.14503	NM	02107	Homo sapiens	2.279566	1.55417	3.483549	5.433043	4.065446	6.649659	1.528163	-3.086099	1.78343	-1.372981	1.167041	-5.142138	197.8892	2.35E-06	0.000134
2600204	EPHX1		14.89342	NM	00012	Homo sapiens	1.4235	-5.546207	2.688442	-1.124244	4.061626	1.82736	1.888613	-3.413838	2.853267	-0.434846	1.510773	-4.327038	594.6248	0.000256	0.0042
5900228	BRAF		54.52972	NM	00433	Homo sapiens	2.072441	2.3131	3.127193	6.667746	4.06023	8.889963	1.508942	-2.072791	1.959154	1.535249	1.298363	-3.723545	188.6763	3.41E-07	3.80E-05
6280270	Kua-UEV		43.9925	NM	19920	Homo sapiens	2.402867	2.531813	3.789331	6.608842	4.059709	7.13061	1.577004	-2.434092	1.689527	-1.743398	1.071352	-5.546293	148.6928	1.09E-06	8.06E-05
4120092	C20orf44		25.61389	NM	01824	Homo sapiens	3.039365	1.906471	4.097652	4.003037	4.055801	3.888943	1.348193	-5.287523	1.334424	-5.622029	-1.010319	-5.663588	109.2843	1.86E-05	0.000588
5910450	NANOS1		16.80868	NM	19946	Homo sapiens	2.092604	-1.952868	2.668841	-0.133121	4.036096	3.057074	1.275368	-5.790021	1.928744	-2.76268	1.512303	-3.98193	58.11138	0.000146	0.002729
5900338	NFX1		23.55557	NM	14713	Homo sapiens	2.640723	0.605133	3.886035	3.478343	4.035834	3.714934	1.47158	-4.627398	1.528307	-4.549348	1.038548	-5.645959	281.7384	2.83E-05	0.0008
6620347	LOC441019		19.08256	XM	49896	PREDICTED:	1.90134	-2.396809	2.501171	-0.124745	4.034444	3.753	1.315478	-5.483602	2.121895	-1.423821	1.613022	-3.278001	311.0346	7.96E-05	0.001743
2640292	LOC136306		37.15699	NM	17495	Homo sapiens	2.346502	1.980345	3.243932	4.951935	4.03222	6.725374	1.382454	-4.240063	1.718396	-1.748784	1.243004	-4.653497	59.90433	2.70E-06	0.000147
6400072	SNRPN		31.3207	NM	02280	Homo sapiens	2.583578	1.423294	4.179784	5.223709	4.032104	4.922967	1.617827	-3.240504	1.560667	-3.8524	-1.036626	-5.643217	5568.851	6.61E-06	0.000275
3850561	PDF		12.55671	NM	02234	Homo sapiens	1.932468	-3.878817	3.551746	0.051156	4.028555	0.826014	1.837933	-4.062975	2.084669	-3.437018	1.134247	-5.5498	85.42455	0.00055	0.007456
1710551	PLS1		36.69081	NM	00267	Homo sapiens	2.443538	1.631417	4.13452	6.029192	4.016588	5.781416	1.692022	-2.278431	1.643759	-2.833525	-1.029361	-5.648324	292.1104	2.88E-06	0.000153
6100382	CNTN1		21.02872	NM	00184	Homo sapiens	1.257358	-5.970589	2.213044	-1.39387	4.014942	3.545921	1.760074	-3.081367	3.193157	2.01538	1.814217	-2.42726	28.74145	4.96E-05	0.001219
4180687	FLJ20160		28.24863	NM	01769	Homo sapiens	1.089447	-6.568842	2.57092	0.549729	4.00929	4.210364	2.35984	0.126193	3.680116	3.74147	1.559477	-3.36701	43.94932	1.13E-05	0.000403
2850288	HIF1A		33.44919	NM	00153	Homo sapiens	4.65083	4.771334	5.467712	5.702536	4.008763	3.573479	1.175642	-6.144913	-1.160166	-6.469136	-1.36394	-4.538833	1074.883	4.69E-06	0.000217
4120112	UNC13A		35.35063	XM	03860	PREDICTED:	1.728229	-2.28112	3.735072	5.055917	4.003587	5.564635	2.161214	0.304144	2.316583	0.877023	1.07189	-5.572963	95.65145	3.51E-06	0.000177
4610082	B3GNT1		15.58442	NM	00657	Homo sapiens	4.501634	1.422963	4.740386	1.481883	4.000574	0.391333	1.053037	-6.494564	-1.125247	-6.681083	-1.184926	-5.475006	74.48374	0.000207	0.003557
1410427	INPP5B		31.55935	NM	00554	Homo sapiens	2.705404	2.893738	2.840284	3.162965	3.998661	6.062858	1.049856	-6.45904	1.478027	-3.930399	1.407839	-3.656544	257.7132	6.36E-06	0.00027
4250463	BCR		17.854	NM	00432	Homo sapiens	2.689722	-0.668515	4.367246	2.541273	3.996367	1.897845	1.623679	-4.461026	1.485792	-5.325957	-1.092804	-5.591773	136.1625	0.00011	0.002208
1440224	FBLIM1		22.02016	NM	00102	Homo sapiens	1.823948	-2.909668	3.4801	2.483143	3.99609	3.480052	1.908004	-2.349544	2.190901	-1.268069	1.148269	-5.423632	107.6787	3.96E-05	0.001025
3180474	PPP2R1B		37.21586	NM	18169	Homo sapiens	1.336976	-4.877736	2.642438	2.716787	3.994734	6.338655	1.976429	-0.08612	2.987888	4.036809	1.511761	-2.848902	251.821	2.67E-06	0.000146
3060056	HAS3		51.0393	NM	00532	Homo sapiens	1.89324	0.250858	3.80814	7.269768	3.993848	7.631564	2.011441	1.09475	2.109531	1.544093	1.048766	-5.601067	2558.839	4.89E-07	4.74E-05
580709	LOC649095		22.59824	XM	94515	PREDICTED:	1.949942	-2.481626	4.033869	3.265605	3.993103	3.149798	2.068712	-1.8144	2.047806	-2.113552	-1.010209	-5.663784	180.8052	3.48E-05	0.000931
1740100	SLC22A2		42.44638	NM	00305	Homo sapiens	2.235104	2.748212	1.948306	0.795278	3.98735	8.180953	-1.147204	-5.889152	1.783966	-0.188704	2.046573	1.124229	30.32054	1.33E-06	9.12E-05
4730341	DFFA		37.65614	NM	21356	Homo sapiens	2.141306	1.185688	3.086981	4.756525	3.984516	6.89518	1.441635	-3.629135	1.860788	-0.56389	1.290749	-4.262816	139.541	2.51E-06	0.000141
1090372	FYN		22.36059	NM	15304	Homo sapiens	2.344123	-0.043977	3.088687	2.170743	3.984133	4.152778	1.31763	-5.376336	1.699626	-3.303746	1.289911	-4.786265	136.1771	3.67E-05	0.000967
7000739	CDCP1		23.04631	NM	17818	Homo sapiens	1.336224	-5.379762	1.816678	-2.74392	3.983699	4.398832	1.359561	-5.071836	2.98131	2.274202	2.192849	-0.477511	38.5465	3.16E-05	0.000863
1030280	FLJ22349		22.39963	NM	02482	Homo sapiens	1.374315	-5.282804	2.545135	0.197206	3.982814	3.854135	1.85193	-2.397554	2.898036	1.549801	1.564873	-3.44667	135.0023	3.64E-05	0.000961
1070128	LOC653094		13.16851	XM	92594	PREDICTED:	1.706067	-4.875439	4.145718	0.59428	3.971744	0.274482	2.429985	-2.452345	2.328011	-2.960126	-1.043803	-5.652889	36.47702	0.000446	0.006359
940059	C9orf52		32.34185	NM	15257	Homo sapiens	2.358466	0.962674	3.878455	5.165738	3.966321	5.305546	1.644482	-2.80881	1.681737	-2.779545	1.022655	-5.655704	314.8133	5.59E-06	0.000244
4640601	DAPK1		53.73241	NM	00493	Homo sapiens	2.356061	3.565617	3.543613	7.473278	3.965832	8.386088	1.504041	-2.300971	1.683246	-0.83905	1.119149	-5.264054	1356.558	3.69E-07	3.96E-05
1660019	CPT1A		23.76337	NM	00187	Homo sapiens	1.251308	-5.967052	2.697748	0.616415	3.961844	3.690579	2.155942	-1.031861	3.166163	2.168294	1.468575	-3.974025	37.83178	2.71E-05	0.000775
290091	UGP2		30.09946	NM	00675	Homo sapiens	1.519925	-3.842601	2.942721	2.813934	3.96002	5.257171	1.936096	-1.028115	2.605405	1.855813	1.3457	-4.256163	2291.94	8.13E-06	0.000318
2340619	RFC3		30.3985	NM	18155	Homo sapiens	2.798163	2.513453	3.583636	4.450515	3.958655	5.198807	1.28071	-5.384145	1.414733	-4.709291	1.104648	-5.487064	172.0294	7.72E-06	0.000308
1260112	LOC654174		32.56765	XM	94043	PREDICTED:	2.969534	3.192948	3.732649	4.944025	3.958	5.368337	1.256981	-5.506481	1.332869	-5.246328	1.060373	-5.60054	79.92572	5.40E-06	0.000238
6450095	FAHD2A		42.15369	NM	01604	Homo sapiens	2.263715	1.313924	4.323623	6.933628	3.955056	6.235449	1.909968	-0.499484	1.747153	-1.758054	-1.093189	-5.491428	49.96132	1.38E-06	9.34E-05
1090722	RNF39		20.29579	NM	02523	Homo sapiens	1.64866	-3.988711	3.264474	1.678506	3.947867	3.072339	1.980077	-2.204054	2.394592	-0.702836	1.209343	-5.244561	75.47006	5.91E-05	0.001386
1580243	ING5		11.45197	NM	03232	Homo sapiens	2.415102	-1.950515	2.600862	-1.719677	3.94722	1.205933	1.076916	-6.464565	1.63439	-4.884852	1.517658	-4.406988	126.6345	0.000821	0.010106
6590291		0	24.47902	BX111122		BX111122 Soa	4.812783	3.758132	4.799443	3.574719	3.946652	2.216493	-1.002779	-6.514365	-1.21946	-6.351157	-1.21608	-5.28912	158.0164	2.33E-05	0.000693
5900050	SERPINI1		33.65458	NM	00502	Homo sapiens	2.317592	0.652881	4.269894	5.721698	3.943454	5.095358	1.842384	-1.651555	1.701531	-2.740902	-1.08278	-5.552241	139.0341	4.54E-06	0.000213
3450050	MEIS3		30.67167	NM	00100	Homo sapiens	2.434902	1.651093	3.168666	3.930208	3.941167	5.70094	1.301353	-5.127421	1.618614	-2.979379	1.243794	-4.784277	1043.489	7.37E-06	0.000298
3710653	C15orf44		71.79243	NM	03080	Homo sapiens	3.922049	8.06027	4.95821	9.884661	3.938056	8.192156	1.264189	-4.816435	1.004081	-6.784436	-1.25905	-4.200193	130.7799	7.45E-08	1.43E-05
1940064	HIST1H2AG		27.6873	NM	02106	Homo sapiens	1.986007	-1.946043	4.575957	4.732655	3.931177	3.61402	2.304099	-0.434582	1.979438	-2.054835	-1.164017	-5.360637	58.06981	1.25E-05	0.000437
4290553	SEC23A		28.88944	NM	00636	Homo sapiens	5.400658	4.615795	5.525878	4.630298	3.929618	2.352752	1.023186	-6.508304	-1.374347	-5.692084	-1.406212	-4.568303	122.5831	1.00E-05	0.000374

510128	DUSP5		16.29687	NM	00441	Homo sapiens	2.236523	-1.462299	2.872703	0.362862	3.923495	2.715222	1.284451	-5.769672	1.754283	-3.679012	1.365785	-4.664906	2688.123	0.000169	0.003035
460465	DPPA4		33.30786	NM	01818	Homo sapiens	1.83664	-1.253139	3.199435	4.251336	3.920261	5.905632	1.742005	-1.753584	2.134475	0.404903	1.225298	-4.858777	11766.38	4.80E-06	0.00022
1690072	TNRC6A		39.78437	NM	02084	Homo sapiens	2.363789	0.748961	5.401248	7.197768	3.912564	4.91021	2.284995	0.502464	1.655209	-3.114573	-1.380488	-4.10727	52.43224	1.88E-06	0.000114
6520332	TCF7		19.90144	NM	20163	Homo sapiens	2.208073	-0.629594	2.499585	0.261396	3.910687	3.994169	1.132021	-6.267634	1.771086	-2.899708	1.564535	-3.351205	47.97956	6.50E-05	0.001491
4810184	LOC648154		18.9904	XM	94387	PREDICTED:	2.87151	0.099465	4.383002	2.892637	3.909155	2.064163	1.526375	-4.794092	1.361359	-5.800034	-1.121215	-5.53556	55.21903	8.15E-05	0.001776
430066	LOC643995		43.49153	XM	93015	PREDICTED:	3.528826	5.435071	4.262885	6.838986	3.905739	6.146702	1.208018	-5.689795	1.10681	-6.533278	-1.091441	-5.497211	3481	1.16E-06	8.34E-05
620128	DRCTNNB1A		9.152425	NM	03258	Homo sapiens	1.942626	-4.110406	2.737041	-2.178512	3.904906	0.092287	1.408938	-5.720733	2.010117	-3.978329	1.426689	-4.904785	49.23661	0.002082	0.020847
6020280	MCL1		37.6799	NM	02196	Homo sapiens	2.189989	0.637212	4.146816	6.226477	3.903415	5.735394	1.893533	-0.874016	1.78239	-1.771749	-1.062356	-5.590017	368.8362	2.50E-06	0.000141
70601		0	34.02962	DA077127		DA077127 BR	1.906259	-1.572392	4.392413	5.671967	3.902704	4.763835	2.304206	0.48576	2.047311	-0.884881	-1.125479	-5.430413	268.3352	4.29E-06	0.000203
2940114	TPD52		12.39794	NM	00102	Homo sapiens	2.659024	-1.787451	4.024887	0.671481	3.901699	0.420716	1.513671	-5.304188	1.467343	-5.724834	-1.031573	-5.658254	100.4518	0.000582	0.007798
1050296	C14orf151		21.31246	NM	03271	Homo sapiens	1.953111	-1.868613	2.933504	1.66547	3.899581	3.923941	1.501964	-4.289883	1.996599	-1.725109	1.329325	-4.60151	318.5637	4.65E-05	0.00116
4670017	KRTCAP3		29.13002	NM	17385	Homo sapiens	1.5002	-4.16563	3.240501	3.238529	3.891107	4.675143	2.160045	-0.204008	2.593725	1.427772	1.200773	-5.126779	355.4089	9.62E-06	0.000362
1400504	TRIM37		34.29457	NM	00100	Homo sapiens	3.374839	4.204832	3.977863	5.374365	3.887022	5.164259	1.178682	-5.979246	1.151765	-6.381427	-1.02337	-5.655086	178.2744	4.12E-06	0.000198
6100008	ARRB1		14.67633	NM	00404	Homo sapiens	1.371936	-5.688191	2.387181	-1.789746	3.886613	1.88632	1.740009	-3.869548	2.83294	-0.185441	1.628118	-3.769072	365.9081	0.000273	0.004432
3360020	ADAM10		46.24746	NM	00111	Homo sapiens	3.44439	5.25839	4.729904	7.601568	3.882904	6.117541	1.37322	-4.423184	1.127313	-6.435621	-1.218136	-4.862394	343.6048	8.35E-07	6.66E-05
4610678	LOC651559		29.33309	XM	94073	PREDICTED:	3.230753	3.158006	3.996711	4.656149	3.882211	4.399831	1.237083	-5.753061	1.201643	-6.203117	-1.029493	-5.651407	5119.673	9.28E-06	0.000353
3890315	MTX1		30.67137	NM	00245	Homo sapiens	2.139067	0.113878	3.452225	4.435308	3.881914	5.345655	1.613893	-2.912543	1.81477	-1.785262	1.124467	-5.405548	384.8237	7.37E-06	0.000298
4210136	FANCA		11.65937	NM	00101	Homo sapiens	2.226304	-3.423071	4.49569	0.667217	3.876951	-0.276373	2.019351	-3.878609	1.741429	-4.995139	-1.159594	-5.534718	46.73066	0.00076	0.009511
6520739	C10orf118		13.57315	NM	01801	Homo sapiens	2.857394	-0.233706	2.792203	-0.707428	3.870046	1.644639	-1.023348	-6.508957	1.354397	-5.893351	1.386019	-4.76035	101.3688	0.00039	0.005775
3130475	FGD4		14.01886	NM	13924	Homo sapiens	1.998314	-3.208312	3.462423	0.62809	3.869884	1.352381	1.732672	-4.122512	1.936574	-3.532635	1.117681	-5.557973	98.19762	0.000337	0.005191
4490152	LOC648140		35.64577	XM	93864	PREDICTED:	2.699863	3.040408	2.580676	5.398333	3.869195	5.994126	1.326244	-4.828231	1.433108	-4.21432	1.080577	-5.53482	891.7522	3.36E-06	0.000171
4480482	CCRN4L		31.22742	NM	01211	Homo sapiens	1.340516	-4.96968	2.358302	1.170714	3.86624	5.665912	1.759249	-1.718671	2.884143	3.335091	1.639417	-2.214628	1404.872	6.72E-06	0.000279
3390132	LOC653252		38.87898	XM	93262	PREDICTED:	3.008909	4.024148	3.981526	6.23424	3.864714	5.974449	1.323246	-4.854647	1.284424	-5.41103	-1.030225	-5.645634	759.2623	2.12E-06	0.000124
3450687	LYPLA2		22.1541	NM	00726	Homo sapiens	1.387537	-5.164198	2.563146	0.398338	3.857085	3.775705	1.847264	-2.327538	2.779808	1.333633	1.504825	-3.702339	700.3874	3.84E-05	0.000999
4730372		0	25.50644	BX374545		BX374545 Ho	1.66347	-3.48695	3.614813	3.346654	3.855001	3.798654	2.173055	-0.734885	2.317445	-0.300831	1.066446	-5.605304	39.84388	1.90E-05	0.000596
4390446	PPP2CB		14.08724	NM	00100	Homo sapiens	2.95075	-0.872108	4.418714	1.510297	3.850391	0.578465	1.497488	-5.301046	1.304886	-6.222832	-1.147601	-5.526269	51.61014	0.000329	0.005103
5310554	AGXT2L1		14.37902	NM	03127	Homo sapiens	1.845129	-3.331476	2.541442	-0.932823	3.847353	2.246403	1.377379	-5.409343	2.08514	-2.369408	1.513847	-4.106555	64.3754	0.0003	0.004761
4570494	BCL2L12		25.57504	NM	13863	Homo sapiens	2.111801	-0.740475	3.494739	3.60017	3.843847	4.309493	1.654862	-3.588129	1.820175	-2.355781	1.099895	-5.521087	361.1366	1.87E-05	0.000591
3190392	KAL1		67.32185	NM	00021	Homo sapiens	2.973354	5.952779	4.835969	9.880371	3.839512	8.155624	1.626436	-1.132059	1.291307	-4.738819	-1.259527	-4.156762	5725.571	1.06E-07	1.83E-05
6060243	PPP4R2		43.50122	NM	17490	Homo sapiens	3.686702	5.483054	4.628306	7.117053	3.829006	5.677119	1.255406	-5.41362	1.038599	-6.751379	-1.208749	-4.961314	792.0438	1.16E-06	8.34E-05
50647	THBS2		20.62741	NM	00324	Homo sapiens	1.842554	-2.719204	3.232773	2.055824	3.826774	3.325817	1.754507	-3.040929	2.076886	-1.65297	1.183744	-5.29833	1490.878	5.46E-05	0.001305
4490494	C9orf85		66.11067	NM	18250	Homo sapiens	2.494109	5.167472	3.738361	8.999304	3.825524	9.186365	1.498876	-1.725335	1.533824	-1.549348	1.023316	-5.643724	434.6208	1.18E-07	1.92E-05
4290433	MRPS12		37.02116	NM	02110	Homo sapiens	1.597156	-2.745224	3.259065	4.74022	3.822382	6.043823	2.040543	0.315583	2.393243	1.921063	1.172846	-5.12171	79.45122	2.75E-06	0.000149
3190093	HTR2C		63.63522	NM	00086	Homo sapiens	3.771344	6.998995	5.387138	9.594626	3.821114	7.107709	1.42844	-3.513047	1.013197	-6.779742	-1.409834	-3.177022	32.32612	1.46E-07	2.17E-05
1450541	PHLDA2		11.04991	NM	00331	Homo sapiens	-1.062294	-6.664726	2.012	-4.403346	3.819914	-0.343547	2.137335	-3.516027	4.057873	0.299941	1.898566	-3.651117	78.16303	0.000956	0.011316
5870577	ANKS6		40.79553	NM	17355	Homo sapiens	1.189733	-6.020608	3.230856	4.280975	3.819624	5.642366	2.715614	2.908634	3.210488	4.347229	1.182233	-5.109536	67.9843	1.64E-06	0.000104
4210519	TM4SF11		10.24518	NM	01599	Homo sapiens	2.192958	-2.903172	2.706037	-1.714136	3.818079	0.624832	1.233966	-6.153029	1.741063	-4.602726	1.410949	-4.83728	61.93825	0.001314	0.014444
5390494	MAP3K5		17.06463	NM	00592	Homo sapiens	1.730991	-4.053701	3.701272	1.611874	3.81521	1.778173	2.138239	-2.212746	2.204061	-2.177757	1.030784	-5.656052	161.734	0.000136	0.002592
4480465	TOP1MT		13.25532	NM	05296	Homo sapiens	2.31797	-1.959278	3.178204	0.146243	3.814252	1.400272	1.371116	-5.590771	1.645514	-4.679381	1.200128	-5.373303	240.5557	0.000433	0.006226
290360	UBE2H		16.04403	NM	00334	Homo sapiens	3.193061	0.202962	4.333861	2.013687	3.811864	1.111252	1.357275	-5.694058	1.193796	-6.494015	-1.13694	-5.527206	291.4022	0.000181	0.003205
6290343	BCL10		16.1418	NM	00392	Homo sapiens	2.929614	0.183751	3.603979	1.454309	3.811845	1.805869	1.230189	-6.062683	1.301142	-6.06435	1.057677	-5.634156	265.0437	0.000176	0.003136
2570035	SETD3		13.80207	NM	19912	Homo sapiens	3.122799	-0.190804	3.823959	0.916884	3.796446	0.817212	1.224529	-6.160937	1.215719	-6.452512	-1.007247	-5.664618	70.52819	0.000361	0.00547
3840736	LOC643674		19.38452	XM	92864	PREDICTED:	2.724098	0.225564	3.887127	2.715587	3.792941	2.492259	1.426941	-5.089404	1.392366	-5.518927	-1.024832	-5.658151	1041.229	7.38E-05	0.001649
1500538	MLL3		40.40967	NM	02123	Homo sapiens	6.666553	6.829433	5.694061	5.913567	3.792452	3.126596	-1.170791	-6.165534	-1.757848	-3.321108	-1.50142	-3.879998	82.56931	1.73E-06	0.000108
6660692	BCL7B		39.74786	NM	00170	Homo sapiens	2.883801	4.512263	3.185106	5.315035	3.790436	6.779418	1.104482	-6.233925	1.314389	-4.906823	1.19005	-4.928483	384.8908	1.88E-06	0.000114
4570242	VAPA		20.03671	NM	00357	Homo sapiens	2.576053	0.319754	3.368277	2.301411	3.78852	3.163065	1.307534	-5.547475	1.470668	-4.910212	1.124765	-5.486616	66.74804	6.29E-05	0.001453
1980600	THOC3		36.71625	NM	03236	Homo sapiens	3.136676	3.751724	4.436429	6.34288	3.784588	5.116584	1.414373	-4.373445	1.20656	-6.068502	-1.172236	-5.201197	1282.341	2.87E-06	0.000153
3850692	FEZ1		13.83958	NM	02254	Homo sapiens	4.076616	0.571179	4.780973	1.251082	3.784544	-0.219297	1.17278	-6.338755	-1.077175	-6.746017	-1.263289	-5.330749	650.8627	0.000357	0.005424
130670		0	25.71134	CR738291		CR738291 Ho	2.208274	-0.330204	3.663332	3.925549	3.78011	4.130785	1.658912	-3.128064	1.711794	-3.030125	1.031877	-5.649351	275.2117	1.82E-05	0.000579
6770070	C6orf106		63.45165	NM	02429	Homo sapiens	1.442625	-2.081669	2.294036	4.744896	3.77875	9.792564	1.590182	-1.209618	2.619357	6.350117	1.647206	0.042293	37.99554	1.48E-07	2.19E-05
5690561	PSEN1		50.94238	NM	00731	Homo sapiens	5.95047	7.954204	5.102897	7.082773	3.774816	4.878325	-1.166096	-6.049193	-1.57636	-3.529243	-1.351827	-4.220072	99.59795	4.94E-07	4.74E-05
2490243	ARF4		39.57706	NM	00166	Homo sapiens	3.257855	4.723644	4.025687	6.338718	3.767756	5.79158	1.235686	-5.508969	1.156514	-6.28494	-1.068457	-5.569473	568.7995	1.93E-06	0.000116
460129	NALP7		30.96443	NM	20682	Homo sapiens	-1.065001	-6.609959	1.551924	-4.027206	3.766768	4.744819	1.6528	-2.836786	4.011611	5.350614	2.42716	0.721537	18.31958	7.06E-06	0.000287
1690050	SAMD3		45.1821	NM	00101	Homo sapiens	5.363898	6.706921	5.820362	7.218379	3.765534	4.183183	1.085099	-6.39571	-1.424472	-4.848952	-1.545694	-3.280627	566.2092	9.47E-07	7.34E-05
4050500	HYOU1		65.63884	NM	00638	Homo sapiens	2.603389	5.766799	3.596339	8.832988	3.765354	9.21455	1.381407	-2.982139	1.446328	-2.434102	1.046996	-5.578502	1079.679	1.23E-07	1.97E-05
3130152	LOC644685		27.6551	XM	93021	PREDICTED:	1.227101	-6.242042	5.230899	3.976578	3.763617	1.749544	4.262808	2.895279	3.067079	0.50044	-1.389859	-4.691263	37.97739	1.26E-05	0.000438
4610132	NEDD1		25.73006	NM	15290	Homo sapiens	5.89814	4.471647	5.24663	3.644045	3.763448	1.42529	-1.124177	-6.375373	-1.567217	-4.981531	-1.394102	-4.735212	191.3453	1.81E-05	0.000577
3940193	RNU22		20.8697	NR	00000	Homo sapiens	3.025524	1.75241	3.30955	2.246823	3.756283	3.187768	1.093877	-6.396191	1.241531	-6.121075	1.134983	-5.455058	142.3954	5.15E-05	0.001254
5290475	RPS15		55.72597	NM	00101	Homo sapiens	1.954967	2.039509	3.059156	7.033751	3.75331	8.844632	1.564812	-1.151294	1.919884	1.743491	1.22691	-4.259175	2202.573	3.02E-07	3.48E-05
4480064	MCOLN3		35.05959	NM	01829	Homo sapiens	1.945705	-0.265305	3.339563	5.044005	3.751838	5.987487	1.716377	-1.666132	1.928266	-0.439064	1.123452	-5.363004	81.03298	3.67E-06	0.000182
1070307	SOCS2		43.06177	NM	00387	Homo sapiens	2.451011	2.765717	3.963511	6.993618	3.749553	6.528398	1.617092	-2.091392	1.529798	-3.03805	-1.057062	-5.587322	1010.131	1.23E-06	8.67E-05
2760064	DNAJC3		10.44969	NM	00626	Homo sapiens	1.487429	-5.559307	2.754568	-1.856582	3.748262	0.174523	1.851899	-4.097316	2.51996	-2.226121	1.360744	-5.037571	65.42631	0.00121	0.013572
7150403	PSMB8		26.87244	NM	14891	Homo sapiens	1.377956	-4.71698	1.83702	-1.732961	3.748118	5.307926	1.333149	-4.893432	2.720057	2.689625	2.040325	-0.264884	26.05167	1.45E-05	0.000486
3180170	RRAS2		39.31414	NM	01225	Homo sapiens	2.094591	1.133603	3.356861	5.754418	3.745874	6.654362	1.602633	-2.131501	1.788356	-0.891305	1.115886	-5.360965	2497.694	2.00E-06	0.000119
7510148	C3orf59		36.94218	NM	17849	Homo sapiens	1.711903	-1.361539	2.811414	4.147856	3.739251	6.68471	1.642274	-1.783297	2.184266	1.60745	1.330025	-3.888771	1594.454	2.78E-06	0.00015
2650487	IL28RA		19.93205	NM	17074	Homo sapiens	1.894825	-3.425856	4.863966	3.268022	3.736514	1.461055	2.566974	-0.89012	1.971957	-3.191215	-1.301739	-5.04865	40.9644	6.45E-05	0.001482
4390386	NUT		35.4155	NM	17574	Homo sapiens	1.138962	-6.292702	2.409076	1.613786	3.735492	5.642474	2.115151	0.549306	3.279734	4.694968	1.550591	-2.658381	53.78299	3.48E-06	0.000175
5260682	OGDHL		74.8124	NM	01824	Homo sapiens	1.641751	0.669736	2.848081	7.895466	3.732715	10.39598	1.734783	1.652756	2.273619	5.389713	1.310607	-2.85473	487.2791	5.92E-08	1.22E-05
6250743	GCH1		36.88078	NM	00102	Homo sapiens	2.086604	1.226834	2.922192	4.646517	3.730493	6.792038	1.400454	-3.822336	1.78783	-0.774538	1.276608	-4.274553	239.2203	2.81E-06	0.000151
4540730	AP1S3		11.6286	NM	17881	Homo sapiens	1.163849	-6.505303	2.396453	-2.737969	3.729318	0.277299	2.059075	-3.317678	3.204296	-0.457419	1.556182	-4.414576	53.41387	0.000769	0.009603
3370088	SLC10A4		15.39324	NM	15267	Homo sapiens	2.278016	-1.941255	3.773242	1.593816	3.728918	1.461473	1.656372	-4.298456	1.636915	-4.632303	-1.011887	-5.663714	110.039	0.00022	0.003716
5360224	CTSL		14.50509	NM	00191	Homo sapiens	2.683604	-0.196648	2.728412	-0.354929	3.727734	2.006582	1.016697	-6.511221	1.389077	-5.617057	1.366265	-4.724455	56.73809	0.000289	0.004628
4850242	ITGB5		29.56574	NM	00221	Homo sapiens	1.480973	-4.011519	2.87767	2.864262	3.725275	5.053064	1.943095	-0.800189	2.515425	1.752371	1.294546	-4.511889	1911.753	8.91E-06	0.000342
630192	SLC25A21		31.05637	NM	03063	Homo sapiens	1.569734	-3.494982	3.44954	4.1798	3.72372	4.763133	2.197532	0.296089	2.372199	0.924394	1.079483	-5.558337	120.2818	6.91E-06	0.000284
3990647	CLK3		16.17405	NM	00399	Homo sapiens	2.380129	-1.907684	4.505798	2.338497	3.722698	1.01951	1.89309	-3.505671	1.564074	-5.104291	-1.210358	-5.360391	49.76751	0.000175	0.003118
1850553	SSSCA1		39.08899	NM	00639	Homo sapiens	1.428755	-3.589685	2.360927	2.669948	3.717107	7.040434	1.652437	-1.451306	2.601641	3.871657	1.574427	-1.856108	381.0839	2.06E-06	0.000122
4780593	RIOK1		50.75472	NM	03148	Homo sapiens	3.276724	5.674124	4.469688	8.097991	3.71619	6.65676	1.364072	-4.195126	1.134117	-6.333772	-1.202761	-4.837621	131.6091	5.04E-07	4.82E-05

1050376	MOCOS	27.63496	NM 01794	Homo sapiens	1.223555	-6.038987	3.170182	2.476128	3.713896	3.710106	2.59096	1.036439	3.035332	2.286335	1.171509	-5.300442	97.74124	1.26E-05	0.00044
4250682	CDC42SE2	23.85681	NM 02024	Homo sapiens	3.029978	1.674411	4.260459	4.046456	3.710894	2.995829	1.406102	-5.023442	1.224726	-6.209526	-1.148095	-5.421025	61.1964	2.66E-05	0.000763
5050066	OSTalpha	11.62534	NM 15267	Homo sapiens	1.153329	-6.50897	1.694729	-4.955044	3.708509	0.692494	1.469424	-5.31613	3.215484	-0.020799	2.188261	-2.268003	27.01646	0.00077	0.009607
6960437	KIAA0514	34.24574	XM 94189	PREDICTED:	1.543631	-2.918695	2.595374	3.026315	3.706461	6.280019	1.681343	-1.681912	2.401131	2.37516	1.428103	-3.241627	243.201	4.15E-06	0.000199
3930196	PPP2R1B	63.01264	NM 18169	Homo sapiens	2.173713	3.953493	3.242549	8.188117	3.701417	9.344071	1.49171	-1.543034	1.702809	0.452397	1.141515	-4.955861	622.5519	1.54E-07	2.25E-05
1990706	MT1X	33.54469	NM 00595	Homo sapiens	2.376974	2.288584	2.942557	4.250482	3.695127	6.230572	1.237942	-5.38285	1.55455	-2.921699	1.255754	-4.529106	9243.448	4.62E-06	0.000215
7330497	LOC647346	16.58854	XM 94283	PREDICTED:	2.940923	0.581446	3.36743	1.361664	3.694464	2.002685	1.145025	-6.299334	1.256226	-6.187681	1.097117	-5.573399	3813.022	0.000155	0.002854
1430594	CPEB1	24.3488	NM 03059	Homo sapiens	2.478487	-0.223647	4.746234	4.534235	3.692175	2.716789	1.914972	-2.421958	1.489689	-4.886483	-1.285485	-4.933711	102.0779	2.40E-05	0.000703
2070168	OSTF1	34.73107	NM 01238	Homo sapiens	1.848318	-0.778537	3.186223	4.77342	3.691607	6.003268	1.72385	-1.520199	1.99728	0.092246	1.158616	-5.177618	569.4231	3.85E-06	0.000189
990075	MDK	47.57497	NM 00239	Homo sapiens	4.114563	6.991032	3.999494	6.795813	3.690208	6.129583	-1.028771	-6.492905	-1.114995	-6.472214	-1.083813	-5.510709	336.2288	7.17E-07	6.14E-05
3850100	LOC649419	24.58054	XM 94156	PREDICTED:	2.786796	1.652903	3.707822	3.795202	3.679914	3.69605	1.330497	-5.269989	1.320482	-5.587653	-1.007584	-5.664164	2562.304	2.29E-05	0.000683
3120619	LOC649426	20.5437	XM 94157	PREDICTED:	2.625517	0.535963	3.544708	2.760166	3.679087	3.001876	1.350099	-5.311936	1.401281	-5.286403	1.03791	-5.646656	882.8579	5.57E-05	0.001326
4040315	UTP14A	31.61791	NM 00664	Homo sapiens	2.472816	1.794224	3.647803	5.106587	3.675914	5.135344	1.475161	-3.823669	1.486529	-3.976217	1.007706	-5.663845	485.3134	6.30E-06	0.000268
4730170	C14orf46	11.60665	NM 00102	Homo sapiens	2.286402	-2.561356	3.46813	0.090837	3.67536	0.431569	1.51685	-5.187015	1.607486	-5.094562	1.059753	-5.639162	60.60778	0.000775	0.009657
1710768	TAP1	34.24355	NM 00059	Homo sapiens	1.229708	-5.810956	3.005697	3.340852	3.665623	5.004272	2.444236	1.652894	2.980888	3.415543	1.219558	-4.940984	426.139	4.15E-06	0.000199
5900671	C1orf24	13.27687	NM 05296	Homo sapiens	1.412169	-5.673493	3.057093	-0.486316	3.663281	0.741507	2.16482	-2.603453	2.594081	-1.460159	1.198289	-5.40043	429.727	0.00043	0.00621
380360	CUTL1	44.86143	NM 18155	Homo sapiens	2.896213	4.918725	3.604701	6.789145	3.660227	6.894842	1.244626	-5.173299	1.263797	-5.256565	1.015404	-5.658441	137.8762	9.84E-07	7.51E-05
6550634	RARRES2	16.95911	NM 00288	Homo sapiens	1.906444	-2.946056	3.431897	1.638812	3.659499	2.070675	1.800156	-3.293788	1.919542	-2.968336	1.066319	-5.619557	1967.353	0.00014	0.002651
3460133	FLJ11806	15.31028	NM 02482	Homo sapiens	3.494108	0.366192	4.689827	1.989945	3.653958	0.334776	1.34221	-5.824664	1.045749	-6.768034	-1.283492	-3.212565	70.83413	0.000225	0.003793
380139	C1orf108	19.23289	XM 94112	PREDICTED:	2.136509	-1.830867	3.937693	2.83207	3.652649	2.237536	1.843051	-2.994149	1.709635	-3.88009	-1.078038	-5.600494	50.7667	7.67E-05	0.001693
130437	ARMC8	11.08525	NM 01415	Homo sapiens	2.247998	-2.756068	3.392192	-0.16404	3.651504	0.280654	1.508983	-5.245148	1.624336	-5.066447	1.076444	-5.624424	344.2929	0.000944	0.011199
5360477	FLJ36090	30.95745	NM 15322	Homo sapiens	4.865903	4.790533	5.2553	5.169555	3.643898	2.594702	1.080026	-6.433312	-1.335357	-5.718439	-1.442219	-4.228425	100.7188	7.03E-06	0.000287
4850471	PDPK1	49.37787	NM 00261	Homo sapiens	3.205123	5.929056	3.870797	7.485944	3.641319	6.967494	1.20769	-5.469652	1.136093	-6.280355	-1.063021	-5.55842	366.199	5.86E-07	5.34E-05
380347	LOC401238	15.5345	XR 00027	PREDICTED:	1.948013	-2.895868	3.305302	1.133033	3.640469	1.804245	1.696755	-3.894209	1.868811	-3.330237	1.101403	-5.567467	185.617	0.000211	0.003595
5260524	PINX1	71.6193	NM 01788	Homo sapiens	1.918699	3.305746	2.846877	8.086589	3.636971	10.37577	1.483754	-0.882171	1.89554	3.093909	1.27753	-3.182419	352.1798	7.55E-08	1.44E-05
6290634	DNMT3B	36.99955	NM 00689	Homo sapiens	2.002053	0.442199	3.336228	5.509365	3.636772	6.215991	1.666403	-2.660923	1.816521	-0.827404	1.090085	-5.481352	8977.16	2.76E-06	0.000149
4230594	BCL11A	48.05923	NM 02289	Homo sapiens	1.378992	-4.038461	3.218003	5.890172	3.633377	6.924152	2.333591	2.842998	2.634806	4.067827	1.129078	-5.258683	885.1465	6.78E-07	5.95E-05
1940398	DHRS4	25.55098	NM 02100	Homo sapiens	2.09462	-0.344087	3.152494	3.363899	3.633261	4.513512	1.505043	-3.824174	1.734568	-2.489651	1.152503	-5.310209	129.8444	1.88E-05	0.000593
7200064	RASGEF1A	25.39944	NM 14531	Homo sapiens	2.206451	-0.062142	3.438984	3.772309	3.632392	4.175759	1.558604	-3.6018	1.64626	-3.253428	1.05624	-5.614094	237.8764	1.94E-05	0.000605
5720221	MARVELD2	31.38859	NM 14472	Homo sapiens	1.957515	-0.44625	3.282066	4.559064	3.629369	5.374954	1.676649	-2.156269	1.85407	-1.153728	1.105819	-5.45167	1116.913	6.54E-06	0.000274
5690576	IGSF8	18.03849	NM 05286	Homo sapiens	1.646436	-4.30017	3.724862	1.995353	3.626511	1.752799	2.262379	-1.519315	2.202644	-1.946383	-1.02712	-5.657516	89.66564	0.000104	0.002132
4250332	LOC645367	18.73391	XM 93267	PREDICTED:	2.637707	0.167592	3.633408	2.468513	3.620671	2.396107	1.377488	-5.277725	1.372659	-5.560488	-1.003518	-5.664886	112.8306	8.70E-05	0.001864
2470044	ZNF101	14.08662	NM 03320	Homo sapiens	1.770614	-3.908883	3.185637	0.446786	3.620074	1.335618	1.799171	-3.631925	2.044531	-2.838037	1.136374	-5.509937	155.9721	0.00033	0.005103
7400296	XRCC6BP1	60.02614	NM 03327	Homo sapiens	1.938837	1.813486	3.63811	8.454877	3.619625	8.403467	1.876439	1.433727	1.866905	1.237456	-1.005107	-5.664051	646.6892	2.01E-07	2.66E-05
1400017	PCDH20	22.82024	NM 02284	Homo sapiens	3.162764	1.302445	4.960974	4.250779	3.617262	2.024733	1.568557	-4.411573	1.143703	-6.56358	-1.371472	-4.658864	79.87026	3.31E-05	0.000896
4830504	CD151	26.35526	NM 00435	Homo sapiens	2.084372	-0.431135	3.353484	3.847105	3.609336	4.41368	1.608871	-3.126248	1.731618	-2.536341	1.076294	-5.568203	373.3115	1.61E-05	0.000526
4200451	LOC133993	22.71202	XM 06863	PREDICTED:	3.946842	2.905922	4.462982	3.556043	3.60721	2.003922	1.130773	-6.33011	-1.094154	-6.684609	-1.237239	-5.182644	171.7295	3.39E-05	0.000912
4920176	CD24	22.23411	NM 01323	Homo sapiens	2.018846	-1.622742	3.621722	3.280184	3.602289	3.195603	1.793957	-2.646294	1.784331	-2.92122	-1.005395	-5.664621	2672.16	3.77E-05	0.000985
7040088	UGP2	41.67397	NM 00675	Homo sapiens	2.453721	2.657631	4.063972	7.049744	3.597933	6.050208	1.656249	-1.864022	1.466317	-3.657808	-1.12953	-5.310105	11107.75	1.46E-06	9.73E-05
4760204	ZNF286	15.76629	NM 02065	Homo sapiens	2.639348	-0.213429	3.268915	1.212192	3.597604	1.887528	1.238532	-5.982603	1.363066	-5.708147	1.10055	-5.565527	528.1008	0.000197	0.003402
1030368	DIAPH2	34.58056	NM 00730	Homo sapiens	2.055958	0.307442	3.57415	5.52824	3.595258	5.546178	1.738436	-1.567868	1.748702	-1.696602	1.005906	-5.664252	5399.31	3.94E-06	0.000191
2970114	GLS2	23.36196	NM 01326	Homo sapiens	1.013004	-6.686984	2.695138	0.317559	3.585536	2.606958	2.66054	0.572402	3.539508	2.721884	1.330372	-4.719462	228.7721	2.95E-05	0.00082
1500180	MCTP1	15.53008	NM 00100	Homo sapiens	1.279173	-6.009055	2.537196	-0.956975	3.576925	1.703842	1.983466	-2.585273	2.79628	0.068493	1.409795	-4.547053	39.11325	0.000211	0.003597
4480181	LOC650224	12.06354	XM 93931	PREDICTED:	3.315966	-1.108358	5.177375	1.205927	3.575893	-1.013279	1.561347	-5.377983	1.078387	-6.748979	-1.447855	-4.942742	96.51987	0.000656	0.008549
990139	HAS3	10.86396	NM 00532	Homo sapiens	1.465716	-5.518484	2.643895	-1.74349	3.575616	0.355057	1.803825	-4.042763	2.439501	-2.086693	1.352405	-4.992469	33.91022	0.001028	0.011956
6510209	DEGS1	75.37343	NM 00367	Homo sapiens	3.261075	7.457926	4.764417	10.52482	3.570191	8.305245	1.460996	-2.312121	1.094789	-6.445304	-1.334499	-3.224132	1383.398	5.67E-08	1.21E-05
1110600	EIF4E3	30.6942	NM 17335	Homo sapiens	1.823603	-1.205583	3.09839	4.153078	3.56727	5.340369	1.699048	-1.932515	1.956166	-0.441374	1.15133	-5.24637	234.4889	7.34E-06	0.000297
3870725	LIN7C	16.96094	NM 01836	Homo sapiens	2.95651	0.441263	3.855011	2.149277	3.565852	1.538788	1.303906	-5.758128	1.206102	-6.392501	-1.081091	-5.60275	154.6288	0.00014	0.002651
840608	TNRC5	17.22983	NM 00658	Homo sapiens	2.311726	-0.833608	3.176102	1.597958	3.565714	2.459622	1.373909	-5.254388	1.542447	-4.592478	1.12267	-5.50034	716.3877	0.00013	0.002513
3140646	LOC644733	11.15427	XM 92783	PREDICTED:	2.999137	-0.376435	2.656181	-1.572361	3.561939	0.493834	-1.129116	-6.381771	1.187654	-6.519278	1.341	-5.004433	63.39035	0.000919	0.010972
1430079	LOC644330	11.70878	XM 93436	PREDICTED:	2.151344	-2.800383	3.294439	0.036689	3.561448	0.531098	1.53134	-5.041069	1.655453	-4.789239	1.081048	-5.615038	137.3207	0.000746	0.009388
2260685	LOC388564	60.90445	XM 93805	PREDICTED:	1.157849	-5.756554	2.20644	3.811304	3.559341	8.818763	1.905637	2.092857	3.074097	7.48355	1.61316	-0.473634	491.3099	1.85E-07	2.51E-05
1580192	TMEM63B	22.46301	NM 01842	Homo sapiens	2.452598	0.810705	3.121713	2.758648	3.558514	3.80513	1.272819	-5.536698	1.450916	-4.64987	1.139923	-5.390879	722.6534	3.58E-05	0.000952
4210164	WDR43	40.64026	XM 94488	PREDICTED:	1.828455	-0.362889	3.378871	6.048528	3.557737	6.466244	1.847937	-0.122483	1.945761	0.369112	1.052937	-5.593012	507.3409	1.67E-06	0.000106
870224	SLCO3A1	25.90193	NM 01327	Homo sapiens	2.781129	1.614335	4.120821	4.565104	3.553112	3.39641	1.481708	-4.345478	1.277579	-5.840107	-1.159778	-5.341852	66.55401	1.75E-05	0.000563
7550139	LOC643025	28.42055	XM 92616	PREDICTED:	3.016146	2.747151	4.020029	4.874385	3.551293	3.873716	1.332836	-5.149678	1.177427	-6.305246	-1.13199	-5.41462	4775.578	1.09E-05	0.000395
2650471	APIP	56.50782	NM 01595	Homo sapiens	2.706631	5.429455	3.772802	8.432119	3.545808	7.900912	1.393911	-3.222892	1.310045	-4.394501	-1.064018	-5.529389	1822.827	2.80E-07	3.32E-05
4250093	ATP2A2	68.12786	NM 00168	Homo sapiens	3.202308	7.611417	3.86613	9.306902	3.539665	8.569044	1.207295	-5.099265	1.105348	-6.346513	-1.092231	-5.359569	1418.835	9.97E-08	1.77E-05
270372	CLSTN1	19.98049	XM 93795	PREDICTED:	3.005466	0.331858	5.088163	3.713133	3.534436	1.212823	1.69297	-4.078229	1.176003	-6.504827	-1.439597	-4.515185	44.86636	6.37E-05	0.001468
4260113	RB1	18.76984	NM 00032	Homo sapiens	4.928102	2.50983	5.161703	2.571265	3.528299	0.101159	1.047402	-6.496388	-1.396736	-5.900492	-1.462944	-4.67029	116.9415	8.62E-05	0.001853
770669	ARSE	15.36112	NM 00004	Homo sapiens	1.83131	-3.257003	3.064772	0.824452	3.528217	1.865627	1.673541	-3.874519	1.926608	-2.895724	1.151217	-5.446996	553.3264	0.000222	0.003746
630050	PAK2	33.34816	XM 93651	PREDICTED:	2.777676	2.437114	4.502845	6.166258	3.527192	4.272846	1.621084	-3.020026	1.269836	-5.71652	-1.276609	-4.680552	90.15702	4.77E-06	0.000219
3190333	CACYBP	18.91351	NM 01441	Homo sapiens	3.049698	1.472732	3.427859	2.155104	3.527101	2.328753	1.123999	-6.32963	1.156541	-6.498589	1.028952	-5.654958	1988.575	8.31E-05	0.001801
7000086	MRPL35	49.84659	NM 01662	Homo sapiens	2.450091	4.520193	3.140574	6.9709	3.520496	7.984216	1.281819	-4.414348	1.436884	-2.904082	1.120972	-5.208236	626.2026	5.56E-07	5.16E-05
7320056	MYC	40.87209	NM 00246	Homo sapiens	1.608587	-2.112471	3.295107	5.762396	3.515248	6.29788	2.048448	1.088378	2.185301	1.722121	1.066808	-5.553646	1784.44	1.62E-06	0.000104
5960497	UBE2I	13.68648	NM 19425	Homo sapiens	2.594361	-1.247001	3.838896	1.289319	3.515131	0.627342	1.479708	-5.197223	1.354912	-5.951934	-1.092106	-5.598463	74.23359	0.000375	0.005629
150475	LOC649270	21.60322	XM 94539	PREDICTED:	3.123223	1.471494	4.373596	3.722954	3.510793	2.088262	1.400347	-5.179343	1.124093	-6.605898	-1.245757	-5.12243	55.11285	4.35E-05	0.001105
5310037	MBTPS1	42.4565	NM 00379	Homo sapiens	3.20783	5.703791	3.374936	6.091109	3.510313	6.405245	1.052093	-6.436212	1.094295	-6.539702	1.040112	-5.622625	486.7439	1.32E-06	9.12E-05
4480091	SPTBN1	11.51503	NM 17831	Homo sapiens	1.673229	-5.052463	4.109455	0.350081	3.509107	-0.694122	2.456003	-2.510209	2.097207	-3.764792	-1.171083	-5.509462	31.72178	0.000802	0.009931
6620014	RBM4	33.00696	NM 00289	Homo sapiens	2.534128	2.359398	3.670779	5.528147	3.507005	5.119475	1.448537	-3.878321	1.38391	-4.641188	-1.046699	-5.620112	749.846	5.03E-06	0.000227
6450605	CWF19L1	61.63238	NM 01829	Homo sapiens	2.012933	3.489473	2.861964	7.581487	3.502136	9.485039	1.421788	-2.011903	1.739817	1.281495	1.223683	-3.998194	284.3454	1.74E-07	2.42E-05
6510541	SEC22L3	30.06362	NM 00420	Homo sapiens	2.118426	0.539619	3.206732	4.465987	3.499707	5.185753	1.513733	-3.324018	1.652031	-2.483605	1.091363	-5.499561	412.6961	8.18E-06	0.00032
2940255	PLA2G4C	17.96283	NM 00370	Homo sapiens	1.078188	-6.615511	2.290959	-1.459433	3.496847	2.008436	2.124824	-1.682143	3.243264	1.667014	1.526368	-3.915096	35.24274	0.000106	0.002163
6220632	MEST	15.88045	NM 17752	Homo sapiens	1.563948	-4.816898	3.522121	1.26157	3.493743	1.152396	2.25207	-1.7904	2.233924	-2.062717	-1.008123	-5.664394	58.13445	0.00019	0.003324
3180296	M-RIP	51.21618	NM 20127	Homo sapiens	2.901661	5.699389	3.655321	7.734074	3.487736	7.317964	1.259734	-4.54136	1.201979	-5.647836	-1.04805	-5.593012	858.4444	4.80E-07	4.68E-05
3390671	LIPT1	20.6134	NM 14519	Homo sapiens	1.375329	-5.026154	2.105123	-0.909062	3.486657	3.763064	1.530633	-3.824453	2.535144	1.1746	1.656272	-2.59831	45.93569	5.47E-05	0.001308
4200332	C9orf23	15.45745	NM 14817	Homo sapiens	1.803601	-3.051282	2.611054	0.036634	3.480685	2.374301	1.44769	-4.831287	1.929853	-2.499313	1.333057	-4.707891	131.1793	0.000215	0.003661

5820128	PHC1		51.66176	NM	00442	Homo sapiens	1.768576	0.259609	3.311444	7.307675	3.472537	7.712538	1.872378	1.143497	1.963465	1.660109	1.048647	-5.585207	2785.493	4.58E-07	4.51E-05
1470228	NAP1L2		32.30481	NM	02196	Homo sapiens	2.338522	1.849695	3.32712	5.065326	3.469873	5.394004	1.422745	-3.942572	1.483789	-3.66974	1.042906	-5.624238	167.9743	5.63E-06	0.000245
4570162	GSTO1		39.53305	NM	00483	Homo sapiens	1.946452	0.701294	3.180683	5.845613	3.464152	6.580169	1.634092	-1.518364	1.779726	-0.539365	1.089122	-5.457617	8524.398	1.94E-06	0.000117
4880500	ZSCAN2		19.00508	NM	00100	Homo sapiens	-1.595346	-4.971952	1.925662	-4.025438	3.462086	0.256387	3.072096	-0.138551	5.523223	3.445789	1.797868	-3.452624	82.27426	8.12E-05	0.001771
620070	U16		11.32041	NR	00244	Homo sapiens	2.364437	-1.632527	2.586968	-1.231774	3.461401	0.942522	1.094116	-6.429867	1.463943	-5.406128	1.338015	-4.918634	115.6883	0.000863	0.010475
3710711	ARID4B		21.33189	NM	01637	Homo sapiens	4.455705	3.041015	4.677113	3.161239	3.459205	1.038596	1.049691	-6.489189	-1.288072	-6.125066	-1.352077	-4.851734	81.10594	4.63E-05	0.001156
2350196	HPSE		27.81532	NM	00666	Homo sapiens	1.388557	-4.887846	3.247643	3.376904	3.458785	3.862625	2.338862	0.677608	2.49092	1.149093	1.065014	-5.596531	32.49758	1.22E-05	0.000429
290592	RNF8		24.82823	NM	00395	Homo sapiens	2.656348	1.941772	3.23005	3.480001	3.45716	4.011346	1.215974	-5.798508	1.301471	-5.528848	1.070312	-5.58301	578.1152	2.17E-05	0.000658
5220014		0	40.3524	BX417162	BX417162 Ho		2.305855	1.239705	4.829264	7.38599	3.456347	4.814506	2.094349	0.297926	1.498944	-3.79727	-1.397216	-3.750282	69.94904	1.74E-06	0.000108
2060228	DTYMK		33.30283	NM	01214	Homo sapiens	2.724632	3.367079	3.393531	5.211687	3.454205	5.330836	1.245501	-5.389732	1.267769	-5.476991	1.017879	-5.657785	3081.1	4.80E-06	0.00022
1710471	SPTBN1		11.2563	NM	00312	Homo sapiens	1.758426	-3.836423	2.170708	-2.423609	3.452553	1.249636	1.234461	-6.032257	1.963434	-3.010502	1.590519	-3.848921	932.222	0.000884	0.010666
1400035	INOC1		15.31234	NM	01755	Homo sapiens	3.185286	0.937476	3.330573	1.009632	3.449648	1.221126	1.045612	-6.492107	1.082995	-6.713257	1.035752	-5.652529	128.4841	0.000225	0.003792
3400343		0	61.09366	AK094462	Homo sapiens		1.368552	-3.338261	3.007346	7.089951	3.444742	8.336556	2.197465	3.751601	2.51707	5.302787	1.145442	-4.96891	1365.794	1.82E-07	2.49E-05
5390747	LOC402562		9.852334	XM	93995	PREDICTED:	2.662267	-2.777075	4.867784	0.396022	3.440669	-1.644576	1.828436	-4.737989	1.292383	-6.420031	-1.414779	-5.085227	126.2338	0.001544	0.016493
4250224	ECOP		28.18948	NM	03079	Homo sapiens	2.286361	1.626484	2.741334	3.288959	3.434853	5.329097	1.198994	-5.717447	1.502323	-3.504861	1.252986	-4.601627	1087.809	1.14E-05	0.000406
4230379		0	12.32281	BE387864	601282486F1		1.827595	-3.477764	2.613438	-0.803476	3.433936	1.291213	1.429988	-5.195135	1.878938	-3.330578	1.313953	-4.945505	76.08924	0.000598	0.007953
5360609	STAT3		20.53745	NM	21366	Homo sapiens	2.097398	-1.077349	3.289006	2.747723	3.433354	3.053508	1.568136	-3.836243	1.636958	-3.652159	1.043888	-5.637464	250.2064	5.57E-05	0.001326
5420239	C14orf29		14.40067	NM	18153	Homo sapiens	1.641893	-3.799412	1.707303	-3.786121	3.428286	2.500299	1.039839	-6.491771	2.088008	-1.587266	2.008012	-1.564485	23.99836	0.000298	0.004738
6270546	C10orf61		43.87439	NM	00101	Homo sapiens	3.920136	6.55505	3.93853	6.582342	3.426037	5.411835	1.004692	-6.513874	-1.144219	-6.319948	-1.149587	-5.223102	404.4421	1.11E-06	8.14E-05
2480048	GNG10		26.23728	NM	00101	Homo sapiens	2.975084	2.900811	3.422289	3.905661	3.424348	3.870854	1.150317	-6.141204	1.151009	-6.405102	1.000602	-5.665028	1746.92	1.64E-05	0.000533
5670441	GTF3C4		15.1236	NM	01220	Homo sapiens	2.021048	-2.294145	3.08708	1.028161	3.416788	1.775225	1.527465	-4.555417	1.690601	-3.956337	1.106802	-5.546905	95.29645	0.000238	0.003969
4880142	RNU31		31.00805	NR	00256	Homo sapiens	3.093483	3.488362	3.962008	5.345576	3.410989	4.111785	1.280759	-5.362503	1.102637	-6.588726	-1.161542	-5.26296	666.9549	6.97E-06	0.000285
6060333	ERGIC1		29.69514	NM	00103	Homo sapiens	1.307004	-5.391224	3.255434	3.585562	3.409749	3.936009	2.49076	1.438492	2.608828	1.76003	1.047402	-5.626471	75.7696	8.72E-06	0.000336
5960678	LOC652771		16.89034	XM	94241	PREDICTED:	3.010739	0.254761	4.352988	2.603123	3.3999	0.829941	1.445821	-5.223926	1.129258	-6.629351	-1.280328	-5.114298	96.77523	0.000142	0.002686
6510112	KLF4		14.1665	NM	00423	Homo sapiens	1.528145	-4.823495	2.770661	-0.143493	3.398282	1.417182	1.813088	-3.285879	2.223795	-1.756979	1.226524	-5.231947	57.57787	0.000321	0.005012
1990131	CACYBP		10.75702	NM	00100	Homo sapiens	2.06671	-3.562848	3.827994	0.286283	3.395142	-0.552781	1.852216	-4.131826	1.642776	-5.120521	-1.127492	-5.566379	42.69147	0.001072	0.012346
2600315	TERF1		40.55525	NM	01748	Homo sapiens	1.826863	-0.342913	3.503727	6.403978	3.393879	6.102512	1.917893	0.375342	1.857764	-0.190494	-1.032367	-5.637228	10211.02	1.69E-06	0.000107
5550402	SLC2A14		32.95751	NM	15344	Homo sapiens	3.474461	4.60362	3.865993	5.33479	3.391652	4.238607	1.112688	-6.275028	-1.024416	-6.77247	-1.139855	-5.344574	1249.461	5.07E-06	0.000228
3800189	ZNF679		31.7196	NM	15336	Homo sapiens	3.524445	3.835612	4.682831	5.781923	3.391582	3.319766	1.328672	-5.21811	-1.039175	-6.758444	-1.380722	-4.219734	336.3464	6.19E-06	0.000264
730736	FLJ20245		17.47428	NM	01772	Homo sapiens	1.274613	-5.978508	2.933802	0.559937	3.387861	1.650106	2.30172	-1.073417	2.657953	-0.035938	1.154768	-5.433126	110.3448	0.000121	0.002385
2710022	SLC37A1		78.8346	NM	01896	Homo sapiens	-1.110963	-6.160946	2.374858	5.041637	3.387625	8.718347	2.63838	6.190713	3.763527	9.477825	1.426454	-1.947501	191.4937	4.41E-08	1.05E-05
7650747	KLHL4		44.65223	NM	05716	Homo sapiens	5.945062	7.14695	5.536765	6.743511	3.380432	3.164654	-1.073743	-6.426848	-1.758669	-2.790836	-1.637887	-2.844484	200.444	1.01E-06	7.64E-05
4760424	NCL		30.36678	NM	00538	Homo sapiens	2.355008	2.203703	2.880379	4.061641	3.380293	5.49389	1.223087	-5.494568	1.435363	-3.93997	1.173558	-5.069735	2618.463	7.76E-06	0.000309
2710424	PLEKHF1		30.55198	NM	02431	Homo sapiens	-1.05214	-6.631991	1.982733	-0.892194	3.3795	4.387032	2.086113	0.073904	3.555708	4.948768	1.704465	-1.923386	335.717	7.52E-06	0.000302
2570286	KBTBD8		32.86107	NM	03250	Homo sapiens	1.85633	-0.314888	2.923011	4.523366	3.371004	5.801221	1.574618	-2.341704	1.815951	-0.664335	1.153264	-5.157514	1326.344	5.15E-06	0.000231
2650047	TMEM37		15.40649	NM	18324	Homo sapiens	1.96711	-2.462009	3.104958	1.183828	3.367982	1.777794	1.578436	-4.246475	1.712148	-3.789679	1.084711	-5.587021	50.37195	0.000219	0.003707
2940725	NUDT15		56.21704	NM	01828	Homo sapiens	2.040817	3.237151	3.036934	7.678658	3.367618	8.634999	1.488097	-1.515335	1.650133	0.031091	1.108888	-5.211953	2043.693	2.88E-07	3.37E-05
1230068	TNFAIP8L3		9.678786	NM	20738	Homo sapiens	1.428234	-5.538325	1.673043	-4.859547	3.367201	0.464443	1.171407	-6.271757	2.357598	-1.942495	2.01262	-2.557408	31.11027	0.001661	0.01742
6380152	C7orf28A		48.84183	NM	01562	Homo sapiens	3.551374	6.389061	4.234972	7.766369	3.36456	5.875998	1.192488	-5.658849	-1.055524	-6.698321	-1.2587	-4.420296	959.3503	6.21E-07	5.58E-05
6180370	RP2		46.4073	NM	00691	Homo sapiens	5.327173	7.072938	5.435124	7.229699	3.362692	3.68684	1.020264	-6.506609	-1.584199	-3.596916	-1.616302	-2.720597	124.6904	8.20E-07	6.62E-05
4570743	FLJ32810		41.47719	XM	93745	PREDICTED:	2.948845	5.704053	2.744765	4.919198	3.362078	6.836972	-1.074353	-6.334649	1.140134	-6.1978	1.224906	-4.496995	104.2765	1.50E-06	9.93E-05
2690112	ASPSCR1		13.49338	XM	94136	PREDICTED:	1.978139	-2.438597	2.625432	-0.250176	3.35953	1.716751	1.327224	-5.547527	1.698329	-3.881559	1.27961	-4.995345	551.6461	0.0004	0.005894
2120598	MYBBP1A		25.86464	NM	01452	Homo sapiens	1.663685	-2.480727	2.772684	2.855138	3.357869	4.557449	1.666592	-2.32349	2.018332	-0.285206	1.211053	-4.952667	516.7245	1.77E-05	0.000567
3120056	LOC652481		27.77497	XM	94194	PREDICTED:	2.599078	2.44738	3.165504	4.091685	3.353527	4.557855	1.217934	-5.673102	1.290276	-5.424098	1.059397	-5.595852	640.2745	1.23E-05	0.000432
4920719	TRIM22		21.38629	NM	00607	Homo sapiens	2.352234	0.166221	3.411428	3.209133	3.351972	3.022306	1.450293	-4.509178	1.425016	-4.919878	-1.017738	-5.660249	685.9412	4.57E-05	0.001149
6960066	TSPAN3		53.93877	NM	00572	Homo sapiens	3.871174	7.68792	3.778493	7.570638	3.351787	6.530901	-1.024528	-6.494701	-1.154958	-6.116522	-1.127307	-5.246698	1573.755	3.62E-07	3.93E-05
1010386	C7orf28B		50.24299	NM	19809	Homo sapiens	3.215358	5.860698	4.296504	8.18496	3.34785	6.13967	1.336245	-4.322834	1.041206	-6.733601	-1.283362	-4.157961	1063.438	5.33E-07	5.00E-05
6380239	LOC440349		28.93009	XM	49612	PREDICTED:	4.574875	4.649949	4.666976	4.661366	3.342556	2.171455	1.020132	-6.508693	-1.368676	-5.488224	-1.39623	-4.387437	82.08568	9.97E-06	0.000372
7330082	HLA-F		24.58567	NM	01895	Homo sapiens	1.500882	-4.038161	2.970306	2.765488	3.34019	3.745895	1.979041	-0.890403	2.225485	0.168391	1.124527	-5.42317	43.29829	2.28E-05	0.000683
2230139	C9orf65		16.26174	NM	13881	Homo sapiens	1.107345	-6.546415	1.960644	-2.684418	3.339907	1.934345	1.770581	-3.140556	3.016139	1.360523	1.703474	-3.007916	39.69423	0.00017	0.003056
3370072	MGST1		26.85846	NM	02030	Homo sapiens	2.369489	1.339509	3.289573	4.215274	3.339571	4.306909	1.388305	-4.493287	1.409406	-4.580348	1.015199	-5.660469	1274.381	1.46E-05	0.000487
7050327	DLG3		29.0784	NM	02112	Homo sapiens	2.136464	1.058715	2.850762	3.884306	3.338987	5.306078	1.334336	-4.604506	1.562857	-2.887406	1.171261	-5.091684	182.0488	9.71E-06	0.000364
1050750	LTBR		20.3509	NM	00234	Homo sapiens	2.118599	-1.118499	3.50392	3.069513	3.338228	2.636864	1.653886	-3.410843	1.575677	-4.113356	-1.049635	-5.631302	54.50546	5.83E-05	0.001372
6590270	C14orf124		11.76529	NM	02019	Homo sapiens	2.873645	-1.286854	4.206182	0.911002	3.336973	-0.642465	1.46371	-5.485279	1.161233	-6.613282	-1.260478	-5.305077	137.8695	0.000731	0.009247
6220392	TMPRSS2		13.31264	NM	00565	Homo sapiens	2.541536	-0.995279	3.374648	0.887572	3.336211	0.751524	1.327798	-5.708505	1.312675	-6.031887	-1.011521	-5.663767	345.3605	0.000425	0.006156
4180528	NME1		57.27747	NM	19817	Homo sapiens	1.55358	-0.583371	2.528917	6.202116	3.334973	9.012422	1.6278	0.31163	2.146638	4.291541	1.318736	-2.915224	772.3424	2.60E-07	3.20E-05
1690348	MGC40168		23.10714	NM	15370	Homo sapiens	1.421872	-4.868757	3.219917	2.675231	3.332138	2.914668	2.264561	-0.142953	2.343486	0.019105	1.034852	-5.647103	65.42205	3.11E-05	0.000855
1710259	HERC5		41.62955	NM	01632	Homo sapiens	1.446518	-2.993836	2.596074	4.534383	3.329659	6.962907	1.794705	0.279483	2.301844	3.319882	1.282575	-3.94287	2173.851	1.47E-06	9.77E-05
1090274	FAM53B		9.399077	NM	01466	Homo sapiens	1.401014	-5.670718	1.809397	-4.32494	3.326284	0.279287	1.291492	-5.91208	2.374198	-1.96225	1.838338	-3.184261	85.87901	0.001871	0.019145
3800300	ZFP64		36.51154	NM	19942	Homo sapiens	2.357723	-0.043959	6.398748	7.229093	3.324488	2.671635	2.713952	1.307448	1.410042	-5.101665	-1.924732	-1.669878	36.00389	2.96E-06	0.000157
1940458	FLVCR		75.90613	NM	01405	Homo sapiens	1.563789	0.291043	2.885023	8.655264	3.324313	10.02312	1.844892	3.122281	2.125807	5.112929	1.152266	-4.61091	2030.775	5.45E-08	1.20E-05
4260274	LOC440525		27.42888	XM	49871	PREDICTED:	2.557533	1.86451	3.574788	4.643445	3.322628	3.996142	1.397749	-4.51213	1.299153	-5.47257	-1.075892	-5.564078	168.5404	1.31E-05	0.000452
430315	MRS2L		49.97454	NM	02066	Homo sapiens	2.167494	4.084599	2.546861	5.905231	3.317746	8.576456	1.175026	-5.336916	1.530683	-1.177736	1.30268	-3.236243	387.2928	5.48E-07	5.11E-05
730392	HNMT		12.0346	NM	00102	Homo sapiens	3.499654	-0.474863	4.707758	1.030066	3.316993	-1.172964	1.345207	-5.942451	-1.055068	-6.765293	-1.419285	-4.962861	87.28235	0.000663	0.00862
1010068	PHLDA1		25.27241	NM	00735	Homo sapiens	2.267498	0.166878	3.775116	4.458965	3.313472	3.365669	1.664881	-2.936461	1.46129	-4.526195	-1.139323	-5.385253	3271.723	1.99E-05	0.000617
5690504	RBPMS		25.44315	NM	00100	Homo sapiens	2.910621	2.067982	4.098745	4.595636	3.311018	2.891924	1.408203	-4.783624	1.137564	-6.505121	-1.237911	-5.008578	379.8428	1.92E-05	0.0006
1070719	ABI2		23.80109	XM	93845	PREDICTED:	3.96478	3.558481	4.058697	3.55338	3.310856	1.967299	1.023688	-6.506718	-1.197509	-6.338493	-1.225876	-5.16295	163.9611	2.69E-05	0.00077
3190328	ALDH3A2		38.23641	NM	00103	Homo sapiens	1.744008	-0.059548	2.458493	4.138113	3.31041	7.129839	1.40968	-3.129422	1.898162	1.072709	1.34652	-3.284844	4356.654	2.32E-06	0.000133
5080494	MAT2B		69.63246	NM	01328	Homo sapiens	4.243791	9.01214	4.252622	9.230991	3.309669	7.154816	1.002081	-6.514285	-1.28224	-4.748868	-1.284908	-3.854173	74.199	8.83E-08	1.63E-05
1740152	VAC14		18.48381	NM	01805	Homo sapiens	1.623803	-3.850362	3.087802	1.807117	3.309262	2.328978	1.901586	-2.198828	2.03797	-1.740217	1.071721	-5.599551	85.23908	9.28E-05	0.001956
5890202	SYPL1		51.90475	NM	18271	Homo sapiens	1.574417	-1.205101	3.042133	6.933368	3.30861	7.702324	1.932228	1.901344	2.101483	2.913284	1.087595	-5.400446	108.0698	4.46E-07	4.44E-05
4610487	FLJ22746		27.1325	NM	02478	Homo sapiens	-1.119518	-6.486566	2.676237	0.807735	3.308016	2.58539	2.996095	2.090692	3.703383	3.666769	1.23607	-5.05308	69.14997	1.39E-05	0.000469
6040133	COG8		55.26833	NM	03238	Homo sapiens	2.141565	3.927624	3.000831	7.632198	3.305983	8.538629	1.401232	-2.508507	1.543722	-1.035474	1.101689	-5.260059	210.2449	3.16E-07	3.61E-05
5820603	PTGFRN		36.69126	NM	02044	Homo sapiens	3.225394	5.33909	3.202155	5.187982	3.304164	5.435922	-1.007257	-6.512982	1.024422	-6.768539	1.031856	-5.640169	170.0039	2.88E-06	0.000153
5690102	PLTP		16.53379	NM	00622	Homo sapiens	1.839685	-3.575366	4.135199	2.374725	3.302171	0.721013	2.247775	-1.816207	1.794965	-3.857447	-1.252267	-5.193803	107.8245	0.000158	0.002885
4850209	DHRS4L2		13.82288	NM	19808	Homo sapiens	1.969907	-2.185515	2.419497	-0.591487	3.297271	2.011762	1.228229	-5.936217	1.673821	-3.793266	1.362792	-4.557955	144.774	0.000359	0.005442
5860056	LOC651745		13.34249	XM	94490	PREDICTED:	2.188122	-2.490133	3.84125	1.379277	3.296939	0.250675	1.755501	-4.015622	1.506744	-5.317865	-1.165096	-5.463753	223.4822	0.000421	0.006116

6960750	LOC642953		9.816576	XM 92633	PREDICTED:	1.701511	-4.439178	2.581382	-1.613353	3.295984	0.154913	1.517112	-5.047416	1.937092	-3.570013	1.276829	-5.175694	44.0884	0.001567	0.016657
510746	IPO7		58.84021	NM 00639	Homo sapiens	2.849357	6.381959	3.687763	8.734236	3.295899	7.745332	1.294244	-4.153257	1.156717	-5.920643	-1.118894	-5.194493	615.3555	2.24E-07	2.86E-05
460102	RPLP0		19.30412	NM 05327	Homo sapiens	2.934553	1.489297	3.453044	2.568895	3.292541	2.144064	1.176685	-6.135018	1.121991	-6.590388	-1.048747	-5.634919	3514.869	7.53E-05	0.001674
3440224	RPL32		16.77239	NM 00099	Homo sapiens	2.379317	0.131372	2.552264	0.510626	3.288532	2.691245	1.072688	-6.433344	1.382133	-5.240468	1.288476	-4.786357	158.7132	0.000147	0.002748
2120521	LOC653383		22.58919	XM 92717	PREDICTED:	2.589618	1.332903	3.384348	3.452462	3.286582	3.166334	1.306891	-5.328434	1.269138	-5.816855	-1.029747	-5.650899	234.2242	3.49E-05	0.000932
5810681	ICAM3		33.57329	NM 00216	Homo sapiens	-1.066215	-6.584678	1.887226	-0.927632	3.285	4.874318	2.01219	0.233121	3.502518	5.537641	1.74065	-1.348465	941.6696	4.60E-06	0.000214
5560671	PCBP3		14.36618	NM 02052	Homo sapiens	1.85801	-2.766965	2.626625	0.099812	3.282623	1.924197	1.413676	-5.013647	1.766741	-3.319664	1.249749	-5.0661	54.33282	0.000301	0.004776
7160435	LOC147804		33.25395	NM 00101	Homo sapiens	2.406172	2.506095	3.303452	5.408415	3.281389	5.317187	1.372908	-4.214867	1.363738	-4.541631	-1.006724	-5.663909	525.2562	4.84E-06	0.000221
2230484	SLC39A8		20.67934	NM 02215	Homo sapiens	3.134083	2.607113	2.979848	1.963058	3.279691	2.729815	-1.051759	-6.471843	1.04646	-6.750186	1.100623	-5.52775	928.5429	5.39E-05	0.001294
2760168	KCNK6		21.1244	NM 00482	Homo sapiens	1.361589	-5.123088	2.559142	1.043104	3.277942	3.229724	1.879526	-1.681368	2.407438	0.659266	1.280875	-4.731126	413.186	4.85E-05	0.001199
450707	MYOHD1		14.38433	NM 00103	Homo sapiens	1.49448	-4.694123	2.260348	-1.181668	3.276939	2.00875	1.512464	-4.433039	2.192695	-1.224187	1.44975	-4.128098	183.0834	0.0003	0.004756
460639	WNT8A		30.20111	NM 05824	Homo sapiens	5.860071	5.427157	4.992883	4.358284	3.27544	1.330256	-1.173685	-6.200715	-1.789094	-3.52521	-1.52434	-3.977714	59.65597	7.99E-06	0.000315
2320358	C5orf16		31.83746	NM 17382	Homo sapiens	1.357234	-4.733153	3.020413	3.944806	3.269597	4.620302	2.225419	1.175999	2.409015	1.890662	1.0825	-5.528085	297.0811	6.07E-06	0.00026
6620154	LOC648256		15.25327	XM 94358	PREDICTED:	2.513738	-0.62718	3.508579	1.734449	3.268332	1.146211	1.395761	-5.298192	1.300188	-5.995488	-1.073508	-5.610423	457.0427	0.000229	0.003837
1240348	MRGPRF		11.19432	NM 14501	Homo sapiens	1.442398	-5.280791	2.021881	-2.920619	3.266781	0.992381	1.401749	-5.306401	2.264826	-1.694229	1.615714	-3.689948	453.197	0.000905	0.010853
6200121	AGPAT2		29.80573	NM 00641	Homo sapiens	1.625422	-2.133784	2.647389	3.410013	3.255873	5.360437	1.628739	-1.974989	2.003094	0.466595	1.229843	-4.673879	379.786	8.55E-06	0.000332
4780070	CLASP2		49.94739	NM 01509	Homo sapiens	3.537542	6.634581	4.125253	7.866748	3.244547	5.846621	1.166136	-5.816101	-1.090304	-6.553173	-1.271442	-4.256755	543.7761	5.50E-07	5.11E-05
7160255	STAMBPL1		26.30492	NM 02079	Homo sapiens	1.638034	-2.734938	3.026069	3.557193	3.24443	4.138436	1.847378	-1.201201	1.980685	-0.591624	1.07216	-5.566991	624.7631	1.62E-05	0.000528
3990520	MAP3K3		13.58126	NM 20335	Homo sapiens	2.202701	-1.697716	3.065714	0.756333	3.243806	1.148819	1.391798	-5.302375	1.472649	-5.161671	1.058091	-5.629893	158.1371	0.000388	0.005767
1400719	MRPL11		24.77666	NM 17073	Homo sapiens	1.197278	-5.967548	1.970445	-0.74055	3.241009	4.318312	1.645772	-2.432771	2.706982	2.852249	1.64481	-2.126888	176.7048	2.20E-05	0.000661
380386	TRIM6		50.41219	NM 00100	Homo sapiens	1.63694	-0.59351	3.081181	7.053995	3.238379	7.503428	1.882281	1.545424	1.978313	2.114288	1.051019	-5.570558	1041.223	5.23E-07	4.93E-05
240608	MME		11.54728	NM 00728	Homo sapiens	1.698258	-4.670734	3.647637	0.388845	3.236306	-0.483508	2.147869	-2.899048	1.905662	-3.977999	-1.127099	-5.557066	80.41138	0.000792	0.009842
580707	CRB3		10.32862	NM 13916	Homo sapiens	-1.087771	-6.630732	1.325775	-6.443439	3.23464	-0.577232	1.44214	-5.495664	3.518548	0.271755	2.43981	-1.814382	32.3817	0.00127	0.014092
4830474		0	18.80082	BU597818	AGENCOURT	1.519874	-4.549834	3.287359	2.081683	3.231017	1.894873	2.162916	-1.158444	2.125845	-1.513272	-1.017438	-5.661131	29.64466	8.55E-05	0.001842
4260195	SPG21		41.48933	XM 94560	PREDICTED:	3.290102	6.023122	3.323265	6.067381	3.229496	5.782172	1.01008	-6.511316	-1.018766	-6.774163	-1.029035	-5.642051	399.3525	1.50E-06	9.92E-05
3370521	PDIA6		30.13525	NM 00574	Homo sapiens	2.732547	3.454146	3.08556	4.433411	3.229433	4.809108	1.129188	-6.143024	1.18184	-6.094043	1.046628	-5.616687	2174.46	8.08E-06	0.000317
7330435	MTMR3		9.767928	NM 02109	Homo sapiens	1.464607	-5.288174	1.495983	-5.520597	3.228908	0.512402	1.021423	-6.509634	2.204624	-2.211027	2.158386	-1.904798	39.44803	0.001599	0.016926
4040202	DAZAP1		16.27514	NM 17071	Homo sapiens	1.476662	-4.971141	3.127935	1.160286	3.228113	1.36048	2.118248	-1.728463	2.186088	-1.652317	1.032027	-5.653444	26.09665	0.00017	0.003048
1070050	GNPTAB		27.66997	NM 02431	Homo sapiens	2.086253	0.489245	3.073484	4.234315	3.216547	4.607727	1.473208	-3.579038	1.541782	-3.255159	1.046548	-5.618348	812.7071	1.25E-05	0.000438
3190338	TMEM67		27.76193	NM 15370	Homo sapiens	5.690204	4.924176	5.030146	4.057022	3.216304	0.878822	-1.13122	-6.338734	-1.769174	-3.787511	-1.563952	-3.905067	57.88038	1.23E-05	0.000433
5870739	C20orf127		14.12135	NM 08075	Homo sapiens	1.723314	-3.226033	2.222921	-1.061713	3.213226	2.197108	1.289911	-5.579774	1.864562	-2.53533	1.445497	-4.0518	668.1268	0.000326	0.005069
1440504	RNF187		15.55133	XM 04749	PREDICTED:	2.642732	0.067453	3.292958	1.579632	3.210936	1.331898	1.246043	-5.916203	1.215006	-6.306732	-1.025545	-5.657589	192.5075	0.00021	0.003581
5870364	SURF5		11.95644	NM 18149	Homo sapiens	1.481747	-5.092171	2.464127	-1.22748	3.20817	0.852059	1.662988	-4.055742	2.165127	-2.077221	1.30195	-4.979191	279.8909	0.000682	0.00881
430240	EDG7		11.19963	NM 01215	Homo sapiens	1.585985	-4.622839	2.427737	-1.432858	3.206989	0.753175	1.530743	-4.726546	2.02208	-2.724439	1.320979	-4.922245	32.30597	0.000903	0.010839
2120768	IRXL1		10.95987	NM 17357	Homo sapiens	1.958624	-3.370136	3.165744	-0.031307	3.206437	0.008347	1.61631	-4.615202	1.637087	-4.77704	1.012854	-5.663613	30.4121	0.00099	0.011611
4200719	TSC22D1		16.91467	NM 00602	Homo sapiens	6.006085	2.548743	5.281186	1.628924	3.20116	-1.449328	-1.137261	-6.404083	-1.876221	-4.549173	-1.649773	-4.337848	531.4655	0.000142	0.002674
1580484	TUBB		28.65393	NM 17801	Homo sapiens	2.761342	3.318431	3.096916	4.214206	3.195321	4.458171	1.121526	-6.197934	1.157162	-6.275395	1.031775	-5.643293	8097.385	1.05E-05	0.000383
1300114	LIG3		48.97053	NM 00231	Homo sapiens	2.190357	4.029785	2.801811	6.737849	3.194652	8.017448	1.279157	-4.133911	1.458508	-2.144731	1.14021	-4.969736	198.3415	6.12E-07	5.53E-05
6350605	PLEKHA3		18.12403	NM 01909	Homo sapiens	2.99331	0.824398	4.108613	2.927867	3.194235	1.018575	1.372598	-5.395771	1.067125	-6.733121	-1.286259	-5.010378	131.3696	0.000102	0.002097
1400717		0	13.26371	BM475722	AGENCOURT	3.582134	0.015366	4.775179	1.511868	3.193524	-1.085266	1.333054	-5.928732	-1.121687	-6.686832	-1.495269	-4.682878	84.97139	0.000432	0.00622
6380706	LOC85390		11.96082	NR 00145	Homo sapiens	1.358001	-5.82025	2.865079	-0.676661	3.191489	0.076452	2.109776	-2.590343	2.350138	-1.964808	1.113927	-5.56219	68.98512	0.000681	0.008801
430609	TPST2		59.92815	NM 00100	Homo sapiens	2.06858	3.934564	3.077594	8.389781	3.186641	8.714654	1.487781	-1.146991	1.540497	-0.713867	1.035432	-5.605461	3158.579	2.03E-07	2.67E-05
3310446	C12orf35		27.18706	NM 01816	Homo sapiens	2.648444	2.411747	3.422742	4.525794	3.184454	3.866438	1.29236	-5.207959	1.202387	-6.068644	-1.074829	-5.562114	943.8012	1.37E-05	0.000465
160082	LOC645436		27.01759	XM 92847	PREDICTED:	3.569415	3.774747	4.01398	4.510644	3.180852	2.622357	1.124548	-6.280518	-1.122157	-6.557242	-1.26192	-4.884614	15207.43	1.42E-05	0.000476
1990672	TSNAX		35.93738	NM 00599	Homo sapiens	3.187628	4.927143	3.578537	5.817024	3.180197	4.762051	1.122633	-6.171069	-1.002337	-6.784887	-1.125256	-5.344031	1148.97	3.22E-06	0.000166
6580753	LIN7A		17.1762	NM 00466	Homo sapiens	1.698085	-3.38255	2.982769	1.596206	3.180094	2.086273	1.756549	-2.910897	1.872753	-2.508311	1.066155	-5.608002	171.497	0.000132	0.002538
3290273	PRIM2A		17.56301	XM 94268	PREDICTED:	2.470531	0.394848	2.97235	1.7842	3.177703	2.304392	1.203122	-5.982864	1.286243	-5.824431	1.069088	-5.600098	178.4336	0.000118	0.002345
1500519	PNRC2		24.95236	NM 01776	Homo sapiens	2.921294	2.281674	3.901082	4.432964	3.17654	2.740601	1.335395	-5.183837	1.087374	-6.660452	-1.228091	-5.030503	336.8552	2.12E-05	0.000647
5910598	SORL1		38.53222	NM 00310	Homo sapiens	2.081325	1.687932	3.29567	6.350621	3.168351	5.964907	1.583448	-1.842644	1.522276	-2.616901	-1.040185	-5.618571	715.1265	2.22E-06	0.000129
7000543	FAM19A4		10.54953	NM 18252	Homo sapiens	2.175841	-2.911967	3.542487	0.248691	3.163269	-0.589377	1.628101	-4.757799	1.453815	-5.678653	-1.119882	-5.56696	114.5068	0.001163	0.013167
4590364	EI24		49.66136	NM 00487	Homo sapiens	2.958871	6.066626	3.563788	7.72226	3.162486	6.636443	1.204442	-5.325923	1.068815	-6.617834	-1.126894	-5.199374	3726.808	5.68E-07	5.23E-05
1500274	LOC161931		12.38478	NM 13917	Homo sapiens	-1.140069	-6.52023	1.42778	-5.915812	3.159435	0.008394	1.627768	-4.524137	3.601975	1.20968	2.212831	-1.943408	31.49353	0.000585	0.007825
4040343	KCNK4		12.49043	NM 03331	Homo sapiens	-1.152098	-6.503434	1.854517	-4.216652	3.157841	-0.239716	2.136585	-2.656035	3.638141	1.022846	1.702783	-3.717208	52.92996	0.000563	0.0076
630450	IFIT2		21.42722	NM 00154	Homo sapiens	6.821972	2.957861	7.793591	3.423705	3.155645	-1.724263	1.142425	-6.401946	-2.161831	-3.79384	-2.46973	-2.376511	53.49511	4.53E-05	0.001142
4830047	GALNT6		9.687209	NM 00721	Homo sapiens	1.862576	-3.313707	1.989154	-3.104967	3.153736	0.655103	1.067959	-6.464772	1.693213	-4.183732	1.585466	-3.839089	68.93169	0.001655	0.017368
2100576		0	9.405402	AL832858	Homo sapiens	-1.278557	-6.291293	1.31135	-6.592601	3.150436	-1.534278	1.676635	-4.931763	4.028013	0.285499	2.402438	-2.393699	19.66614	0.001865	0.019101
670189	CDH3		24.55266	NM 00179	Homo sapiens	-1.207272	-6.099563	1.921429	-2.141491	3.148349	2.540712	2.319687	0.165962	3.800912	4.230634	1.638545	-2.853559	1211.235	2.30E-05	0.000686
240215		0	17.95452	BX104232	BX104232 So	2.209083	-0.6888	3.189241	2.341469	3.148325	2.187053	1.443695	-4.656248	1.425173	-5.019982	-1.012996	-5.662614	288.8733	0.000107	0.002166
3290470	LOC653994		13.02834	XM 94442	PREDICTED:	2.522211	-1.385707	3.83672	1.382514	3.147317	-0.078455	1.521173	-4.998518	1.247841	-6.319123	-1.219045	-5.330517	385.7529	0.000468	0.006588
5860301	CLK1		11.90456	NM 00407	Homo sapiens	2.462961	-2.388348	4.539394	1.313785	3.143255	-1.103161	1.843064	-4.179544	1.276209	-6.344154	-1.44417	-4.8147	68.30088	0.000695	0.008925
2260427	LOC389203		11.30517	XM 37169	PREDICTED:	2.472204	-1.21555	2.908382	-0.238976	3.142289	0.301345	1.176433	-6.237922	1.271048	-6.192152	1.080425	-5.607555	221.8672	0.000868	0.010512
5910504	LOC653698		26.7039	XM 93044	PREDICTED:	2.614783	2.4604	3.246084	4.26271	3.139422	3.928858	1.241435	-5.524345	1.200643	-6.0542	-1.033975	-5.641959	805.5733	1.50E-05	0.000499
1260148	LOC652864		20.09803	XM 94257	PREDICTED:	2.43336	1.02229	2.838785	2.238071	3.137558	3.088977	1.166611	-6.068506	1.289394	-5.629147	1.105247	-5.492145	2258.035	6.19E-05	0.001435
5390349	LIMK2		21.81313	NM 00103	Homo sapiens	1.894954	-1.349921	2.967083	2.935114	3.137168	3.390109	1.565781	-3.344827	1.655538	-2.949989	1.057324	-5.607397	94.43383	4.15E-05	0.001067
3870333	TJP2		31.64286	NM 20162	Homo sapiens	1.160956	-6.067044	2.176176	1.376355	3.136993	5.167237	1.874469	-0.07923	2.702076	3.893694	1.441516	-3.016235	508.6361	6.27E-06	0.000267
4540167	CSMD1		22.98893	NM 03322	Homo sapiens	1.998303	-1.56888	3.972673	4.20981	3.133729	2.28417	1.988023	-1.495196	1.568195	-4.082329	-1.267714	-4.887876	55.33676	3.19E-05	0.000871
3420392	FAM83E		20.28733	NM 01770	Homo sapiens	1.16679	-6.248931	2.134112	-0.682264	3.132518	2.966411	1.829045	-1.88723	2.684731	1.805329	1.467832	-3.623457	59.05908	5.92E-05	0.001387
5960470	NACA		21.19716	NM 00559	Homo sapiens	1.96189	-0.866975	2.804869	2.54661	3.128286	3.500064	1.429677	-4.268483	1.594527	-3.303149	1.115306	-5.441839	1079.039	4.77E-05	0.001182
2900167	C14orf118		35.03003	NM 01792	Homo sapiens	2.547514	4.032646	2.802563	4.879328	3.128117	5.916796	1.100117	-6.214243	1.22791	-5.491354	1.116163	-5.309824	194.2545	3.68E-06	0.000182
4060100	MICB		36.25999	NM 00593	Homo sapiens	1.343543	-4.202192	2.435495	3.540894	3.127495	6.083946	1.81274	0.190163	2.327796	3.177498	1.284131	-4.009138	626.4097	3.07E-06	0.000161
4040414	BIRC4		17.00924	NM 00116	Homo sapiens	2.454145	0.204856	3.033344	1.810774	3.126119	2.016888	1.236008	-5.845683	1.273812	-5.917846	1.030585	-5.652169	236.1257	0.000138	0.002624
3180164	RNU34		18.24534	NR 00001	Homo sapiens	1.326756	-5.446451	2.494548	0.43014	3.124371	2.412991	1.880186	-1.947457	2.354895	0.097362	1.25248	-4.938636	132.5017	9.88E-05	0.002047
1240739	CDS1		53.49967	NM 00126	Homo sapiens	1.446226	-1.788444	2.397471	5.682104	3.121952	8.507773	1.657743	0.694613	2.15869	4.465934	1.302186	-3.06369	637.207	3.78E-07	4.03E-05
20288	SUCLA2		37.21518	NM 00385	Homo sapiens	2.172973	2.498022	2.994413	5.796583	3.119748	6.159643	1.378026	-3.652754	1.435705	-3.291071	1.041856	-5.611451	1411.909	2.67E-06	0.000146
380152	KCNC4		21.0662	NM 00497	Homo sapiens	1.968767	-1.061421	3.013804	2.897623	3.116309	3.150186	1.530808	-3.67477	1.582874	-3.548187	1.034012	-5.644945	85.69767	4.92E-05	0.001211
2650075	AP1S1		15.38554	NM 00128	Homo sapiens	1.933875	-2.095232	2.77773	1.006889	3.114656	1.942714	1.436355	-4.750457	1.610578	-3.960406	1.121295	-5.483986	747.0836	0.00022	0.003722
3130274	RAD51		17.74405	NM 00287	Homo sapiens	2.090416	-0.834297	2.805921	1.732596	3.114295	2.608672	1.342278	-5.131655	1.489797	-4.423667	1.109901	-5.492463	515.9987	0.000113	0.002263

1500402		0	11.76781	AA594014		nn16h08.s1 N	1.272046	-5.968677	1.626629	-4.523275	3.107459	1.114599	1.27875	-5.768924	2.442882	-0.629385	1.910367	-2.261965	25.20509	0.00073	0.009243
7570364	CHAC2		41.54302	NM	00100	Homo sapiens	1.930259	0.94544	3.410071	6.90914	3.106089	6.035473	1.76664	-0.146426	1.609157	-1.65396	-1.097867	-5.397252	525.7187	1.49E-06	9.87E-05
2100086	JPH1		21.17977	NM	02064	Homo sapiens	1.886904	-1.371603	2.876559	2.687046	3.105815	3.337795	1.524486	-3.620329	1.645985	-2.995039	1.079698	-5.555726	643.5127	4.79E-05	0.001186
4250349	SAMD3		51.48157	NM	15255	Homo sapiens	4.931368	7.678673	4.895639	7.706353	3.105265	4.095821	-1.007298	-6.513172	-1.588067	-2.994483	-1.576561	-2.475968	121.095	4.67E-07	4.57E-05
2360390	ATP2C1		19.30863	NM	00100	Homo sapiens	3.084198	1.501945	3.929139	3.113167	3.103129	1.252812	1.273958	-5.767625	1.006138	-6.784521	-1.266186	-5.026935	544.1186	7.52E-05	0.001673
2070309	HNRPM		12.8075	NM	00596	Homo sapiens	1.08672	-6.642967	4.016827	0.002068	3.100031	-1.678874	3.696284	-0.110977	2.852649	-1.907704	-1.295738	-5.275582	34.76247	0.000504	0.006987
6250504	MDFIC		18.06616	NM	19907	Homo sapiens	4.303021	2.985947	3.524192	1.387772	3.093245	0.356069	-1.220995	-6.085185	-1.391103	-5.66533	-1.139319	-5.496771	55.90955	0.000104	0.002121
6420026	SLC39A6		44.99307	NM	01231	Homo sapiens	4.135151	7.202946	3.732674	6.438148	3.091513	4.773407	-1.107826	-6.229734	-1.337582	-4.774497	-1.207394	-4.845758	406.5895	9.69E-07	7.44E-05
1820521	FEN1		42.67625	NM	00411	Homo sapiens	2.436402	4.57679	2.864778	6.20382	3.089965	6.925384	1.175823	-5.508956	1.268249	-4.773749	1.078605	-5.454417	1532.211	1.29E-06	8.93E-05
6660743	BFSP1		21.99788	NM	00119	Homo sapiens	1.269637	-5.476529	2.006876	-0.545169	3.088278	3.86389	1.580669	-2.92408	2.432411	1.771858	1.538849	-2.79711	34.61941	3.98E-05	0.001029
4150097	IGSF1		20.09241	NM	20583	Homo sapiens	1.920881	-1.670941	3.199511	2.934333	3.087399	2.583682	1.665647	-3.027714	1.607282	-3.629431	-1.036313	-5.644323	658.0115	6.20E-05	0.001437
5870333	CECR2		31.13259	XM	92601	PREDICTED:	2.374481	1.479366	3.988461	5.921536	3.086601	3.751151	1.679719	-2.268991	1.299906	-5.370843	-1.292185	-4.466881	173.9231	6.82E-06	0.000281
4180670	C20orf14		9.599501	NM	01246	Homo sapiens	1.960895	-3.152468	2.567155	-1.316389	3.083386	0.055866	1.309175	-5.79863	1.572438	-4.928213	1.201091	-5.357845	179.4224	0.001717	0.017849
6760600	CLSTN1		13.1765	NM	00100	Homo sapiens	1.859959	-3.36184	3.319575	1.034327	3.08188	0.414118	1.784757	-3.550956	1.656962	-4.385379	-1.077127	-5.60872	90.11004	0.000445	0.006346
1780280	HOOK1		16.7253	NM	01588	Homo sapiens	1.301557	-5.559707	2.099357	-1.137941	3.081595	2.467101	1.612958	-3.409853	2.367622	0.296402	1.467876	-3.741048	522.7583	0.000149	0.002776
5820397	VIPR1		14.41463	NM	00462	Homo sapiens	1.5024	-4.72215	2.711604	0.283312	3.079289	1.302853	1.804848	-2.954163	2.04958	-2.00022	1.135597	-5.46655	84.33994	0.000297	0.004722
510082	ASPHD1		17.15965	NM	18171	Homo sapiens	1.366244	-5.222552	2.469402	0.277363	3.077154	2.219871	1.807439	-2.386	2.252273	-0.386451	1.246113	-4.977369	164.318	0.000132	0.002545
4290274	KIAA0251		24.18285	NM	01502	Homo sapiens	2.529427	1.451958	3.549106	4.231435	3.07643	2.98084	1.403127	-4.588936	1.216256	-6.064938	-1.153644	-5.31407	57.16358	2.48E-05	0.000722
1940156	TUBB6		15.7526	NM	03252	Homo sapiens	2.686361	0.498213	3.149892	1.557349	3.072098	1.303824	1.17255	-6.170907	1.143591	-6.536679	-1.025323	-5.657177	461.4454	0.000197	0.003414
6220762	ARHGAP22		17.39978	NM	02122	Homo sapiens	1.443846	-4.64599	2.459943	0.476452	3.071517	2.467691	1.703743	-2.833004	2.127316	-0.742433	1.248613	-4.929279	76.80301	0.000124	0.002428
2750647	GLT6D1		33.58316	NM	18297	Homo sapiens	2.187664	0.350498	4.561274	6.566985	3.070506	3.379939	2.084998	-0.061951	1.403555	-4.692247	-1.485512	-3.308011	57.61272	4.59E-06	0.000214
10414	OTUD6B		32.5063	NM	01602	Homo sapiens	2.147974	1.911544	2.91744	5.028755	3.069649	5.484646	1.358228	-4.06127	1.42909	-3.609443	1.052172	-5.590451	752.2727	5.45E-06	0.00024
4480608	STAM2		18.15427	NM	00584	Homo sapiens	3.209494	2.170353	3.160739	1.818396	3.067175	1.521745	-1.015425	-6.511018	-1.046401	-6.754675	-1.030505	-5.653114	102.6925	0.000101	0.002082
1770068	LOC653743		10.62017	XM	92936	PREDICTED:	1.457759	-5.128931	2.036988	-2.675148	3.058693	0.739203	1.397342	-5.262511	2.098216	-2.157555	1.501576	-4.093709	21.91613	0.001131	0.012891
7040341	PABPC3		22.68986	NM	03097	Homo sapiens	3.440993	2.604425	4.252036	3.962544	3.057134	1.392088	1.2357	-5.903698	-1.125562	-6.585793	-1.390857	-4.42577	343.0571	3.41E-05	0.000915
7160014	CLK2P		10.78817	XR	00026	PREDICTED:	1.933319	-3.376734	3.130283	0.039845	3.056114	-0.192909	1.619124	-4.542744	1.58076	-4.959755	-1.024269	-5.659841	51.24251	0.001059	0.012221
650369		0	11.37653	CN272139		17000600002	1.202677	-6.283091	1.835301	-3.63432	3.055082	0.670831	1.526014	-4.644417	2.540235	-0.561819	1.664622	-3.407574	33.34941	0.000845	0.010297
5310452	NUP50		32.51566	NM	15364	Homo sapiens	1.99387	1.090912	2.870605	4.97349	3.051311	5.533239	1.439715	-3.224413	1.530346	-2.585507	1.062951	-5.555781	495.7835	5.44E-06	0.000239
670367	C10orf78		22.39394	NM	14524	Homo sapiens	2.836689	2.410391	3.183577	3.216757	3.04877	2.795668	1.122286	-6.262586	1.074764	-6.684501	-1.044217	-5.63253	315.1738	3.64E-05	0.000961
6660017	LOC644774		30.73546	XM	92786	PREDICTED:	4.195342	5.247105	3.8985	4.608743	3.048458	2.556639	-1.076143	-6.416143	-1.376218	-5.131315	-1.278843	-4.750273	2928.8	7.29E-06	0.000296
2680626	SPR		24.59436	NM	00312	Homo sapiens	1.21616	-5.780681	2.068021	0.153028	3.047835	4.171762	1.700451	-1.79189	2.506113	2.463617	1.473793	-3.060993	174.3144	2.28E-05	0.000683
5290674	IVD		49.80836	NM	00222	Homo sapiens	1.392369	-2.578484	2.312055	5.071235	3.04129	8.081624	1.660519	0.592374	2.184256	4.460532	1.315406	-2.975599	736.0011	5.59E-07	5.17E-05
6940360	APBB2		12.93877	NM	17307	Homo sapiens	3.277415	0.259261	3.814445	1.027074	3.035843	-0.629198	1.163858	-6.307908	-1.079573	-6.73129	-1.25647	-5.253535	51.23099	0.000482	0.00675
7550706	LOC402644		23.37208	XM	93829	PREDICTED:	2.099885	-0.067529	3.203462	3.820668	3.032435	3.29217	1.525542	-3.53465	1.444096	-4.379087	-1.056399	-5.606869	6128.517	2.94E-05	0.000819
5290575	WWTR1		18.0602	NM	01547	Homo sapiens	1.263359	-5.7081	2.087536	-0.860713	3.025904	2.731604	1.65237	-2.935427	2.395127	0.777366	1.44951	-3.709148	43.40811	0.000104	0.002123
3170280	BAD		13.36769	NM	00432	Homo sapiens	1.162965	-6.391387	2.103344	-2.157094	3.025613	0.964232	1.808605	-3.048752	2.601638	-0.029856	1.438477	-4.288729	57.74088	0.000417	0.006078
6900050	SMYD2		10.30948	NM	02019	Homo sapiens	1.769744	-3.612661	2.334338	-1.54806	3.024047	0.556952	1.319026	-5.646751	1.708749	-3.994237	1.295462	-4.979436	47.44504	0.00128	0.014176
5360674	HAPLN3		18.62498	NM	17823	Homo sapiens	1.321976	-5.334441	2.311799	0.20364	3.021846	2.715576	1.748745	-2.327347	2.285855	0.307321	1.307141	-4.550319	277.0553	8.95E-05	0.001905
5720487	ZFP42		37.49642	NM	17490	Homo sapiens	1.3953	-3.367785	2.386587	3.922982	3.021329	6.40668	1.710447	-0.139988	2.165362	2.902682	1.265963	-4.003091	1870.024	2.57E-06	0.000143
2000059	LOC652194		12.25127	XM	94156	PREDICTED:	1.512663	-4.96995	2.867262	-0.05721	3.021175	0.303593	1.895506	-3.03253	1.997255	-2.81959	1.053679	-5.636775	87.94342	0.000613	0.008114
5270195	LOC347364		13.6253	XM	93714	PREDICTED:	2.158864	-1.942862	3.372308	1.397849	3.018935	0.486702	1.562075	-4.499229	1.39839	-5.553127	-1.117052	-5.534127	54.84077	0.000383	0.005709
5870706	SF1		27.97525	NM	00463	Homo sapiens	2.955568	3.524507	3.40666	4.602876	3.018725	3.501122	1.152624	-6.073717	1.021369	-6.774822	-1.12851	-5.375027	710.1888	1.18E-05	0.00042
10019	C10orf114		12.40587	NM	00101	Homo sapiens	2.508251	-0.361124	2.597259	-0.357167	3.015646	0.841408	1.035486	-6.498633	1.20229	-6.354097	1.161088	-5.409599	218.8531	0.00058	0.00778
7100279	THOC4		41.32052	XM	94625	PREDICTED:	2.493858	4.556308	3.014968	6.411645	3.011232	6.374364	1.208958	-5.238745	1.207459	-5.511518	-1.001241	-5.66498	4994.102	1.53E-06	0.0001
6960041	LOC653337		45.63043	XM	93282	PREDICTED:	1.279053	-4.644416	2.775227	5.629591	3.009808	6.417408	2.169751	3.034934	2.353153	3.936989	1.084527	-5.434416	195.2833	8.98E-07	7.03E-05
4890576	LOC652613		10.83545	XM	94214	PREDICTED:	2.622129	-0.679434	2.699904	-0.74967	3.008853	0.049064	1.029661	-6.505133	1.147484	-6.579953	1.11443	-5.550802	117.0143	0.001039	0.012058
7200097	UBE2D3		14.50823	NM	18188	Homo sapiens	1.981344	-2.108389	2.96213	1.21435	3.008756	1.295303	1.49501	-4.535842	1.518543	-4.651146	1.015741	-5.661857	37.12034	0.000288	0.004627
3180504	LOC644655		29.5071	XM	92776	PREDICTED:	1.321089	-4.736896	2.449841	2.759629	3.008371	4.813679	1.85441	-0.185195	2.27719	2.12314	1.227986	-4.655708	133.9828	9.01E-06	0.000345
7100386	LOC654189		36.07436	XM	94268	PREDICTED:	2.500461	2.6915	4.013141	6.768486	3.00754	4.280884	1.60496	-2.371175	1.202794	-5.932168	-1.33436	-3.99083	94.71346	3.15E-06	0.000165
360382	LOC653942		16.97586	XM	93811	PREDICTED:	2.760691	0.654945	3.547462	2.41038	3.005625	1.044729	1.28499	-5.708083	1.088722	-6.685867	-1.180274	-5.336427	3230.169	0.000139	0.002642
6370605	LOC646197		42.89197	XM	92915	PREDICTED:	2.122424	3.322135	2.794225	6.362165	3.003847	7.07111	1.316526	-3.809909	1.415291	-2.85667	1.075019	-5.456951	7307.899	1.25E-06	8.79E-05
6860326	PTRH2		29.74281	NM	00101	Homo sapiens	1.820735	-0.131848	2.637567	4.052922	3.002908	5.322423	1.448627	-3.166602	1.649283	-1.557999	1.138514	-5.194493	111.0847	8.64E-06	0.000335
4250142	KIAA0564		28.65054	NM	00100	Homo sapiens	1.774176	-0.540692	2.553235	3.626768	3.001979	5.235846	1.439111	-3.297454	1.692042	-1.257686	1.175755	-4.960612	246.3198	1.05E-05	0.000383
4230678	PRKAG2		31.32915	NM	01620	Homo sapiens	1.957012	1.165666	2.563352	4.171502	3.000497	5.754845	1.30983	-4.367854	1.533203	-2.334716	1.170536	-4.931812	332.6439	6.60E-06	0.000275
2100241	FLJ23834		23.68311	NM	15275	Homo sapiens	4.376817	2.998434	5.549854	4.300841	3.000131	0.03113	1.268011	-5.927405	-1.458876	-5.382476	-1.849871	-2.898939	78.61941	2.75E-05	0.000786
7050139	STAT1		24.81567	NM	13926	Homo sapiens	2.308083	1.40709	3.040086	3.909388	2.998219	3.741611	1.317148	-4.917833	1.299009	-5.312781	-1.013964	-5.660867	272.9182	2.18E-05	0.000659
4570138	LOC642859		14.85786	XM	93207	PREDICTED:	1.720771	-2.759173	2.153194	-0.698975	2.99442	2.435226	1.251296	-5.647121	1.740162	-2.719931	1.390688	-4.11628	68.71508	0.000259	0.004239
4890519	FAM21C		14.01952	NM	01526	Homo sapiens	2.182633	-2.380904	4.09079	2.003585	2.993676	-0.286557	1.874246	-3.431429	1.371589	-5.836072	-1.366477	-4.842278	34.23333	0.000337	0.005191
3800243	PBEF1		12.0888	NM	00574	Homo sapiens	2.677101	-0.563144	3.198969	0.517141	2.989429	-0.052876	1.194938	-6.18164	1.116667	-6.65391	-1.070094	-5.620604	579.7094	0.00065	0.00849
7200338	LOC642502		19.96102	XM	92659	PREDICTED:	2.020722	-1.039104	3.198167	3.072746	2.988734	2.438652	1.582685	-3.485807	1.479042	-4.419193	-1.070074	-5.588606	597.6851	6.40E-05	0.001473
4050376	RPS6KB2		13.22401	NM	00395	Homo sapiens	1.41633	-5.086063	2.226329	-1.191296	2.986257	1.406366	1.5719	-4.028309	2.108447	-1.46689	1.341337	-4.616468	309.0061	0.000438	0.006269
4290292	COQ6		12.38095	NM	18247	Homo sapiens	2.409837	-0.40269	2.344785	-0.950981	2.982703	1.105728	-1.027743	-6.503911	1.23772	-6.169728	1.272058	-4.978668	55.28205	0.000586	0.007834
1090438	ZNF114		32.13623	NM	15360	Homo sapiens	2.130128	2.272289	2.627292	4.484104	2.981068	5.74033	1.233396	-5.118344	1.399478	-3.658449	1.134654	-5.176662	279.4162	5.78E-06	0.00025
4890047	FLJ10847		42.42203	NM	01824	Homo sapiens	1.389443	-3.276249	2.616916	5.278458	2.980775	6.601405	1.883428	1.466496	2.145303	3.077333	1.139041	-5.062919	822.6543	1.33E-06	9.12E-05
2060497	SLC4A7		39.26844	NM	00361	Homo sapiens	2.708236	4.354006	3.579181	6.812342	2.980417	5.134317	1.321591	-4.438618	1.100501	-6.497427	-1.2009	-4.785988	408.4586	2.01E-06	0.00012
3850348	LOC644816		25.84751	XM	92791	PREDICTED:	1.585043	-2.619953	2.667058	3.230874	2.980187	4.278568	1.68264	-1.722	1.880192	-0.519361	1.117406	-5.37365	551.9616	1.77E-05	0.000568
7570739	STEAP2		25.56548	NM	15299	Homo sapiens	1.598294	-2.894979	3.033997	3.815055	2.973941	3.590114	1.898273	-0.722608	1.860697	-1.142362	-1.020194	-5.656504	216.5745	1.87E-05	0.000592
2750670	DDX31		15.7906	NM	13862	Homo sapiens	2.371568	-0.432553	3.237486	1.963114	2.971052	1.205912	1.365125	-5.249733	1.25278	-6.082703	-1.089677	-5.569487	32.93193	0.000195	0.003385
4760341	CYCSL1		27.57562	NR	00156	Homo sapiens	2.047006	0.599464	3.043899	4.549947	2.966024	4.269304	1.487	-3.274413	1.448957	-3.836382	-1.026256	-5.648597	3357.717	1.28E-05	0.000443
7160364	BCCIP		37.65319	NM	07846	Homo sapiens	2.495746	3.842217	3.267151	6.370672	2.962933	5.425367	1.309088	-4.444706	1.187193	-5.849491	-1.102675	-5.380613	2477.867	2.51E-06	0.000141
6200315	THYN1		17.59547	NM	01417	Homo sapiens	1.831154	-1.838434	2.563256	1.404194	2.960612	2.715272	1.399804	-4.58614	1.616801	-3.302553	1.15502	-5.302939	294.54	0.000117	0.002331
2470743	CHMP2A		14.50509	NM	01445	Homo sapiens	1.445495	-4.786969	2.337841	-0.39608	2.958953	1.713585	1.617329	-3.58198	2.047017	-1.460822	1.265678	-4.909234	132.7419	0.000289	0.004628
7050451	GCLM		35.68277	NM	00206	Homo sapiens	2.057506	2.112522	2.820074	5.530624	2.957881	5.978861	1.370627	-3.58771	1.437605	-3.102114	1.048867	-5.588018	695.7142	3.34E-06	0.00017
3990138		0	14.86081	BM790440		K-EST007027	1.258195	-5.818814	1.831085	-2.595873	2.95293	2.061166	1.455327	-4.434482	2.346958	0.183061	1.612667	-2.957022	20.16194	0.000258	0.004238
20066	SLC9A7		10.58794	NM	03259	Homo sapiens	1.43468	-5.331557	2.415923	-1.435834	2.951683	0.150486	1.683946	-3.979109	2.057381	-2.54698	1.221762	-5.262764	53.3025	0.001145	0.013007
3460309	LIPT1		17.08495	NM	14519	Homo sapiens	2.54242	0.51737	3.185125	2.211294	2.950488	1.517013	1.252793	-5.76427	1.160504	-6.443225	-1.079525	-5.58275	364.0867	0.000135	0.002585
6420630	NOMO1		38.37607	NM	01428	Homo sapiens	2.286721	3.588713	2.893477	6.008412	2.946812	6.162984	1.265339	-4.648543	1.288663	-4.642325	1.018433	-5.653195	1125.581	2.27E-06	0.000131

6130100	NDUFB1		23.83996	NM	00454	Homo sapiens	1.512306	-3.19419	2.373725	2.053715	2.946017	4.227209	1.569607	-2.573812	1.94803	-0.038097	1.241094	-4.631407	215.6985	2.66E-05	0.000765
3440133	TMEM98		12.94186	NM	01554	Homo sapiens	2.716034	-0.217411	3.249557	0.899103	2.943224	0.078002	1.196435	-6.15701	1.083648	-6.710948	-1.104081	-5.564874	294.8953	0.000482	0.006745
1260228	PLSCR1		35.84848	NM	02110	Homo sapiens	2.615194	3.668922	3.561744	6.398138	2.942582	4.645639	1.361943	-4.189444	1.125187	-6.381912	-1.210414	-4.774086	1825.751	3.26E-06	0.000168
5820176	LOC552891		10.97939	NM	00412	Homo sapiens	2.648192	-0.849869	3.089071	0.024601	2.938892	-0.406921	1.166483	-6.276704	1.109773	-6.674106	-1.0511	-5.642191	266.5458	0.000983	0.011546
2030139	LOC550112		13.68439	XR	001037	PREDICTED:	2.357095	-0.454733	2.726959	0.559063	2.93838	1.151435	1.156915	-6.211314	1.246611	-6.106088	1.07753	-5.59202	124.7237	0.000376	0.005629
2600167	THAP1		26.43293	NM	01810	Homo sapiens	2.451938	2.643616	2.78552	3.750326	2.936567	4.224702	1.136048	-6.083334	1.197651	-5.943251	1.054226	-5.596475	239.196	1.58E-05	0.00052
5080576	CALCR		13.74347	NM	00174	Homo sapiens	7.484419	1.657327	6.136645	0.480396	2.934811	-3.365585	-1.219627	-6.343696	-2.550222	-3.766902	-2.090985	-3.876994	230.0296	0.000368	0.00555
7200743	LRRC8B		22.28375	NM	01535	Homo sapiens	2.044636	-1.183937	3.857751	4.213126	2.931429	1.916301	1.886767	-1.874083	1.433717	-4.85153	-1.315997	-4.608473	76.6398	3.73E-05	0.000978
2320546	LOC647916		24.4465	XM	93901	PREDICTED:	2.284008	1.463479	2.927594	3.752044	2.931307	3.723514	1.281779	-5.141579	1.283405	-5.385354	1.001268	-5.664999	1999.209	2.35E-05	0.000694
2750408	RUVBL1		23.62402	XM	93970	PREDICTED:	2.154473	0.849504	2.826905	3.446603	2.930516	3.749593	1.312109	-4.895811	1.360201	-4.755812	1.036652	-5.635773	1814.424	2.79E-05	0.000792
7570674	CHRM3		11.19101	NM	00074	Homo sapiens	1.43057	-5.288115	2.456097	-1.102794	2.927072	0.304109	1.716866	-3.711598	2.046088	-2.436413	1.191757	-5.337794	99.25043	0.000906	0.010859
1580026	LNK		19.85066	NM	00547	Homo sapiens	1.453134	-4.091008	2.308487	0.95108	2.925034	3.281046	1.588626	-2.909414	2.012913	-0.34653	1.267078	-4.621493	1214.344	6.58E-05	0.001505
5810373	WNK1		15.49057	NM	01897	Homo sapiens	2.797012	0.780233	3.193161	1.605682	2.923457	0.832026	1.141633	-6.277626	1.045207	-6.75794	-1.092255	-5.569315	283.5488	0.000213	0.003632
4490242	TCEA1		17.34077	NM	00675	Homo sapiens	2.969229	0.680677	4.154735	2.911731	2.919186	0.21921	1.399264	-5.290384	-1.017143	-6.781533	-1.423251	-4.471566	289.6449	0.000126	0.002457
4220091	ALDH3A2		29.13644	NM	00103	Homo sapiens	1.323347	-4.503751	2.193526	2.070171	2.912269	5.132288	1.657559	-1.220274	2.200683	2.288916	1.327665	-3.708116	556.4231	9.61E-06	0.000362
7650554	DYSF		32.34293	NM	00349	Homo sapiens	-1.086779	-6.506135	2.141058	0.776374	2.909691	3.989854	2.326856	2.016635	3.16219	4.904605	1.358997	-3.802413	236.388	5.59E-06	0.000244
3180167		0	11.59276	CB160856		K-EST022061	2.289931	-0.805979	2.199686	-1.488363	2.907042	0.954453	-1.041026	-6.491394	1.269489	-6.013619	1.321571	-4.752012	96.32075	0.000779	0.009697
1500564	ATP5F1		31.17975	NM	00168	Homo sapiens	2.563723	3.317808	3.203924	5.309193	2.904939	4.357416	1.249715	-5.230927	1.133094	-6.347856	-1.102923	-5.422262	3777.981	6.77E-06	0.00028
6650541	TRA16		25.35093	NM	17688	Homo sapiens	1.937123	0.274132	2.582694	3.343021	2.904665	4.493703	1.333262	-4.460765	1.499473	-3.17954	1.124665	-5.310512	862.386	1.96E-05	0.000609
2710162	LOC342541		15.59469	XM	93986	PREDICTED:	1.755231	-3.445633	3.435807	2.053822	2.902013	0.649194	1.957467	-2.322662	1.653351	-4.061608	-1.183939	-5.331991	207.2345	0.000207	0.003547
6220139	SS18		26.02219	NM	00563	Homo sapiens	3.063742	3.864966	3.051898	3.669091	2.900968	3.159712	-1.003881	-6.51411	-1.05611	-6.716913	-1.052027	-5.612486	1913.882	1.71E-05	0.000553
3520082	RUVBL1		21.04191	NM	00370	Homo sapiens	1.897982	-1.026814	2.839613	2.944985	2.900093	3.099606	1.496122	-3.652256	1.527988	-3.646186	1.021299	-5.656026	1545.971	4.95E-05	0.001217
3780382	LHX4		12.83174	NM	03334	Homo sapiens	1.090395	-6.58356	1.746068	-3.703953	2.899065	0.888754	1.601317	-3.982971	2.658729	0.412975	1.660339	-3.155832	25.92397	0.0005	0.006943
4010048	ACOX1		34.07573	NM	00403	Homo sapiens	1.93591	1.417185	2.67331	5.088714	2.897223	5.888618	1.380906	-3.425403	1.496569	-2.418526	1.083759	-5.444511	402.7276	4.26E-06	0.000202
4070082	PTK9		17.58328	NM	19897	Homo sapiens	1.609843	-3.230288	2.496302	1.206178	2.896674	2.583949	1.550649	-3.493783	1.799352	-2.06407	1.160386	-5.274989	36.59448	0.000118	0.002337
6060372	LOC642269		18.18943	XM	93069	PREDICTED:	2.362792	0.354458	3.083916	2.53877	2.896218	1.940272	1.3052	-5.366773	1.225761	-6.082055	-1.064808	-5.602159	535.0274	0.0001	0.002068
1240296		0	24.09833	AW606588		QV0-HT0398-	2.867961	2.162589	3.90492	4.485367	2.894801	1.970991	1.361567	-5.008655	1.009359	-6.783463	-1.348942	-4.394132	113.9298	2.52E-05	0.000733
4010196	ATP6V0A4		23.14011	NM	13084	Homo sapiens	1.519159	-3.056953	2.280996	1.745104	2.89411	4.205934	1.501486	-3.064896	1.905073	-0.195805	1.268792	-4.403233	30.00542	3.09E-05	0.000851
3390239	NUDT10		22.49779	NM	15318	Homo sapiens	1.860921	-1.193481	2.984569	3.492411	2.89333	3.161462	1.603812	-2.807495	1.554784	-3.40003	-1.031534	-5.645175	143.3451	3.56E-05	0.000946
7400719	NUP98		14.07087	NM	13913	Homo sapiens	2.501495	-0.13035	2.99381	1.122802	2.892161	0.783148	1.196808	-6.081765	1.156173	-6.496609	-1.035147	-5.650112	129.188	0.000331	0.005124
3800025		0	16.57857	AK000776		Homo sapiens	3.641592	1.561485	4.191652	2.285624	2.890748	-0.452197	1.151049	-6.311492	-1.25974	-6.247746	-1.450023	-4.51136	857.9634	0.000156	0.002857
5890138	CER1		18.29256	NM	00545	Homo sapiens	8.848235	2.765062	7.617159	1.876989	2.889668	-3.21303	-1.161619	-6.408365	-3.062025	-2.574746	-2.635998	-2.728475	254.7409	9.76E-05	0.002032
2570255	TRIM38		37.4893	NM	00635	Homo sapiens	1.360493	-3.381504	2.069197	2.89162	2.88804	6.778979	1.520917	-1.391012	2.122789	3.384868	1.39573	-2.437314	32.36669	2.57E-06	0.000143
4760095	GNAQ		41.77904	NM	00207	Homo sapiens	2.427757	4.748824	2.856912	6.40519	2.874934	6.444213	1.17677	-5.458793	1.184194	-5.642111	1.006308	-5.663484	663.3665	1.44E-06	9.63E-05
4230681	NOM1		21.00874	NM	13840	Homo sapiens	1.705672	-2.387587	2.896383	2.94676	2.874706	2.832612	1.698089	-2.302991	1.68538	-2.605896	-1.007541	-5.663931	113.2525	4.99E-05	0.001223
4920221	GSTM4		20.1884	NM	00085	Homo sapiens	2.242929	-0.077342	3.419645	3.500142	2.869324	1.94662	1.524634	-3.92561	1.279275	-5.763329	-1.191795	-5.18526	66.57417	6.06E-05	0.00141
2140471		0	30.24311	BQ722442		AGENCOURT	1.733779	-2.165348	3.99574	5.71174	2.869285	2.866717	2.304642	1.067243	1.654931	-2.787004	-1.392591	-3.868369	36.05753	7.93E-06	0.000313
6290093	SH3GL3		26.96393	NM	00302	Homo sapiens	1.753849	-0.876358	2.671138	3.860603	2.867864	4.540059	1.523015	-2.65282	1.635183	-1.896456	1.073649	-5.528021	1109.407	1.43E-05	0.00048
510563	ORC6L		25.79942	NM	01432	Homo sapiens	2.597673	2.489497	3.271632	4.433771	2.864727	3.185708	1.259447	-5.379285	1.102805	-6.562509	-1.14204	-5.305331	1000.894	1.79E-05	0.000572
3420632	DOPEY2		17.69013	NM	00512	Homo sapiens	1.95786	-1.664711	3.185482	2.623964	2.862098	1.648914	1.627022	-3.407962	1.46185	-4.69081	-1.112988	-5.491266	119.6077	0.000114	0.002287
6560324	PFN1		24.59082	NM	00502	Homo sapiens	2.154198	1.04622	2.881401	3.862773	2.858863	3.746856	1.337575	-4.617263	1.327113	-4.956022	-1.007884	-5.663572	18129.4	2.28E-05	0.000683
240730	ZBTB8		10.30606	NM	14462	Homo sapiens	1.410904	-5.317729	2.132873	-2.14325	2.857641	0.346698	1.511706	-4.636915	2.025397	-2.353552	1.339808	-4.759087	52.07029	0.001282	0.014185
1190634	SERTAD3		11.54581	NM	20334	Homo sapiens	1.862681	-3.590987	3.39911	0.777527	2.853975	-0.588921	1.824848	-3.60004	1.532186	-5.12788	-1.191009	-5.388114	40.94875	0.000793	0.009845
5310367	MAML1		16.27243	NM	01475	Homo sapiens	2.186192	-0.846293	3.173269	2.22635	2.853934	1.271956	1.451505	-4.633894	1.305436	-5.740312	-1.111893	-5.50663	263.5384	0.00017	0.003048
7560372	NFRKB		51.49674	NM	00616	Homo sapiens	3.322837	7.156981	3.594206	7.875169	2.853353	5.713238	1.081668	-6.28175	-1.164538	-5.943314	-1.259643	-4.094036	209.0867	4.66E-07	4.57E-05
5420167	SCAND1		25.35159	NM	01655	Homo sapiens	1.531937	-2.651619	2.3239	2.447476	2.850818	4.590019	1.516968	-2.647268	1.860923	-0.080303	1.226739	-4.611643	480.2964	1.96E-05	0.000609
6520639	LOC644063		13.15194	XM	93157	PREDICTED:	2.386524	-1.115938	3.37511	1.373554	2.848155	-0.010031	1.414237	-5.22756	1.193432	-6.41966	-1.185017	-5.364353	1168.675	0.000448	0.006386
6400241	FGD6		32.58805	NM	01835	Homo sapiens	2.668683	4.36558	2.845705	4.883234	2.846248	4.850369	1.066333	-6.380465	1.066536	-6.649005	1.000191	-5.665034	347.8342	5.38E-06	0.000238
4010519	RAE1		20.2654	NM	00361	Homo sapiens	2.12782	-0.121026	3.066704	3.192402	2.845669	2.467752	1.441243	-4.249743	1.337364	-5.242123	-1.077674	-5.562501	121.8111	5.95E-05	0.001391
4210524	VPS16		15.29816	NM	02257	Homo sapiens	3.58796	1.647879	3.601687	1.431141	2.845171	-0.401643	1.003826	-6.514293	-1.26107	-6.220554	-1.265895	-5.147259	66.71624	0.000226	0.003803
6520082	MOSPD1		35.52205	NM	01955	Homo sapiens	2.020058	1.835376	2.950896	5.933289	2.844094	5.53576	1.460798	-2.662782	1.407927	-3.444341	-1.037552	-5.619347	458.7941	3.42E-06	0.000173
3460102	NGRN		36.24521	NM	01664	Homo sapiens	2.781214	5.175017	2.852086	5.334341	2.843122	5.270376	1.025482	-6.491924	1.022259	-6.767623	-1.003153	-5.664716	567.7586	3.08E-06	0.000161
130138	PITPNC1		25.7293	NM	18167	Homo sapiens	1.463137	-3.317013	2.343651	2.486456	2.842446	4.498796	1.601798	-1.941464	1.942706	0.424308	1.212828	-4.725866	1224.658	1.81E-05	0.000577
3310019	GLRX2		13.86152	NM	19796	Homo sapiens	1.977185	-1.539081	2.45292	0.302474	2.841873	1.620337	1.240612	-5.761426	1.437333	-4.835192	1.158567	-5.339097	201.9632	0.000354	0.005397
1850301	SLCO4A1		30.24745	NM	01635	Homo sapiens	1.324759	-4.689134	2.69876	3.824662	2.841735	4.304881	2.03717	0.994516	2.145096	1.474357	1.052978	-5.594257	702.3861	7.92E-06	0.000313
110743	GTF2H2		14.39638	NM	00151	Homo sapiens	2.564424	-0.475975	3.601888	1.912417	2.840285	0.015666	1.40456	-5.260449	1.107572	-6.659265	-1.268143	-5.08609	284.3735	0.000299	0.004743
4180368	MPST		13.52176	NM	00101	Homo sapiens	1.491799	-4.385525	2.178679	-0.84484	2.839656	1.639761	1.460437	-4.428714	1.903511	-1.985334	1.303384	-4.673089	51.37404	0.000396	0.005849
4640435	ZNF597		10.81442	NM	15245	Homo sapiens	2.414095	-1.712497	3.375063	0.495084	2.836717	-0.845734	1.398066	-5.502108	1.175065	-6.532695	-1.189778	-5.404914	54.88091	0.001048	0.012134
7550546	CHPF		11.47044	NM	02453	Homo sapiens	1.409476	-5.05632	1.826884	-2.963313	2.836263	1.187211	1.296145	-5.536533	2.012282	-1.747428	1.552514	-3.48235	752.5906	0.000815	0.010056
2710093	SEC22L3		42.42621	NM	03297	Homo sapiens	1.420589	-2.271699	2.233474	4.541315	2.835074	7.275032	1.572216	-0.380063	1.995703	3.170678	1.269356	-3.532422	648.5071	1.33E-06	9.12E-05
4070341	DNMT3B		29.64195	NM	00689	Homo sapiens	2.270906	1.711529	3.421982	5.515774	2.834839	3.756188	1.50688	-3.145242	1.24833	-5.57987	-1.207117	-4.875228	900.4642	8.80E-06	0.000339
2360598	TRIM5		14.99189	NM	03303	Homo sapiens	2.782955	1.332426	2.545702	0.273468	2.831393	1.180413	-1.093197	-6.390359	1.017405	-6.78029	1.112225	-5.506494	544.9175	0.000248	0.0041
1110138	DOCK5		11.94259	NM	02494	Homo sapiens	-1.202606	-6.329093	1.446887	-5.732486	2.831124	-0.460578	1.740035	-3.863042	3.404728	1.202309	1.9567	-2.538822	52.48763	0.000685	0.008846
1340619	RPL36		34.78902	NM	01541	Homo sapiens	1.311672	-4.047198	1.94853	1.911782	2.830879	6.409247	1.485531	-1.877105	2.158221	3.447844	1.452828	-1.962085	767.1227	3.82E-06	0.000188
4640152	AASS		13.96385	NM	00576	Homo sapiens	1.762551	-3.057745	2.805912	1.007468	2.828619	1.027139	1.591961	-3.872905	1.604844	-4.037933	1.008093	-5.664139	1915.784	0.000343	0.005252
3710066	SEC24D		46.26225	NM	01482	Homo sapiens	1.688021	0.029813	3.136574	7.42356	2.828176	6.379464	1.858137	1.528005	1.675439	-0.150634	-1.109045	-5.254067	1588.734	8.34E-07	6.66E-05
3370246	USP48		16.98553	NM	00103	Homo sapiens	1.723578	-2.844201	2.897662	2.018757	2.826058	1.745474	1.68119	-2.960865	1.639647	-3.449414	-1.025337	-5.654943	193.716	0.000139	0.002637
7550243	ESRRA		18.89083	NM	00445	Homo sapiens	1.502374	-3.43189	2.021524	-0.060465	2.824791	3.51815	1.345553	-4.587151	1.880218	-0.706164	1.397357	-3.580632	510.412	8.36E-05	0.001807
380243	LOC643025		23.4996	XM	92616	PREDICTED:	2.673803	2.454777	3.161996	3.792383	2.823013	2.713671	1.182584	-5.927067	1.055804	-6.72014	-1.120079	-5.417063	3872.093	2.86E-05	0.000807
6200390		0	17.74029	BU078529		in27e08.x1 Hu	1.31484	-5.348042	2.35742	0.52441	2.822817	2.232255	1.792933	-1.970387	2.14689	-0.224568	1.197418	-5.115012	120.6389	0.000113	0.002265
3990025	PLS3		27.65767	NM	00503	Homo sapiens	2.652299	2.897664	3.367186	4.91178	2.822668	3.265479	1.269535	-5.257481	1.064234	-6.689926	-1.192909	-5.020803	2951.67	1.26E-05	0.000438
2760735	POLR3G		25.27427	NM	00646	Homo sapiens	-1.415219	-5.047763	2.270922	-0.880692	2.821907	1.062205	3.213853	2.479319	3.993618	4.03475	1.242626	-5.045992	52.44383	1.99E-05	0.000617
5270164	LOC645508		16.52877	XM	92853	PREDICTED:	1.893974	-3.267076	4.383133	2.905773	2.821011	-0.371831	2.314251	-1.486578	1.489466	-5.215729	-1.553745	-4.020844	41.22614	0.000158	0.002888
6250551	PTHB1		12.9547	NM	00103	Homo sapiens	1.112488	-6.513089	1.636619	-4.072986	2.819628	1.098774	1.471134	-4.541039	2.534525	0.40833	1.722837	-2.657805	1160.746	0.00048	0.00673
6270326	HLA-C		19.22558	NM	00211	Homo sapiens	2.336876	0.26589	3.327412	3.20231	2.81614	1.707915	1.423872	-4.612319	1.205088	-6.186951	-1.18155	-5.235411	45.41694	7.68E-05	0.001695
6370121	ATP1A1		38.49506	NM	00070	Homo sapiens	2.073091	2.879073	2.756751	6.061217	2.814328	6.249923	1.329778	-3.718218	1.357551	-3.624333	1.020886	-5.64823	648.082	2.23E-06	0.000129
4570309	CKMT1A		21.33143	NM	00101	Homo sapiens	1.19057	-6.044587	2.347833	0.97441	2.812709	2.738616	1.972025	-0.536213	2.36249	1.242009	1.198002	-5.051222	1840.774	4.63E-05	0.001156

6290647		0	14.27304	CX783027	HESC3 18 A	2.240411	-0.765558	2.913732	1.317078	2.808646	0.94819	1.300535	-5.5435	1.25363	-6.044954	-1.037415	-5.646529	44.47014	0.00031	0.004873
2940026	ANKRD19		12.21961	NM 00101	Homo sapiens	2.118292	-1.921897	3.024248	0.801736	2.806036	0.136187	1.427682	-5.085519	1.324669	-5.846324	-1.077765	-5.601223	119.1524	0.00062	0.00819
4280669	SMARCA2		56.5973	NM 13904	Homo sapiens	-1.191572	-5.370873	1.908952	1.926664	2.805244	6.66829	2.274655	4.419229	3.342652	8.322163	1.46952	-1.637247	676.4995	2.78E-07	3.31E-05
2510035	ARHGAP9		16.14395	NM 03249	Homo sapiens	1.498879	-4.300716	2.65826	1.177301	2.804535	1.620747	1.773499	-2.441893	1.871089	-2.098212	1.055027	-5.619486	68.32052	0.000176	0.003135
5700528	LOC649604		10.79995	XM 94514	PREDICTED:	1.467948	-4.757224	1.880386	-2.769277	2.80408	0.94909	1.280962	-5.642237	1.910203	-2.357253	1.491226	-3.845015	39.00815	0.001054	0.012183
1190706	FAM18B		27.42145	NM 01607	Homo sapiens	2.617084	3.027834	3.165629	4.656354	2.80384	3.478104	1.209602	-5.642101	1.07136	-6.662101	-1.129034	-5.33239	389.0202	1.31E-05	0.000452
6510707	C1orf171		9.233658	NM 13846	Homo sapiens	1.719221	-3.732176	2.135526	-2.142506	2.802538	0.171377	1.242148	-5.938929	1.630121	-4.274516	1.312341	-4.875721	523.1055	0.002009	0.020246
5260438	FLJ22662		14.31161	NM 02482	Homo sapiens	1.418231	-4.730693	2.09709	-0.931439	2.800405	1.88324	1.478666	-4.16351	1.974576	-1.328717	1.335377	-4.420494	354.7204	0.000307	0.004834
270593	SNFT		34.67755	NM 01866	Homo sapiens	1.121133	-6.236384	2.24358	2.698217	2.798401	5.11907	2.001172	1.570799	2.496048	4.066289	1.247293	-4.299038	116.0759	3.88E-06	0.000189
4480221	SLC39A14		35.87604	NM 01535	Homo sapiens	1.467523	-2.327078	2.411388	4.476337	2.797598	6.085335	1.643169	-0.412862	1.906341	1.619218	1.160161	-4.877448	2507.657	3.25E-06	0.000168
730544	SLC30A1		22.77211	NM 02119	Homo sapiens	1.585967	-2.39556	2.342652	2.194999	2.796623	4.029618	1.477113	-3.196684	1.763355	-1.063626	1.193785	-4.905282	676.3958	3.35E-05	0.000903
7380519	HEY2		20.37653	NM 01225	Homo sapiens	1.282637	-5.400455	2.323047	1.018577	2.796085	2.853663	1.811149	-1.374546	2.179949	0.540791	1.203628	-4.999611	279.0011	5.79E-05	0.001366
1340709	WDR77		14.92187	NM 02410	Homo sapiens	1.321486	-5.24954	1.714891	-2.804263	2.794948	2.345412	1.297699	-5.247827	2.115004	-0.212261	1.629811	-2.508316	148.1276	0.000253	0.004173
4250291	AOX1		34.19668	NM 00115	Homo sapiens	2.708085	3.641265	3.728635	6.364805	2.791269	3.731804	1.376853	-4.204454	1.030717	-6.759718	-1.335821	-3.932221	143.4203	4.18E-06	0.0002
6650020	ADM		17.50507	NM 00112	Homo sapiens	2.006648	-1.169808	3.020951	2.502067	2.789071	1.737614	1.505472	-4.030122	1.389916	-5.046974	-1.083139	-5.560728	9336.655	0.00012	0.002371
5570307	PHF19		18.28256	NM 00100	Homo sapiens	-1.16549	-6.321648	1.87608	-2.639604	2.787378	1.11656	2.186552	-0.68817	3.248661	2.646971	1.485746	-3.79916	156.3267	9.78E-05	0.002034
3780209	PDLIM1		33.88869	NM 02099	Homo sapiens	1.43936	-2.616427	2.220245	3.516359	2.78729	6.093042	1.542523	-1.367656	1.936479	1.876932	1.255397	-3.973873	4128.537	4.38E-06	0.000207
7550709	KIAA1411		18.90687	NM 02081	Homo sapiens	3.646211	2.230992	4.141329	2.914271	2.785177	-0.141584	1.13579	-6.321853	-1.309148	-5.958513	-1.486918	-4.194047	251.8592	8.33E-05	0.001803
6020647	LOC642443		14.15147	XM 93103	PREDICTED:	2.167549	-0.943283	2.818877	1.195077	2.78312	1.031768	1.300491	-5.510738	1.283994	-5.860662	-1.012848	-5.662713	948.47	0.000323	0.00503
7400343	KIAA0514		15.78956	NM 01469	Homo sapiens	1.496272	-4.018848	2.245921	0.145214	2.782591	2.235922	1.501012	-3.831566	1.859683	-1.676653	1.238953	-4.883378	94.30697	0.000195	0.003385
4210575	LOC653938		17.81074	XM 93699	PREDICTED:	1.59094	-3.291436	2.585629	1.679019	2.779803	2.330709	1.625221	-2.907835	1.747271	-2.308874	1.075097	-5.567258	134.7402	0.000111	0.002229
5490768	SPTLC1		41.98464	NM 00641	Homo sapiens	3.055378	5.839409	3.466734	6.926092	2.779744	4.819243	1.134633	-5.99217	-1.099158	-6.484139	-1.247142	-4.35194	420.3821	1.41E-06	9.46E-05
2750403	LOC51136		35.29359	NM 01612	Homo sapiens	3.575562	5.93136	3.310675	5.223541	2.778853	3.554374	-1.08001	-6.357999	-1.286704	-5.269751	-1.191382	-4.97516	93.54824	3.54E-06	0.000178
6620040	RNU33		18.06742	NR 00002	Homo sapiens	1.721368	-2.01979	2.374061	1.361603	2.778149	2.912191	1.379171	-4.466852	1.613919	-2.876873	1.17021	-5.166193	104.2016	0.000104	0.002121
6550523	TATDN3		31.2819	XM 93184	PREDICTED:	2.058719	1.643675	2.919822	5.323097	2.777181	4.787557	1.418271	-3.347608	1.348985	-4.284702	-1.051362	-5.588641	302.8731	6.65E-06	0.000277
2370471	WHSC1L1		53.82567	NM 02303	Homo sapiens	4.094885	6.915325	5.294635	8.740529	2.77502	3.513561	1.292988	-4.970404	-1.475624	-3.6576	-1.907963	-0.348361	56.66171	3.66E-07	3.95E-05
70575	ENDOGL1		32.1607	NM 00510	Homo sapiens	2.535624	3.390951	3.28673	5.742634	2.774549	4.103782	1.296222	-4.775765	1.094228	-6.545185	-1.1846	-4.945983	165.6064	5.76E-06	0.00025
2450519	MCM3		41.34975	NM 00238	Homo sapiens	2.153281	3.968996	2.611022	6.148588	2.774116	6.780503	1.212578	-4.904469	1.28832	-4.195707	1.062464	-5.503515	4035.9	1.53E-06	0.0001
2640156	IL27RA		28.70202	NM 00484	Homo sapiens	1.675159	-1.398071	2.830634	4.586278	2.771292	4.336483	1.68977	-1.159108	1.654346	-1.644124	-1.021413	-5.652368	537.6452	1.04E-05	0.000381
1770672	IFI30		28.4886	NM 00633	Homo sapiens	1.290707	-4.894716	2.398818	2.903248	2.769503	4.400334	1.85853	0.144177	2.145725	1.786943	1.154528	-5.106034	855.593	1.08E-05	0.000391
160553	WTAP		31.18666	NM 15285	Homo sapiens	2.807571	4.172923	3.184771	5.218654	2.766686	3.840576	1.134351	-6.076886	-1.014778	-6.778837	-1.151114	-5.179542	1692.815	6.76E-06	0.00028
5550224	TFF3		9.986027	NM 00322	Homo sapiens	2.820885	-1.488119	3.923222	0.377541	2.759099	-2.019783	1.390777	-5.742849	-1.022394	-6.781293	-1.421921	-4.884725	675.2923	0.001461	0.015777
6370541	DPP3		22.94864	NM 00570	Homo sapiens	1.46738	-3.588935	2.478429	2.505066	2.759039	3.569239	1.689017	-1.648328	1.880249	-0.488468	1.113221	-5.390426	428.1183	3.22E-05	0.000876
6620431	ITPR3		11.23354	NM 00222	Homo sapiens	1.368808	-5.376868	2.099464	-1.81902	2.758818	0.64135	1.53379	-4.281704	2.01549	-1.95952	1.314058	-4.760819	2186.519	0.000892	0.010737
4230544	FLJ39599		12.53854	NM 17380	Homo sapiens	1.440566	-5.134461	2.752594	0.187222	2.758591	0.152454	1.910772	-2.586199	1.914936	-2.787035	1.002179	-5.664978	104.6201	0.000554	0.007495
2650307	BDNF		11.62272	NM 00170	Homo sapiens	1.067284	-6.631994	1.811241	-3.489662	2.755708	0.249258	1.697057	-3.575537	2.581982	-0.034161	1.521447	-3.884221	91.23811	0.000771	0.009612
4760674	AP3B2		16.4335	NM 00464	Homo sapiens	2.919382	1.077108	3.514541	2.297944	2.751587	0.263887	1.203865	-6.0636	-1.060981	-6.737157	-1.277278	-4.980048	280.5317	0.000162	0.002943
5810504	AZIN1		9.614385	NM 01587	Homo sapiens	2.197114	-3.06003	3.784893	0.351551	2.751506	-1.872201	1.722666	-4.53102	1.252328	-6.392815	-1.375571	-4.986667	49.58496	0.001706	0.017771
620722	RBM4		15.11742	NM 00289	Homo sapiens	2.070335	-0.905804	2.675579	1.328084	2.750157	1.533712	1.292341	-5.430222	1.328363	-5.467128	1.027874	-5.652757	2989.566	0.000239	0.003971
3610301	KDR		19.73005	NM 00225	Homo sapiens	1.084058	-6.547019	2.009797	-0.774602	2.748499	2.467268	1.853956	-1.263567	2.535379	1.907533	1.367551	-4.028252	194.7889	6.78E-05	0.001543
1110021	GPR3		27.08187	NM 00528	Homo sapiens	1.261567	-5.116403	2.154919	1.821182	2.74772	4.510929	1.708128	-0.831754	2.178021	2.134337	1.275092	-4.162814	502.0924	1.40E-05	0.000472
1400255	RPL34		24.37477	NM 03362	Homo sapiens	1.609282	-1.717465	2.211671	2.228767	2.746093	4.604673	1.374321	-3.815367	1.706409	-0.959929	1.241637	-4.416813	6407.596	2.38E-05	0.000701
7320731	LOC653400		17.63405	XM 92727	PREDICTED:	1.866184	-1.380746	2.625267	1.972391	2.745129	2.35899	1.406757	-4.413746	1.470986	-4.179432	1.045657	-5.626487	386.266	0.000116	0.002313
7650376	PCBD1		17.92027	NM 00100	Homo sapiens	2.406017	1.063662	2.725237	2.024778	2.74451	2.044122	1.132676	-6.209051	1.140686	-6.441863	1.007072	-5.664131	665.9604	0.000108	0.002182
4920327	ANTXR1		24.45749	NM 01815	Homo sapiens	1.277556	-5.05337	2.077589	1.088831	2.743894	4.181257	1.626222	-1.712477	2.147768	1.688829	1.32071	-3.871344	339.7116	2.34E-05	0.000694
6660097	QKI		23.88855	NM 20685	Homo sapiens	3.421024	4.100264	3.038492	2.933889	2.743756	1.954078	-1.125895	-6.242063	-1.24684	-5.880881	-1.107421	-5.482418	311.9538	2.64E-05	0.000759
3870746	ARFGEF1		29.93172	NM 00642	Homo sapiens	1.705478	-0.441029	2.494819	4.229351	2.741527	5.215456	1.462827	-2.610439	1.607483	-1.39004	1.098888	-5.370199	3053.821	8.37E-06	0.000326
4490341	CNOT7		22.22689	NM 05402	Homo sapiens	1.915063	-0.016614	2.500356	2.818258	2.741213	3.731981	1.305626	-4.766505	1.431396	-3.88567	1.096329	-5.452553	183.9374	3.78E-05	0.000986
3850255	CCNE1		30.71111	NM 00123	Homo sapiens	1.884646	0.78121	2.724914	4.95781	2.738428	4.975116	1.445849	-2.905635	1.453019	-3.057468	1.004959	-5.664235	716.1688	7.32E-06	0.000297
5810070	TPM4		30.19129	NM 00329	Homo sapiens	2.611427	3.887395	2.902458	4.799096	2.738341	4.180782	1.111445	-6.176445	1.048599	-6.71469	-1.059933	-5.570829	1091.642	8.00E-06	0.000315
1980196	FAM86B1		15.83938	NM 03291	Homo sapiens	1.577097	-3.231712	2.15823	0.065952	2.737739	2.47981	1.368483	-4.647639	1.735936	-2.189264	1.268511	-4.644948	266.2096	0.000192	0.003349
5050338	SLC39A3		12.84766	NM 21356	Homo sapiens	1.386607	-5.040263	2.049897	-1.417127	2.732487	1.352895	1.478355	-4.289217	1.970628	-1.580594	1.332987	-4.502949	298.2254	0.000498	0.006919
5360162	DTX2		15.00675	NM 02089	Homo sapiens	-1.113169	-6.554305	2.42896	-1.318256	2.731401	-0.394438	2.703842	-0.028789	3.04051	0.733231	1.124515	-5.52056	130.616	0.000247	0.004086
380468	CNIH		26.89832	NM 00577	Homo sapiens	2.191169	1.889997	2.82132	4.407478	2.728782	4.033216	1.287586	-4.825158	1.245354	-5.457208	-1.033912	-5.634582	1255.968	1.45E-05	0.000485
630711	RPLP0		29.34274	NM 00100	Homo sapiens	2.606022	3.625011	2.992037	4.831298	2.728371	3.886645	1.148124	-5.974113	1.046949	-6.722454	-1.096639	-5.442814	42464.16	9.27E-06	0.000353
3170053	LOC653722		20.33469	XM 93491	PREDICTED:	1.485316	-3.1978	1.965028	0.215272	2.72584	3.91104	1.32297	-4.537561	1.835192	-0.438066	1.387176	-3.401155	24.57929	5.85E-05	0.001376
160372	YWHAE		21.70755	NM 00676	Homo sapiens	2.385281	1.906772	2.696123	2.93777	2.724443	2.997639	1.130317	-6.151876	1.142189	-6.356112	1.010504	-5.662585	497.1349	4.25E-05	0.001085
7210307	MATN2		13.24734	NM 00238	Homo sapiens	3.179194	0.975567	3.149247	0.638707	2.7242	-0.561485	-1.009509	-6.513432	-1.167019	-6.516661	-1.156026	-5.453615	64.88566	0.000434	0.006233
7400392		40422	27.90506	NM 14471	Homo sapiens	2.597768	3.291623	3.04874	4.68051	2.720327	3.53629	1.1736	-5.840602	1.047179	-6.725696	-1.120726	-5.349498	574.0986	1.20E-05	0.000424
2760634	MYSM1		15.07434	XM 05548	PREDICTED:	1.439086	-4.395281	2.106495	-0.464555	2.720169	2.110871	1.463773	-4.059327	1.890206	-1.428158	1.291324	-4.567788	71.06845	0.000242	0.004017
1090678	MT1A		32.05703	NM 00594	Homo sapiens	1.509483	-1.706164	2.13084	3.187698	2.719967	6.049087	1.411635	-2.679199	1.801919	1.023009	1.276477	-3.698469	8678.4	5.86E-06	0.000253
5220767	RPS6KC1		21.34535	NM 01242	Homo sapiens	2.508892	1.503133	3.253616	3.656433	2.719526	1.990779	1.296834	-5.276049	1.083955	-6.653177	-1.196391	-5.108058	226.4071	4.61E-05	0.001156
1240477	RCHY1		20.61916	NM 01543	Homo sapiens	2.899715	1.514373	4.005006	3.831723	2.718705	0.651643	1.381172	-5.104525	-1.06658	-6.721599	-1.47313	-3.944987	192.7149	5.47E-05	0.001307
1770671	LOC645052		14.70354	XM 92810	PREDICTED:	1.176281	-6.385431	3.116138	0.690751	2.718179	-0.455807	2.649144	-0.210781	2.310825	-1.515219	-1.146406	-5.471168	43.50221	0.000271	0.004404
1820014	ASPSCR1		9.123727	NM 02408	Homo sapiens	1.783575	-3.349308	2.181852	-1.864038	2.717617	0.016195	1.223302	-6.002273	1.52369	-4.788553	1.245555	-5.123587	242.7566	0.002108	0.021025
7560477	RRBP1		23.13116	NM 00458	Homo sapiens	1.783247	-0.750948	2.50371	3.051951	2.717503	3.870586	1.404017	-3.796419	1.523907	-2.938256	1.085391	-5.48806	319.4693	3.10E-05	0.000852
7150601	C6orf66		21.75225	NM 01416	Homo sapiens	1.654643	-1.860768	2.39422	2.393818	2.714434	3.682266	1.446971	-3.48699	1.640495	-2.051475	1.133744	-5.272482	1251.434	4.20E-05	0.001078
60671	PCK2		20.79288	NM 00101	Homo sapiens	1.563071	-3.307206	2.841852	2.93434	2.714393	2.449238	1.81812	-1.412422	1.736576	-2.149876	-1.046957	-5.622624	169.4968	5.25E-05	0.001269
1070110	FLJ40432		13.88245	NM 15252	Homo sapiens	1.929722	-2.128895	2.896134	1.353751	2.714037	0.731891	1.500803	-4.376155	1.406439	-5.185333	-1.067094	-5.606299	429.2098	0.000352	0.005371
1690615	RHD		17.10815	NM 01612	Homo sapiens	3.700171	2.73372	3.113526	1.193552	2.711253	-0.003539	-1.188418	-6.134603	-1.364746	-5.618024	-1.148372	-5.443038	50.40923	0.000134	0.002576
670192	DDX19-DDX19L		24.93665	NM 00101	Homo sapiens	1.290381	-4.828967	2.023321	1.061166	2.709724	4.401598	1.568003	-1.997648	2.099941	1.724336	1.339246	-3.609582	172.2397	2.13E-05	0.000649
4810450	BDKRB2		32.58793	NM 00062	Homo sapiens	1.343376	-4.358678	2.832176	4.745327	2.70959	4.258813	2.108253	1.760977	2.017001	1.102055	-1.045241	-5.608376	43.71941	5.38E-06	0.000238
770523	UBE2L3		20.82906	NM 19815	Homo sapiens	1.6216	-2.305061	2.417236	2.230228	2.708049	3.376212	1.490648	-3.244193	1.669985	-2.00207	1.120308	-5.358312	169.2554	5.20E-05	0.001261
4830593	C6orf211		30.03675	NM 02457	Homo sapiens	2.047209	1.687041	2.800382	5.039005	2.704457	4.644727	1.367903	-3.796802	1.321046	-4.521235	-1.03547	-5.626948	593.9589	8.21E-06	0.000321
2970494	GAD1		29.50368	NM 01344	Homo sapiens	2.165976	2.516665	2.632976	4.554905	2.703288	4.798396	1.215608	-5.277205	1.24807	-5.217202	1.026704	-5.642556	810.9982	9.01E-06	0.000345
6060139	C7orf20		34.11987	XM 94607	PREDICTED:	1.64679	0.126465	2.123916	3.674126	2.702011	6.545334	1.289731	-3.917722	1.640775	0.003205	1.272184	-3.569696	1950.725	4.23E-06	0.000201
6130040	ZC3HAV1		27.27952	NM 02011	Homo sapiens	1.432581	-3.469403	2.499823	3.47521	2.700087	4.257803	1.744979	-0.594691	1.884771	0.260991	1.080111	-5.493946	3355.942	1.35E-05	0.000461

10370	C1GALT1	43.48463	NM	02015	Homo sapiens	1.678843	0.403981	2.791892	6.87166	2.697075	6.480394	1.662986	0.345816	1.606508	-0.380961	-1.035156	-5.611923	1659.005	1.16E-06	8.34E-05
6940376	CSNK1A1	13.04001	NM	00102	Homo sapiens	3.116869	1.164389	2.801762	0.046638	2.696016	-0.32644	-1.112467	-6.376126	-1.156102	-6.528851	-1.039223	-5.648623	79.29044	0.000466	0.00657
4830370	BAX	13.18877	NM	00432	Homo sapiens	1.672549	-2.796822	1.971178	-1.282011	2.695205	1.919214	1.178547	-5.987752	1.611436	-3.295683	1.367307	-4.13082	526.6499	0.000443	0.006329
270326	DKK4	26.1705	NM	01442	Homo sapiens	17.23677	3.899676	16.15685	3.508549	2.692081	-4.336349	-1.06684	-6.499621	-6.402768	-0.008212	-6.001621	-0.271753	376.6982	1.66E-05	0.000539
5420333	ZNF93	12.87135	NM	03121	Homo sapiens	2.008919	-1.556137	2.564871	0.502226	2.69201	0.895364	1.276742	-5.607479	1.340029	-5.508972	1.04957	-5.630583	482.5784	0.000493	0.006876
2000767	RNPC3	14.93023	NM	01761	Homo sapiens	3.180343	1.673315	2.992427	0.937221	2.691344	-0.00511	-1.062797	-6.465161	-1.181693	-6.420596	-1.111871	-5.531524	154.098	0.000253	0.004164
4260026	PAK1	37.05459	NM	00257	Homo sapiens	1.574033	-0.876441	2.490226	5.305861	2.690915	6.141429	1.582068	-0.679969	1.709567	0.386552	1.08059	-5.417789	3977.864	2.74E-06	0.000148
2120026	PPAP2A	32.61734	NM	00371	Homo sapiens	1.901791	0.444684	3.222191	6.016316	2.689415	4.223313	1.694293	-0.988401	1.414149	-3.731351	-1.198101	-4.788199	258.2739	5.35E-06	0.000237
6040008	IQGAP2	16.4084	NM	00663	Homo sapiens	1.281172	-5.517098	2.1589	-0.185629	2.68861	2.012146	1.685098	-2.502595	2.098555	-0.265533	1.245361	-4.831798	637.799	0.000163	0.002961
6130113	P18SRP	13.12933	NM	17382	Homo sapiens	2.345624	-0.406767	2.77125	0.804992	2.688083	0.484452	1.181455	-6.111517	1.145999	-6.510429	-1.030939	-5.65251	34.33635	0.000452	0.006417
2750494	CD3EAP	15.28523	NM	01209	Homo sapiens	1.280832	-5.448971	1.759559	-2.271577	2.686225	2.312687	1.373762	-4.602079	2.09725	-0.012512	1.526647	-2.9743	88.24789	0.000227	0.003811
2690646	PTK9	11.83108	XM	93693	PREDICTED:	2.010651	-2.111633	2.806433	0.562809	2.682403	0.121342	1.395783	-5.142177	1.334097	-5.720978	-1.046238	-5.639608	63.27355	0.000714	0.009091
6250544	YTHDC1	20.04948	NM	00103	Homo sapiens	2.191511	-0.1053	3.371468	3.641425	2.681542	1.562289	1.538422	-3.722604	1.223604	-6.046075	-1.257287	-4.835493	38.89306	6.27E-05	0.00145
3940739	PPIA	14.29931	NM	20343	Homo sapiens	3.027906	1.66898	2.565096	-0.009461	2.681487	0.331875	-1.180426	-6.126976	-1.129189	-6.572131	1.045375	-5.639287	694.4759	0.000308	0.004845
450750	PDZD4	21.70749	NM	03251	Homo sapiens	1.023833	-6.674956	1.740106	-1.89785	2.681313	3.022934	1.6996	-1.740946	2.618898	2.989907	1.54089	-2.561794	2006.497	4.25E-05	0.001085
10601	SRRM1L	24.24241	XM	93281	PREDICTED:	3.222697	3.64676	3.334039	3.764804	2.67974	1.75723	1.03455	-6.491508	-1.202616	-6.135571	-1.244165	-4.873573	1007.419	2.45E-05	0.000714
6900475	CDK8	37.89363	NM	00126	Homo sapiens	3.539831	6.329899	3.273535	5.627195	2.679237	3.6705	-1.081348	-6.337016	-1.321209	-4.824178	-1.221816	-4.699515	265.7638	2.43E-06	0.000138
160672	LOC644086	11.61172	XM	92946	PREDICTED:	1.055625	-6.646903	1.704606	-3.877811	2.678743	0.295366	1.614783	-3.866149	2.537589	0.090348	1.571473	-3.538826	48.66427	0.000774	0.009646
2000315	BMPR1A	45.01094	NM	00432	Homo sapiens	1.656462	0.189731	2.883407	7.207008	2.676169	6.398497	1.740702	1.058024	1.615593	-0.284819	-1.077438	-5.422055	2682.804	9.67E-07	7.44E-05
3390554	DAZL	10.20397	NM	00135	Homo sapiens	3.855052	-0.529216	4.632225	0.220415	2.675954	-3.081422	1.201599	-6.322099	-1.440627	-6.050728	-1.731056	-4.298623	80.00527	0.001336	0.014628
4050487	LOC399753	11.87323	XM	93072	PREDICTED:	1.926523	-2.206545	2.568412	0.202759	2.675726	0.519234	1.333186	-5.368551	1.388889	-5.313576	1.041782	-5.642063	92.19713	0.000703	0.008986
3990356	FGFR4	12.94514	NM	02296	Homo sapiens	1.584076	-3.532517	2.0835	-0.877419	2.67477	1.601391	1.315277	-5.210782	1.688536	-2.922122	1.283787	-4.680155	319.8991	0.000481	0.006741
2810619	CLDN10	14.19413	NM	00698	Homo sapiens	1.687693	-3.140022	2.643724	1.053365	2.671445	1.09971	1.566472	-3.745586	1.582898	-3.879211	1.010486	-5.663328	1978.32	0.000318	0.004983
5310152	PHF17	44.4934	NM	19932	Homo sapiens	1.870006	2.487074	2.693939	6.996957	2.671176	6.883007	1.440605	-1.743367	1.428432	-2.104216	-1.008522	-5.661498	576.8297	1.03E-06	7.76E-05
1050192	STEAP1	24.09771	NM	01244	Homo sapiens	2.044719	0.956102	2.674713	3.731164	2.67038	3.673782	1.308108	-4.680653	1.305989	-4.951006	-1.001623	-5.664964	853.4593	2.52E-05	0.000733
4120041	C1orf116	22.68832	NM	02393	Homo sapiens	1.105994	-6.42361	2.054085	0.269629	2.667655	3.112862	1.85723	-0.552317	2.411998	2.280268	1.298707	-4.250144	36.95537	3.41E-05	0.000915
4010288	SPANXC	11.6432	NM	02266	Homo sapiens	1.073856	-6.611143	1.331654	-5.910401	2.667616	0.539128	1.240068	-5.839895	2.484147	0.164302	2.003235	-1.491965	50.81175	0.000765	0.009556
5390220	MYO6	21.42428	NM	00499	Homo sapiens	2.831495	2.541826	3.168071	3.333596	2.665917	1.712066	1.118869	-6.267886	-1.062109	-6.71238	-1.188361	-5.156124	220.3392	4.53E-05	0.001142
2650717	LOC645015	18.56549	XM	92807	PREDICTED:	1.335519	-4.716893	1.865317	-0.768797	2.661444	3.272231	1.396699	-4.022945	1.992817	0.278703	1.426805	-3.239937	226.7621	9.09E-05	0.001926
7560131	CTSC	32.08239	NM	00181	Homo sapiens	1.810077	0.286929	2.862088	5.531358	2.660541	4.742491	1.581197	-1.541556	1.46985	-2.845073	-1.075754	-5.491318	5613.207	5.83E-06	0.000252
5690626	ZSWIM4	12.89841	NM	02307	Homo sapiens	1.724017	-2.87711	2.398661	0.192159	2.65742	1.120563	1.391321	-4.839328	1.541412	-4.112253	1.107876	-5.500519	263.5841	0.000489	0.006826
6760253	LOC348180	18.22825	XM	93918	PREDICTED:	-1.035293	-6.671079	2.578409	0.205358	2.654054	0.412975	2.669408	0.870484	2.747722	0.980266	1.029338	-5.653645	76.2813	9.92E-05	0.002055
1710093	SQLE	23.42729	NM	00312	Homo sapiens	2.411079	2.381658	2.738709	3.488263	2.653534	3.126804	1.135885	-6.091936	1.100559	-6.540237	-1.032099	-5.640839	721.6695	2.91E-05	0.000815
3290402	PNMT	20.6685	NM	00268	Homo sapiens	1.650774	-2.584223	2.871228	3.170761	2.652086	2.357504	1.739322	-1.831901	1.606571	-2.989467	-1.08263	-5.535577	289.1406	5.40E-05	0.001296
150600	PKMYT1	18.58625	NM	18268	Homo sapiens	1.5783	-2.661335	2.184133	1.081897	2.651813	3.152527	1.383852	-4.167681	1.68017	-1.930163	1.214126	-4.82001	1139.531	9.04E-05	0.00192
4280754	CXorf56	21.52043	NM	02210	Homo sapiens	1.89341	0.112201	2.338044	2.404444	2.649884	3.72574	1.234832	-5.291338	1.39953	-4.027605	1.133377	-5.252302	204.7518	4.43E-05	0.001121
5670301	37681	22.43841	NM	17845	Homo sapiens	1.334149	-4.729754	2.375071	2.104245	2.646933	3.213441	1.780215	-0.940971	1.983986	0.222717	1.114465	-5.381329	450.8135	3.60E-05	0.000955
4610477	LARP2	10.93079	NM	17804	Homo sapiens	1.297748	-5.67388	1.854961	-2.736694	2.645238	0.669161	1.429369	-4.739737	2.038329	-1.538014	1.426034	-4.105759	145.2113	0.001001	0.011706
5670468	COL6A2	11.45694	NM	00184	Homo sapiens	1.450232	-4.657669	2.011357	-1.672707	2.640702	0.962925	1.386921	-4.898428	1.820883	-2.45348	1.312896	-4.625731	1380.129	0.00082	0.010092
6760255	CYP1B1	21.71248	NM	00010	Homo sapiens	2.279919	0.505803	3.469631	4.137478	2.640065	1.651716	1.521822	-3.722757	1.157965	-6.363557	-1.314221	-4.468777	402.8462	4.24E-05	0.001085
5860500	EIF2C3	13.25257	NM	02485	Homo sapiens	1.592508	-3.548414	2.285985	-0.028122	2.637237	1.330034	1.435462	-4.451147	1.656027	-3.215211	1.153655	-5.330304	123.6964	0.000433	0.006226
3930112	LOC400197	33.06573	XM	92885	PREDICTED:	1.589274	-0.424903	2.094912	3.536224	2.635294	6.322196	1.318157	-3.529555	1.658174	0.215861	1.25795	-3.711924	249.9904	4.98E-06	0.000226
1440372	PRPS1L1	14.51024	NM	17588	Homo sapiens	1.149047	-6.391172	2.379966	-0.372715	2.634371	0.516451	2.071252	-1.27701	2.292658	-0.49646	1.106894	-5.520878	109.5618	0.000288	0.004625
7570477	DDX25	13.98401	NM	01326	Homo sapiens	1.118808	-6.477113	2.045317	-1.55744	2.628981	0.850716	1.828122	-2.246656	2.349806	0.056526	1.285366	-4.780755	49.25561	0.000341	0.00523
5890497	TIAL1	29.91768	XM	94555	PREDICTED:	2.427365	3.603229	2.755874	4.797905	2.625674	4.25443	1.135336	-5.984154	1.081697	-6.574628	-1.049587	-5.592933	1639.781	8.39E-06	0.000326
3310170	UBE2G1	26.25306	NM	00334	Homo sapiens	1.927743	0.760862	2.62576	4.181069	2.624766	4.138566	1.36209	-3.934149	1.361574	-4.180675	-1.000379	-5.66503	1336.725	1.64E-05	0.000532
2350110	PRMT1	9.787973	NM	19831	Homo sapiens	1.467454	-4.640891	1.675878	-3.719081	2.623659	0.665716	1.142031	-6.231797	1.787899	-2.800453	1.565543	-3.343479	34.97441	0.001586	0.016817
4540240	C21orf70	23.16125	NM	05819	Homo sapiens	1.643372	-1.179077	2.101997	1.918517	2.619605	4.45743	1.279076	-4.652703	1.594043	-1.7006	1.246246	-4.300877	475.0915	3.08E-05	0.000848
5490161	LOC642904	30.07718	XR	00053	PREDICTED:	2.713293	3.623255	3.37449	5.487915	2.616121	3.037159	1.243688	-5.338917	-1.037144	-6.748559	-1.289883	-4.284038	840.1272	8.16E-06	0.000319
6330332	MLL3	15.241	NM	17060	Homo sapiens	4.175963	2.364953	3.36352	0.634583	2.615682	-1.328749	-1.241546	-6.055622	-1.59651	-4.865614	-1.285906	-5.115841	119.1453	0.00023	0.00385
2000167	CABLES1	36.77254	NM	13837	Homo sapiens	1.135138	-5.94959	1.893303	1.910821	2.614933	6.029804	1.667906	0.30949	2.303627	4.683465	1.381149	-2.452589	876.1669	2.85E-06	0.000152
5900332	LOH11CR2A	15.30477	NM	19831	Homo sapiens	2.747329	0.414993	3.566905	2.22249	2.612801	-0.346461	1.298317	-5.682802	-1.051488	-6.751874	-1.365165	-4.636803	77.1687	0.000226	0.003797
5900592	SYNJ2	20.24368	NM	00389	Homo sapiens	1.399875	-4.130548	2.236328	1.472777	2.612593	3.127643	1.59752	-2.290207	1.866305	-0.492702	1.168252	-5.092156	661.8436	5.98E-05	0.001398
870554	CAPN11	14.30756	NM	00705	Homo sapiens	1.61205	-2.975999	2.106226	-0.20363	2.609693	2.010943	1.306551	-5.10362	1.618866	-3.008708	1.239037	-4.821019	150.1443	0.000307	0.004837
2760332	LOC653479	20.15808	XM	93017	PREDICTED:	2.198661	0.80419	2.83027	3.144519	2.60738	2.294476	1.28727	-5.169657	1.185895	-6.128799	-1.085484	-5.523918	689.7228	6.10E-05	0.001419
110538	LOC642432	44.21014	XM	37089	PREDICTED:	4.413814	6.771066	4.586913	7.051784	2.606731	2.233293	1.039218	-6.480065	-1.693237	-2.328411	-1.759642	-1.489534	428.7409	1.07E-06	7.94E-05
2630397	MRE11A	9.520916	NM	00559	Homo sapiens	1.966727	-1.92234	1.76687	-3.319476	2.604527	0.388276	-1.113114	-6.337473	1.324295	-5.649961	1.474091	-3.899275	54.54868	0.001775	0.018353
3130431	MATN3	14.91963	NM	00238	Homo sapiens	3.925076	2.046869	3.444141	0.892083	2.601995	-1.297256	-1.139638	-6.339689	-1.508487	-5.227254	-1.323654	-4.97865	221.7828	0.000254	0.004175
4050735	PSPH	19.73905	NM	00457	Homo sapiens	2.231628	1.354149	2.500718	2.342317	2.601443	2.707223	1.12058	-6.188933	1.165715	-6.201183	1.040278	-5.628896	877.4248	6.76E-05	0.00154
4670291	VPS25	39.88788	NM	03235	Homo sapiens	1.879967	2.422862	2.569228	6.307382	2.598724	6.410721	1.366634	-2.782195	1.382324	-2.799287	1.01148	-5.65883	1789.353	1.85E-06	0.000113
4210474	RASAL1	12.23564	NM	00465	Homo sapiens	1.428013	-4.910126	2.377441	-0.277029	2.598465	0.503887	1.664859	-3.325833	1.819637	-2.672306	1.092967	-5.551365	44.86584	0.000617	0.008148
110441	0	13.00819	AV726540		AV726540 HT	2.488423	-1.179073	3.829461	1.774723	2.595479	-1.21151	1.538911	-4.797644	1.043021	-6.765961	-1.475435	-4.388646	55.61597	0.000471	0.006624
6280161	TRIM23	19.75428	NM	00165	Homo sapiens	2.595203	2.112408	2.819217	2.683048	2.594631	1.832983	1.086319	-6.367875	-1.000221	-6.785032	-1.086558	-5.532316	250.2288	6.74E-05	0.001536
6200647	PARVB	10.60732	NM	01332	Homo sapiens	1.481315	-4.515885	2.000544	-1.840462	2.593994	0.655922	1.350519	-5.162504	1.751142	-2.948962	1.296644	-4.737097	284.4141	0.001137	0.012938
3840347	ALDH1B1	20.66186	NM	00069	Homo sapiens	1.818029	-0.930751	2.64389	3.060044	2.593514	2.818469	1.454262	-3.608764	1.426553	-4.084247	-1.019424	-5.656182	678.7514	5.41E-05	0.001297
6110121	PRICKLE1	23.23748	NM	15302	Homo sapiens	1.730443	-1.052992	2.577479	3.490264	2.589935	3.499828	1.489491	-2.95182	1.496689	-3.112302	1.004833	-5.664395	2322.21	3.03E-05	0.000838
6660162	LOC641949	17.49008	XM	93571	PREDICTED:	1.629321	-2.899433	2.656058	2.151565	2.589282	1.852591	1.630163	-2.751874	1.589178	-3.270669	-1.02579	-5.652292	32.77951	0.000121	0.002376
5050241	PRKCQ	21.27171	NM	00625	Homo sapiens	1.163477	-6.062164	1.958727	-0.000354	2.588522	3.167695	1.683511	-1.522593	2.224816	1.731967	1.321533	-3.982445	676.727	4.69E-05	0.00117
360398	SLC13A5	16.63672	NM	17755	Homo sapiens	1.80301	-1.914055	2.730335	2.150754	2.588416	1.586161	1.514321	-3.715004	1.435609	-4.526064	-1.054828	-5.612286	69.04363	0.000153	0.002825
3310053	KIAA1727	23.01178	NM	03339	Homo sapiens	1.393259	-3.6976	2.042675	1.397784	2.586108	4.148149	1.466113	-2.817864	1.856158	0.338872	1.26604	-4.170504	549.6297	3.18E-05	0.000869
670494	TOM1L1	25.13542	NM	00548	Homo sapiens	2.122572	1.571642	2.744943	4.192809	2.585185	3.529492	1.293215	-4.756017	1.217949	-5.679867	-1.061798	-5.566085	223.0034	2.04E-05	0.000629
1500338	ADK	14.27435	NM	00672	Homo sapiens	2.838015	1.251951	2.776232	0.804499	2.58229	0.108894	-1.022254	-6.507226	-1.09903	-6.652157	-1.075105	-5.594946	102.6035	0.00031	0.004873
870400	NQO2	36.30125	NM	00090	Homo sapiens	1.554737	-0.840028	2.406398	5.244789	2.579362	6.020639	1.547785	-0.801479	1.659034	0.176829	1.071877	-5.452715	2617.899	3.05E-06	0.000161
4060053	LOC91664	20.58655	XM	93761	PREDICTED:	1.949304	0.226358	2.464255	2.690718	2.576996	3.124688	1.264172	-5.114685	1.322008	-4.863351	1.045751	-5.613642	845.7438	5.51E-05	0.001315
20762	LIMS1	11.60666	NM	00498	Homo sapiens	1.695067	-2.851303	2.043403	-1.156728	2.573855	1.146944	1.2055	-5.884514	1.518439	-4.108759	1.259592	-4.827052	244.0067	0.000775	0.009657
130717	FAM3C	34.50208	NM	01488	Homo sapiens	2.151197	3.204944	2.740823	5.840967	2.572952	5.12564	1.274092	-4.42792	1.196056	-5.545508	-1.065244	-5.514584	996.6954	3.99E-06	0.000193
4480576	STEAP3	25.38835	NM	01823	Homo sapiens	1.487085	-2.440239	2.139132	2.485615	2.571603	4.629359	1.438473	-2.810307	1.729291	-0.228354	1.202171	-4.60573	445.0682	1.94E-05	0.000606

6350414	CICE		24.05894	XR	000206	PREDICTED:	2.256654	1.934458	2.796529	3.992573	2.57015	3.087405	1.239237	-5.323698	1.138921	-6.315251	-1.08808	-5.484705	645.3634	2.55E-05	0.000738
7400592	LOC654053		23.12218	XM	94367	PREDICTED:	1.411325	-3.952863	2.516179	2.947325	2.569973	3.128651	1.782848	-0.759901	1.820964	-0.670416	1.021379	-5.653422	693.6502	3.10E-05	0.000853
2230014	TAF1A		12.52822	NM	00568	Homo sapiens	4.243822	1.272144	3.716487	0.16994	2.56872	-2.407061	-1.141891	-6.378507	-1.652116	-5.06099	-1.446824	-4.786399	40.90669	0.000556	0.007516
2450066	TAF9		17.0762	NM	01628	Homo sapiens	1.956191	-1.242492	2.984414	2.658755	2.567734	1.208411	1.525625	-3.779463	1.31262	-5.485499	-1.162275	-5.285194	998.7636	0.000135	0.002589
2120767	BAG2		16.43986	NM	00428	Homo sapiens	-1.060445	-6.633433	2.180063	-1.100346	2.566016	0.407789	2.311837	-0.128658	2.721119	1.199067	1.177038	-5.291004	103.6929	0.000162	0.00294
3940612	WTAP		28.5307	NM	00490	Homo sapiens	2.527603	4.145029	2.491866	3.839602	2.565937	4.122946	-1.014341	-6.507324	1.015166	-6.776995	1.029725	-5.637895	658.0159	1.07E-05	0.000389
1850068	LOC652672		28.88064	XM	94225	PREDICTED:	2.227059	2.86827	2.667456	4.716774	2.564576	4.256413	1.197748	-5.438292	1.151552	-6.098094	-1.040116	-5.614874	1702.512	1.01E-05	0.000374
6510041	AP3S1		23.21835	NM	00128	Homo sapiens	2.936181	3.387755	3.023963	3.47603	2.564471	1.854396	1.029897	-6.495146	-1.144946	-6.386073	-1.179176	-5.146921	1925.642	3.04E-05	0.00084
3460368	RNF125		9.871375	NM	01783	Homo sapiens	1.816074	-3.764608	3.1412	0.239122	2.561686	-1.399098	1.729665	-3.985909	1.410562	-5.648679	-1.226224	-5.288045	51.6434	0.001532	0.01641
520280	C14orf120		16.36302	XM	94515	PREDICTED:	1.937339	-1.076114	2.699134	2.090581	2.561043	1.529715	1.393218	-4.567397	1.321938	-5.329566	-1.05392	-5.613434	1295.481	0.000165	0.002992
2900468	ADAM17		18.06233	NM	00318	Homo sapiens	3.051319	2.544016	2.901475	1.889381	2.559029	0.687533	-1.051644	-6.470571	-1.192374	-6.264739	-1.133819	-5.423874	64.8009	0.000104	0.002122
940273	TM2D3		14.97932	NM	07847	Homo sapiens	3.543444	1.886949	3.289609	1.093081	2.558852	-0.969202	-1.077163	-6.450137	-1.384779	-5.644471	-1.28558	-5.039785	70.64179	0.000249	0.004113
6760487	FXYD5		14.93409	NM	01416	Homo sapiens	1.308835	-5.257233	2.030874	-0.631258	2.557965	1.789842	1.551666	-3.282587	1.954383	-0.832405	1.259539	-4.702997	2855.691	0.000253	0.004162
1240615	CR2		13.80598	NM	00187	Homo sapiens	1.189312	-6.312415	2.88633	0.472612	2.557298	-0.602522	2.426891	-0.585402	2.150234	-1.826925	-1.128664	-5.49814	25.39612	0.000361	0.005465
2750114	ZDHHC22		27.89165	NM	17497	Homo sapiens	-1.451696	-4.079009	1.53429	-3.788708	2.555892	2.018149	2.227322	0.95987	3.710378	5.51691	1.665847	-2.026979	147.9734	1.20E-05	0.000424
6480390	HIATL2		12.07273	NM	03231	Homo sapiens	1.765538	-2.293264	2.091852	-0.757872	2.551127	1.233963	1.184824	-5.971819	1.444957	-4.54419	1.219554	-5.009087	283.9436	0.000654	0.008528
6130519		0	30.43628	AK026659		Homo sapiens	1.178451	-5.727012	2.201135	2.814427	2.544975	4.481074	1.867821	0.982224	2.159594	2.743749	1.15621	-4.984613	656.7398	7.67E-06	0.000307
4180369	RIS1		38.56275	NM	01544	Homo sapiens	1.620384	-0.08096	2.57755	6.128315	2.543178	5.948364	1.590703	-0.283932	1.569491	-0.685482	-1.013515	-5.656794	8600.304	2.21E-06	0.000129
5910609	LOC652595		21.52357	XM	94211	PREDICTED:	2.157823	1.335467	2.61539	3.208722	2.540108	2.859095	1.21205	-5.558343	1.177163	-6.075699	-1.029637	-5.643558	3824.437	4.43E-05	0.00112
5820189	AGMAT		10.67352	NM	02475	Homo sapiens	1.30974	-5.465772	1.513127	-4.480996	2.539982	0.888993	1.155288	-6.141411	1.939303	-1.575349	1.678632	-2.504655	101.8304	0.001107	0.01267
6770563	MAPK8IP1		12.19878	NM	00545	Homo sapiens	1.248725	-5.915248	2.159276	-1.102603	2.538708	0.413538	1.729185	-2.877802	2.033041	-1.463371	1.175722	-5.287244	102.3384	0.000625	0.008238
770010	C21orf56		14.72194	NM	03226	Homo sapiens	1.760872	-1.960073	2.333762	0.891132	2.534496	1.694892	1.325344	-4.973543	1.439341	-4.365852	1.086013	-5.531209	986.7367	0.00027	0.00438
5890537	SLC35F2		17.58947	NM	01751	Homo sapiens	1.625506	-2.774819	2.603909	2.209993	2.531032	1.875434	1.601907	-2.8065	1.557073	-3.368499	-1.028793	-5.648337	823.7476	0.000118	0.002334
4050719	PH-4		14.85837	NM	01773	Homo sapiens	1.281174	-5.479642	2.073384	-0.487318	2.529575	1.573956	1.618347	-2.86117	1.97442	-0.80115	1.220023	-4.947957	28.82037	0.000258	0.004239
2710403	LOC647958		38.35953	XM	93701	PREDICTED:	3.358578	6.997076	2.499228	4.077257	2.528865	4.169791	-1.343846	-3.943591	-1.328097	-4.353326	1.011859	-5.660448	75.72347	2.28E-06	0.000131
940592	SLC30A6		12.67497	NM	01796	Homo sapiens	2.190611	-2.258173	3.884676	1.792152	2.528072	-1.488957	1.77333	-3.787221	1.154049	-6.571556	-1.536616	-4.170553	61.86882	0.000528	0.007233
5220338	KATNA1		23.39667	NM	00704	Homo sapiens	1.573561	-1.573933	2.066152	2.034278	2.527098	4.432399	1.313042	-4.167982	1.605975	-1.336889	1.223094	-4.422933	496.4999	2.93E-05	0.000818
630239	NOLC1		21.33515	NM	00474	Homo sapiens	1.705741	-1.20421	2.416651	2.8474	2.5262	3.290856	1.416775	-3.594218	1.480999	-3.225583	1.045331	-5.610607	855.7195	4.62E-05	0.001156
7400255	PHLDB2		49.39768	NM	14575	Homo sapiens	2.85335	6.197248	3.583838	8.279646	2.523138	4.814334	1.256011	-4.661723	-1.130874	-6.179425	-1.420389	-2.423437	670.6549	5.84E-07	5.34E-05
6620608	MAPK10		11.64953	NM	00275	Homo sapiens	-1.244267	-6.216416	2.038581	-3.252923	2.520985	-1.604299	2.536539	-1.063974	3.136778	0.35782	1.236637	-5.265486	72.84541	0.000763	0.00954
1470020	CEP76		27.26076	NM	02489	Homo sapiens	2.03445	2.21883	2.365594	3.888859	2.520918	4.583828	1.162768	-5.667615	1.239115	-5.163112	1.06566	-5.52146	213.7021	1.35E-05	0.000462
5550114	S100PBP		22.08368	NM	00101	Homo sapiens	2.28162	1.65339	2.835574	3.675428	2.51838	2.433056	1.24279	-5.38988	1.103769	-6.533124	-1.125951	-5.343899	375.4567	3.90E-05	0.001012
4880400	VSNL1		13.46075	NM	00338	Homo sapiens	-1.875871	-3.918453	1.165382	-6.872549	2.51614	-2.161184	2.186106	-2.667954	4.719954	2.399392	2.159069	-2.392171	957.4753	0.000404	0.005938
2970554	YME1L1		24.88621	NM	13931	Homo sapiens	1.996265	1.361443	2.50142	3.833723	2.516092	3.858465	1.25305	-4.957792	1.2604	-5.139587	1.005866	-5.66396	2089.388	2.15E-05	0.000653
6040349	RABL3		33.02313	NM	17382	Homo sapiens	1.551247	-0.730586	2.188693	4.262226	2.512687	5.930851	1.410925	-2.267943	1.619785	-0.059621	1.148031	-4.844123	522.3964	5.02E-06	0.000227
7330112	ZNF24		16.386	NM	00696	Homo sapiens	2.155329	0.411529	2.480177	1.633166	2.512429	1.716887	1.150719	-6.084183	1.165682	-6.270407	1.013004	-5.661632	196.1092	0.000164	0.002974
5270402	FLJ20280		18.31268	NM	01774	Homo sapiens	2.62073	2.320558	2.510917	1.669488	2.512212	1.62596	-1.043734	-6.473933	-1.043196	-6.745117	1.000516	-5.665029	670.4511	9.71E-05	0.002024
6480632	LOC401703		9.199363	XM	94000	PREDICTED:	1.685777	-3.319848	1.921544	-2.340664	2.510566	0.23632	1.139856	-6.24086	1.489263	-4.602549	1.306536	-4.709896	223.634	0.00204	0.020509
2640243	C1orf108		9.699568	NM	02459	Homo sapiens	2.682453	-1.195326	3.337878	0.078401	2.50996	-2.068179	1.244338	-6.095875	-1.068723	-6.744749	-1.329853	-5.04121	37.22878	0.001646	0.017302
6060730	GALNACT-2		13.28736	NM	01859	Homo sapiens	2.994671	1.29508	2.730968	0.265303	2.505328	-0.540877	-1.09656	-6.399708	-1.195321	-6.361808	-1.090064	-5.574441	58.48089	0.000428	0.006197
4050192	C10orf125		18.44671	NM	19847	Homo sapiens	1.463831	-3.294438	1.991754	0.553512	2.503039	3.205707	1.360645	-4.1171	1.709924	-1.232385	1.256701	-4.386607	621.4522	9.37E-05	0.001972
4250097	DNAJB12		16.90585	XM	94453	PREDICTED:	-1.014614	-6.685439	-1.168746	-6.636363	2.501964	0.760072	-1.151912	-6.154595	2.538529	1.155871	2.924161	1.672817	22.78172	0.000142	0.002677
3610167	M6PR		31.71989	NM	00235	Homo sapiens	1.834074	1.231487	2.560474	5.291742	2.499514	4.985868	1.396058	-2.926533	1.362821	-3.547392	-1.024389	-5.642085	3017.099	6.19E-06	0.000264
2140452	TMEM63A		16.90904	NM	01469	Homo sapiens	1.099597	-6.486639	1.963627	-0.908705	2.498289	1.679096	1.78577	-1.576616	2.272005	0.936486	1.272283	-4.599718	1143.885	0.000142	0.002677
3370273	NHP2L1		14.19051	NM	00100	Homo sapiens	1.824715	-1.552286	2.294561	0.705279	2.498177	1.541986	1.25749	-5.45387	1.369078	-4.903246	1.088739	-5.523257	1306.678	0.000319	0.004987
5820270	PHF5A		13.97591	NM	03275	Homo sapiens	1.698029	-2.524752	2.354241	0.776042	2.49716	1.329071	1.386456	-4.597715	1.470623	-4.22559	1.060707	-5.599406	981.4598	0.000341	0.005237
6280474	DDX21		28.73124	NM	00472	Homo sapiens	1.998433	1.579016	2.759498	5.120122	2.493352	3.992509	1.380831	-3.562849	1.247653	-5.20193	-1.106742	-5.337564	8437.831	1.03E-05	0.00038
4200411	ZMYND11		15.22152	NM	21247	Homo sapiens	1.776902	-1.621756	2.301994	1.054226	2.492927	1.850541	1.29551	-5.104158	1.402962	-4.515975	1.082942	-5.53134	65.97301	0.000231	0.003869
3310615	ZFX		14.86288	NM	00341	Homo sapiens	2.617116	1.856796	1.959649	-1.398878	2.490493	1.081517	-1.335502	-5.028356	-1.050843	-6.735343	1.270887	-4.719076	125.2092	0.000258	0.004237
3520093	CKMT1B		13.91133	NM	02099	Homo sapiens	1.139501	-6.39804	2.215155	-0.667701	2.490404	0.430578	1.943969	-1.558382	2.185521	-0.570056	1.124257	-5.454544	1267.124	0.000349	0.005324
1110167	C2orf28		21.75128	NM	01608	Homo sapiens	2.196567	1.870031	2.47005	2.968409	2.489613	3.01068	1.124505	-6.11298	1.133411	-6.325451	1.00792	-5.663344	517.1848	4.20E-05	0.001078
3840382	LOC649150		13.3212	XM	94079	PREDICTED:	2.885848	0.408122	3.377297	1.355759	2.486448	-1.156661	1.170296	-6.227843	-1.160631	-6.524828	-1.358282	-4.750792	258.3494	0.000424	0.006149
3190156	SNRPD2		21.41767	NM	17754	Homo sapiens	1.264705	-5.088682	1.996181	0.850578	2.483918	3.425994	1.578377	-1.930886	1.964029	0.849135	1.244335	-4.417586	238.7263	4.54E-05	0.001143
3140427	PTK7		19.28245	NM	15288	Homo sapiens	2.511521	1.873783	2.829219	2.80167	2.482992	1.471304	1.126496	-6.209734	-1.01149	-6.782133	-1.13944	-5.337568	1231.368	7.57E-05	0.00168
1090730	KCR1		13.82604	NM	00101	Homo sapiens	1.925541	-0.827078	2.14795	0.113975	2.481937	1.613483	1.115505	-6.248803	1.288956	-5.459102	1.155491	-5.254297	84.76593	0.000359	0.005438
4860129	TCF20		14.23323	XM	93547	PREDICTED:	2.361664	-0.326477	3.142876	1.895501	2.474378	-0.245712	1.330788	-5.394618	1.047726	-6.752111	-1.270168	-4.943549	48.03047	0.000314	0.004924
2260133	GK		17.91558	NM	00016	Homo sapiens	1.607769	-3.027064	2.778882	2.634476	2.473553	1.435218	1.728409	-2.05172	1.5385	-3.619975	-1.123438	-5.402805	71.73822	0.000108	0.002182
2060327		37681	17.90214	NM	17845	Homo sapiens	1.330681	-5.877555	4.506656	2.82508	2.471023	-1.646537	3.386729	1.185152	1.856961	-3.647245	-1.823802	-3.064285	36.32625	0.000108	0.002187
2690709	SCAMP1		22.42089	NM	00486	Homo sapiens	2.903327	2.819236	3.332229	3.824968	2.470811	1.02034	1.147728	-6.142831	-1.17505	-6.276383	-1.348638	-4.257222	82.82455	3.62E-05	0.000958
4050333	LAMC3		19.40413	NM	00605	Homo sapiens	1.376798	-4.633755	2.620803	2.413479	2.470231	1.760715	1.903549	-0.690241	1.794185	-1.576417	-1.060955	-5.590792	862.5094	7.35E-05	0.001645
360209	CTTNBP2NL		17.52011	NM	01870	Homo sapiens	2.782514	2.164329	2.777536	1.920334	2.466985	0.735291	-1.001792	-6.514391	-1.127901	-6.513158	-1.125883	-5.429659	259.2456	0.00012	0.002364
1510008	TAPBP		23.63198	NM	00319	Homo sapiens	1.167796	-5.865471	1.850925	0.275511	2.46421	3.846437	1.584973	-1.541223	2.110138	2.208265	1.33134	-3.522401	896.2632	2.78E-05	0.000792
1070730	C14orf101		11.02206	NM	01779	Homo sapiens	2.363825	-1.833378	3.617133	1.043012	2.461191	-1.90029	1.530203	-4.939489	1.04119	-6.768731	-1.469668	-4.488563	83.75307	0.000967	0.011399
6040048	DPP10		11.77088	NM	00100	Homo sapiens	1.814206	-2.508189	2.535019	0.49239	2.460888	0.158497	1.397316	-4.878875	1.356455	-5.383384	-1.030124	-5.651744	143.0405	0.00073	0.009235
4540538	HRB2		32.9602	NM	00704	Homo sapiens	1.519713	-1.517581	2.479762	5.170564	2.458953	5.039305	1.63173	-0.249824	1.618037	-0.558141	-1.008462	-5.662089	185.4781	5.07E-06	0.000228
4120113	FEZ1		11.90819	NM	02254	Homo sapiens	2.953526	-0.050382	3.495544	0.903066	2.454757	-1.83969	1.183516	-6.232693	-1.203185	-6.443829	-1.423988	-4.63491	917.3563	0.000694	0.008915
3800072	RAB20		11.36048	NM	01781	Homo sapiens	1.706248	-2.943399	2.306349	-0.133211	2.454273	0.427808	1.351708	-5.070317	1.438403	-4.760683	1.064138	-5.602917	887.2924	0.00085	0.010344
3850059	TCEAL8		32.90368	NM	15333	Homo sapiens	1.625853	0.161433	2.238364	4.67366	2.454121	5.778288	1.376733	-2.635274	1.509436	-1.193242	1.09639	-5.274816	2052.096	5.11E-06	0.00023
7320288	MGC15875		10.705	NM	15337	Homo sapiens	2.38652	0.102956	1.770532	-3.170026	2.452924	0.023681	-1.347911	-5.207044	1.027825	-6.772614	1.385417	-4.303023	72.90898	0.001094	0.012546
2760408	LOC387921		21.06245	NM	00101	Homo sapiens	1.275319	-5.288182	2.465332	2.305222	2.451706	2.19927	1.933111	-0.097251	1.922426	-0.332885	-1.005558	-5.664303	78.25572	4.93E-05	0.001212
4050328	PACSIN3		27.05691	NM	01622	Homo sapiens	1.41134	-2.729175	1.979092	2.341009	2.450977	5.055591	1.402279	-2.697165	1.736631	0.604642	1.238435	-4.04487	1816.58	1.41E-05	0.000473
2100020	ZD52F10		18.39638	NM	03331	Homo sapiens	1.48921	-2.915393	1.971813	0.650927	2.450019	3.227406	1.324067	-4.370193	1.645181	-1.582182	1.242521	-4.442125	2463.973	9.50E-05	0.001992
1780041	IQCB1		16.09138	NM	00102	Homo sapiens	2.307675	-0.521115	3.51238	2.81009	2.44794	-0.325884	1.522043	-4.301746	1.060782	-6.732141	-1.434831	-4.146648	54.38139	0.000179	0.003173
4730647	ARHGEF6		28.90417	NM	00484	Homo sapiens	1.254567	-5.010316	2.388094	3.647763	2.446277	3.884448	1.90352	1.110568	1.949897	1.299584	1.024364	-5.645606	473.2932	1.00E-05	0.000373
5130180	VENTX		11.84545	NM	01446	Homo sapiens	1.681963	-2.681549	2.084918	-0.592254	2.442368	1.029092	1.239574	-5.625294	1.452094	-4.395397	1.171446	-5.217143	1807.437	0.00071	0.009062
6770075	JAZF1		15.82068	NM	17506	Homo sapiens	1.066811	-6.588597	1.67222	-2.626665	2.436516	1.703193	1.567494	-2.935201	2.283925	1.258518	1.457055	-3.274935	300.5071	0.000193	0.003361
3310136	KIAA0971		29.48958	NM	01492	Homo sapiens	1.533633	-1.299768	2.252257	4.10752	2.436088	5.027789	1.468576	-1.898649	1.588443	-0.787892	1.081621	-5.411295	573.5763	9.03E-06	0.000346

4220138	LOC651076		13.45122	XM	940198	PREDICTED:	1.31228	-5.303775	2.110665	-0.430651	2.433668	1.023206	1.608395	-3.0232	1.854533	-1.631846	1.153033	-5.296931	137.7792	0.000406	0.005952
6370379	C9orf97		38.24158	NM	13924	Homo sapiens	4.892356	7.001807	3.623127	4.803086	2.432349	1.115703	-1.350313	-4.841016	-2.011371	-0.643935	-1.489559	-3.29162	76.84852	2.31E-06	0.000133
4880368	TLE6		13.11798	NM	02476	Homo sapiens	1.995776	-1.460476	2.738189	1.31166	2.431326	0.143783	1.371992	-5.002035	1.218236	-6.144392	-1.126212	-5.451083	338.4315	0.000454	0.006434
1740603	NUP35		23.89433	NM	13828	Homo sapiens	1.81042	0.115637	2.487136	3.826664	2.430707	3.527471	1.373789	-3.715579	1.34262	-4.278757	-1.023215	-5.648331	1295.16	2.63E-05	0.000759
7150064	GTF2E1		22.77529	NM	00551	Homo sapiens	1.644995	-0.947372	2.202093	2.78939	2.426348	3.908447	1.338662	-3.914102	1.474988	-2.684089	1.101837	-5.351668	2420.674	3.35E-05	0.000903
4280246	CKAP5		21.08053	NM	00100	Homo sapiens	2.878992	2.627873	3.23881	3.457336	2.423136	0.711608	1.12498	-6.2474	-1.188126	-6.22111	-1.336619	-4.358837	165.6986	4.90E-05	0.001208
5080349	DIAPH3		16.28768	NM	03093	Homo sapiens	1.879876	-0.384811	2.181108	1.125545	2.422847	2.262404	1.16024	-5.935645	1.288833	-5.215014	1.110833	-5.398446	265.164	0.000169	0.003039
2230201	CHST8		11.8843	NM	02246	Homo sapiens	-1.18062	-6.332791	1.754977	-3.832028	2.419948	-0.96925	2.071961	-1.854233	2.857039	0.727752	1.378905	-4.564805	45.35993	0.0007	0.008966
290376	C18orf4		9.717088	NM	03216	Homo sapiens	-1.809647	-4.615118	1.222812	-6.798356	2.417869	-3.182767	2.212859	-3.209721	4.375489	0.897635	1.977301	-3.377477	134.3538	0.001634	0.017208
4220139	TMEM48		20.85722	NM	01808	Homo sapiens	1.511515	-2.665418	2.267839	2.449861	2.417403	3.143891	1.500375	-2.630797	1.599325	-1.936975	1.06595	-5.544921	691.9098	5.17E-05	0.001255
3180487	LRRC16		13.34703	NM	01764	Homo sapiens	1.371577	-5.092269	2.453483	0.52449	2.417203	0.325901	1.788804	-2.277887	1.762353	-2.651338	-1.015009	-5.661408	539.1488	0.00042	0.006109
6200594	SON		17.64925	NM	05818	Homo sapiens	2.790141	2.184403	2.829882	2.087726	2.416535	0.52568	1.014243	-6.510655	-1.154604	-6.401037	-1.171049	-5.255257	144.287	0.000116	0.002305
5130735	C9orf91		17.09859	NM	15304	Homo sapiens	1.261326	-5.355127	2.000314	0.084907	2.416349	2.226356	1.585882	-2.426489	1.915721	-0.222779	1.207985	-4.85145	217.6879	0.000134	0.002579
7610647		0	9.673957	BM467642		AGENCOURT	1.546302	-3.843462	1.314425	-5.785736	2.410135	0.478962	-1.176409	-6.034785	1.558644	-3.845876	1.833603	-1.672072	33.39592	0.001664	0.017441
3360088	KEL		15.76661	NM	00042	Homo sapiens	2.55625	1.284785	2.750454	1.72087	2.406908	0.388736	1.075972	-6.415662	-1.062047	-6.717473	-1.142733	-5.375373	654.4738	0.000197	0.003402
7510427	PIK3CD		11.38962	NM	00502	Homo sapiens	1.155257	-6.306889	1.76696	-2.768634	2.404328	0.435163	1.529496	-3.814135	2.081207	-0.761235	1.360714	-4.270828	283.4345	0.000841	0.01027
4810326	COMTD1		31.39349	NM	14458	Homo sapiens	-1.104306	-6.322624	1.788467	-0.013586	2.404212	3.803049	1.975014	1.689151	2.654985	5.043778	1.344287	-3.319802	785.4319	6.53E-06	0.000274
7040193	GCSH		12.4106	NM	00448	Homo sapiens	2.407061	-0.35146	2.91729	1.041556	2.403192	-0.719241	1.211972	-6.007122	-1.00161	-6.784995	-1.213923	-5.202065	108.1457	0.000579	0.007776
6200438	GTF2IP1		33.41622	NR	00220	Homo sapiens	2.875048	5.08507	3.07203	5.623738	2.402068	3.039921	1.068514	-6.371911	-1.196905	-5.796974	-1.27891	-4.135535	4819.417	4.72E-06	0.000217
4290609	C17orf80		32.5569	NM	01794	Homo sapiens	2.429838	3.9253	2.970441	5.885967	2.401599	3.592114	1.222485	-5.18917	-1.011758	-6.779977	-1.23686	-4.35865	300.7237	5.40E-06	0.000238
6900392	VPS13A		14.11042	NM	00101	Homo sapiens	1.881681	-2.895907	3.59831	2.273962	2.397967	-1.108372	1.912285	-2.611598	1.274375	-6.056276	-1.500567	-4.031847	89.28373	0.000327	0.005079
6130661		0	46.64731	AK027125		Homo sapiens	3.547093	6.054316	4.607274	8.027791	2.397722	2.197496	1.298887	-4.86117	-1.479359	-3.51896	-1.921521	-0.154749	95.69385	7.97E-07	6.53E-05
1260753	ZNRD1		9.598628	NM	17078	Homo sapiens	1.768829	-2.460396	1.885046	-2.108156	2.397433	0.347866	1.065703	-6.439725	1.355378	-5.256277	1.271816	-4.780245	533.2071	0.001718	0.017851
6020736	RAB28		27.12458	NM	00424	Homo sapiens	2.675918	4.23044	2.66657	4.050073	2.397182	2.852579	-1.003506	-6.514063	-1.116276	-6.408694	-1.112377	-5.350182	353.7296	1.39E-05	0.000469
3990577	PVRL1		12.58585	NM	20328	Homo sapiens	1.179125	-6.10538	1.634883	-3.127865	2.396242	1.183541	1.386521	-4.495355	2.03222	-0.337757	1.465697	-3.354336	254.5713	0.000545	0.007409
1340717	RAB9P1		9.620708	NR	00003	Homo sapiens	1.296405	-5.564998	1.712393	-3.213039	2.394462	0.242924	1.320878	-5.247591	1.847002	-2.145283	1.398314	-4.099	21.58856	0.001702	0.017734
510039	PAWR		36.77373	NM	00258	Homo sapiens	1.408494	-2.044359	2.204982	4.999601	2.393672	6.013045	1.565489	0.012477	1.699455	1.274655	1.085575	-5.320012	1488.07	2.85E-06	0.000152
7550377	C6orf85		13.28097	NM	02194	Homo sapiens	-1.154534	-6.380794	1.802449	-3.199271	2.392296	-0.51135	2.08099	-1.344698	2.761988	1.053428	1.327248	-4.670822	1275.422	0.000429	0.006205
6860541	LOC654121		32.08097	XM	94244	PREDICTED:	1.692562	1.273415	2.050484	4.020752	2.392109	6.021694	1.211467	-4.65882	1.413306	-2.046381	1.166607	-4.540347	2697.682	5.84E-06	0.000252
6590040	LOC650759		20.27649	XM	94479	PREDICTED:	1.53456	-2.781246	2.468546	2.866658	2.391733	2.47305	1.608634	-2.012839	1.558579	-2.651843	-1.032116	-5.638472	371.5745	5.93E-05	0.001389
7200187	FAM113B		26.45075	NM	13837	Homo sapiens	1.55008	-0.999014	2.024564	2.863886	2.391636	4.984732	1.306103	-3.776129	1.542911	-1.148645	1.181309	-4.578767	510.6377	1.58E-05	0.000519
7610594	RIPK5		10.44349	NM	01537	Homo sapiens	1.765203	-2.438674	2.091677	-0.94097	2.390378	0.380853	1.18495	-5.994728	1.354166	-5.244824	1.142804	-5.371155	182.7332	0.001213	0.013589
70025	HOMER2		11.92274	NM	00483	Homo sapiens	1.017588	-6.683616	1.49844	-4.543353	2.389967	0.370043	1.472542	-4.200039	2.34866	0.464468	1.59497	-2.918098	442.8238	0.00069	0.008891
990202	FLJ10560		10.8817	NM	01813	Homo sapiens	1.812667	-1.751918	1.840578	-1.901603	2.38863	0.91466	1.015398	-6.509486	1.317744	-5.333291	1.297761	-4.507355	314.0857	0.001021	0.011891
110255	SNRPC		23.2599	NM	00309	Homo sapiens	1.888067	0.426647	2.59585	3.982341	2.387614	3.01677	1.374872	-3.839256	1.264581	-5.170543	-1.087215	-5.460296	2746.405	3.01E-05	0.000834
1010639		37681	16.45297	XM	93800	PREDICTED:	1.231989	-5.604011	1.948436	-0.295184	2.387457	2.029956	1.581537	-2.501231	1.937889	-0.135694	1.225319	-4.746771	505.2668	0.000161	0.002937
1440767	MSH2		33.42175	NM	00025	Homo sapiens	2.091328	2.035386	3.246367	6.556153	2.387141	3.365558	1.552299	-1.921445	1.141448	-6.186716	-1.359939	-3.30739	2633.551	4.71E-06	0.000217
5090400	MAPK1		29.58665	NM	13895	Homo sapiens	1.728865	0.908315	2.242828	4.390302	2.384449	5.115985	1.297283	-3.818854	1.379199	-2.996084	1.063144	-5.498595	417.5486	8.88E-06	0.000342
150543	LIMA1		17.38647	NM	01635	Homo sapiens	1.315639	-4.8591	2.100594	0.783673	2.383844	2.194026	1.596634	-2.266437	1.811929	-0.828323	1.134843	-5.27491	948.6319	0.000124	0.002436
290270	HYLS1		17.17217	NM	14501	Homo sapiens	1.431818	-3.848811	2.209971	1.332235	2.383837	2.141049	1.543472	-2.737642	1.664902	-1.965299	1.078674	-5.523236	231.3068	0.000132	0.002539
2340156	XRN2		19.64576	NM	01225	Homo sapiens	2.184008	1.342571	2.577697	2.911988	2.383584	2.028294	1.18026	-5.810314	1.091381	-6.578839	-1.081438	-5.517071	1097.248	6.92E-05	0.001572
1030402	CIITA		8.708647	NM	00024	Homo sapiens	1.461929	-4.529595	1.710468	-3.283823	2.381272	0.100036	1.170007	-6.090509	1.628856	-3.530531	1.392176	-4.160742	18.29215	0.002537	0.024186
6400500	HDAC9		16.92236	NM	01470	Homo sapiens	3.336561	1.503685	4.007586	2.592695	2.378406	-1.54171	1.201113	-6.129171	-1.402856	-5.547063	-1.684988	-3.383351	68.18913	0.000141	0.002672
3290682	D15Wsu75e		25.91532	NM	01570	Homo sapiens	1.28783	-4.490872	2.117279	2.644218	2.377512	4.041491	1.644067	-0.57305	1.846138	0.967494	1.122909	-5.19142	1125.472	1.75E-05	0.000563
4070370	TLCD1		36.01438	NM	13846	Homo sapiens	1.007982	-6.686205	1.863421	1.776639	2.376317	5.033831	1.848666	1.952183	2.3575	5.067851	1.275244	-3.547771	1145.263	3.18E-06	0.000165
540193	MMP25		16.3826	NM	02271	Homo sapiens	1.704845	-2.024732	2.508544	2.101291	2.375372	1.476784	1.471421	-3.664614	1.393307	-4.538912	-1.056064	-5.600086	1459.539	0.000164	0.002976
2650139	ACSL3		16.38985	NM	00445	Homo sapiens	1.987507	-0.376904	2.59975	2.242684	2.374286	1.256126	1.308046	-5.032719	1.194605	-6.083966	-1.094961	-5.495359	773.3127	0.000164	0.002973
4050068	LOC391692		15.44635	XM	37303	PREDICTED:	1.384107	-4.789116	2.514351	1.408788	2.371946	0.76466	1.816587	-1.657986	1.713701	-2.525324	-1.060037	-5.601333	496.7514	0.000216	0.003669
5420113	SLC25A20		12.15139	NM	00038	Homo sapiens	1.486353	-4.440137	2.519116	0.536459	2.370098	-0.103979	1.69483	-2.99067	1.594572	-3.838297	-1.062874	-5.607837	27.85678	0.000635	0.008348
780669	NUFIP1		13.1768	NM	01234	Homo sapiens	1.674521	-2.908084	2.515209	1.094372	2.369739	0.447276	1.502047	-3.92347	1.415174	-4.773692	-1.061387	-5.602978	123.7417	0.000445	0.006346
4830072	STT3B		25.91106	NM	17886	Homo sapiens	2.141319	2.602399	2.48316	4.161087	2.369165	3.580827	1.15964	-5.73772	1.106405	-6.407165	-1.048116	-5.590409	2215.787	1.75E-05	0.000563
7560095	MRPL47		10.41295	NM	02040	Homo sapiens	1.732254	-3.218202	2.539076	0.124798	2.367998	-0.577237	1.465764	-4.604706	1.367004	-5.430864	-1.072246	-5.598195	1309.557	0.001228	0.013704
3400131	KCMF1		18.04965	XM	94213	PREDICTED:	2.008828	0.69835	2.286361	1.97606	2.367457	2.324621	1.138157	-6.041649	1.178527	-6.033004	1.035469	-5.632985	293.0403	0.000104	0.002127
5960689	LOC133993		12.18491	XM	92693	PREDICTED:	2.934426	0.848605	2.801486	0.198775	2.366814	-1.302459	-1.047454	-6.487074	-1.239821	-6.216402	-1.183653	-5.347989	2689.969	0.000628	0.008271
4230356	LOC133993		24.55041	XM	92693	PREDICTED:	5.937842	4.143724	5.296781	3.326545	2.365073	-2.354151	-1.121028	-6.391332	-2.510638	-1.585541	-2.239585	-1.967559	88.46263	2.30E-05	0.000686
3800463	VAV3		15.16447	NM	00611	Homo sapiens	-1.27351	-5.807573	1.697473	-3.638262	2.36438	-0.391286	2.161748	-0.783554	3.011062	2.041062	1.392883	-4.28224	135.2934	0.000235	0.00393
5420280	ECE1		10.40637	NM	00139	Homo sapiens	2.571157	-1.108669	3.329149	0.541627	2.362119	-2.154023	1.294806	-5.872682	-1.088496	-6.711908	-1.409391	-4.690385	42.37154	0.001231	0.013731
5050181	FLJ90086		9.606815	XM	37182	PREDICTED:	1.305305	-5.461006	1.683561	-3.267726	2.359724	0.292069	1.289784	-5.392636	1.807796	-2.229717	1.401627	-4.018139	399.9403	0.001712	0.017816
7400066	RABL2A		42.37666	NM	01341	Homo sapiens	3.060206	7.077118	2.720489	5.917677	2.358102	4.268417	-1.124873	-5.93798	-1.297741	-4.341599	-1.153678	-4.919004	303.4101	1.34E-06	9.15E-05
1570181	OGFOD1		10.72243	NM	00103	Homo sapiens	1.82078	-2.278127	2.303585	-0.168355	2.358063	0.010507	1.265164	-5.599632	1.295085	-5.68456	1.023649	-5.656255	27.53249	0.001086	0.012472
2100193	SLC25A12		23.54384	NM	00370	Homo sapiens	1.328155	-4.251599	2.209405	2.665539	2.354971	3.389596	1.663514	-0.792715	1.773114	-0.054276	1.065884	-5.5315	392.7069	2.84E-05	0.000801
10273	DSCR1L2		18.57407	NM	01344	Homo sapiens	1.681305	-1.408118	2.310579	2.327897	2.354952	2.499475	1.374277	-3.999306	1.400669	-3.989609	1.019204	-5.654994	251.8408	9.07E-05	0.001923
5290008	RPL9		11.93799	NM	00102	Homo sapiens	1.44597	-4.166741	1.941526	-1.033137	2.353872	1.065301	1.342715	-4.802303	1.627884	-2.871811	1.212382	-4.956533	2679.047	0.000686	0.008857
3830050	IREB2		34.49406	NM	00413	Homo sapiens	2.2801	4.159165	2.713565	6.009917	2.353369	4.360379	1.190108	-5.292604	1.032134	-6.74012	-1.153056	-4.908607	702.3487	3.99E-06	0.000193
6040630	CXXC6		10.67058	NM	03062	Homo sapiens	2.930424	-0.12055	3.162763	0.160392	2.35167	-2.192761	1.079285	-6.455781	-1.246103	-6.308931	-1.344901	-4.92131	1171.181	0.001109	0.012681
620408	ARFGEF2		18.86911	NM	00642	Homo sapiens	1.413179	-3.678602	2.134751	1.601475	2.351212	2.70252	1.510602	-2.607759	1.663775	-1.465991	1.101399	-5.401007	176.715	8.41E-05	0.001814
2190008	TNFRSF12A		13.10706	NM	01663	Homo sapiens	1.190447	-6.108195	2.088108	-0.73891	2.350376	0.454396	1.754053	-2.200478	1.974363	-1.108958	1.125601	-5.419678	2430.191	0.000455	0.006452
780289	LRRC45		9.478975	XM	93825	PREDICTED:	1.402162	-4.706833	1.546652	-4.028859	2.349711	0.539401	1.103048	-6.322042	1.675777	-2.864815	1.519224	-3.201725	171.3862	0.001808	0.018629
290131	FLJ20718		11.72302	NM	18292	Homo sapiens	1.932342	-0.737354	1.794849	-1.920592	2.349029	1.096362	-1.076604	-6.390292	1.215639	-5.95666	1.308761	-4.351898	546.0794	0.000742	0.009348
1030035	CDC42		20.85732	NM	04447	Homo sapiens	2.596876	3.11267	2.478836	2.432197	2.348462	1.798073	-1.047619	-6.456747	-1.105777	-6.517507	-1.055515	-5.595005	677.634	5.17E-05	0.001255
4120050	MKKS		16.74039	NM	17078	Homo sapiens	1.732071	-0.87336	2.027911	0.917894	2.348231	2.642466	1.170801	-5.767913	1.355736	-4.346917	1.157956	-5.081107	588.564	0.000149	0.002769
5810044	LOC653496		19.35644	XM	93023	PREDICTED:	1.165955	-5.916201	1.70872	-1.055059	2.347835	3.051862	1.465511	-2.82886	2.013658	1.402635	1.374031	-3.22885	411.2556	7.43E-05	0.001657
780224	USP54		30.65553	NM	15258	Homo sapiens	3.383791	5.198448	3.279041	4.831707	2.346255	1.503669	-1.031945	-6.489567	-1.442209	-4.06464	-1.397563	-3.612597	122.0126	7.39E-06	0.000298
3830463	MTRF1L		17.59744	NM	01904	Homo sapiens	2.753842	1.010899	3.661374	3.083372	2.34524	-0.764655	1.329552	-5.406414	-1.174226	-6.406969	-1.561194	-3.527637	78.81533	0.000117	0.002331
2600768	THY1		26.37473	NM	00628	Homo sapiens	-1.109512	-6.341049	1.801803	-0.511313	2.345194	2.783789	1.999123	1.213583	2.602022	4.109407	1.301582	-3.939943	4980.724	1.60E-05	0.000525
5360255	PPIAL4		16.44653	NM	17823	Homo sapiens	1.826732	-0.863363	2.373826	1.932497	2.34493	1.749561	1.299494	-4.916558	1.283675	-5.301659	-1.012323	-5.661437	3825.833	0.000161	0.002938
5870754	BOLA2		22.25622	NM	00103	Homo sapiens	1.495677	-1.942656	1.917019	1.612448	2.344282	4.222235	1.281706	-4.277585	1.567371	-1.254102	1.222879	-4.277197	1026.829	3.75E-05	0.000981

4920470	UBE2E3		33.19038	XM	94499	PREDICTED:	2.724632	5.30668	2.715257	5.182567	2.343626	3.50208	-1.003453	-6.513999	-1.162571	-5.968008	-1.158571	-4.97074	883.9011	4.89E-06	0.000223
6040600	ICA1		10.65321	NM	02230	Homo sapiens	1.137347	-6.389067	1.737873	-3.008703	2.342751	0.091102	1.528005	-3.866033	2.059838	-0.94249	1.348057	-4.366142	510.3649	0.001116	0.012742
6290528	CHID1		16.7993	NM	02394	Homo sapiens	1.348178	-4.384881	1.939263	0.22632	2.339053	2.46616	1.438433	-3.375378	1.734974	-1.021446	1.206155	-4.766235	204.2035	0.000146	0.002731
2490307	TPP2		27.70918	NM	00329	Homo sapiens	2.144927	2.396635	2.780386	5.121856	2.337552	3.177365	1.296261	-4.475463	1.089805	-6.522282	-1.189443	-4.773683	504.8466	1.24E-05	0.000436
7330541	ARPC4		21.09336	NM	00102	Homo sapiens	1.434148	-2.54288	1.670117	-0.348567	2.335329	4.330892	1.164536	-5.546399	1.628373	-0.524737	1.398303	-2.445987	4512.787	4.89E-05	0.001206
3420343	CCDC64		26.90008	NM	20731	Homo sapiens	1.347515	-3.769671	2.270832	3.613193	2.333949	3.906699	1.685199	-0.138672	1.732039	0.106857	1.027795	-5.636863	1133.663	1.45E-05	0.000485
830484	AIF1		18.18888	NM	00162	Homo sapiens	1.47354	-3.044275	2.098671	1.479706	2.331297	2.698787	1.424238	-3.375203	1.582107	-2.11108	1.110845	-5.347875	267.9911	0.0001	0.002068
3360307	LOC650982		22.32556	XM	94008	PREDICTED:	2.350876	2.78387	2.485964	3.20166	2.32966	2.429203	1.057463	-6.417998	-1.009107	-6.78245	-1.067093	-5.547985	2914.573	3.70E-05	0.000972
5910474	PPID		24.33014	NM	00503	Homo sapiens	1.946351	1.493623	2.401542	3.902882	2.328624	3.49904	1.233869	-5.008062	1.196405	-5.641411	-1.031314	-5.631984	2473.764	2.41E-05	0.000705
3400601	LOC653888		22.99284	XM	93625	PREDICTED:	1.365931	-3.304754	1.812073	1.006449	2.326443	4.381482	1.326622	-3.636408	1.703192	0.263218	1.283857	-3.583015	1049.409	3.19E-05	0.000871
630626	C21orf91		19.29591	NM	01744	Homo sapiens	1.482627	-3.116284	2.322127	2.416764	2.325979	2.389716	1.566225	-2.223907	1.568823	-2.415041	1.001659	-5.664958	148.9729	7.55E-05	0.001676
650767	PPP2R5A		29.82602	NM	00624	Homo sapiens	1.524688	-0.89886	2.138219	4.134004	2.324016	5.179175	1.402397	-2.26819	1.524256	-0.974272	1.086893	-5.33908	898.3326	8.52E-06	0.000331
2940221	CSNK2A1P		20.37744	NR	00220	Homo sapiens	1.477171	-2.274557	1.838146	0.819747	2.322362	3.901421	1.244369	-4.773875	1.572169	-1.350895	1.263427	-3.93126	466.1801	5.79E-05	0.001366
6620601	ARHGAP26		15.18642	NM	01507	Homo sapiens	-1.001373	-6.689328	2.139538	-0.732944	2.321619	0.053929	2.142476	-0.312671	2.324807	0.340059	1.085103	-5.553833	36.80921	0.000234	0.003906
4480364	SYPL1		28.68663	NM	18271	Homo sapiens	1.782784	0.843163	2.495294	5.031974	2.320155	4.135374	1.399662	-2.868373	1.301423	-4.272154	-1.075486	-5.458264	3289.647	1.04E-05	0.000381
7000465	TARS		35.18258	NM	15229	Homo sapiens	1.624396	0.419286	2.388407	5.833281	2.315008	5.41614	1.470336	-1.235262	1.42515	-2.011604	-1.031706	-5.6154	9859.022	3.60E-06	0.00018
4920671	ADAMTS1		11.96767	NM	00698	Homo sapiens	1.745561	-1.961273	2.080976	-0.231301	2.313912	0.887076	1.192153	-5.848326	1.325598	-5.183316	1.111936	-5.438938	936.8987	0.000679	0.008785
7040471	TAF4B		17.08292	XM	29080	PREDICTED:	1.336689	-4.718252	2.22209	1.324841	2.313683	1.734924	1.662384	-1.829304	1.730906	-1.512844	1.041219	-5.624908	827.1633	0.000135	0.002586
5960184	EDNRB		14.84567	NM	00399	Homo sapiens	1.720649	-1.622562	2.212125	1.131559	2.312938	1.585539	1.285634	-5.03347	1.344224	-4.795267	1.045573	-5.617717	2183.669	0.000259	0.004251
840121	C9orf126		13.83009	NM	17369	Homo sapiens	1.698527	-1.569368	1.923629	-0.273753	2.312855	1.885406	1.132528	-6.091212	1.361683	-4.527436	1.202339	-4.866097	112.944	0.000358	0.005436
3930575	PI4K2B		15.41446	NM	01832	Homo sapiens	1.855181	-0.607631	2.233485	1.329723	2.311275	1.668124	1.203918	-5.64684	1.245849	-5.587912	1.034829	-5.63651	892.9907	0.000218	0.0037
5690162	EEF1B2		18.62699	NM	02112	Homo sapiens	1.890741	0.606373	2.104851	1.7364	2.311214	2.830016	1.113242	-6.130394	1.222386	-5.520837	1.098041	-5.400444	2267.671	8.95E-05	0.001905
6580408	WDR50		25.06671	NM	01600	Homo sapiens	1.868895	1.241726	2.357186	4.033498	2.310055	3.75076	1.261273	-4.612017	1.236054	-5.143754	-1.020402	-5.649819	1524.378	2.07E-05	0.000635
1240019	SLC30A5		23.88509	NM	02290	Homo sapiens	2.287801	2.660683	2.622796	3.970439	2.309526	2.515391	1.146427	-5.938187	1.009496	-6.782116	-1.135643	-5.21381	818.682	2.64E-05	0.000759
6840129	LOC648210		20.33609	XM	93916	PREDICTED:	2.480004	2.599078	2.579652	2.800161	2.309045	1.560871	1.040181	-6.473443	-1.074039	-6.649998	-1.117194	-5.379659	27861.49	5.85E-05	0.001376
2070673	LYPLA1		11.07277	NM	00633	Homo sapiens	2.065708	-1.268422	2.525852	0.373653	2.308285	-0.540705	1.222753	-5.883504	1.11743	-6.583864	-1.094255	-5.546224	1059.524	0.000948	0.011239
7200168	SRI		14.49489	NM	00313	Homo sapiens	1.767439	-1.456135	2.265492	1.179781	2.307168	1.331986	1.281794	-5.126142	1.305374	-5.194166	1.018396	-5.657463	988.5595	0.000289	0.004639
2450717	CA11		14.51077	NM	00121	Homo sapiens	1.71864	-1.746367	2.237202	1.114273	2.306443	1.405156	1.301728	-4.946299	1.342017	-4.867579	1.03095	-5.643541	2149.469	0.000288	0.004625
1690176	TBPIP		10.47607	NM	01655	Homo sapiens	2.212736	-1.054734	2.60991	0.146959	2.306165	-1.036353	1.179495	-6.130387	1.042223	-6.75998	-1.13171	-5.468163	325.6158	0.001197	0.013464
4260278	ACOT9		22.72786	NM	00103	Homo sapiens	1.439572	-2.626084	2.022901	2.275595	2.304927	3.920306	1.40521	-2.876045	1.601119	-0.980847	1.139417	-5.043787	910.7442	3.38E-05	0.00091
3360603	KIAA0664		11.28887	NM	01522	Homo sapiens	1.068152	-6.601722	1.553119	-4.000074	2.30413	0.304948	1.454025	-4.191706	2.157119	-0.111012	1.48355	-3.431279	740.2299	0.000873	0.010564
4210181		0	14.557	BX956267		DKFZp781G0	1.162743	-6.09794	1.712245	-1.935392	2.300859	1.626558	1.472591	-3.403095	1.97882	0.124662	1.343768	-3.891063	181.7523	0.000284	0.004579
4890255	CLDN23		28.13688	NM	19428	Homo sapiens	1.469996	-2.256801	2.408583	4.533257	2.300635	3.944024	1.638496	-0.404623	1.565062	-1.31251	-1.046921	-5.583322	431.443	1.15E-05	0.000409
4890491	GCHFR		31.42891	NM	00525	Homo sapiens	1.223766	-4.75927	1.940969	2.894161	2.299605	5.181258	1.586062	-0.00759	1.879121	2.578237	1.184772	-4.40785	2302.024	6.49E-06	0.000273
1980367	LOC441511		20.26425	XM	49714	PREDICTED:	3.679856	1.753766	5.11359	3.700528	2.296023	-2.19488	1.389617	-5.452504	-1.602709	-4.751189	-2.227151	-1.702505	41.51067	5.95E-05	0.001391
3420747	CDC42EP2		13.2844	NM	00677	Homo sapiens	1.177659	-6.041965	1.699168	-2.264806	2.295357	1.286936	1.442835	-3.808074	1.949084	-0.329693	1.350871	-3.93791	221.2371	0.000429	0.006199
6900059	TUBB4Q		27.21936	NM	02004	Homo sapiens	1.457368	-2.196008	2.24159	3.98909	2.290066	4.218568	1.538109	-1.171749	1.571372	-1.009651	1.021626	-5.645941	6802.192	1.36E-05	0.000464
4180711	PNPLA5		19.9393	NM	13881	Homo sapiens	-1.161522	-6.170073	1.830105	-1.535554	2.287067	0.996992	2.125707	0.597726	2.656479	2.77947	1.249692	-4.691172	104.1602	6.44E-05	0.00148
620010	PCNXL2		21.57287	NM	02493	Homo sapiens	1.046109	-6.621392	1.858521	-0.030093	2.286907	2.55717	1.776603	-0.247713	2.186108	2.2434	1.230498	-4.512823	535.2545	4.38E-05	0.00111
6400113	LAMP2		16.78728	NM	00229	Homo sapiens	1.686856	-1.965463	2.515451	2.409662	2.28673	1.330151	1.491207	-3.366372	1.355616	-4.745902	-1.100021	-5.457731	519.7002	0.000147	0.002739
1770546	DNAJC7		26.33207	NM	00331	Homo sapiens	1.644727	0.463621	1.945535	2.872341	2.286195	5.046449	1.182892	-5.141797	1.390015	-2.602869	1.175099	-4.525363	5401.002	1.61E-05	0.000526
5050309		0	9.896516	BU536065		AGENCOURT	-1.273623	-6.041427	1.578021	-5.008257	2.285834	-1.955536	2.009805	-2.520401	2.911292	0.321792	1.448545	-4.403341	15809.66	0.001516	0.016279
4040215	LIAS		17.9684	NM	00685	Homo sapiens	1.537766	-2.330797	2.101766	1.659342	2.285769	2.637499	1.366766	-3.86411	1.486422	-2.903775	1.087547	-5.453519	1133.594	0.000106	0.002162
4490554	CSNK1D		15.10427	NM	13906	Homo sapiens	1.629031	-2.121548	2.149379	1.08011	2.285286	1.743367	1.319422	-4.656759	1.40285	-4.158648	1.063231	-5.570143	487.0766	0.00024	0.003985
6020564	STK32A		11.87362	NM	14500	Homo sapiens	1.368974	-5.456914	2.8681	0.714155	2.284846	-1.341904	2.095072	-1.631381	1.66902	-3.921486	-1.255271	-5.062558	45.83401	0.000703	0.008986
650685	LOC652324		10.65186	XM	94174	PREDICTED:	1.617339	-3.263638	2.171017	-0.370151	2.284347	0.109191	1.342339	-5.009657	1.412411	-4.77773	1.052201	-5.619284	774.8596	0.001117	0.012742
3400706	ZNF643		24.41455	NM	02307	Homo sapiens	2.092017	2.225048	2.486189	4.075151	2.28192	3.036396	1.188417	-5.500115	1.090775	-6.509139	-1.089517	-5.425429	135.6118	2.36E-05	0.000697
7400288		0	25.38861	CR593993		full-length cDN	1.607856	-0.66557	2.242648	3.860958	2.28072	4.032044	1.394806	-2.867625	1.418485	-2.811244	1.016976	-5.653514	222.8751	1.94E-05	0.000606
5340097		0	13.62343	AA227844		zr28h03.s1 St	-1.138717	-6.373123	1.024458	-7.06653	2.280028	-0.038276	1.166567	-6.077031	2.596306	1.503454	2.225595	-0.058125	17.09293	0.000383	0.005711
1500482	PANK1		21.00085	NM	13831	Homo sapiens	2.095113	2.144144	2.182568	2.430733	2.27932	2.915112	1.041742	-6.45416	1.087922	-6.530641	1.044329	-5.603942	702.2669	5.00E-05	0.001225
3120470	TAF9L		30.27632	NM	01597	Homo sapiens	2.031712	3.44678	2.237223	4.563282	2.277446	4.754901	1.101152	-6.049031	1.120949	-6.134929	1.017979	-5.650145	1075.793	7.88E-06	0.000312
160408	PDSS1		13.16414	NM	01431	Homo sapiens	1.707141	-2.148341	2.268361	0.830302	2.275838	0.814827	1.328749	-4.871658	1.333129	-5.091166	1.003296	-5.664807	983.8396	0.000447	0.006363
630437	ATP5S		20.03564	NM	01568	Homo sapiens	2.055554	1.079464	2.530671	3.219246	2.275243	1.986459	1.231138	-5.335761	1.106876	-6.479794	-1.112264	-5.367132	754.4052	6.29E-05	0.001453
3400048		0	21.25083	DA191955		DA191955 BR	-1.044225	-6.623146	1.406608	-3.876366	2.271942	2.750481	1.468815	-2.747261	2.372418	3.452849	1.615193	-1.251709	14.88454	4.71E-05	0.001172
1740133	RBBP5		19.84153	NM	00505	Homo sapiens	1.957444	1.371359	2.099578	2.041732	2.271792	2.978174	1.072612	-6.33534	1.160591	-6.007569	1.082023	-5.461895	878.2147	6.59E-05	0.001507
2120446	RANBP1		18.19544	NM	00288	Homo sapiens	1.958052	1.012295	2.092399	1.606526	2.270605	2.561978	1.068613	-6.366363	1.159625	-6.073776	1.085169	-5.46405	7497.685	0.0001	0.002068
2340026	C4orf13		15.13203	NM	00102	Homo sapiens	2.031062	0.284691	2.269803	1.286264	2.269193	1.233224	1.117545	-6.204226	1.117244	-6.47336	-1.000269	-5.665033	98.34274	0.000238	0.003963
3610564		0	8.712935	BX097226		BX097226 So	2.47873	-2.665717	4.064247	0.170202	2.267692	-3.652912	1.639649	-5.035318	-1.093063	-6.730581	-1.792239	-3.914684	67.1075	0.002532	0.024154
50278	DPY19L3		24.53397	NM	20732	Homo sapiens	2.408626	3.00762	2.75595	4.243387	2.265773	2.065897	1.1442	-5.978277	-1.063048	-6.669611	-1.21634	-4.697198	107.8807	2.31E-05	0.000687
2030019	SSR3		12.58098	NM	00710	Homo sapiens	1.94743	-1.089871	2.410305	0.881271	2.264614	0.183186	1.237685	-5.648599	1.162873	-6.331898	-1.064334	-5.593841	222.4765	0.000546	0.007416
5900689	HELLS		22.8717	NM	01806	Homo sapiens	1.798991	0.289787	2.420129	3.842457	2.264044	3.01237	1.345271	-3.874747	1.258508	-5.047664	-1.068941	-5.516708	1235.908	3.28E-05	0.000888
3190441	LOC642946		14.30639	XM	93187	PREDICTED:	1.463655	-3.679737	2.127135	0.678428	2.263397	1.34279	1.453303	-3.617126	1.546401	-3.06666	1.064059	-5.57357	450.1091	0.000307	0.004837
1940414	RSAFD1		16.6764	NM	01826	Homo sapiens	1.72869	-0.857865	2.115987	1.502016	2.261069	2.256308	1.224041	-5.319474	1.307967	-4.791954	1.068565	-5.538574	560.6952	0.000151	0.002803
840161	ASAH1		16.02727	NM	17792	Homo sapiens	1.929412	-0.398023	2.482726	2.166432	2.258644	1.090958	1.286779	-5.088428	1.170639	-6.182278	-1.099211	-5.465219	149.2798	0.000182	0.003215
7320450	ZNF81		16.64748	NM	00713	Homo sapiens	1.336651	-4.307715	1.849102	0.007956	2.258501	2.532899	1.383384	-3.679489	1.689671	-1.023316	1.221405	-4.560549	82.09065	0.000153	0.00282
840433	LOC649679		28.75795	XM	94504	PREDICTED:	1.42428	-2.34476	2.214525	4.235368	2.256692	4.439653	1.554838	-0.709562	1.584444	-0.571576	1.019041	-5.648978	6444.421	1.03E-05	0.000379
4890192	HIST1H2AC		17.34789	NM	00351	Homo sapiens	-1.216755	-5.9143	1.684468	-2.925687	2.255805	0.319369	2.049584	-0.269547	2.744761	2.528204	1.339179	-4.248831	134.8473	0.000126	0.002456
450615	C3orf58		20.46219	NM	17355	Homo sapiens	2.006767	0.142122	2.93235	3.922359	2.254899	1.159808	1.461231	-3.620948	1.123647	-6.44147	-1.300435	-4.276378	2170.535	5.67E-05	0.001344
5910088	WBSCR1		15.03143	NM	02217	Homo sapiens	2.0826	0.448607	2.306541	1.326827	2.253637	1.019391	1.107529	-6.259033	1.082126	-6.629781	-1.023475	-5.65291	5350.43	0.000245	0.00406
5670746	VHL		13.60813	NM	00055	Homo sapiens	2.771323	1.059605	2.853151	1.050457	2.253111	-1.147495	1.029527	-6.501803	-1.229998	-6.169675	-1.266316	-4.964627	103.0361	0.000385	0.005729
6130154	NP		12.76307	NM	00027	Homo sapiens	1.333693	-4.823428	1.870745	-0.949051	2.251887	1.20513	1.40268	-4.083041	1.688459	-1.996138	1.203738	-4.915089	3319.318	0.000512	0.007055
4900048	LMNB1		28.52678	NM	00557	Homo sapiens	2.148949	2.8361	2.692925	5.25766	2.251078	3.17042	1.253136	-4.782834	1.047525	-6.700072	-1.196282	-4.639339	6550.021	1.07E-05	0.000389
1940682	C18orf26		13.4313	NM	17362	Homo sapiens	1.240224	-5.684308	2.044084	-0.326647	2.249837	0.700066	1.648158	-2.424743	1.814057	-1.490173	1.100658	-5.474686	463.1413	0.000408	0.005977
2100187	PRKX		19.57021	NM	00504	Homo sapiens	1.611334	-1.120731	2.046469	2.030154	2.24855	3.190689	1.270047	-4.590005	1.395458	-3.437222	1.098746	-5.358026	1310.44	7.05E-05	0.001595
650451	RPE		27.22485	NM	00691	Homo sapiens	1.823378	1.698031	2.227177	4.271785	2.247524	4.350486	1.221457	-4.790575	1.232615	-4.903262	1.009136	-5.661324	1579.118	1.36E-05	0.000464
4120762	ZNF394		17.90069	NM	03216	Homo sapiens	1.675659	-0.446512	1.80274	0.354175	2.24715	3.289988	1.075839	-6.302062	1.341054	-3.998923	1.24652	-4.151149	256.1191	0.000108	0.002187
780408	REL		20.6533	NM	00290	Homo sapiens	2.140863	0.530111	3.11624	4.056015	2.246608	0.739978	1.4556	-3.852921	1.049394	-6.729725	-1.387087	-3.787012	112.4993	5.42E-05	0.001299

1820440	FAM111A		16.59967	NM	02207	Homo sapiens	1.107576	-6.415365	1.979557	-0.164266	2.244624	1.26752	1.787287	-1.010513	2.026609	0.339746	1.133902	-5.299026	375.9012	0.000155	0.002847
990280	FLJ37953		22.3945	NM	15238	Homo sapiens	2.503986	2.735875	2.839026	3.802265	2.243186	1.272495	1.133803	-6.106762	-1.116263	-6.466925	-1.265622	-4.480334	60.21514	3.64E-05	0.000961
5890692	ITGB5		15.5422	XM	94469	PREDICTED:	1.187044	-5.810209	1.666924	-1.720005	2.242393	2.10196	1.404265	-3.636586	1.889056	0.2161	1.345228	-3.623413	14806.72	0.00021	0.003589
5550315	LOC644762		21.02444	XM	93033	PREDICTED:	1.705532	0.180062	1.982175	2.125018	2.237894	3.69171	1.162203	-5.588527	1.312139	-4.140908	1.12901	-5.109567	1367.566	4.97E-05	0.00122
7650692	TNRC6A		17.98365	NM	01449	Homo sapiens	1.75361	-0.516722	2.211152	2.219413	2.237397	2.314973	1.260915	-4.934462	1.275881	-5.0433	1.011869	-5.660825	1715.209	0.000106	0.002155
6770176	C15orf23		13.78229	NM	03328	Homo sapiens	2.068679	0.126839	2.269981	0.880926	2.236255	0.66498	1.097309	-6.31463	1.081006	-6.64245	-1.015081	-5.660278	683.4938	0.000364	0.005501
5550711	LOC645460		10.95056	XM	92849	PREDICTED:	2.068843	-1.661794	2.800046	0.788465	2.235354	-1.307371	1.353435	-5.303138	1.080485	-6.69721	-1.252619	-5.037012	32.22272	0.000994	0.011639
1820288	TFB2M		20.4957	NM	02236	Homo sapiens	1.642805	-0.794039	2.136121	2.632103	2.234086	3.154476	1.300289	-4.242023	1.359922	-3.813873	1.045862	-5.593141	1778.549	5.63E-05	0.001336
6980039	BTBD7		16.93794	NM	01816	Homo sapiens	1.077922	-6.514707	1.674141	-1.734465	2.233936	1.957425	1.55312	-2.314094	2.072448	1.296091	1.334377	-3.746947	553.2508	0.000141	0.002663
2970220	CYP20A1		25.54248	NM	17753	Homo sapiens	1.754761	0.709128	2.307993	4.233126	2.233931	3.787152	1.315274	-3.812638	1.273068	-4.567836	-1.033153	-5.62179	853.9481	1.88E-05	0.000593
2030647	ARMCX6		17.7535	NM	01900	Homo sapiens	1.815268	0.230747	2.039329	1.526568	2.233698	2.626862	1.123431	-6.046329	1.230506	-5.393298	1.09531	-5.403552	730.6822	0.000113	0.002258
2600438	LOC644422		24.31848	XM	93025	PREDICTED:	1.892644	1.918912	2.133463	3.350485	2.233606	3.903924	1.12724	-5.883334	1.180151	-5.618173	1.046939	-5.577865	1364.994	2.41E-05	0.000706
6580646	LOC649260		19.42015	XM	93832	PREDICTED:	1.541754	-3.487847	3.05359	3.555038	2.231782	0.390123	1.980595	-0.479211	1.447561	-4.289064	-1.368229	-4.006137	38.37091	7.32E-05	0.001641
580367	IDI1		18.19249	NM	00450	Homo sapiens	1.78144	-0.545398	2.350457	2.633242	2.231727	1.983278	1.319414	-4.47559	1.252766	-5.349147	-1.053201	-5.589275	1657.27	0.0001	0.002068
7610528	C6orf192		19.33696	NM	05283	Homo sapiens	1.103528	-6.34947	1.73794	-0.691445	2.229757	2.585651	1.574895	-1.691117	2.020572	1.582409	1.282989	-3.983734	1360.374	7.47E-05	0.001664
1690608	ZWILCH		14.35707	NM	01797	Homo sapiens	1.9745	-0.960422	2.706126	1.995439	2.228868	-0.00976	1.370537	-4.769008	1.128826	-6.490726	-1.214126	-5.018293	789.1612	0.000302	0.00478
5890477	FANCL		14.96415	NM	01806	Homo sapiens	2.175086	0.739722	2.362698	1.376698	2.226996	0.680464	1.086255	-6.353113	1.023866	-6.77164	-1.060935	-5.590243	1485.863	0.00025	0.004128
4490324	QTRTD1		18.35988	NM	02463	Homo sapiens	1.505897	-3.058123	2.438068	2.689981	2.22507	1.606551	1.619014	-1.95407	1.477571	-3.395267	-1.095726	-5.448853	348.783	9.59E-05	0.002006
130021	PREI3		25.81809	NM	01538	Homo sapiens	1.93051	2.069504	2.309468	4.216489	2.22222	3.696077	1.1963	-5.215213	1.151105	-5.94384	-1.039262	-5.605129	535.1748	1.78E-05	0.00057
2650343	CCNJ		17.14526	NM	01908	Homo sapiens	2.191242	0.958298	2.593413	2.501882	2.222068	0.799273	1.183536	-5.851401	1.014068	-6.780177	-1.167117	-5.160179	832.222	0.000133	0.002553
6020471	IGSF1		35.74979	NM	00155	Homo sapiens	1.830812	1.894597	2.703063	6.708954	2.221133	4.354507	1.476429	-1.510952	1.213196	-5.101119	-1.216974	-4.141743	1020.611	3.31E-06	0.000169
2070703	ZNF92		8.968996	NM	00713	Homo sapiens	2.383938	-2.136472	3.414068	0.145835	2.220465	-3.063181	1.432112	-5.465089	-1.073621	-6.740001	-1.537546	-4.368881	174.5141	0.002257	0.022107
6590066	LOC642323		15.69121	XM	92586	PREDICTED:	1.766639	-1.685299	2.609075	2.371113	2.217274	0.598557	1.476858	-3.683636	1.255081	-5.658384	-1.176704	-5.128786	150.495	0.000201	0.003466
6860669	FLJ16542		16.01855	NM	00100	Homo sapiens	1.584898	-1.72243	1.898385	0.556213	2.215629	2.512728	1.197796	-5.446953	1.397963	-3.663151	1.167112	-4.947925	708.9017	0.000183	0.00322
6900500	C9orf41		10.2953	NM	15242	Homo sapiens	1.736158	-2.824727	2.394813	0.145548	2.215282	-0.684146	1.379375	-4.925413	1.275968	-5.813302	-1.081042	-5.569877	101.867	0.001288	0.014231
6290364	FAAH		13.62251	NM	00144	Homo sapiens	1.17643	-5.962969	1.632803	-2.337686	2.212169	1.488651	1.38793	-3.997362	1.880408	-0.234121	1.354829	-3.702643	640.5303	0.000383	0.005711
3990768	THRAP5		9.928374	NM	00548	Homo sapiens	1.557476	-3.264134	1.811337	-1.787456	2.211035	0.467571	1.162995	-6.007629	1.419627	-4.423955	1.220665	-4.896379	1094.27	0.001496	0.016101
2450484	AGPS		20.39536	NM	00365	Homo sapiens	1.774105	0.519183	2.070542	2.42796	2.205756	3.203641	1.167091	-5.5932	1.243307	-5.05579	1.065303	-5.516963	971.1505	5.77E-05	0.001363
870685	ZNF549		17.05103	NM	15326	Homo sapiens	1.724986	-0.619661	2.089784	1.6755	2.20561	2.29971	1.211478	-5.355807	1.278624	-4.965502	1.055425	-5.574896	212.2785	0.000136	0.0026
5570035	PPP1CA		12.64464	NM	00270	Homo sapiens	1.496192	-3.367241	1.981788	-0.108586	2.203972	1.103899	1.324554	-4.68907	1.473054	-3.649859	1.112113	-5.398038	2074.554	0.000534	0.007293
4120142	ZSCAN2		15.64518	NM	01789	Homo sapiens	-1.033711	-6.657771	1.325099	-5.115469	2.203652	1.3833	1.369769	-4.186875	2.277939	2.002021	1.663009	-1.502467	142.7617	0.000204	0.003506
1740615	ZBTB3		16.54516	NM	02478	Homo sapiens	1.069333	-6.557795	1.784685	-1.092302	2.202284	1.499731	1.668971	-1.543045	2.059494	0.950276	1.23399	-4.612439	1710.732	0.000157	0.002877
3140095	ANKRD13C		10.99099	NM	03081	Homo sapiens	2.478667	-0.057084	2.623674	0.161361	2.200145	-1.506528	1.058502	-6.467973	-1.126592	-6.581429	-1.1925	-5.276991	209.2761	0.000978	0.011509
3060670	FOXA3		9.35413	NM	00449	Homo sapiens	1.533347	-3.680812	1.899328	-1.575156	2.19958	0.000857	1.238681	-5.617818	1.434496	-4.494301	1.158084	-5.271463	3400.975	0.001907	0.01945
4810209	AURKAIP1		18.2246	NM	01790	Homo sapiens	1.280628	-4.688657	1.845135	0.459986	2.198374	2.75314	1.440805	-2.803862	1.716638	-0.362201	1.191444	-4.702852	855.1509	9.93E-05	0.002056
4810703	LOC645385		15.68805	XM	92842	PREDICTED:	2.453447	1.701298	2.433026	1.372385	2.198043	0.237446	-1.008393	-6.512893	-1.116196	-6.518705	-1.106906	-5.461641	27482.31	0.000201	0.003468
2120528	RNU15B		20.80265	NR	00002	Homo sapiens	-1.15585	-6.046232	1.413424	-4.042492	2.196781	1.882369	1.633706	-1.534567	2.539149	3.723857	1.554226	-1.948564	179.306	5.23E-05	0.001267
4760161	LOC90120		15.80981	XM	93123	PREDICTED:	1.844615	-0.205066	2.164998	1.527024	2.196129	1.649002	1.173686	-5.776686	1.190562	-5.910657	1.014379	-5.659419	470.1521	0.000194	0.003368
5130471	C22orf16		24.13929	NM	21372	Homo sapiens	1.337399	-3.679458	2.083723	2.954907	2.194658	3.595112	1.558041	-1.021081	1.640989	-0.367721	1.053239	-5.555629	4337.166	2.50E-05	0.000728
2030278	SLC29A1		11.06364	NM	00495	Homo sapiens	1.654563	-2.483253	2.017896	-0.431863	2.194273	0.470446	1.219595	-5.647231	1.326195	-5.125538	1.087406	-5.51753	2658.723	0.000951	0.011273
5670437	TSR1		15.8206	NM	01812	Homo sapiens	1.928327	0.28648	2.168886	1.463128	2.193856	1.550328	1.12475	-6.113109	1.137699	-6.300279	1.011513	-5.66149	1032.989	0.000194	0.003361
650040	PTPRZ1		28.17093	NM	00285	Homo sapiens	2.400305	2.774428	3.250874	5.598521	2.193007	1.484391	1.354359	-4.261595	-1.094527	-6.545816	-1.482382	-2.675122	4198.244	1.14E-05	0.000407
5550630		0	14.94303	BG674767		602620884F1	1.290312	-5.446396	2.502448	1.390943	2.192946	-0.037296	1.939413	-0.913783	1.699546	-2.600445	-1.141136	-5.344619	27.12914	0.000252	0.004152
110497	PIGO		11.79192	NM	03263	Homo sapiens	2.0449	-2.051915	3.1732	1.444419	2.188603	-1.816855	1.551763	-4.352813	1.070274	-6.7228	-1.449874	-4.224265	33.4705	0.000724	0.009188
4780129	TMEM69		15.85533	NM	01648	Homo sapiens	1.94893	-0.291211	2.512751	2.277168	2.188466	0.720303	1.289298	-5.072723	1.122906	-6.452543	-1.148179	-5.249153	757.5784	0.000192	0.00334
650504	CHPT1		20.98957	NM	02024	Homo sapiens	1.428684	-2.961745	2.147043	2.701292	2.188032	2.897239	1.502811	-2.035682	1.531501	-1.948897	1.019091	-5.652163	1027.538	5.01E-05	0.001228
1190048	LOC643997		11.45315	XM	29296	PREDICTED:	1.737548	-2.401468	2.36027	0.604886	2.187446	-0.243664	1.358391	-4.865953	1.258927	-5.789644	-1.079007	-5.56095	3933.861	0.000821	0.010104
4060056	KIAA0701		38.69158	NM	01505	Homo sapiens	1.957946	3.52145	2.593318	6.992286	2.187078	4.869267	1.324509	-3.060667	1.117027	-6.096623	-1.185745	-4.322779	395.5565	2.18E-06	0.000127
4890603		0	13.23197	AL049310		Homo sapiens	1.502597	-4.898574	3.47375	1.78966	2.186774	-2.095301	2.31183	-1.111606	1.455329	-5.228571	-1.588527	-3.708185	32.42668	0.000436	0.006254
6760048	MT2A		16.21411	NM	00595	Homo sapiens	1.263545	-4.916651	1.681065	-1.01853	2.184757	2.534478	1.330436	-4.049586	1.72907	-0.371024	1.299627	-3.772215	8617.247	0.000173	0.003088
3180347	GART		21.80759	NM	17508	Homo sapiens	1.919019	1.365295	2.287847	3.398294	2.184755	2.785188	1.192196	-5.408601	1.138475	-6.152476	-1.047187	-5.590404	1023.328	4.15E-05	0.001068
6220181	HMG1L1		14.46999	NM	00100	Homo sapiens	1.881094	-0.412888	2.216425	1.269295	2.183823	1.047452	1.178264	-5.822396	1.160932	-6.201344	-1.014929	-5.659647	7615.22	0.000292	0.004661
4220408	SKI		29.89866	NM	00303	Homo sapiens	2.071719	3.552248	2.343439	4.977471	2.182404	4.036809	1.131157	-5.792787	1.053427	-6.648668	-1.073788	-5.439679	246.226	8.41E-06	0.000327
60673	MLF2		11.70954	NM	00543	Homo sapiens	1.43192	-3.875296	1.810994	-1.160936	2.181541	1.07617	1.264732	-5.160645	1.523508	-3.164684	1.20461	-4.87272	4787.888	0.000746	0.009388
6370139	RBM16		24.79631	NM	01489	Homo sapiens	2.549993	3.689104	2.667404	3.995985	2.179673	1.686512	1.046044	-6.451127	-1.169897	-6.049562	-1.223763	-4.626156	1275.468	2.19E-05	0.00066
2750129	NFYA		21.58471	NM	02170	Homo sapiens	2.028435	1.586484	2.448631	3.621219	2.178506	2.196898	1.207153	-5.384438	1.073984	-6.607553	-1.123995	-5.251123	354.2526	4.37E-05	0.001108
5130070	TRIM61		15.46041	NM	00101	Homo sapiens	3.290296	2.183488	2.977848	1.144242	2.176659	-1.695204	-1.104924	-6.375734	-1.511627	-4.734768	-1.368082	-4.551151	52.0287	0.000215	0.003659
1260021	UBE2C		12.76515	NM	18179	Homo sapiens	1.454452	-3.326245	1.721576	-1.300111	2.174941	1.695701	1.18366	-5.674923	1.495368	-3.023118	1.263343	-4.308822	293.9034	0.000512	0.007055
1980647	HMGB3		22.78643	NM	00534	Homo sapiens	1.422379	-2.126085	1.794069	1.591094	2.174576	4.354472	1.261316	-4.130332	1.52883	-0.89705	1.212092	-4.125419	3602.463	3.34E-05	0.000902
290086	TBC1D8		17.42823	NM	00706	Homo sapiens	1.53865	-3.420728	2.811609	2.968669	2.174379	0.259816	1.827322	-1.259162	1.413173	-4.484653	-1.293063	-4.452278	138.8441	0.000123	0.002411
6480204	ESCO1		13.27566	NM	05291	Homo sapiens	1.741993	-1.618478	2.228804	1.018787	2.173563	0.682298	1.279456	-5.138425	1.247745	-5.640498	-1.025415	-5.650623	586.6376	0.00043	0.00621
2350707	MPP6		13.31648	NM	01644	Homo sapiens	1.690711	-1.849923	2.165032	0.877521	2.173511	0.871628	1.280545	-5.076556	1.28556	-5.291159	1.003916	-5.66467	2392.232	0.000424	0.006153
10609	EHD4		21.56389	NM	13926	Homo sapiens	1.466381	-1.876105	1.882536	1.914233	2.1732	3.886289	1.283798	-4.015211	1.482016	-1.767447	1.1544	-4.820355	2088.969	4.39E-05	0.001111
4810044	BSPRY		16.91863	NM	01768	Homo sapiens	1.107061	-6.336981	1.641983	-1.621503	2.172747	2.13755	1.483191	-2.64589	1.962626	1.087043	1.323246	-3.668173	308.9546	0.000141	0.002673
2490494	FLJ22313		35.1872	NM	02237	Homo sapiens	3.974364	6.646693	2.994997	4.232164	2.172328	0.832465	-1.327001	-4.704673	-1.829541	-0.956044	-1.378704	-3.67878	238.4715	3.60E-06	0.00018
7650202	LOC645330		10.79006	XM	93282	PREDICTED:	2.192928	-2.051888	3.377366	1.07283	2.171639	-2.524528	1.540117	-4.724617	-1.009803	-6.783962	-1.555215	-4.013457	57.76268	0.001058	0.012215
2750707	PSMD6		25.16954	NM	01481	Homo sapiens	1.818039	1.614374	2.173496	3.910007	2.17135	3.857219	1.195517	-5.114573	1.194336	-5.384726	-1.000988	-5.664991	3154.461	2.03E-05	0.000627
3840753	SLC7A3		16.33689	NM	03280	Homo sapiens	1.488592	-2.642475	1.969894	1.069975	2.170311	2.272123	1.323327	-4.197426	1.457962	-3.03348	1.10174	-5.369836	2831.315	0.000167	0.003006
110131	XPOT		23.37229	NM	00723	Homo sapiens	1.911217	1.674769	2.279289	3.766636	2.169462	3.103346	1.192585	-5.318514	1.135121	-6.130344	-1.050624	-5.57225	2854.374	2.94E-05	0.000819
2850343	GRINL1A		13.31494	NM	00101	Homo sapiens	1.908773	-0.369647	2.125736	0.62902	2.166112	0.796428	1.113666	-6.216967	1.134819	-6.3743	1.018994	-5.656646	30.70119	0.000424	0.006154
1110670	LOC647908		19.05424	XM	93841	PREDICTED:	2.03552	1.004056	2.490213	3.094686	2.164691	1.444633	1.223379	-5.390117	1.063458	-6.670098	-1.150378	-5.154533	1593.497	8.02E-05	0.001753
4220477	DMXL1		30.58824	NM	00550	Homo sapiens	1.751132	0.617769	2.704419	5.972661	2.162399	3.296994	1.544384	-1.237419	1.234858	-5.04696	-1.250657	-3.976069	543.556	7.48E-06	0.0003
7400475	DDX17		13.19886	NM	03088	Homo sapiens	2.168148	0.203292	2.405746	1.01773	2.159407	-0.179193	1.109585	-6.289654	-1.004048	-6.784684	-1.114077	-5.446994	183.0486	0.000441	0.006311
2320519	RIMS2		13.88274	NM	01467	Homo sapiens	1.093983	-6.495623	1.925339	-0.886965	2.158786	0.395248	1.759935	-1.529952	1.973327	-0.363287	1.12125	-5.386669	239.1962	0.000352	0.005371
5700136	SMCHD1		11.99765	XM	93889	PREDICTED:	2.082161	-0.481045	2.394264	0.681856	2.158549	-0.450023	1.149894	-6.138825	1.036687	-6.759071	-1.109201	-5.476277	177.0843	0.000672	0.008711
1340392		0	13.38309	BX089019		BX089019 So	1.345457	-4.973843	2.317699	0.861041	2.158196	0.034158	1.722612	-2.052248	1.604062	-3.10281	-1.073906	-5.563806	302.4002	0.000415	0.006054
5220079	USF2		18.69024	NM	00336	Homo sapiens	2.489486	0.309074	3.864812	3.711221	2.157896	-1.392278	1.552454	-4.057461	-1.153664	-6.470555	-1.791009	-2.341986	32.08103	8.80E-05	0.001881

1070279	GRB2		19.22249	NM	00208	Homo sapiens	1.712858	0.14351	1.967198	1.900699	2.156261	3.090614	1.148489	-5.738655	1.258867	-4.82181	1.096108	-5.347599	957.5489	7.69E-05	0.001696
3930465	ANKRD27		14.35984	NM	03213	Homo sapiens	1.700447	-2.149424	2.495146	1.908643	2.154694	0.271206	1.467347	-3.761698	1.267134	-5.57213	-1.158005	-5.225519	341.6103	0.000302	0.00478
2510754	ABHD5		16.57697	NM	01600	Homo sapiens	1.766668	-1.155674	2.484164	2.591222	2.152957	0.934594	1.40613	-3.941824	1.218654	-5.782734	-1.153838	-5.180447	809.8499	0.000156	0.002857
7400482	VRK2		15.05223	NM	00629	Homo sapiens	1.820152	-0.812619	2.329207	1.843574	2.151373	0.884197	1.279677	-5.045725	1.181974	-6.058915	-1.082661	-5.512102	646.4726	0.000244	0.004039
1340274	LCMT1		14.67307	NM	01630	Homo sapiens	1.655404	-1.548461	2.065842	1.081805	2.15049	1.527365	1.247939	-5.138943	1.299073	-4.92146	1.040975	-5.619519	819.543	0.000274	0.004435
840689	PPAP2A		17.90682	NM	17689	Homo sapiens	1.511015	-2.17146	2.02351	1.794965	2.149921	2.538686	1.339172	-3.866674	1.422832	-3.184013	1.062471	-5.537904	1034.551	0.000108	0.002185
5900730	PRKD3		18.08493	NM	00581	Homo sapiens	1.252408	-5.062393	1.949438	1.002065	2.148691	2.223842	1.556552	-1.79678	1.715648	-0.566089	1.102211	-5.362641	317.3684	0.000103	0.002113
1580047	LOC221710		19.52029	XM	92760	PREDICTED:	1.313718	-4.109178	1.889197	1.281016	2.148608	2.995409	1.438053	-2.527032	1.635517	-0.674141	1.137313	-5.064584	436.6707	7.14E-05	0.001609
7100059	RAD50		11.02691	NM	00573	Homo sapiens	1.903853	-1.980823	2.609804	0.815089	2.148152	-1.130993	1.370801	-5.025396	1.128318	-6.540558	-1.214907	-5.11687	522.6759	0.000965	0.011389
4560017	ZNF33A		15.0934	NM	00697	Homo sapiens	2.529386	1.04863	2.857414	1.920803	2.145672	-0.9089	1.129686	-6.252525	-1.178832	-6.311812	-1.331711	-4.484957	233.3863	0.000241	0.003995
6760372	KIAA1524		29.38456	NM	02089	Homo sapiens	1.673524	0.14518	2.543186	5.587525	2.144676	3.46478	1.519659	-1.310091	1.281533	-4.38534	-1.185813	-4.561763	1236.161	9.20E-06	0.000351
6330564	POLR2D		17.33212	NM	00480	Homo sapiens	1.485479	-2.185114	1.835672	0.798822	2.143463	2.893567	1.235744	-4.869778	1.442944	-2.733663	1.167672	-4.826716	2510.192	0.000126	0.002462
1690091	MRPL18		29.69915	NM	01416	Homo sapiens	1.614683	0.535912	2.172064	4.8971	2.142393	4.675386	1.345195	-2.724308	1.326819	-3.211696	-1.013849	-5.654834	2667.067	8.71E-06	0.000336
770725	VPS13A		10.01529	NM	01518	Homo sapiens	2.084282	-1.364761	2.525972	0.15222	2.142234	-1.471878	1.211915	-5.963454	1.027804	-6.773198	-1.17913	-5.296742	520.0915	0.001443	0.015616
3130240	KCNS3		15.87037	NM	00225	Homo sapiens	1.453421	-3.293089	2.113482	1.471153	2.141534	1.584525	1.454144	-3.140147	1.473444	-3.184558	1.013273	-5.659992	578.3048	0.000191	0.00333
6560274	VIL2		17.20173	NM	00337	Homo sapiens	1.07973	-6.478127	1.593467	-1.921155	2.140929	2.149809	1.475802	-2.6017	1.982837	1.402072	1.343566	-3.416921	8372.435	0.000131	0.002526
1980021	USP10		20.92791	NM	00515	Homo sapiens	1.766425	0.35218	2.232759	3.274393	2.139226	2.688519	1.263998	-4.614075	1.211048	-5.438521	-1.043723	-5.598403	1602.636	5.08E-05	0.001243
5270487	OGDH		14.4776	NM	00254	Homo sapiens	1.422618	-3.321825	1.787272	-0.285224	2.136616	2.062081	1.256327	-4.887059	1.501891	-2.598738	1.195462	-4.729207	683.4072	0.000291	0.004655
5670133	NRBF2		18.88179	NM	03075	Homo sapiens	2.018011	1.035355	2.437754	3.017836	2.134169	1.419484	1.207998	-5.486657	1.057561	-6.686742	-1.142249	-5.188443	1140.504	8.38E-05	0.00181
1400482	CHST7		14.97536	NM	01988	Homo sapiens	1.477845	-3.155979	2.09878	1.222345	2.134119	1.377259	1.420163	-3.548594	1.444075	-3.556919	1.016838	-5.657232	2583.348	0.000249	0.004117
5260754	ZFP64		17.53249	NM	19942	Homo sapiens	1.286044	-4.804859	2.037416	1.339849	2.133747	1.878508	1.58425	-1.717101	1.659156	-1.25189	1.047281	-5.598921	2348.936	0.000119	0.00236
5870243	ELL2		13.03529	NM	01208	Homo sapiens	1.816927	-1.360187	2.343836	1.24356	2.133565	0.154088	1.29	-5.146762	1.174271	-6.197899	-1.098554	-5.480148	256.3157	0.000467	0.006578
2470204	LOC649708		15.98814	XM	93877	PREDICTED:	1.489039	-2.428159	1.870189	0.668316	2.132528	2.387217	1.255971	-4.784363	1.432151	-3.110052	1.140274	-5.09982	534.8166	0.000184	0.003242
4830296	PRKACB		14.7327	NM	20757	Homo sapiens	1.919677	-0.320378	2.352595	1.755348	2.132142	0.590221	1.225516	-5.517911	1.110678	-6.500606	-1.103395	-5.441612	99.57922	0.000269	0.004368
1030014	LOC643940		23.98942	XM	92719	PREDICTED:	1.901738	1.897138	2.243391	3.898106	2.130845	3.192751	1.179653	-5.385169	1.120472	-6.21829	-1.052817	-5.557653	5888.578	2.58E-05	0.000747
5890687		0	13.12772	DA430048		DA430048 CC	-1.193271	-6.096977	1.515003	-4.317278	2.130455	-0.60613	1.807809	-1.893312	2.54221	1.459593	1.406238	-3.93426	102.6985	0.000452	0.006418
2640255	ZSCAN2		19.96107	NM	00100	Homo sapiens	-1.307895	-5.027249	1.485598	-3.871802	2.128206	0.58887	1.943006	-0.054958	2.78347	3.667836	1.432559	-3.287117	322.7963	6.40E-05	0.001473
2350040	FABP5		14.00029	NM	00144	Homo sapiens	1.669272	-1.169589	1.875928	0.152138	2.127516	1.742203	1.1238	-6.069744	1.274517	-5.04257	1.134114	-5.203331	5228.688	0.000339	0.005215
2030673		0	11.72682	XM	49847	PREDICTED:	-1.006757	-6.688282	1.443122	-4.464755	2.12729	0.134541	1.452873	-3.871466	2.141664	0.480377	1.474089	-3.192562	1101.002	0.000741	0.009338
7000608	RPS7		14.84625	NM	00101	Homo sapiens	1.517506	-2.053005	1.687294	-0.732796	2.127162	2.48088	1.111886	-6.085364	1.401749	-3.372224	1.260695	-4.048358	4163.79	0.000259	0.004251
7050468	ITPK1		13.32326	NM	01421	Homo sapiens	1.243874	-5.267647	1.604914	-2.108683	2.12659	1.663777	1.290255	-4.661322	1.709651	-0.961806	1.325049	-3.722896	1256.942	0.000423	0.006147
160184	SPSB2		19.54308	NM	03264	Homo sapiens	1.581405	-0.852581	1.935222	1.984076	2.123279	3.221116	1.223736	-4.875086	1.342654	-3.671724	1.097175	-5.319461	267.6906	7.10E-05	0.001603
2710398	ATF1		25.27761	NM	00517	Homo sapiens	1.816168	1.344983	2.324634	4.467108	2.122868	3.26783	1.279967	-4.184221	1.168872	-5.734174	-1.095044	-5.330166	861.785	1.98E-05	0.000617
6660709	ITGAM		10.36145	NM	00063	Homo sapiens	-1.438992	-5.367451	1.633348	-4.83051	2.122418	-2.746127	2.350374	-1.388698	3.054143	0.487438	1.299428	-5.024077	22.20604	0.001254	0.013948
610195	PLDN		19.96797	NM	01238	Homo sapiens	2.108104	2.360563	2.14111	2.337665	2.121958	2.17675	1.015656	-6.505444	1.006572	-6.783415	-1.009025	-5.662317	650.3478	6.39E-05	0.001472
1400753	DNAJB6		27.71649	NM	05824	Homo sapiens	1.217885	-4.650855	1.791518	2.188578	2.121105	4.69897	1.471007	-0.929331	1.74163	1.906427	1.183971	-4.287636	6642.262	1.24E-05	0.000435
1770433	MXRA7		31.50239	NM	00100	Homo sapiens	3.925046	5.777777	3.287345	4.268904	2.119262	-0.127949	-1.193987	-5.84457	-1.852082	-1.38088	-1.551174	-2.796404	168.5552	6.42E-06	0.000271
6480717	C10orf42		13.56611	NM	13835	Homo sapiens	1.220731	-5.485441	1.633576	-1.86873	2.119209	1.599179	1.338195	-4.198896	1.736016	-0.765772	1.297282	-3.966498	1073.405	0.00039	0.005787
6960093		0	11.03852	BM979066		UI-CF-DU1-ac	1.597262	-4.17536	2.936922	1.015886	2.118483	-1.95928	1.838722	-2.751099	1.326321	-5.745559	-1.386332	-4.468846	43.2166	0.000961	0.011349
4890286	TEAD4		18.28949	NM	20144	Homo sapiens	-1.097818	-6.398818	1.291389	-5.152302	2.118142	1.697144	1.417711	-3.360069	2.325335	3.033136	1.640204	-1.187786	3088.59	9.76E-05	0.002032
2710491	LOC644850		24.46656	XM	92987	PREDICTED:	1.766254	1.237412	2.164155	3.923641	2.118015	3.595762	1.22528	-4.73597	1.199157	-5.309426	-1.021784	-5.644133	2033.119	2.34E-05	0.000693
3440400	DCUN1D5		24.91452	NM	03229	Homo sapiens	1.568479	-0.205533	2.004616	3.489167	2.117695	4.222482	1.278064	-3.809937	1.350158	-3.037704	1.056409	-5.513746	6896.483	2.14E-05	0.00065
3290707	KLF10		12.06668	NM	00565	Homo sapiens	2.600073	1.035884	2.3516	-0.183736	2.117224	-1.283763	-1.105662	-6.346043	-1.228058	-6.102912	-1.1107	-5.499561	155.8558	0.000655	0.008544
3310520	THRAP3		15.83945	NM	00511	Homo sapiens	1.779339	0.417467	1.704035	-0.511026	2.113276	2.482722	-1.044192	-6.439692	1.187675	-5.686765	1.24016	-4.217958	471.9467	0.000192	0.003349
50040	SNAI3		21.88563	NM	17831	Homo sapiens	-1.173347	-5.805167	1.282492	-5.101481	2.112134	2.065159	1.504807	-2.258903	2.478266	4.156622	1.646899	-0.900829	348.4427	4.08E-05	0.001052
7560093	GSR		17.2913	NM	00063	Homo sapiens	1.300961	-4.506343	1.936535	1.043035	2.11194	2.144168	1.488541	-2.371099	1.623369	-1.2705	1.090577	-5.416567	795.9286	0.000128	0.002483
2370494	GABPB2		27.06946	NM	18142	Homo sapiens	1.598862	0.479789	2.011489	3.967501	2.111877	4.623782	1.258075	-3.913123	1.320862	-3.222436	1.049907	-5.535056	991.2993	1.40E-05	0.000473
110025	LOC388654		13.0409	XM	37127	PREDICTED:	1.893681	-0.398523	2.115066	0.646364	2.111692	0.575543	1.116907	-6.195764	1.115126	-6.472336	-1.001598	-5.664973	41105.37	0.000466	0.00657
3840538	ACADM		31.17466	NM	00001	Homo sapiens	2.405072	4.694723	2.548408	5.239151	2.11068	2.900123	1.059598	-6.368954	-1.139477	-6.071497	-1.207387	-4.425945	2610.9	6.77E-06	0.00028
2350754	UNG		11.69727	NM	08091	Homo sapiens	1.950484	-3.373729	3.967299	1.607002	2.109665	-3.171487	2.034007	-2.907492	1.081611	-6.724638	-1.880535	-3.020227	23.6564	0.00075	0.009413
830356	ADH5		31.74996	NM	00067	Homo sapiens	2.735071	4.885113	2.934548	5.481654	2.109195	1.75495	1.072933	-6.344659	-1.296737	-4.778549	-1.391312	-3.076579	467.2947	6.16E-06	0.000263
2320528	CXorf33		18.10033	NM	19845	Homo sapiens	1.027782	-6.663348	1.71783	-1.001885	2.107635	1.711824	1.671396	-0.966598	2.050664	1.606395	1.226917	-4.509349	288.3847	0.000103	0.002108
5090376	GAD1		11.36153	NM	01344	Homo sapiens	1.600074	-2.434079	1.910672	-0.465197	2.106828	0.680321	1.194115	-5.703006	1.316707	-4.970727	1.102664	-5.433022	1507.446	0.00085	0.010343
5810438	GPIAP1		21.77889	NM	20336	Homo sapiens	2.116751	2.470277	2.308879	3.327936	2.105593	2.138748	1.090766	-6.235349	-1.005299	-6.783966	-1.096546	-5.383288	1259.865	4.18E-05	0.001072
4880491	MLLT7		17.86413	NM	00593	Homo sapiens	1.386193	-2.92246	1.653073	-0.235637	2.103994	3.337487	1.192527	-5.173264	1.517822	-1.436188	1.272777	-3.619256	1236.279	0.000109	0.002204
2690239		0	12.91237	AI557257		PT2.1 15 E0	-1.072824	-6.566008	1.341068	-5.198397	2.103391	0.240848	1.43873	-3.867687	2.256569	1.294092	1.568445	-2.448726	26.99807	0.000487	0.006801
4830594	FRMD5		14.62847	NM	03289	Homo sapiens	-1.081979	-6.528279	1.546329	-3.377746	2.102556	0.417527	1.673095	-1.900983	2.274921	1.569313	1.359708	-3.829843	96.18411	0.000278	0.004494
1770347	DC2		18.45674	NM	02122	Homo sapiens	2.287126	2.417934	2.258519	2.052966	2.101413	1.139188	-1.012666	-6.509585	-1.088375	-6.566469	-1.074762	-5.522993	8085.235	9.35E-05	0.001969
6060152	LOC650406		20.82711	XM	93949	PREDICTED:	1.472963	-3.59448	2.877479	3.909975	2.100946	0.491187	1.953531	0.05699	1.42634	-4.080979	-1.369611	-3.727667	42.61718	5.20E-05	0.001261
10025	CD55		14.73952	NM	00057	Homo sapiens	1.433751	-3.690941	2.160952	1.346288	2.099948	0.950536	1.507202	-2.882059	1.464652	-3.492632	-1.029051	-5.643238	809.2291	0.000268	0.004361
2230754	CCDC5		12.60525	NM	13844	Homo sapiens	2.169552	0.42363	2.231688	0.453508	2.099297	-0.27538	1.02864	-6.496698	-1.033466	-6.760695	-1.063064	-5.590387	1641.408	0.000541	0.007374
2260201	LOC150084		23.41107	XM	93279	PREDICTED:	-1.000853	-6.689332	1.68829	-0.29314	2.098207	2.821943	1.689729	0.108711	2.099996	3.038129	1.242801	-4.077272	428.8509	2.92E-05	0.000816
5090091	DHX29		23.11849	NM	01903	Homo sapiens	1.573923	0.041609	1.829743	2.332255	2.096848	4.324719	1.162536	-5.299772	1.332242	-3.161686	1.145979	-4.7603	1280.835	3.11E-05	0.000853
5870136	FAM82B		20.84213	NM	01603	Homo sapiens	1.75125	0.487973	2.161268	3.177269	2.096813	2.736875	1.234129	-4.848873	1.197324	-5.508882	-1.03074	-5.629396	683.4112	5.19E-05	0.001259
6420762	MARS2		14.11442	NM	13839	Homo sapiens	1.334588	-4.652109	2.076227	0.745755	2.094307	0.799531	1.555707	-2.53716	1.569254	-2.640805	1.008708	-5.663084	1011.047	0.000327	0.005075
5560075	HSPC152		17.89768	NM	01640	Homo sapiens	1.779172	0.104524	2.152362	2.395898	2.092635	1.989988	1.209755	-5.292904	1.176185	-5.865308	-1.028541	-5.638475	15429.5	0.000108	0.002188
6960537	ZNF452		17.37414	NM	05292	Homo sapiens	2.822641	1.615464	3.308842	2.724322	2.091812	-1.529773	1.17225	-6.114261	-1.349376	-5.454155	-1.581807	-3.261846	67.39923	0.000125	0.002443
7550672	KIAA0241		8.853252	NM	01506	Homo sapiens	1.530572	-3.241864	1.582735	-3.169156	2.091806	0.22313	1.03408	-6.486077	1.366682	-4.687614	1.32164	-4.147391	305.5178	0.002377	0.022991
1990468	TRAPPC6B		14.44831	NM	17745	Homo sapiens	1.566435	-1.761582	1.827521	0.198958	2.09109	1.977195	1.166675	-5.687352	1.334936	-4.243971	1.144222	-5.089437	723.5593	0.000294	0.004682
6100438	UFM1		18.00162	NM	01661	Homo sapiens	1.838132	0.876578	2.008135	1.847547	2.090466	2.335784	1.092487	-6.207831	1.137277	-6.147672	1.040998	-5.606872	1334.659	0.000105	0.002148
5340703	ENSA		13.9605	NM	20716	Homo sapiens	-1.027192	-6.670758	1.666168	-2.466534	2.089744	0.279054	1.711474	-1.669397	2.146568	0.853828	1.254222	-4.597823	111.22	0.000343	0.005255
4290709	NHP2L1		9.964117	NM	00500	Homo sapiens	1.497033	-3.575739	1.887787	-1.048997	2.088727	0.100256	1.261019	-5.311823	1.395245	-4.496241	1.106442	-5.442082	1851.02	0.001474	0.015893
460239	PLEKHJ1		11.41334	NM	01804	Homo sapiens	1.106507	-6.420593	1.572732	-3.101978	2.087728	0.420829	1.421348	-3.881366	1.886773	-0.527685	1.327453	-4.03733	327.5614	0.000833	0.010209
2370615	TADA2L		12.08787	NM	13343	Homo sapiens	1.600057	-2.054339	1.83594	-0.492756	2.08734	1.110016	1.147422	-5.952905	1.304541	-4.887655	1.136932	-5.2232	158.9595	0.00065	0.00849
4760017	ARL6IP2		32.87971	NM	02237	Homo sapiens	2.209629	4.169353	2.604451	5.987596	2.085962	3.218924	1.178682	-5.330037	-1.059285	-6.625222	-1.248561	-3.881419	682.5048	5.13E-06	0.00023
4390397	CRLF3		12.57737	NM	01598	Homo sapiens	1.835624	-0.70561	2.06622	0.457056	2.085939	0.5187	1.125623	-6.143524	1.136365	-6.350205	1.009543	-5.662814	221.336	0.000546	0.00742
4120445	CDKAL1		18.93019	NM	01777	Homo sapiens	1.963601	2.109085	1.849217	1.01931	2.085809	2.65835	-1.061855	-6.360806	1.062237	-6.62812	1.127942	-5.130025	179.1445	8.28E-05	0.001796

2260243	MRPL39	27.31098	NM	01744	Homo sapiens	1.904838	2.260947	2.373871	4.980166	2.085264	3.276558	1.246233	-4.510337	1.09472	-6.392951	-1.138403	-4.973252	4395.642	1.34E-05	0.000459
5490068	IQWD1	12.34945	NM	01844	Homo sapiens	3.178106	0.056534	3.86073	1.121232	2.081923	-3.462207	1.21479	-6.177512	-1.526524	-5.319569	-1.854406	-3.23658	59.38409	0.000592	0.007896
4150358	NANS	20.52924	NM	01894	Homo sapiens	1.139475	-5.919614	1.661804	-0.201727	2.081501	3.128148	1.458395	-1.955905	1.82672	1.490737	1.252555	-3.85708	1772.296	5.58E-05	0.001328
2810504	SOCS5	12.77325	NM	01401	Homo sapiens	1.941772	-1.016969	2.511918	1.428659	2.081052	-0.609731	1.293622	-5.257845	1.071728	-6.683926	-1.207043	-5.034361	380.895	0.00051	0.007042
7380575	SAMD1	11.43723	NM	13835	Homo sapiens	2.013701	-0.473855	2.208238	0.248912	2.080529	-0.46188	1.096606	-6.331467	1.033186	-6.761584	-1.061383	-5.595621	662.3869	0.000826	0.010145
4180673	LOC645070	10.41562	XM	93495	PREDICTED:	-1.10669	-6.505939	1.584492	-4.061178	2.079722	-1.232916	1.753541	-2.507332	2.301607	0.083916	1.312548	-4.58944	34.72264	0.001227	0.013694
1940612	RCC1	12.86108	NM	00126	Homo sapiens	1.597936	-2.237171	2.034163	0.610421	2.077747	0.820316	1.272995	-4.998194	1.300269	-5.008901	1.021426	-5.653078	3317.682	0.000495	0.006895
2760463	LOC389293	13.43707	XM	93168	PREDICTED:	1.350508	-4.420643	1.97304	0.317699	2.077631	0.912864	1.460961	-3.242493	1.538406	-2.769598	1.05301	-5.593	1154.152	0.000408	0.00597
7200358	USP46	16.84237	NM	02283	Homo sapiens	1.726319	0.001781	1.941561	1.419789	2.076535	2.283494	1.124682	-5.979583	1.202868	-5.527075	1.069519	-5.502783	326.5976	0.000144	0.00271
3400301	FBXO3	11.86749	NM	03340	Homo sapiens	1.909352	-0.829646	2.218934	0.583734	2.076505	-0.215209	1.16214	-6.009659	1.087544	-6.621558	-1.068591	-5.573389	421.036	0.000704	0.009001
510451	SLC19A1	9.833435	NM	00305	Homo sapiens	1.438375	-3.931089	1.774871	-1.604589	2.075691	0.268953	1.233942	-5.456403	1.44308	-3.977354	1.169489	-5.118171	1003.291	0.001556	0.016584
6480328	TARSL1	21.66241	NM	02515	Homo sapiens	1.662136	-0.13274	2.184131	3.531742	2.072805	2.798079	1.31405	-3.830958	1.247073	-4.88328	-1.053708	-5.55469	409.93	4.29E-05	0.001094
3130521	IHPK2	14.18159	NM	00100	Homo sapiens	1.847044	-1.03547	2.462347	1.933167	2.069661	-0.043377	1.333128	-4.765474	1.120526	-6.47284	-1.189734	-5.039103	613.9427	0.00032	0.004996
20154	38961	10.42428	NM	14579	Homo sapiens	1.713712	-2.334965	2.188667	0.120267	2.069321	-0.552825	1.27715	-5.337905	1.207508	-6.051996	-1.057674	-5.603945	146.4972	0.001222	0.013658
1990079	LOC200810	14.49361	NM	00101	Homo sapiens	1.108048	-6.372665	1.783362	-0.938821	2.063833	0.905909	1.609462	-1.888426	1.862584	-0.131612	1.157271	-5.107238	158.1091	0.00029	0.004639
2650280	PPHLN1	10.44684	NM	20143	Homo sapiens	2.823235	0.250079	2.735758	-0.290265	2.062157	-2.757258	-1.031975	-6.502667	-1.369069	-5.699434	-1.326649	-4.873258	32.2088	0.001211	0.013581
5220075	MGC5297	20.63342	NM	02409	Homo sapiens	1.769027	0.872768	2.081731	2.956668	2.062128	2.784309	1.176766	-5.409187	1.165685	-5.785112	-1.009506	-5.661345	541.5559	5.45E-05	0.001304
3170482	HSPA4	20.83882	NM	19843	Homo sapiens	1.502776	-1.29098	1.920605	2.429453	2.061976	3.405581	1.278038	-3.996491	1.372112	-2.990234	1.073608	-5.434736	986.791	5.19E-05	0.001259
4120100	AK3L1	20.20453	NM	00100	Homo sapiens	1.190909	-5.424178	1.820579	1.04003	2.060543	2.769397	1.528731	-1.321902	1.730228	0.473166	1.131806	-5.070239	7887.341	6.04E-05	0.001407
7560037	BCAN	15.37205	NM	19842	Homo sapiens	-1.452646	-4.439495	1.27431	-6.018808	2.059768	-1.058662	1.851121	-1.711886	2.992114	2.844225	1.61638	-2.738494	66.73248	0.000221	0.003736
1010504	LOC646463	13.97788	XM	92938	PREDICTED:	1.542656	-2.773351	2.194309	1.430446	2.059109	0.615821	1.422423	-3.691682	1.334782	-4.730551	-1.06566	-5.560647	379.4876	0.000341	0.005237
5270025	STUB1	13.48324	NM	00586	Homo sapiens	1.522281	-2.096434	1.696297	-0.765327	2.058897	1.918623	1.114313	-6.080252	1.352508	-3.986557	1.21376	-4.510229	2044.666	0.000401	0.005908
1580278	RNF25	16.17083	NM	02245	Homo sapiens	1.043092	-6.623785	1.533488	-2.455504	2.0561	1.698465	1.470136	-2.61783	1.971158	1.388256	1.3408	-3.416502	222.6516	0.000175	0.003118
3830348	GTF2I	12.94823	XM	93950	PREDICTED:	1.987618	-0.495708	2.409895	1.321862	2.055598	-0.466888	1.212454	-5.730037	1.034202	-6.759348	-1.172357	-5.175516	299.5746	0.000481	0.006738
2340592	EMG1	18.99445	NM	00633	Homo sapiens	1.057003	-6.555907	1.557575	-1.621584	2.054642	2.503022	1.473577	-2.072129	1.943837	1.969517	1.319129	-3.315481	3591.469	8.14E-05	0.001775
6900220	KIAA1274	11.84599	NM	01443	Homo sapiens	1.476231	-3.312691	1.924979	-0.100029	2.05303	0.657253	1.303982	-4.72914	1.390724	-4.182473	1.066521	-5.556132	1228.731	0.00071	0.009062
1400142	PKP4	23.4117	NM	00362	Homo sapiens	2.130122	3.67646	1.961818	2.371697	2.051709	2.956464	-1.08579	-6.197526	-1.038218	-6.717243	1.045821	-5.578874	206.1084	2.92E-05	0.000816
4070554	UBQLN1	13.53466	NM	05306	Homo sapiens	1.774969	-0.856891	2.123497	1.114106	2.050415	0.635405	1.196357	-5.63585	1.155184	-6.196353	-1.035642	-5.632629	320.069	0.000395	0.005833
5720719	SOS1	8.769729	NM	00563	Homo sapiens	1.86961	-3.346653	2.992227	0.190532	2.049989	-2.982573	1.600455	-4.426615	1.09648	-6.689405	-1.45963	-4.391449	40.59914	0.002468	0.023655
3440170	ATP5I	20.14095	NM	00710	Homo sapiens	1.204874	-5.019659	1.562701	-0.692396	2.04789	3.594902	1.296983	-3.619526	1.699672	0.972166	1.310481	-2.963334	2324.149	6.13E-05	0.001422
2940066	DCUN1D1	17.28825	NM	02064	Homo sapiens	2.019303	1.768974	2.002157	1.415574	2.047352	1.661093	-1.008563	-6.511766	1.013891	-6.777942	1.022573	-5.648463	888.243	0.000128	0.002484
3170468	ZNF589	10.88525	NM	01608	Homo sapiens	1.194798	-5.827383	1.590726	-2.691562	2.046444	0.552548	1.331377	-4.507623	1.712795	-1.44599	1.286484	-4.233334	2711.418	0.001019	0.011881
510725	ZNF259	17.34417	NM	00390	Homo sapiens	1.600213	-0.991591	1.980578	1.8924	2.04642	2.291923	1.237696	-4.844708	1.278842	-4.62803	1.033244	-5.624477	1798.363	0.000126	0.002456
1500296	KIAA1957	11.24144	XM	06516	PREDICTED:	1.837477	-0.484017	1.768818	-1.284697	2.045183	0.518934	-1.038816	-6.472777	1.113039	-6.459899	1.156243	-5.144921	125.1572	0.000889	0.010716
4540358	LOC643430	20.6618	XM	92811	PREDICTED:	2.002896	2.089219	2.190673	3.032536	2.042654	2.076818	1.093753	-6.19688	1.01985	-6.769228	-1.072464	-5.489022	8385.712	5.41E-05	0.001297
5080326	PCNXL2	10.62564	NM	02493	Homo sapiens	-1.22206	-5.999456	1.308873	-5.874639	2.041794	-1.419437	1.599521	-3.453818	2.495195	0.872359	1.559963	-3.195886	158.1174	0.001128	0.012866
5050142	RNF126	12.75993	NM	19446	Homo sapiens	1.374085	-4.004839	1.832821	-0.263186	2.041235	1.109776	1.333849	-4.248282	1.485523	-2.992437	1.113712	-5.330485	584.5039	0.000513	0.007061
270348	CXorf38	16.80098	NM	14497	Homo sapiens	1.408891	-2.965052	1.863152	1.1336	2.040821	2.369297	1.322425	-3.827798	1.44853	-2.592353	1.095359	-5.351568	410.5949	0.000146	0.00273
1170220	ANKRD5	15.88815	NM	02209	Homo sapiens	1.211529	-5.254412	1.580682	-1.383942	2.039297	2.396748	1.3047	-4.019961	1.683243	-0.148195	1.290138	-3.611317	699.9935	0.00019	0.00332
6550242	ZFAND3	11.73191	NM	02194	Homo sapiens	1.938757	-0.147817	1.967734	-0.258723	2.039225	0.120516	1.014946	-6.508617	1.051821	-6.717074	1.036332	-5.634664	439.8497	0.00074	0.009323
3840367	PTPN12	27.11379	NM	00283	Homo sapiens	2.329365	3.732733	2.597913	4.808772	2.038892	1.843351	1.115288	-6.068586	-1.142466	-6.125688	-1.274179	-3.945811	4072.802	1.39E-05	0.00047
6660184	MGC27348	9.796551	XM	17115	PREDICTED:	1.95199	-1.899088	2.535649	0.321917	2.036266	-1.860699	1.299007	-5.501192	1.043175	-6.756066	-1.245244	-5.011091	98.69436	0.00158	0.016768
3170075	CDC73	13.25332	NM	02452	Homo sapiens	1.558824	-3.161944	2.404297	1.58355	2.036151	-0.321083	1.542379	-3.143837	1.30621	-5.26015	-1.180805	-5.100491	56.65755	0.000433	0.006226
3360161	LOC653874	12.24063	XM	93621	PREDICTED:	1.270937	-5.692105	2.47249	0.73619	2.035092	-1.316272	1.945407	-1.300111	1.601253	-3.586619	-1.214928	-5.063463	262.7476	0.000615	0.00814
2810255	HIP1	19.38667	NM	00533	Homo sapiens	2.20751	2.136889	2.418142	2.961457	2.034809	0.856853	1.095416	-6.257813	-1.084873	-6.577194	-1.188388	-4.881957	708.6368	7.38E-05	0.001649
3780139	PRPF38B	17.52795	NM	01806	Homo sapiens	1.535237	-1.516975	1.949658	1.852342	2.034637	2.398049	1.269939	-4.415668	1.325292	-4.001235	1.043587	-5.593548	681.8973	0.00012	0.002361
7510332	RET	12.32887	NM	02097	Homo sapiens	2.286322	-1.756007	3.752109	1.772448	2.030263	-3.134635	1.641111	-4.280472	-1.126121	-6.630425	-1.84809	-2.892703	36.61533	0.000596	0.007943
1400228	BCCIP	15.04737	NM	07846	Homo sapiens	1.432314	-2.909166	1.839027	0.612957	2.02814	1.929314	1.283955	-4.413294	1.415988	-3.171559	1.102833	-5.329592	5626.523	0.000244	0.004042
2000717	ZNF43	10.24792	NM	00342	Homo sapiens	1.636649	-3.079029	2.30955	0.36301	2.027627	-1.081769	1.411145	-4.50301	1.238889	-5.917982	-1.139041	-5.367048	105.7907	0.001312	0.014431
4150154	WDR23	16.18318	NM	02523	Homo sapiens	2.279572	1.973173	2.214491	1.406561	2.027161	0.29945	-1.029389	-6.49091	-1.124515	-6.40604	-1.09241	-5.470774	205.4309	0.000174	0.003111
5570730	C12orf31	12.00867	NM	03233	Homo sapiens	1.386174	-3.919172	1.795854	-0.605651	2.026492	0.94663	1.295547	-4.64785	1.461931	-3.262948	1.128428	-5.253642	1751.149	0.000669	0.008683
160196	XRCC6	21.60658	NM	00146	Homo sapiens	1.586254	-0.074218	1.920077	2.772416	2.026432	3.519942	1.210448	-4.762329	1.277496	-4.093798	1.055391	-5.5213	6929.447	4.34E-05	0.001105
70703	ZDHHC23	13.64296	NM	17357	Homo sapiens	1.297507	-4.824138	1.93593	0.3131	2.025203	0.842859	1.492038	-2.809667	1.560841	-2.399176	1.046114	-5.606943	735.9774	0.000381	0.005687
5390736	MRPL12	10.88828	NM	00294	Homo sapiens	1.268322	-5.156643	1.650308	-2.011338	2.023782	0.647766	1.301175	-4.695054	1.595638	-2.224431	1.226305	-4.632163	4551.435	0.001018	0.011875
7160274	NPY1R	16.60422	NM	00090	Homo sapiens	2.076916	0.881195	2.44631	2.48777	2.023069	0.242348	1.177857	-5.80892	-1.026617	-6.76563	-1.209207	-4.830617	498.2096	0.000154	0.002845
1500634	USF1	11.38078	NM	00712	Homo sapiens	1.462339	-2.868017	1.483724	-2.992178	2.020741	1.421247	1.014624	-6.506782	1.381855	-3.792383	1.361939	-3.244674	1021.945	0.000843	0.010286
5900008	ARMC8	12.73288	NM	01539	Homo sapiens	1.692075	-1.185044	1.94602	0.396748	2.020513	0.829645	1.150079	-5.918748	1.194104	-5.843602	1.03828	-5.624483	123.9626	0.000518	0.007113
1190605	C1orf182	16.85884	NM	14462	Homo sapiens	-1.418038	-4.839352	1.862055	-2.426487	2.0185	-1.637184	2.640465	1.408811	2.86231	2.005931	1.084017	-5.563021	54.73687	0.000144	0.002702
4850440	TSPAN12	25.07097	NM	01233	Homo sapiens	3.056303	3.941258	3.156025	4.072097	2.016249	-0.566687	1.032629	-6.490546	-1.515836	-3.662023	-1.565296	-2.639307	995.9297	2.07E-05	0.000635
7400685	HCST	10.80454	NM	01426	Homo sapiens	-1.297061	-5.660931	1.410723	-5.36034	2.016008	-1.89029	1.829793	-2.344942	2.614885	0.902729	1.42906	-4.065706	118.333	0.001052	0.012167
1990437	LOC649418	16.91684	XM	93849	PREDICTED:	2.101353	1.312607	2.34399	2.34168	2.013857	0.480559	1.115467	-6.167905	-1.043447	-6.730766	-1.16393	-5.08005	1310.184	0.000141	0.002673
620630	DPEP1	13.77807	NM	00441	Homo sapiens	1.273282	-4.802713	1.720877	-0.641806	2.013677	1.517229	1.351528	-3.808515	1.581485	-1.666976	1.170146	-4.892083	34.592	0.000364	0.005503
50307	HNRPH2	13.92844	NM	00103	Homo sapiens	1.927917	-0.45281	2.407895	1.786355	2.013395	-0.28743	1.248962	-5.390343	1.044337	-6.737789	-1.195937	-4.991551	430.1054	0.000347	0.005301
3190717	FUSIP1	24.59329	NM	05401	Homo sapiens	1.844138	2.033366	2.184685	4.211281	2.010835	3.036357	1.184665	-5.186651	1.090393	-6.406454	-1.086457	-5.354604	1432.94	2.28E-05	0.000683
510544	DHFR	19.99058	NM	00079	Homo sapiens	1.762188	-0.157947	2.437113	3.725696	2.010464	1.317763	1.383004	-3.562713	1.140891	-6.181712	-1.212214	-4.58454	281.652	6.36E-05	0.001466
2710102	LRP8	18.19959	NM	03330	Homo sapiens	1.61057	-1.34272	2.257102	2.983666	2.01025	1.463314	1.40143	-3.292923	1.248161	-5.165617	-1.122796	-5.233031	2681.596	1.00E-04	0.002068
5420324	LOC643039	12.15509	XM	92640	PREDICTED:	-1.046782	-6.631394	1.177775	-6.367213	2.009487	0.124934	1.232873	-5.407674	2.103494	0.93923	1.706172	-1.326702	15.14465	0.000635	0.008339
7050678	MAPKAPK5	19.26152	NM	13907	Homo sapiens	1.688524	-0.049592	2.122437	2.958723	2.00841	2.170511	1.256977	-4.583573	1.189447	-5.588685	-1.056775	-5.547035	788.8994	7.61E-05	0.001685
2710639	GABRB3	9.667191	NM	00081	Homo sapiens	-1.221402	-6.101173	1.561462	-4.564435	2.007476	-2.175572	1.907173	-2.152609	2.451936	0.024439	1.285639	-4.860897	97.73382	0.001669	0.017464
4640161	LOC644239	12.69432	XM	93206	PREDICTED:	1.563025	-2.757752	2.183376	1.123737	2.006412	0.061682	1.396891	-4.034279	1.283672	-5.262839	-1.088199	-5.491561	453.4797	0.000525	0.007195
4200446	APH1B	10.8401	NM	03130	Homo sapiens	1.107371	-6.401528	1.560129	-3.04615	2.005982	0.174037	1.408859	-3.873069	1.811482	-0.827944	1.28578	-4.27571	145.7214	0.001037	0.012039
6020095	LDB2	18.74053	NM	00129	Homo sapiens	1.467604	-2.382513	2.106987	2.710167	2.005668	2.002694	1.435664	-2.597151	1.366627	-3.609879	-1.050516	-5.573691	8107.19	8.69E-05	0.001863
130523	MRPS34	13.70218	NM	02393	Homo sapiens	1.16638	-5.815885	1.490955	-2.678125	2.005174	1.679733	1.278275	-4.510956	1.719143	-0.239186	1.344892	-3.255658	2076.113	0.000373	0.005607
7560068	ZNF627	14.31995	NM	14529	Homo sapiens	2.326181	1.3175	2.347049	1.158651	2.005001	-0.650385	1.008971	-6.512608	-1.160189	-6.289928	-1.170597	-5.172567	454.1573	0.000306	0.004824
4290360	CNOT1	16.82079	NM	20699	Homo sapiens	2.494699	2.230801	2.45625	1.843555	2.003996	-0.477325	-1.015653	-6.508624	-1.244862	-5.712718	-1.225676	-4.835397	58.54241	0.000145	0.002725
2640187	KCTD3	18.94723	NM	01612	Homo sapiens	1.999855	1.901668	2.129777	2.493225	2.003157	1.639005	1.064965	-6.361409	1.001651	-6.784925	-1.06321	-5.534232	1477.531	8.24E-05	0.001791
110475	FKBP1A	10.94335	NM	05401	Homo sapiens	1.618464	-2.345133	2.003818	0.04444	2.002867	-0.014948	1.238098	-5.390655	1.237511	-5.654542	-1.000475	-5.665029	1663.503	0.000997	0.011666

3180386	OVCA2		9.008538	NM	08082	Homo sapiens	1.309923	-4.860759	1.459052	-3.859678	2.002039	0.279252	1.113845	-6.186295	1.528363	-2.971815	1.37215	-3.567289	20.69245	0.002218	0.021833
1980681	LONRF1		17.66404	NM	15227	Homo sapiens	2.004594	1.203866	2.296121	2.619286	2.001929	0.877864	1.145429	-5.930905	-1.001331	-6.784973	-1.146954	-5.131464	1571.479	0.000115	0.0023
610291	WARS		13.26472	NM	21364	Homo sapiens	1.425022	-3.427479	1.922546	0.505595	2.001495	0.986163	1.349135	-4.039897	1.404537	-3.718697	1.041065	-5.61557	2610.909	0.000432	0.00622
1770193	PSMD3		15.81087	NM	00280	Homo sapiens	1.201611	-5.467178	1.768635	0.002955	2.001297	1.706853	1.471886	-2.370892	1.665511	-0.668849	1.131549	-5.144881	973.5122	0.000194	0.003368
1500280	LOC644245		16.69578	XM	93207	PREDICTED:	1.393273	-3.203058	1.924881	1.504635	2.000067	1.991056	1.381554	-3.191673	1.435517	-2.803101	1.03906	-5.60931	635.3596	0.00015	0.002794
7000703	CPNE8		10.83054	NM	15363	Homo sapiens	1.563428	-3.054122	2.119039	0.307071	1.999178	-0.427209	1.35538	-4.578655	1.278714	-5.438725	-1.059955	-5.590277	159.0055	0.001041	0.012078
7550671	HRSP12		15.95741	NM	00583	Homo sapiens	1.704211	-0.945013	2.240432	2.335552	1.998837	0.864036	1.314644	-4.427109	1.172881	-5.985167	-1.120868	-5.289023	2882.753	0.000186	0.003265
2970181	TADA3L		11.18855	NM	00635	Homo sapiens	1.584766	-2.11441	1.803738	-0.639817	1.997971	0.659907	1.138173	-6.003507	1.260735	-5.263454	1.107684	-5.373603	399.735	0.000907	0.010864
240768	GABPA		24.46842	NM	00204	Homo sapiens	2.873283	3.852662	2.950032	3.941435	1.997905	-0.282147	1.026711	-6.496716	-1.438148	-4.068877	-1.476563	-3.027971	493.846	2.34E-05	0.000693
6940528	XRCC4		10.04566	NM	02255	Homo sapiens	1.762261	-4.143962	3.577959	0.883516	1.997596	-3.607386	2.030323	-2.92812	1.133542	-6.632377	-1.791132	-3.334691	43.02879	0.001425	0.015444
6290040	HAS2		18.55836	XM	93871	PREDICTED:	4.13505	3.714488	2.731528	0.298971	1.997145	-2.590788	-1.513823	-4.518463	-2.070481	-1.943223	-1.367717	-4.578335	1675.53	9.10E-05	0.001927
4490273	AZIN1		21.24516	NM	01587	Homo sapiens	1.419397	-1.807595	1.790068	2.067931	1.995485	3.710183	1.261147	-3.910114	1.405868	-2.068454	1.114754	-5.060628	11014.8	4.72E-05	0.001173
5420184	PLAC9		11.90172	NM	00101	Homo sapiens	-1.138636	-6.313742	1.451834	-4.476986	1.995356	-0.759346	1.653111	-2.461479	2.271985	0.957573	1.374369	-3.92855	395.4218	0.000695	0.008929
4260152	HOMER1		20.56891	NM	00427	Homo sapiens	1.762127	0.417263	2.277106	3.638806	1.995033	1.875819	1.292248	-4.254542	1.132173	-6.169241	-1.141387	-5.048291	1121.936	5.53E-05	0.001319
2650347	PFKFB2		12.60061	NM	00101	Homo sapiens	1.562576	-3.442027	2.527508	1.547936	1.994769	-1.033687	1.617527	-2.906001	1.276591	-5.618824	-1.267068	-4.690117	87.20228	0.000542	0.007381
50133	LSM7		13.71844	NM	01619	Homo sapiens	1.644974	-1.212377	1.943882	0.834805	1.994441	1.126296	1.18171	-5.598085	1.212445	-5.581086	1.026009	-5.643997	10345.98	0.000371	0.005584
4050201	GPATC4		18.82102	NM	18267	Homo sapiens	1.055113	-6.560918	1.614708	-0.929978	1.992773	2.213084	1.530364	-1.371608	1.888682	1.688364	1.234139	-4.138524	1646.805	8.51E-05	0.001834
5220300	BMP2K		13.48216	NM	01759	Homo sapiens	1.795575	-0.527715	2.085548	1.106466	1.992753	0.483999	1.161493	-5.860323	1.109813	-6.45638	-1.046566	-5.607821	457.6552	0.000401	0.005908
780500	COMT		9.871979	NM	00075	Homo sapiens	-1.29534	-5.776353	-1.148657	-6.79957	1.989808	-2.426441	1.1277	-6.307208	2.577477	0.270231	2.285606	-0.666133	144.4908	0.001532	0.01641
6770132	OGFOD1		16.92031	NM	00103	Homo sapiens	1.323168	-3.804811	1.787352	0.84643	1.989188	2.375383	1.350813	-3.314182	1.503353	-1.731591	1.112925	-5.2059	1654.68	0.000141	0.002673
2510184	PTP4A1		20.38712	NM	00346	Homo sapiens	2.058528	2.713615	2.048298	2.438654	1.987872	1.982033	-1.004994	-6.513385	-1.035543	-6.732319	-1.030397	-5.630385	381.1153	5.78E-05	0.001364
1030189	EPB41L2		21.0727	NM	00143	Homo sapiens	1.814477	1.080684	2.222047	3.613427	1.986633	2.081334	1.224622	-4.932228	1.094879	-6.430584	-1.118499	-5.190738	1086.15	4.91E-05	0.00121
6550091	SURF6		14.0827	NM	00675	Homo sapiens	1.406397	-3.626199	2.011829	1.082456	1.986494	0.866334	1.430484	-3.237806	1.41247	-3.650036	-1.012753	-5.660131	792.8935	0.00033	0.005108
2360253	ATE1		18.43891	NM	00704	Homo sapiens	1.805273	-1.085354	2.840438	3.680529	1.985343	-0.276022	1.573412	-2.675062	1.099746	-6.551594	-1.430704	-3.316006	27.24834	9.39E-05	0.001975
3120520	CPVL		16.62522	NM	03131	Homo sapiens	1.334802	-3.880039	1.901583	1.336464	1.983425	1.880835	1.424618	-2.701068	1.485933	-2.23934	1.043039	-5.597641	1604.682	0.000153	0.002833
3290292	PRMT3		14.82124	NM	00578	Homo sapiens	1.590183	-1.468285	1.975952	1.367927	1.981408	1.355501	1.242594	-4.94276	1.246025	-5.161237	1.002761	-5.664774	632.173	0.000261	0.004277
6650215	LYN		17.28042	NM	00235	Homo sapiens	1.923073	1.700834	1.898885	1.278355	1.981398	1.827564	-1.012738	-6.507723	1.030329	-6.747287	1.043454	-5.596934	2485.817	0.000128	0.002488
3290040	DNAJB6		17.29758	NM	00549	Homo sapiens	1.304856	-5.108942	2.603852	2.659547	1.980854	-0.433958	1.995509	0.097848	1.518063	-3.433599	-1.31451	-4.200816	25.98448	0.000127	0.002481
6650482		0	11.84452	CB066794		iq31f03.x1 HR	-1.218836	-6.376146	3.152179	-0.758915	1.979442	-4.130795	3.841988	0.923337	2.412614	-2.398185	-1.592459	-4.285032	26.149	0.00071	0.009063
6450487	PTPN2		19.26374	NM	00282	Homo sapiens	1.173281	-5.606088	1.828625	1.142951	1.978764	2.246392	1.558557	-0.979483	1.686522	0.107913	1.082105	-5.412894	859.8403	7.61E-05	0.001685
2490376	AFG3L2		16.94545	NM	00679	Homo sapiens	1.293469	-3.960524	1.639022	-0.119623	1.978635	2.788357	1.267152	-4.151907	1.529712	-1.075433	1.207205	-4.243803	1621.669	0.00014	0.002659
6480593	KIAA0020		17.91298	NM	01487	Homo sapiens	1.319147	-3.643787	1.755013	0.964991	1.978456	2.73962	1.330415	-3.347605	1.499799	-1.456174	1.127317	-5.044827	4570.044	0.000108	0.002182
1440445	UIP1		12.56403	NM	20710	Homo sapiens	-1.146934	-6.264532	1.533905	-3.78969	1.977496	-0.790965	1.759287	-1.673496	2.268057	1.018969	1.289191	-4.468674	65.06101	0.000549	0.007448
6590730	TPM3		12.58468	NM	15364	Homo sapiens	1.379763	-3.780985	1.803542	-0.180109	1.976776	1.027202	1.307139	-4.380592	1.432692	-3.316211	1.096052	-5.401931	416.1548	0.000545	0.00741
4760379	LOC645625		19.54961	XM	93520	PREDICTED:	1.916138	1.322752	2.258202	3.214477	1.974443	1.431747	1.178517	-5.542952	1.030428	-6.749749	-1.143716	-5.067562	4712.352	7.08E-05	0.001601
2470291	FKBP2		8.926056	NM	00447	Homo sapiens	1.494917	-3.142929	1.584684	-2.695171	1.973924	0.168666	1.060048	-6.415104	1.320424	-4.83019	1.245627	-4.524889	372.9522	0.002301	0.022443
7550291	ST7		12.82883	NM	02190	Homo sapiens	-1.010814	-6.686127	1.635809	-2.451387	1.973142	-0.081342	1.653499	-1.848072	1.99448	0.324533	1.206218	-4.853165	333.3506	0.000501	0.006946
4040612	CDC42		15.139	NM	00179	Homo sapiens	1.79966	0.372457	1.940746	1.156285	1.971071	1.318013	1.078396	-6.300246	1.095247	-6.473282	1.015625	-5.656746	2887.058	0.000237	0.003956
1440397	BUB1		25.77489	NM	00433	Homo sapiens	2.005821	2.625671	2.423858	4.835421	1.970765	2.120844	1.208412	-5.071815	-1.017788	-6.770805	-1.229907	-4.16405	8920.938	1.80E-05	0.000574
6290209	AKAP1		11.76269	NM	00348	Homo sapiens	1.605938	-2.949229	2.36046	1.148368	1.970408	-0.914677	1.469833	-3.827181	1.226951	-5.890978	-1.197955	-5.036337	682.4225	0.000732	0.009252
1940240	RPS21		15.41847	NM	00102	Homo sapiens	1.476708	-2.032837	1.788048	0.749788	1.970153	2.128329	1.210834	-5.041328	1.334152	-3.80853	1.101845	-5.293073	8299.913	0.000218	0.003697
4730068	LOC285141		11.30397	XM	93914	PREDICTED:	2.059589	-0.515207	2.363141	0.641728	1.968487	-1.372026	1.147384	-6.142602	-1.046281	-6.743279	-1.200486	-5.08879	692.9703	0.000868	0.010515
1450575	C8orf38		19.84171	NM	15241	Homo sapiens	1.41647	-2.273099	1.90732	2.429727	1.967559	2.844325	1.34653	-3.041273	1.389058	-2.706462	1.031583	-5.619099	821.3926	6.59E-05	0.001507
2680068	LOC647523		16.09444	XM	93732	PREDICTED:	1.714892	-0.643106	2.192194	2.346222	1.96729	0.911093	1.278328	-4.692726	1.14718	-6.148812	-1.114322	-5.306191	5655.079	0.000179	0.003171
6370100		0	12.47465	BX106644		BX106644 So	1.511719	-2.549068	1.092088	-6.813004	1.966411	0.793951	-1.384246	-3.666647	1.300779	-4.757422	1.800598	-0.145522	15.57738	0.000566	0.007629
6480438	NALP2		13.98298	NM	01785	Homo sapiens	1.124329	-6.170461	1.553237	-2.048504	1.966124	1.389837	1.381479	-3.386823	1.748709	-0.007752	1.265824	-4.012809	4266.237	0.000341	0.00523
3450541	CHORDC1		17.96302	NM	01212	Homo sapiens	1.611575	-0.657904	2.026997	2.494165	1.965855	2.020948	1.257774	-4.522028	1.219834	-5.215361	-1.031102	-5.627285	1917.626	0.000106	0.002163
4830156	ZNF283		12.37707	XM	94099	PREDICTED:	1.610717	-1.330808	1.74901	-0.418561	1.962723	1.155291	1.085858	-6.265789	1.21854	-5.461393	1.122191	-5.235659	131.6498	0.000586	0.007843
3140131	TBCC		17.64093	NM	00319	Homo sapiens	1.45172	-2.137768	1.896741	1.898661	1.961355	2.339586	1.306547	-3.783965	1.351056	-3.456795	1.034066	-5.61651	832.8553	0.000116	0.00231
4900685	FLJ20323		18.25766	NM	01900	Homo sapiens	1.389304	-2.653827	1.811323	1.588816	1.960945	2.743517	1.303763	-3.632905	1.411459	-2.448665	1.082604	-5.37598	1778.823	9.85E-05	0.002042
6220376	ELOVL6		13.33174	NM	02409	Homo sapiens	1.594136	-1.95661	2.059712	1.202369	1.960873	0.519024	1.292055	-4.681819	1.230054	-5.515122	-1.050406	-5.594541	1521.308	0.000422	0.006133
4390561	HSPA4		18.81451	NM	00215	Homo sapiens	1.194288	-5.436088	1.908222	1.536887	1.960682	1.874273	1.59779	-0.767491	1.641716	-0.520455	1.027492	-5.636084	6054.561	8.52E-05	0.001836
5260484	LARP2		29.02077	NM	03223	Homo sapiens	1.105746	-6.082149	1.881866	3.055193	1.958741	3.628373	1.701897	1.707489	1.77142	2.258104	1.04085	-5.575169	2280.14	9.81E-06	0.000367
3290669	MGAT2		20.17213	NM	00240	Homo sapiens	1.833207	0.759096	2.331586	3.653773	1.958738	1.371504	1.271862	-4.576499	1.068476	-6.612822	-1.190351	-4.687523	784.4823	6.08E-05	0.001415
1820209	ARF3		11.17157	NM	00165	Homo sapiens	1.471017	-2.697576	1.599189	-1.754003	1.957314	1.123642	1.087132	-6.258486	1.330586	-4.280702	1.223941	-4.447865	3408.707	0.000913	0.01091
2140221	PRO1853		21.35817	NM	14473	Homo sapiens	1.36945	-2.748685	1.928876	2.82529	1.957111	2.997313	1.408504	-2.095465	1.429122	-2.037771	1.014638	-5.654486	2681.579	4.60E-05	0.001155
2940722	CDK5RAP3		21.34903	NM	17609	Homo sapiens	2.424804	3.863502	2.117943	2.073098	1.956253	0.983748	-1.144886	-5.88228	-1.239514	-5.287709	-1.082653	-5.462302	409.2218	4.61E-05	0.001155
1230348	DIPA		12.61504	NM	00684	Homo sapiens	1.08503	-6.44746	1.397272	-3.872193	1.955021	1.027955	1.287773	-4.523527	1.801813	0.165819	1.399169	-2.890274	545.343	0.000539	0.007359
6060707	CSTB		20.98207	NM	00010	Homo sapiens	1.444538	-1.875486	1.983554	3.071658	1.954511	2.812401	1.373141	-2.655469	1.353035	-3.145983	-1.01486	-5.654523	6233.022	5.02E-05	0.001229
3520242	PGBD5		13.63757	NM	02455	Homo sapiens	1.210751	-5.584007	1.89299	0.147755	1.952845	0.507951	1.563484	-2.06711	1.612921	-1.834567	1.031619	-5.636393	438.4479	0.000381	0.005693
6900068	WDR74		15.23009	NM	01809	Homo sapiens	1.357393	-3.123682	1.577194	-0.788435	1.948756	2.556335	1.161928	-5.454878	1.43566	-2.205368	1.235585	-3.934579	1651.728	0.000231	0.00386
130092	PGAM4		17.96325	NM	00102	Homo sapiens	1.884731	1.332816	2.069324	2.36613	1.948498	1.499195	1.097941	-6.165096	1.033833	-6.739193	-1.06201	-5.53239	22102.65	0.000106	0.002163
1410471	FLJ20551		10.76851	NM	01787	Homo sapiens	1.3675	-3.837417	1.566403	-2.135313	1.946162	0.960868	1.145451	-5.871569	1.423153	-3.33157	1.24244	-4.307086	888.6714	0.001067	0.012301
1300719	LOC644131		13.97588	XM	92972	PREDICTED:	1.749954	-1.392501	2.392359	2.028486	1.944978	-0.433393	1.367098	-4.343148	1.111445	-6.491528	-1.230019	-4.727869	1734.173	0.000341	0.005237
3440615	ATP11C		13.05912	NM	17369	Homo sapiens	-1.02572	-6.671642	1.69259	-2.057599	1.944947	-0.331196	1.736122	-1.283593	1.99497	0.257	1.149095	-5.210065	174.1829	0.000463	0.006537
6420278	SEMA6A		12.7529	NM	02079	Homo sapiens	1.137545	-6.105702	1.583386	-1.985198	1.944545	0.931632	1.391932	-3.437981	1.709423	-0.60612	1.228093	-4.436441	17579.88	0.000514	0.007076
2350730	CMTM7		12.14999	NM	13841	Homo sapiens	2.393586	1.371053	2.010147	-0.783033	1.944447	-1.218933	-1.190752	-5.880787	-1.230985	-5.896762	-1.033788	-5.643272	204.5888	0.000636	0.008348
60259	PPARGC1B		12.61257	NM	13326	Homo sapiens	-1.074937	-6.561994	1.689216	-2.454021	1.943636	-0.788339	1.815801	-1.122192	2.089286	0.336753	1.150614	-5.248223	172.4128	0.00054	0.007363
4540575	ARL4		14.53726	NM	00573	Homo sapiens	2.107795	1.811386	1.841047	-0.195397	1.942615	0.472712	-1.144889	-5.936144	-1.08503	-6.56627	1.055169	-5.57962	1191.492	0.000286	0.004598
510719	LOC653206		17.72267	XM	93267	PREDICTED:	1.243142	-4.510236	1.631355	0.063147	1.941486	2.818488	1.312284	-3.412245	1.561757	-0.47308	1.190106	-4.356941	665.5767	0.000113	0.002274
1980128	FLJ20512		11.04923	NM	01785	Homo sapiens	1.270123	-4.859064	1.532894	-2.443215	1.941228	0.956736	1.206886	-5.328686	1.528378	-2.23304	1.266382	-4.085303	3795.557	0.000957	0.011316
3400605	PSMD13		15.26534	NM	00281	Homo sapiens	1.216274	-5.087057	1.595869	-0.904117	1.939786	2.099988	1.312097	-3.75121	1.59486	-0.679582	1.215505	-4.257396	899.9016	0.000228	0.003828
1740609	MRPL30		16.29141	NM	14521	Homo sapiens	1.5027	-1.704853	1.888628	1.619419	1.939688	1.958484	1.256823	-4.487211	1.290802	-4.31907	1.027035	-5.635592	1148.645	0.000169	0.003037
3060091	DLG1		22.34567	NM	00408	Homo sapiens	2.15836	2.637876	2.443842	3.913595	1.939578	0.976331	1.132268	-5.964877	-1.112799	-6.369627	-1.259987	-4.133784	100.6781	3.68E-05	0.000969
4480377	WDR4		11.48916	NM	01866	Homo sapiens	-1.543478	-4.55592	1.467806	-5.334602	1.939432	-2.933211	2.265526	-1.012705	2.993471	1.174413	1.321314	-4.803319	74.3493	0.00081	0.010002
3120286	PPP3R1		16.14053	NM	00094	Homo sapiens	1.505082	-0.989669	1.571023	-0.521098	1.938996	2.895296	1.043812	-6.412593	1.288299	-3.933532	1.234226	-3.824864	3185.37	0.000176	0.003136
5360403	PIP5K2A		13.75714	NM	00502	Homo sapiens	-1.185392	-5.942251	1.360295	-4.886479	1.937719	-0.388354	1.612483	-2.231886	2.296957	1.871573	1.424484	-3.277832	190.809	0.000367	0.00553

7610438	SLCO3A1		14.49231	XM	94106	PREDICTED:	1.191034	-5.649074	1.8269	0.142879	1.936571	0.901368	1.533877	-1.985053	1.625958	-1.319745	1.060031	-5.553435	316.7672	0.00029	0.004639
7570403	MID1IP1		15.63018	NM	02124	Homo sapiens	1.518582	-1.528115	1.83959	1.2314	1.936163	1.93433	1.211387	-5.020026	1.274981	-4.513139	1.052497	-5.557512	3006.634	0.000205	0.003519
6250162	CARD10		16.50296	NM	01455	Homo sapiens	1.160703	-5.700318	1.618952	-0.615904	1.936146	2.130811	1.394803	-2.698681	1.66808	0.100959	1.195925	-4.443406	1003.138	0.000159	0.002904
2710414	MT1F		14.10155	NM	00594	Homo sapiens	1.27531	-4.478022	1.640763	-0.683413	1.93572	1.786375	1.28656	-4.186238	1.517843	-1.716505	1.179769	-4.670589	11456.15	0.000328	0.005087
130307	CCNC		21.19899	NM	00519	Homo sapiens	1.247189	-4.415909	1.84304	2.171581	1.935355	2.874245	1.477755	-1.196238	1.551774	-0.505005	1.050089	-5.545953	2286.429	4.77E-05	0.001182
1440368	RTN4IP1		10.16047	NM	03273	Homo sapiens	1.292635	-4.946664	1.733993	-1.3591	1.935254	0.044058	1.341441	-4.33561	1.497139	-3.123839	1.116068	-5.345395	2032.929	0.00136	0.014853
3140452	RTCD1		20.76907	NM	00372	Homo sapiens	1.646208	-0.116964	2.172114	3.673043	1.935247	2.041731	1.319464	-3.671083	1.175579	-5.64978	-1.122396	-5.12794	1831.733	5.28E-05	0.001273
270377	ARHGAP11A		12.15876	NM	01478	Homo sapiens	1.409078	-3.627733	1.900812	0.281597	1.934969	0.466884	1.348976	-4.074818	1.373216	-4.073571	1.01797	-5.655468	204.5932	0.000634	0.008333
10068	ACO2		18.21773	NM	00109	Homo sapiens	1.553891	-0.619717	1.855861	2.024791	1.934942	2.608529	1.194331	-5.035997	1.245224	-4.63041	1.042612	-5.582438	1206.578	9.95E-05	0.002059
840450	LOC440145		14.33631	XM	93394	PREDICTED:	1.977851	-0.27213	2.520341	2.122542	1.934868	-0.885132	1.274282	-5.235008	-1.022215	-6.773242	-1.30259	-4.334004	1001.815	0.000304	0.004804
3140291	SCAMP5		10.61457	NM	13896	Homo sapiens	-1.088243	-6.513785	1.362364	-5.043405	1.932089	-0.818386	1.482582	-3.539333	2.102582	0.452831	1.418189	-3.49906	1074.882	0.001133	0.012913
7560041	MRPL16		19.38044	NM	01784	Homo sapiens	1.412783	-2.127316	1.843326	2.200337	1.93101	2.867721	1.304749	-3.452045	1.366813	-2.824025	1.047568	-5.556666	3842.879	7.39E-05	0.00165
940053	ACOT8		18.08877	NM	18338	Homo sapiens	1.047862	-6.583073	1.527723	-1.542318	1.928703	2.150207	1.457943	-1.896943	1.840607	1.694048	1.262469	-3.719084	364.8134	0.000103	0.002112
2370239	QPCTL		11.05441	NM	01765	Homo sapiens	1.568747	-2.23013	1.866112	-0.146779	1.92867	0.237485	1.189556	-5.61706	1.229433	-5.537452	1.033523	-5.633684	173.3394	0.000955	0.011299
650390	WDR12		16.84827	NM	01825	Homo sapiens	1.526018	-1.384599	1.92366	1.97246	1.92682	1.948573	1.260575	-4.410136	1.262646	-4.632206	1.001643	-5.664921	4504.103	0.000144	0.002707
4850544	PTGES2		13.10816	NM	19893	Homo sapiens	-1.003338	-6.688965	1.343942	-4.514542	1.92619	0.685666	1.348428	-3.950654	1.932619	0.989428	1.433239	-2.660293	819.6469	0.000455	0.006452
4850008	C9orf77		10.91541	NM	01601	Homo sapiens	2.013961	-1.101336	2.504297	0.808204	1.925417	-1.953918	1.243469	-5.694437	-1.045987	-6.747446	-1.300652	-4.629057	240.3307	0.001007	0.011768
6450678	NUDT21		11.73696	NM	00700	Homo sapiens	1.987378	0.170219	2.023636	0.104148	1.925039	-0.561882	1.018244	-6.505741	-1.032383	-6.757758	-1.051218	-5.605393	3781.566	0.000739	0.009312
2690692	SOX11		16.44854	XM	93807	PREDICTED:	1.012963	-6.681897	1.419471	-3.086873	1.9248	1.71539	1.401306	-2.81518	1.900168	1.769278	1.355998	-2.890972	1012.367	0.000161	0.002938
3120647	ARHGAP27		17.5316	NM	19928	Homo sapiens	1.200536	-5.283957	1.791148	0.912135	1.924556	1.937772	1.491958	-1.632261	1.603081	-0.633646	1.074482	-5.451282	451.5305	0.000119	0.00236
2190475	DHRS8		12.63822	NM	01624	Homo sapiens	3.190415	1.403817	2.771292	0.015435	1.923452	-3.226612	-1.151237	-6.271607	-1.658692	-4.185157	-1.44079	-4.352787	196.7097	0.000535	0.007308
6760154	RSL1D1		13.47789	NM	01565	Homo sapiens	1.514593	-2.057897	1.851009	0.670299	1.922933	1.159699	1.222117	-5.091507	1.269604	-4.834955	1.038856	-5.613122	5627.056	0.000402	0.005911
5090204	C2orf25		18.35671	NM	01570	Homo sapiens	1.827385	1.14059	2.068059	2.629586	1.922762	1.572779	1.131704	-5.881618	1.052193	-6.672288	-1.075567	-5.460819	11703.21	9.59E-05	0.002006
4760196	UEV3		16.86837	NM	01831	Homo sapiens	1.673338	-1.085094	2.322791	2.916719	1.921695	0.484733	1.388118	-3.628645	1.14842	-6.154538	-1.20872	-4.660919	154.8954	0.000143	0.002698
4670370	SLC7A6OS		12.08458	NM	03217	Homo sapiens	1.283203	-4.753359	1.738868	-0.622742	1.920981	0.729217	1.3551	-3.84272	1.49702	-2.604798	1.104731	-5.35021	344.3068	0.000651	0.008498
5360553	C14orf129		20.20619	NM	01647	Homo sapiens	1.703764	0.610341	2.079635	3.313154	1.92006	2.136483	1.220613	-4.816202	1.126952	-6.112466	-1.08311	-5.389568	2363.214	6.03E-05	0.001407
4200709		0	12.87949	BC090942		Homo sapiens	1.393554	-3.498056	1.839849	0.343968	1.918886	0.881245	1.320257	-4.135613	1.376973	-3.76226	1.042958	-5.605256	746.781	0.000492	0.006863
1050086	PEO1		16.13325	NM	02183	Homo sapiens	1.24865	-4.545034	1.630646	-0.193539	1.918373	2.350171	1.305926	-3.637015	1.536357	-0.988143	1.17645	-4.566158	1360.647	0.000177	0.003141
2510088	RANBP6		12.34936	NM	01241	Homo sapiens	1.689102	-0.671318	1.793815	-0.097812	1.916026	0.776232	1.061993	-6.382061	1.134346	-6.218332	1.068129	-5.522173	1071.419	0.000592	0.007896
4260082	KIAA0179		13.22117	NM	01505	Homo sapiens	1.638688	-1.328112	1.994608	1.101095	1.914975	0.506056	1.217198	-5.29705	1.168602	-5.984443	-1.041585	-5.613111	1597.936	0.000438	0.006273
2900634	GPRC5B		12.40664	NM	01623	Homo sapiens	1.384984	-3.867688	1.9336	0.511988	1.914915	0.330027	1.396117	-3.605147	1.382626	-3.977598	-1.009757	-5.662188	670.4356	0.00058	0.00778
2970358	CDV3		18.42177	XM	94527	PREDICTED:	2.048128	2.630997	1.920196	1.526706	1.912561	1.420517	-1.066624	-6.338752	-1.070882	-6.585467	-1.003992	-5.664421	4574.139	9.43E-05	0.001983
4060338	LOC653170		9.969852	XM	92632	PREDICTED:	1.536638	-5.079277	3.46219	0.719677	1.911539	-3.910815	2.253094	-2.083404	1.243975	-6.326219	-1.811205	-3.234933	23.76231	0.001471	0.015861
2940025	GTF3C1		13.52863	NM	00152	Homo sapiens	1.598427	-1.552961	1.982779	1.201292	1.911152	0.652761	1.240456	-5.027133	1.195646	-5.713929	-1.037478	-5.620958	2586.55	0.000395	0.005839
6280750	RRS1		25.22532	NM	01516	Homo sapiens	1.121688	-5.896099	1.774161	2.181636	1.910136	3.326025	1.581688	0.522822	1.702912	1.676287	1.076642	-5.36089	2843.323	2.01E-05	0.000621
1010750	POLR3E		14.81936	NM	01811	Homo sapiens	1.484905	-1.938504	1.814241	0.973509	1.910074	1.68685	1.221789	-4.915675	1.286327	-4.394973	1.052823	-5.55738	2089.534	0.000262	0.004278
6350376	HSPA8		19.04757	NM	00659	Homo sapiens	1.455587	-1.312475	1.767969	1.883484	1.908617	3.061119	1.214609	-4.568923	1.311235	-3.404989	1.079554	-5.35453	44676.4	8.03E-05	0.001756
5050731	HSGT1		16.12002	XM	93918	PREDICTED:	2.039428	1.869423	1.978646	1.231673	1.907427	0.682681	-1.030719	-6.480917	-1.069204	-6.62122	-1.037338	-5.620721	411.6291	0.000177	0.003151
3190020	ARHGAP12		19.49151	NM	01828	Homo sapiens	2.00892	2.324206	2.09683	2.677367	1.907295	1.327664	1.04376	-6.437247	-1.053282	-6.670897	-1.099374	-5.333396	1212.895	7.19E-05	0.001618
5260243	RBM11		11.23941	NM	14477	Homo sapiens	2.228218	0.542767	2.130318	-0.221304	1.906909	-1.523537	-1.045956	-6.471319	-1.168497	-6.281236	-1.117157	-5.433147	87.85349	0.00089	0.010722
2340392	GOSR2		11.87302	NM	05402	Homo sapiens	1.322692	-4.007725	1.5461	-1.822982	1.906553	1.353883	1.168904	-5.555005	1.441419	-2.714039	1.233137	-4.211698	867.0144	0.000703	0.008986
5390162	RTEL1		11.94387	NM	03295	Homo sapiens	-1.318897	-5.25967	1.257528	-6.054285	1.905956	-1.631802	1.65855	-2.68464	2.51376	1.588047	1.515637	-3.173716	77.89917	0.000685	0.008845
4810609	LOC645001		11.04066	XM	92806	PREDICTED:	2.149157	-0.236673	2.362995	0.45136	1.90437	-1.897533	1.099498	-6.342145	-1.128539	-6.504796	-1.240827	-4.909558	5239.137	0.00096	0.011346
990048	UQCR		12.48514	NM	00683	Homo sapiens	1.242414	-4.9794	1.59868	-1.380751	1.903198	1.206378	1.286753	-4.320847	1.531856	-1.815789	1.190481	-4.639611	615.3321	0.000564	0.00761
7050291	LOC649150		11.70127	XM	94079	PREDICTED:	1.805023	-0.394295	1.913011	0.080568	1.902973	-0.042111	1.059826	-6.409627	1.054265	-6.697399	-1.005275	-5.664253	51828.1	0.000748	0.009409
2060753	PRDM5		12.84153	NM	01869	Homo sapiens	1.136335	-6.438969	2.74706	0.892526	1.902494	-2.661137	2.417474	0.114679	1.674237	-3.596899	-1.443926	-4.063456	80.61472	0.000499	0.00693
5360139	LOC642646		11.37913	XM	92610	PREDICTED:	-1.084461	-6.504484	1.113504	-6.753632	1.901869	-0.463002	1.207552	-5.580738	2.062503	0.811435	1.708003	-1.249002	23.26497	0.000844	0.01029
2100632	MRPL4		10.92872	NM	01595	Homo sapiens	1.079287	-6.502266	1.507149	-3.106136	1.90156	0.095209	1.396431	-3.676959	1.761868	-0.652714	1.261694	-4.293718	387.8935	0.001002	0.01171
5310692	CTDP1		14.89183	NM	00471	Homo sapiens	1.45316	-1.684639	1.550777	-0.818724	1.900926	2.51715	1.067175	-6.286398	1.308133	-3.695393	1.22579	-3.947343	570.8779	0.000256	0.004201
6960564	C1orf25		14.80718	NM	03093	Homo sapiens	1.605893	-1.33117	2.050714	1.848114	1.89841	0.753573	1.276992	-4.588563	1.182152	-5.79726	-1.080227	-5.465637	637.7452	0.000263	0.004292
5870402	LOC653566		12.65988	XM	93479	PREDICTED:	1.750515	-0.790148	2.056236	1.02073	1.898233	-0.060506	1.174646	-5.750044	1.084385	-6.580299	-1.083237	-5.487079	2288.495	0.000531	0.007261
2450243	SHB		8.387802	NM	00302	Homo sapiens	2.394937	0.090068	1.77734	-3.163798	1.89819	-2.55575	-1.347484	-5.2227	-1.261695	-5.960217	1.067994	-5.602327	212.1674	0.002938	0.027072
70228	PNPT1		17.81668	NM	03310	Homo sapiens	1.475435	-1.666962	1.912256	2.291078	1.896998	2.124818	1.296062	-3.793057	1.285721	-4.169069	-1.008043	-5.662083	416.1251	0.000111	0.002226
6590619		0	13.33907	BM352836		ig67g12.y1 HF	-1.454915	-4.703426	1.617139	-4.054801	1.895387	-2.495566	2.3528	0.115967	2.757627	1.409127	1.172062	-5.302321	168.2628	0.000421	0.00612
6250064	RSU1		12.90782	NM	15272	Homo sapiens	1.237311	-5.203927	1.792822	-0.129895	1.89529	0.600301	1.448966	-2.834758	1.531781	-2.218954	1.057155	-5.563603	1546.353	0.000487	0.006808
2060373	LOC653573		10.85517	XM	93004	PREDICTED:	2.016822	-0.494642	2.244692	0.383352	1.893974	-1.56992	1.112985	-6.271506	-1.064862	-6.699417	-1.185175	-5.131517	604.8899	0.001031	0.011986
7210037	C1orf109		10.56697	NM	01785	Homo sapiens	1.64115	-3.077824	2.4418	0.883492	1.893561	-1.879154	1.487859	-3.983458	1.153802	-6.387491	-1.289528	-4.623211	38.91338	0.001155	0.013095
7610484	DNAJB14		17.71829	NM	00103	Homo sapiens	2.406659	1.643953	2.782107	2.856374	1.892078	-1.351292	1.156004	-6.049123	-1.271966	-5.568348	-1.470398	-3.2749	188.8315	0.000114	0.002275
110079	PDCD2		16.3386	NM	00259	Homo sapiens	1.532676	-0.954538	1.79884	1.42008	1.892066	2.149518	1.173659	-5.314638	1.234485	-4.81647	1.051826	-5.547398	1233.517	0.000167	0.003006
4890274	PLXDC2		14.84764	NM	03281	Homo sapiens	1.017283	-6.679086	1.709413	-1.037772	1.891498	0.330436	1.680371	-0.859044	1.859362	0.36692	1.106519	-5.353342	651.9803	0.000259	0.004251
6130142	RFP		11.88321	NM	00651	Homo sapiens	1.194781	-5.631504	1.677196	-1.166054	1.891323	0.499287	1.403769	-3.343255	1.582988	-1.779677	1.12767	-5.214762	734.4396	0.0007	0.008966
6770196	MRPL30		14.26635	NM	14521	Homo sapiens	1.519652	-1.956312	1.935608	1.37077	1.890488	0.988255	1.273718	-4.514581	1.244026	-5.092131	-1.023867	-5.644794	950.871	0.000311	0.004879
780296	HSC20		10.75731	NM	17200	Homo sapiens	1.358298	-3.913613	1.661444	-1.209068	1.889674	0.599929	1.223181	-5.167753	1.391207	-3.645958	1.137368	-5.138992	622.9113	0.001071	0.012346
5670612	FAM54A		13.18112	NM	13841	Homo sapiens	1.418797	-3.361398	1.966119	1.031201	1.886907	0.421303	1.385765	-3.554744	1.329934	-4.378512	-1.04198	-5.610721	301.7475	0.000444	0.006342
5360504	HIST1H2BJ		12.79555	NM	02105	Homo sapiens	-1.814519	-3.234028	1.233271	-6.524517	1.885508	-3.298096	2.237793	-1.246541	3.42129	2.051066	1.528868	-3.910834	59.94172	0.000507	0.007005
5260201	GCNT2		14.82555	NM	00149	Homo sapiens	1.09655	-6.303191	1.492258	-2.105939	1.884576	1.597668	1.360867	-3.190141	1.718642	0.453037	1.262902	-3.784948	1700.395	0.000261	0.004273
110114	KIAA1279		11.43868	NM	01563	Homo sapiens	1.779638	-1.189356	2.143144	0.776032	1.883727	-0.841293	1.204259	-5.658026	1.058489	-6.698864	-1.137715	-5.280675	3165.528	0.000825	0.010143
3840411	MTHFD1		13.14727	NM	00595	Homo sapiens	1.179704	-5.78139	1.803752	-0.183111	1.883256	0.365242	1.528987	-2.140449	1.59638	-1.713698	1.044077	-5.605712	1617.344	0.000449	0.006392
5570524	CASP3		18.09798	NM	03299	Homo sapiens	-1.003115	-6.68891	1.557003	-1.379506	1.882568	1.586735	1.561853	-0.898707	1.888433	1.870294	1.209098	-4.341627	6052.938	0.000103	0.002109
3170192	LEMD3		16.57321	NM	01431	Homo sapiens	1.501111	-2.26335	2.154235	2.624818	1.881056	0.755834	1.435094	-2.817906	1.253109	-5.047641	-1.145227	-5.05674	2203.82	0.000156	0.002857
6350762	VPS18		11.17352	NM	02085	Homo sapiens	1.060072	-6.565895	1.292142	-5.02446	1.88091	0.449842	1.218919	-5.23201	1.774323	-0.094843	1.455653	-2.422648	1106.269	0.000912	0.010907
5270368	ZNF701		9.820706	NM	01826	Homo sapiens	1.36216	-3.844962	1.523582	-2.487634	1.880373	0.584079	1.118504	-6.060496	1.380435	-3.732806	1.234179	-4.354442	263.5153	0.001565	0.016637
5310427	SEC63		11.13344	NM	00721	Homo sapiens	1.597404	-1.472272	1.720703	-0.678133	1.880141	0.540931	1.077187	-6.312076	1.176998	-5.859669	1.092659	-5.408163	948.0746	0.000926	0.011046
4070541	PDE3B		17.11022	NM	00092	Homo sapiens	1.810203	0.849842	2.057456	2.387145	1.878935	1.075999	1.136589	-5.861702	1.037969	-6.726415	-1.095012	-5.362168	393.437	0.000134	0.002576
7210653	CDKN3		16.72685	NM	00519	Homo sapiens	1.269399	-4.348379	1.795786	1.210982	1.8775	1.843333	1.414674	-2.354224	1.479046	-1.789963	1.045503	-5.577096	2808.088	0.000149	0.002776
4670148	SLC33A1		17.57042	NM	00473	Homo sapiens	1.652602	-0.154722	2.03279	2.661609	1.875463	1.46456	1.230054	-4.805648	1.134854	-6.083255	-1.083887	-5.402875	602.0626	0.000118	0.002344
610156	CXorf39		16.9729	NM	20731	Homo sapiens	1.339702	-3.37264	1.832392	1.645597	1.875336	1.951302	1.367761	-2.864901	1.399816	-2.67786	1.023436	-5.64041	954.4819	0.000139	0.002643

5490452	WDR20	15.90474	NM	18130	Homo sapiens	1.725993	-0.058443	2.066889	2.200029	1.874847	0.811618	1.197507	-5.338556	1.086243	-6.515325	-1.102431	-5.333942	322.5711	0.000189	0.003306
5220441	APLP2	17.1362	NM	00164	Homo sapiens	1.687653	0.59234	1.857987	1.843155	1.874729	1.931337	1.10093	-6.061466	1.11085	-6.242213	1.00901	-5.661347	1653.71	0.000133	0.002558
6040170	DKFZp686I1521	10.48054	NM	20749	Homo sapiens	1.784597	-1.060714	1.99062	0.00377	1.87457	-0.800453	1.115445	-6.196929	1.050416	-6.718725	-1.061907	-5.57698	101.7338	0.001195	0.013443
2710343	GIYD1	13.17423	NM	00101	Homo sapiens	1.126162	-6.179868	1.693758	-0.895597	1.874033	0.513439	1.504009	-2.232025	1.664089	-0.921054	1.106435	-5.331592	164.4738	0.000445	0.006349
4070463	ATG4C	11.08864	NM	03285	Homo sapiens	1.69112	-1.684888	2.078491	0.608868	1.873678	-0.732432	1.229062	-5.432784	1.107951	-6.496816	-1.109311	-5.40211	1376.618	0.000942	0.011187
2340553	KIAA0133	12.96545	NM	01477	Homo sapiens	1.22183	-5.165707	1.640656	-0.85674	1.872666	1.106563	1.342786	-3.614719	1.532673	-1.707471	1.141413	-5.032222	783.3483	0.000478	0.006708
2750671	CPSF6	10.66748	NM	00700	Homo sapiens	1.859823	-0.831767	2.084184	0.23038	1.872426	-1.11301	1.120635	-6.194675	1.006776	-6.783868	-1.113092	-5.409671	731.7352	0.00111	0.012694
2650170	EXOSC4	11.9323	NM	01903	Homo sapiens	1.116817	-6.226531	1.49701	-2.632692	1.872425	0.703165	1.340427	-3.840135	1.676574	-0.635671	1.250776	-4.154684	1204.56	0.000688	0.00887
5670711	CDCP1	14.56994	NM	17818	Homo sapiens	-1.048312	-6.622332	1.755551	-1.225324	1.87164	-0.428492	1.840366	-0.194257	1.962063	0.452567	1.066127	-5.555032	23.32176	0.000283	0.004566
20605	15E1.2	11.18231	NM	17681	Homo sapiens	1.280972	-4.841399	1.759321	-0.615519	1.87057	0.18875	1.373427	-3.740841	1.460274	-3.090766	1.063234	-5.548114	495.5685	0.000909	0.010883
2750598	SEPHS2	16.9974	NM	01224	Homo sapiens	1.171256	-5.459118	1.631126	0.007695	1.867584	2.169943	1.39263	-2.376254	1.594514	-0.160583	1.144966	-4.842419	1388.273	0.000138	0.002629
4860255	CDC6	12.84992	NM	00125	Homo sapiens	1.067235	-6.521216	1.483693	-2.703902	1.866464	0.757176	1.390222	-3.238189	1.748879	0.080418	1.257985	-4.057394	190.9596	0.000497	0.006915
6330411	SMARCD1	9.221551	NM	00307	Homo sapiens	1.472366	-4.475701	2.399728	0.195586	1.864307	-2.452536	1.629845	-3.31178	1.266198	-5.863422	-1.287196	-4.740688	71.53429	0.00202	0.020336
4200180	AES	10.84096	NM	19896	Homo sapiens	1.564074	-2.197206	1.88026	0.05415	1.863527	-0.120091	1.202156	-5.491472	1.191457	-5.842711	-1.008979	-5.662668	6170.05	0.001037	0.012038
1580035	ACY1	18.01234	NM	00066	Homo sapiens	1.340399	-3.115272	1.808292	1.879936	1.863476	2.305801	1.34907	-2.853201	1.390239	-2.517191	1.030517	-5.619571	2720.567	0.000105	0.002142
2230731	BCOR	11.6773	NM	01774	Homo sapiens	1.658263	-1.892002	2.137962	1.011028	1.863195	-0.746056	1.289279	-4.919939	1.123582	-6.409847	-1.147471	-5.205183	4608.521	0.000755	0.009463
5570717	ATP5G3	15.40276	NM	00100	Homo sapiens	1.266686	-4.648071	1.875207	1.201485	1.862772	1.054123	1.480405	-2.042814	1.470587	-2.362424	-1.006676	-5.663319	385.4439	0.000219	0.003709
650538	DKFZp667M241	9.602391	NM	20732	Homo sapiens	2.337709	0.3592	2.007549	-1.492364	1.862292	-2.353366	-1.164459	-6.105022	-1.255286	-5.898292	-1.077999	-5.572973	65.97319	0.001715	0.017831
3930551	BCAP29	12.16831	NM	00100	Homo sapiens	1.745239	-0.593814	1.94693	0.596733	1.861587	-0.057561	1.115566	-6.126923	1.066666	-6.645995	-1.045844	-5.604795	396.4941	0.000632	0.008313
2030594	SWAP70	16.56364	NM	01505	Homo sapiens	1.567113	-0.815522	1.922752	2.127856	1.861392	1.598583	1.226939	-4.763231	1.187784	-5.482121	-1.032965	-5.619254	1754.741	0.000156	0.002864
4590598	GAP43	16.07483	NM	00204	Homo sapiens	-2.134448	-1.671597	-1.02203	-7.071381	1.860399	-3.323826	2.08844	-1.739575	3.970925	3.291278	1.901383	-2.243907	737.6349	0.00018	0.003182
6650537	BAK1	15.69418	NM	00118	Homo sapiens	1.104965	-6.154478	1.340837	-3.474314	1.86011	2.248416	1.213466	-4.720378	1.683411	0.883345	1.387275	-2.060027	1173.668	0.000201	0.003464
7650368	IGSF9B	9.200153	XM	94066	PREDICTED:	-1.537841	-4.661144	1.202333	-6.64915	1.859383	-3.443784	1.848997	-2.941834	2.859435	0.618608	1.546479	-3.841586	142.1152	0.002039	0.020506
3060360	DMXL2	28.19619	NM	01526	Homo sapiens	4.39182	5.891185	2.511098	1.115915	1.859097	-2.119685	-1.748964	-2.270155	-2.362341	0.716313	-1.350708	-4.275828	38.96759	1.14E-05	0.000406
3870754	FBXO28	14.7575	NM	01517	Homo sapiens	1.5671	-1.423059	1.984913	1.78738	1.858492	0.810049	1.266615	-4.568401	1.185944	-5.68394	-1.068023	-5.507291	2106.834	0.000267	0.004342
1660341	SDHA	10.77587	NM	00416	Homo sapiens	1.503195	-2.279819	1.685368	-0.849402	1.858483	0.531	1.12119	-6.029881	1.236355	-5.242168	1.102717	-5.343327	1322.271	0.001064	0.012275
2490682	FUCA2	13.07652	NM	03202	Homo sapiens	1.6564	-0.78443	1.889793	0.842382	1.858447	0.554118	1.140904	-5.875759	1.12198	-6.287499	-1.016867	-5.655271	1337.214	0.00046	0.006509
670735	MBNL3	20.96011	NM	13348	Homo sapiens	3.044975	3.110115	3.084347	3.030681	1.858161	-2.126059	1.01293	-6.511207	-1.638704	-3.205297	-1.659892	-2.459751	67.14972	5.04E-05	0.001235
6220494	C13orf10	17.7625	NM	02211	Homo sapiens	1.87881	1.216194	2.13907	2.718218	1.858081	0.748457	1.138524	-5.868165	-1.011156	-6.780004	-1.151225	-4.988562	2140.863	0.000112	0.002254
2470653	GNB4	12.47536	NM	02162	Homo sapiens	1.844334	-0.020967	1.987685	0.675308	1.857434	-0.270123	1.077725	-6.337586	1.007103	-6.783414	-1.070124	-5.534094	766.1502	0.000566	0.007629
1050521	TBRG4	10.25835	NM	19912	Homo sapiens	1.327665	-4.316606	1.683294	-1.156671	1.856958	0.194784	1.26786	-4.773225	1.398665	-3.658122	1.103169	-5.363298	3582.093	0.001307	0.0144
7200463	SLC16A1	11.38751	NM	00305	Homo sapiens	1.516434	-3.582035	2.343822	1.147293	1.856644	-1.553532	1.545614	-3.211086	1.224349	-5.894776	-1.262397	-4.643944	322.4749	0.000841	0.010274
520333	MAPK1	14.04094	NM	13895	Homo sapiens	1.325895	-3.579383	1.648354	-0.105702	1.855405	1.738909	1.243201	-4.496172	1.399361	-2.715133	1.125611	-5.06511	1129.523	0.000334	0.00517
2940403	REXO2	14.78673	NM	01552	Homo sapiens	1.218329	-5.218464	1.815842	0.656867	1.855063	0.919731	1.490437	-1.986368	1.52263	-1.850676	1.021599	-5.647649	11729.58	0.000264	0.004313
5050592	0	16.68568	AK026966		Homo sapiens	1.184097	-5.278991	1.625607	0.026342	1.853694	2.142397	1.372866	-2.581508	1.565492	-0.408164	1.140309	-4.87429	10640.57	0.000151	0.002799
4260326	C10orf82	11.1032	NM	14466	Homo sapiens	1.457577	-2.98027	1.825687	-0.010103	1.8529	0.143175	1.25255	-4.933052	1.271219	-4.998764	1.014905	-5.658014	473.6911	0.000937	0.011136
6200397	MGC15763	12.67767	NM	13838	Homo sapiens	1.250814	-4.787524	1.635367	-0.780452	1.852688	1.097085	1.307442	-3.959636	1.481186	-2.157942	1.132888	-5.080487	476.9161	0.000528	0.007228
3180747	CLIC4	19.37043	NM	01394	Homo sapiens	2.027557	1.684968	2.373054	3.380096	1.85267	0.171106	1.170401	-5.685801	-1.094397	-6.497453	-1.280883	-3.999562	1420.059	7.41E-05	0.001652
4180601	IQGAP1	16.01778	NM	00387	Homo sapiens	1.639328	-0.795658	2.099861	2.449972	1.852487	0.678714	1.280928	-4.441907	1.130029	-6.205617	-1.133536	-5.124801	2578.567	0.000183	0.00322
1300736	CENPE	13.86803	NM	00181	Homo sapiens	1.473122	-1.89053	1.736508	0.54029	1.851475	1.483386	1.178794	-5.333873	1.256838	-4.656149	1.066206	-5.489766	2974.703	0.000354	0.005392
1050711	NUP153	20.11368	NM	00512	Homo sapiens	1.924836	1.995536	2.151424	3.284128	1.85142	1.145586	1.117718	-5.984044	-1.039654	-6.71713	-1.16204	-4.829189	3140.021	6.17E-05	0.001431
20349	FKBP4	14.4158	NM	00201	Homo sapiens	1.009007	-6.685761	1.369142	-3.704675	1.851224	1.124215	1.35692	-3.350972	1.834699	1.24355	1.352105	-2.940078	3818.591	0.000297	0.004721
7200379	GEMIN5	17.20624	NM	01546	Homo sapiens	1.671281	-0.615271	2.219026	3.009666	1.849325	0.525287	1.327739	-3.980108	1.106531	-6.393165	-1.199911	-4.613012	503.1885	0.000131	0.002524
4880022	PINK1	15.58256	NM	03240	Homo sapiens	-1.054504	-6.568358	1.372239	-3.752468	1.848392	0.952738	1.447031	-2.401061	1.949135	1.953557	1.34699	-3.057916	1058.393	0.000208	0.003558
1090239	ANXA11	13.49126	NM	14586	Homo sapiens	-1.035654	-6.643015	1.421463	-3.551468	1.847265	0.344377	1.472143	-2.530574	1.913127	1.091832	1.299552	-3.756392	220.6152	0.0004	0.005896
1110632	SCML2	14.54863	NM	00608	Homo sapiens	2.424735	1.552188	2.421735	1.292121	1.846971	-1.774674	-1.001239	-6.514418	-1.312817	-5.316144	-1.311192	-4.379915	1138.138	0.000285	0.004588
3870632	APOL1	9.3758	NM	14534	Homo sapiens	-1.164257	-6.181823	1.17226	-6.520764	1.846778	-1.68514	1.364812	-4.637383	2.150124	0.343245	1.5754	-2.625914	17.26423	0.001889	0.019293
4390438	AIM1	14.45578	NM	00162	Homo sapiens	-1.52725	-3.961891	1.422568	-5.0479	1.845797	-2.318612	2.172616	-0.110282	2.818992	2.234162	1.29751	-4.620629	33.3079	0.000293	0.004678
3130593	NTAN1	13.86476	XM	94289	PREDICTED:	1.865817	0.721669	1.924282	0.877085	1.845777	0.246107	1.031335	-6.47941	-1.010857	-6.78069	-1.042533	-5.607611	1917.66	0.000354	0.005393
3290142	CMAS	13.04588	NM	01868	Homo sapiens	1.502784	-2.229746	1.908609	1.034769	1.845326	0.505356	1.270049	-4.605926	1.227939	-5.306816	-1.034293	-5.625001	3387.055	0.000465	0.00656
7560014	MRPL44	13.59047	NM	02291	Homo sapiens	1.439627	-2.417976	1.768891	0.642467	1.844317	1.227866	1.228715	-4.817182	1.281108	-4.434323	1.04264	-5.593042	2756.277	0.000387	0.005753
4850092	CSNK1A1	13.25734	NM	00189	Homo sapiens	1.679131	-0.610298	1.920921	1.036598	1.843986	0.409858	1.143997	-5.855317	1.098179	-6.453625	-1.041722	-5.607432	982.5201	0.000433	0.006226
5870326	LOC341457	12.44742	XM	29208	PREDICTED:	1.572993	-1.192536	1.7339	0.036163	1.843507	0.90767	1.102294	-6.119151	1.171974	-5.777041	1.063214	-5.521255	45632.23	0.000572	0.007692
110725	FLJ90166	13.42757	NM	15336	Homo sapiens	2.872414	1.978456	2.319715	-0.258581	1.842516	-2.670083	-1.238262	-5.776769	-1.558963	-4.062746	-1.258993	-4.904772	233.9465	0.000409	0.005981
1430392	SMG5	11.03657	NM	01532	Homo sapiens	1.151536	-5.890092	1.346515	-4.089164	1.842371	0.84851	1.16932	-5.552513	1.599925	-1.03493	1.368252	-2.873548	2279.691	0.000961	0.011352
2140605	OMA1	18.72739	NM	14524	Homo sapiens	1.465253	-1.725494	1.992604	2.982333	1.841603	1.762788	1.359904	-2.91472	1.25685	-4.513346	-1.081994	-5.382038	852.085	8.72E-05	0.001866
5720398	PDZD8	13.35726	NM	17379	Homo sapiens	1.354492	-3.789173	1.879628	0.902541	1.841119	0.554126	1.387699	-3.269544	1.359269	-3.821054	-1.020916	-5.649626	681.1161	0.000419	0.006095
3990706	CSNK1G3	15.73016	NM	00103	Homo sapiens	1.823475	0.59497	2.074789	2.092615	1.840894	0.404204	1.137821	-5.902351	1.009552	-6.781511	-1.127055	-5.190071	1000.034	0.000199	0.003431
2710497	RIOK2	11.75929	NM	01834	Homo sapiens	1.506508	-2.441551	1.887723	0.525203	1.839744	0.113968	1.253045	-4.905061	1.221198	-5.474689	-1.026079	-5.643455	2062.91	0.000733	0.009259
450138	CREB1	19.10883	NM	00437	Homo sapiens	1.8729	1.8638	2.033268	2.804581	1.839085	1.303716	1.085625	-6.203085	-1.018387	-6.769199	-1.105586	-5.25249	748.3411	7.91E-05	0.001735
3420504	GLT28D1	10.28507	NM	01846	Homo sapiens	1.261037	-4.948778	1.625123	-1.541884	1.838604	0.214961	1.28872	-4.498908	1.45801	-2.939358	1.131363	-5.179396	654.5381	0.001293	0.01428
6900735	AD031	11.31789	NM	03202	Homo sapiens	2.013756	-0.105698	2.171546	0.468204	1.836008	-1.557416	1.078356	-6.379492	-1.096812	-6.581741	-1.182754	-5.090123	504.2868	0.000864	0.010478
6960315	TTRAP	11.03233	NM	01661	Homo sapiens	1.549941	-2.640335	2.041408	0.666791	1.835492	-0.747363	1.317088	-4.601199	1.184234	-5.983991	-1.112186	-5.371772	424.0735	0.000963	0.011368
6040465	GALNT1	18.3629	NM	02047	Homo sapiens	1.426497	-2.171366	1.957519	2.774895	1.834996	1.754435	1.372256	-2.727122	1.286366	-4.098424	-1.06677	-5.471018	2063.816	9.58E-05	0.002005
2970523	PRKCI	11.37533	NM	00274	Homo sapiens	1.518086	-2.825266	2.038434	0.797708	1.833054	-0.62984	1.342766	-4.30613	1.207477	-5.772941	-1.112043	-5.36276	2254.268	0.000845	0.010299
4890725	0	11.07554	XM	03670	PREDICTED:	1.268771	-4.689986	1.601421	-1.367178	1.832066	0.639001	1.262183	-4.603868	1.443969	-2.774979	1.144026	-5.031313	4678.585	0.000947	0.011233
5050131	USP6	9.038841	NM	00450	Homo sapiens	1.628109	-3.463463	2.412873	0.261232	1.831859	-2.629713	1.482009	-4.250051	1.125145	-6.541265	-1.317172	-4.579788	275.8638	0.002188	0.021613
4210131	NMD3	15.91506	NM	01593	Homo sapiens	1.533769	-0.97957	1.844922	1.765709	1.831821	1.607289	1.202869	-4.978583	1.194327	-5.337997	-1.007152	-5.662692	1619.79	0.000188	0.003298
6130239	SGK	12.25548	NM	00562	Homo sapiens	1.067443	-6.514761	1.443393	-3.048176	1.831776	0.628707	1.352197	-3.586306	1.716041	-0.069942	1.269076	-3.905033	725.7481	0.000612	0.008108
5050400	HIRIP5	14.99848	NM	00100	Homo sapiens	1.465993	-2.004531	1.857468	1.537509	1.831444	1.274793	1.267038	-4.299338	1.249286	-4.766908	-1.01421	-5.656483	2759.643	0.000248	0.004094
2070441	PAK1IP1	17.31827	NM	01790	Homo sapiens	1.157634	-5.481402	1.534357	-0.544152	1.831204	2.507183	1.325424	-2.916807	1.58185	0.245628	1.193467	-4.170598	4726.449	0.000127	0.002469
1010438	PPM1B	13.2339	NM	00103	Homo sapiens	1.580295	-2.184302	2.180763	1.755503	1.828062	-0.551945	1.379972	-3.897833	1.156785	-6.144522	-1.192937	-4.850746	410.3687	0.000436	0.006252
4070592	LOC205251	10.32755	NM	17492	Homo sapiens	1.180555	-5.752037	1.584948	-1.981815	1.827476	0.055534	1.342545	-3.96456	1.547981	-2.086187	1.15302	-5.038747	567.6301	0.001271	0.014095
1230433	LOC285407	13.18344	XM	93996	PREDICTED:	1.206138	-5.478992	1.830102	0.280879	1.827419	0.206935	1.517324	-2.063928	1.5151	-2.297642	-1.001468	-5.664961	237.7057	0.000444	0.006339
6550195	LOC645102	10.12861	XM	92813	PREDICTED:	2.731967	0.075303	2.676369	-0.383061	1.826474	-3.754765	-1.020774	-6.50931	-1.49576	-5.057677	-1.46532	-4.277033	44.39112	0.001378	0.015003
2370669	CDR2L	14.01045	NM	01460	Homo sapiens	1.388565	-2.342643	1.527528	-0.867908	1.826346	2.172738	1.100077	-6.006407	1.315276	-3.450686	1.195622	-4.22124	1008.863	0.000338	0.005202
2350474	AP1GBP1	15.53085	NM	00724	Homo sapiens	2.161887	2.675148	1.664818	-1.150037	1.826166	0.150912	-1.298573	-4.381725	-1.183839	-5.793245	1.096917	-5.384416	144.706	0.000211	0.003597

3840397	CDC2		13.78224	NM	00178	Homo sapiens	1.89941	0.03307	2.230773	1.723181	1.825174	-0.831339	1.174456	-5.794399	-1.040673	-6.737812	-1.222225	-4.693105	1560.372	0.000364	0.005501
3420722	SNX7		10.92947	NM	15223	Homo sapiens	1.412769	-4.761468	2.526432	0.975305	1.82442	-2.477147	1.788284	-2.166861	1.291379	-5.652507	-1.384786	-4.130822	21.32339	0.001002	0.011709
5960332	FLJ12716		18.92236	NM	02194	Homo sapiens	2.274378	2.525552	2.359517	2.735371	1.823278	-0.539222	1.037434	-6.471541	-1.247412	-5.382703	-1.294107	-4.049171	257.4669	8.29E-05	0.001798
1260300	ACPL2		11.42705	NM	15228	Homo sapiens	1.694561	-0.594172	1.77418	-0.223458	1.82317	0.114408	1.046985	-6.436345	1.075896	-6.586841	1.027613	-5.64015	808.2494	0.000829	0.010167
4220370	SALL3		19.0088	NM	17199	Homo sapiens	1.302501	-3.712683	1.912368	2.602439	1.822015	1.81058	1.468228	-1.409108	1.398859	-2.494646	-1.04959	-5.551419	1619.862	8.11E-05	0.001771
3990142	PRKCBP1		12.66519	NM	01240	Homo sapiens	2.213147	0.606962	2.343804	0.947649	1.821773	-1.938239	1.059037	-6.442022	-1.214831	-5.991371	-1.286551	-4.535621	2153.57	0.00053	0.00725
6130521	CAPN1		13.47904	NM	00518	Homo sapiens	1.260463	-4.446558	1.617051	-0.458043	1.821756	1.424735	1.282902	-4.001125	1.445306	-2.16553	1.126592	-5.060779	6935.554	0.000402	0.005911
2120088	WDR58		12.42729	NM	02433	Homo sapiens	1.460921	-2.091273	1.665431	-0.208993	1.821219	1.150419	1.139987	-5.752446	1.246624	-4.814271	1.093542	-5.33522	1802.682	0.000576	0.007736
60411	F11R		15.97599	NM	01694	Homo sapiens	1.457129	-2.307993	1.996812	2.284056	1.820502	0.89708	1.370374	-3.178519	1.249375	-4.871908	-1.096847	-5.331398	3865.744	0.000185	0.00325
6280220	NAP1L3		18.12377	NM	00453	Homo sapiens	1.369789	-2.976374	1.963615	2.688096	1.819946	1.499805	1.433517	-2.037173	1.328632	-3.597613	-1.078942	-5.40577	1814.626	0.000102	0.002097
1410307	SLC27A3		14.49362	NM	02433	Homo sapiens	1.467296	-2.050147	1.852479	1.410969	1.819302	1.088083	1.262512	-4.388924	1.239902	-4.913079	-1.018236	-5.651273	3697.382	0.00029	0.004639
5570273	LOC440508		14.40043	XM	94190	PREDICTED:	1.587022	-2.313668	2.354167	2.363155	1.81792	-0.865284	1.483387	-3.08008	1.145492	-6.255334	-1.294979	-4.13787	21.27194	0.000298	0.004738
2320204	SMU1		10.58341	NM	01822	Homo sapiens	1.486744	-2.083264	1.561533	-1.606489	1.817185	0.71985	1.050304	-6.41104	1.222259	-5.234879	1.163719	-4.83957	875.9241	0.001147	0.013028
3420184	LYAR		18.23144	NM	01781	Homo sapiens	1.183822	-5.221647	1.720224	1.204145	1.816199	2.046904	1.45311	-1.389489	1.534182	-0.583913	1.055792	-5.513748	2935.672	9.91E-05	0.002054
4670192	PTPRG		11.70021	NM	00284	Homo sapiens	1.384527	-3.759327	1.908576	0.552774	1.816078	-0.186683	1.378504	-3.67006	1.311695	-4.602512	-1.050933	-5.587334	2004.954	0.000749	0.00941
270603	AMACR		15.02601	NM	01432	Homo sapiens	1.808015	-0.708338	2.429705	2.549947	1.815932	-1.012834	1.343852	-4.414277	1.004379	-6.78448	-1.337993	-3.850037	61.6743	0.000246	0.004066
6770270	LRP1		15.57515	NM	00233	Homo sapiens	1.608882	-1.908333	2.369131	2.763455	1.815929	-0.609138	1.472533	-3.000868	1.12869	-6.332063	-1.304639	-3.967581	338.2218	0.000208	0.003563
1980082	MOSC1		18.89296	NM	02274	Homo sapiens	1.084165	-6.299172	1.565637	-0.204293	1.812535	2.296266	1.444095	-1.303085	1.671826	1.183222	1.157697	-4.602139	1949.126	8.36E-05	0.001806
4610095	HTRA2		10.94545	NM	01324	Homo sapiens	1.663714	-1.314832	1.906986	0.2557	1.812209	-0.488889	1.146223	-5.929693	1.089255	-6.546629	-1.052299	-5.588419	518.6325	0.000996	0.01166
2120653	FLJ22318		20.37324	NM	02276	Homo sapiens	-1.167565	-5.592035	1.324405	-3.992455	1.811623	1.298507	1.546329	-0.83352	2.115188	3.727472	1.367877	-2.53987	825.129	5.80E-05	0.001366
4390093	LOC440359		12.75001	XM	49614	PREDICTED:	1.308495	-3.712437	1.544426	-1.066801	1.810999	1.553532	1.180307	-5.21775	1.384032	-2.792618	1.172603	-4.588518	4277.868	0.000515	0.007081
6980047	AKAP7		14.37459	NM	01637	Homo sapiens	1.044326	-6.607545	1.575271	-1.445953	1.810721	0.68762	1.508409	-1.70386	1.733866	0.29972	1.149466	-4.956466	973.7162	0.000301	0.004766
3170451	RPL26L1		15.60541	NM	01609	Homo sapiens	1.655795	0.174846	1.847549	1.633473	1.809762	1.266677	1.115808	-5.946929	1.092987	-6.401924	-1.02088	-5.646001	1720.509	0.000206	0.003539
50427	ERICH1		10.98153	NM	20733	Homo sapiens	1.518499	-2.240002	1.812131	0.066149	1.808803	-0.013701	1.193369	-5.45123	1.191178	-5.731606	-1.00184	-5.664922	1612.585	0.000982	0.01154
6940379	PTPN2		12.21833	NM	08042	Homo sapiens	1.363694	-4.757409	2.378071	1.354172	1.808685	-1.826379	1.743844	-1.753665	1.326313	-5.138759	-1.314806	-4.29235	372.0955	0.00062	0.00819
3450048	FTSJ3		9.846344	NM	01764	Homo sapiens	1.118929	-6.225389	1.409768	-3.660989	1.808176	0.06629	1.259926	-4.766608	1.615988	-1.306807	1.282605	-3.903227	801.9937	0.001548	0.016524
6110315	SLC15A3		12.30561	NM	01658	Homo sapiens	-1.280981	-5.367131	1.403851	-4.768724	1.807928	-1.76005	1.798307	-1.324133	2.315921	1.360637	1.287835	-4.448703	350.9123	0.000601	0.007989
3440561	C14orf35		10.61941	NM	00101	Homo sapiens	1.402612	-2.754979	1.413084	-2.96847	1.806008	1.071014	1.007466	-6.511808	1.287604	-4.281908	1.278061	-3.582674	501.1765	0.001131	0.012892
6480458	APRT		16.0066	NM	00103	Homo sapiens	1.189211	-5.195301	1.640739	0.248488	1.805777	1.797254	1.379686	-2.449234	1.518466	-0.89757	1.100588	-5.22209	3695.751	0.000183	0.003227
4640068	CXorf40B		9.890404	NM	00101	Homo sapiens	1.802497	-2.188744	2.42814	0.623715	1.803135	-2.577862	1.347098	-5.026149	1.000354	-6.78503	-1.346621	-4.342046	24.95397	0.00152	0.016317
4260541	MCM5		10.81089	NM	00673	Homo sapiens	1.189226	-5.573192	1.571947	-1.744635	1.802167	0.298196	1.321823	-3.97562	1.515412	-2.07501	1.146456	-5.026074	5998.141	0.001049	0.012145
5690241	SH2BP1		19.26712	NM	01463	Homo sapiens	1.904493	1.214983	2.324933	3.537207	1.801186	0.109871	1.220762	-5.137901	-1.057355	-6.664418	-1.290779	-3.79172	639.9063	7.60E-05	0.001685
3370280	MT1JP		12.33237	NM	17562	Homo sapiens	-1.117867	-6.272479	1.231003	-5.7219	1.800867	-0.385595	1.376098	-3.738251	2.013131	1.374306	1.462926	-2.485395	16.16288	0.000596	0.007935
7150132	SLC25A4		16.06139	NM	00115	Homo sapiens	1.181053	-5.241448	1.595006	-0.056607	1.800196	1.96491	1.350496	-2.713573	1.52423	-0.653461	1.128645	-4.946319	2255.608	0.00018	0.003191
4860296	BTBD10		14.51453	NM	03232	Homo sapiens	1.619436	-0.022476	1.730059	0.789781	1.799841	1.371901	1.068309	-6.293128	1.1114	-6.22941	1.040335	-5.592776	1555.798	0.000288	0.004622
830762	TXN		15.25959	NM	00332	Homo sapiens	1.378858	-2.297368	1.617987	0.426951	1.799479	2.210717	1.173425	-5.11093	1.30505	-3.450877	1.112172	-5.072716	15052.45	0.000229	0.003831
5220070	LMLN		9.030692	NM	03302	Homo sapiens	3.114564	0.72442	1.797804	-4.050178	1.798872	-4.106749	-1.732427	-3.837355	-1.731398	-4.082052	1.000594	-5.665031	35.45425	0.002196	0.02166
6110148	ABCF2		11.42152	NM	00569	Homo sapiens	1.248405	-4.571742	1.392267	-3.038352	1.798194	1.298706	1.115237	-5.93636	1.440394	-2.171793	1.291558	-3.320744	704.953	0.000831	0.010186
2710528	PGM2		16.01437	NM	01829	Homo sapiens	1.317752	-3.709718	1.860147	1.778381	1.796313	1.191485	1.411607	-2.374696	1.363165	-3.206311	-1.035536	-5.610392	1184.748	0.000183	0.003222
7560687	WDR44		11.77408	NM	01904	Homo sapiens	1.465457	-3.058019	1.996106	0.948633	1.795761	-0.53026	1.362104	-3.934365	1.225393	-5.520032	-1.111565	-5.336664	430.2353	0.000729	0.009227
4210326	MRPS30		16.57977	NM	01664	Homo sapiens	1.30454	-3.327878	1.665074	1.020615	1.795493	2.261472	1.276369	-3.598778	1.376342	-2.355015	1.078327	-5.352829	4866.058	0.000155	0.002857
2070307	TSPYL1		13.7791	NM	00330	Homo sapiens	1.521038	-1.170461	1.736121	0.751452	1.794817	1.229523	1.141405	-5.688859	1.179995	-5.524637	1.033809	-5.614913	3559.038	0.000364	0.005503
6330730	ATF7IP2		13.4129	NM	02499	Homo sapiens	-1.097932	-6.355543	1.263632	-5.213594	1.79368	0.075048	1.387382	-3.329938	1.969339	1.64135	1.419464	-2.580421	932.7573	0.000411	0.006004
5860463	FAM103A1		13.12015	NM	03145	Homo sapiens	1.511888	-1.700153	1.819203	0.933971	1.793596	0.668248	1.203266	-5.147672	1.186328	-5.589437	-1.014277	-5.656935	676.5593	0.000453	0.006432
2690593	FLJ20516		12.42251	NM	01785	Homo sapiens	1.388831	-3.046157	1.73118	0.270039	1.792865	0.760671	1.246501	-4.620704	1.290916	-4.33027	1.035632	-5.614735	1485.267	0.000577	0.007745
5390204	PPP1R1B		15.38631	NM	18150	Homo sapiens	1.427932	-4.506444	2.902753	2.688463	1.792647	-2.391404	2.032836	-0.505798	1.255415	-5.797428	-1.619255	-2.594135	24.27645	0.00022	0.003722
1740673	CRSP3		17.518	NM	01597	Homo sapiens	2.382052	2.169839	2.481549	2.382996	1.79125	-1.500463	1.041769	-6.470313	-1.329827	-4.923718	-1.385372	-3.621147	361.241	0.00012	0.002364
5900204		0	8.820397	BQ439091		AGENCOURT	2.068034	-2.305213	2.874079	0.154098	1.790885	-3.954158	1.389764	-5.333197	-1.154756	-6.538637	-1.604838	-3.692444	336.0935	0.002412	0.023258
6380717	UCHL3		17.09429	NM	00600	Homo sapiens	1.41242	-2.122825	1.848182	2.255324	1.789902	1.698304	1.308522	-3.392829	1.267259	-4.207211	-1.03256	-5.613077	3414.864	0.000135	0.002581
7560768	USP12		10.85401	NM	18248	Homo sapiens	1.447645	-2.756473	1.745566	-0.175965	1.788046	0.127702	1.205797	-5.254544	1.235141	-5.205261	1.024336	-5.644252	167.2038	0.001032	0.011987
2630400	SLC39A1		14.11552	NM	01443	Homo sapiens	1.398708	-2.593747	1.752047	0.967766	1.787591	1.237326	1.252618	-4.346284	1.27803	-4.258337	1.020287	-5.646403	7103.822	0.000326	0.005075
4760048	RPL34		11.77946	NM	00099	Homo sapiens	1.193441	-5.40889	1.575166	-1.304823	1.786481	0.652645	1.319853	-3.761651	1.496917	-1.906529	1.134155	-5.057597	1411.892	0.000727	0.009218
830754	SIVA		9.919362	NM	00642	Homo sapiens	1.347196	-3.907524	1.654154	-1.057139	1.783746	0.022305	1.227849	-5.049142	1.324043	-4.252995	1.078344	-5.467347	1946.854	0.001502	0.016147
7650441	FHL2		10.18722	NM	00145	Homo sapiens	1.223353	-5.200481	1.556699	-1.839877	1.782211	0.205976	1.272486	-4.498235	1.456825	-2.649798	1.144865	-5.028206	2344.976	0.001345	0.014722
240504	RDH14		14.25083	NM	02090	Homo sapiens	1.474733	-1.403414	1.697872	0.776073	1.781416	1.517399	1.151308	-5.506826	1.207958	-5.059975	1.049205	-5.551536	949.6194	0.000313	0.0049
6100184	TRIP10		9.235	NM	00424	Homo sapiens	1.330535	-3.86211	1.401222	-3.373365	1.780666	0.452324	1.053127	-6.398535	1.338309	-3.855153	1.270795	-3.803838	935.4548	0.002008	0.020239
7100291	FLJ10099		10.57078	NM	01799	Homo sapiens	1.565548	-1.583596	1.721105	-0.448984	1.78046	0.001302	1.099363	-6.171066	1.137276	-6.160151	1.034487	-5.624401	578.533	0.001153	0.013084
2360148	ABCE1		10.35025	NM	00294	Homo sapiens	1.491572	-2.313497	1.709837	-0.521115	1.780273	0.027818	1.146332	-5.816839	1.193555	-5.644336	1.041195	-5.60715	3952.62	0.001259	0.014002
840563	MYOHD1		10.13694	NM	00103	Homo sapiens	1.14392	-5.971803	1.431831	-3.143326	1.77958	0.252552	1.251689	-4.704476	1.555686	-1.54675	1.24287	-4.14293	1038.044	0.001373	0.014959
940725	SYAP1		17.44487	NM	03279	Homo sapiens	1.237704	-4.348044	1.699824	1.335808	1.779245	2.061887	1.373368	-2.215712	1.437537	-1.535724	1.046723	-5.549462	2649.632	0.000122	0.002402
2060500	UBE2E3		13.47717	NM	00635	Homo sapiens	2.144111	1.516148	2.000853	0.419131	1.778458	-1.16432	-1.071598	-6.374343	-1.205601	-5.81831	-1.125049	-5.307051	647.6574	0.000402	0.005911
4540068	PGM3		16.80864	NM	01559	Homo sapiens	1.724045	0.761969	1.946233	2.341731	1.778278	0.931763	1.128876	-5.835463	1.031457	-6.737905	-1.094448	-5.318359	872.2206	0.000146	0.002729
4280703	CHD4		10.4948	NM	00127	Homo sapiens	1.521633	-1.788485	1.644101	-0.869718	1.777813	0.284203	1.080484	-6.266341	1.168359	-5.829627	1.081329	-5.437305	2235.092	0.001189	0.013382
6580131	TSC22D2		16.89974	NM	01477	Homo sapiens	2.086682	2.653152	1.889414	1.082981	1.776806	0.134482	-1.104407	-6.123859	-1.1744	-5.800446	-1.063376	-5.527678	775.5621	0.000142	0.002681
6100450	SCAP2		12.83592	NM	00393	Homo sapiens	2.36994	0.991003	2.37442	0.746636	1.776639	-2.486414	1.00189	-6.514379	-1.333946	-5.26533	-1.336467	-4.310032	214.9201	0.0005	0.006935
7560446	ZNF217		13.41838	NM	00652	Homo sapiens	2.144292	1.141967	2.15012	0.914334	1.776586	-1.501011	1.002718	-6.514251	-1.206973	-5.881833	-1.210253	-4.842855	2600.397	0.00041	0.005997
2690753	PSMA2		16.30414	NM	00278	Homo sapiens	1.382338	-2.399801	1.768587	1.742816	1.776151	1.764567	1.279418	-3.70645	1.284889	-3.865476	1.004276	-5.66407	3206.047	0.000168	0.00303
6110379	C10orf7		12.56374	NM	00602	Homo sapiens	1.331971	-3.561062	1.669392	0.001363	1.776033	0.94708	1.253325	-4.411455	1.333387	-3.627646	1.06388	-5.497571	5636.679	0.000549	0.007448
3710475	GTF2H3		11.80968	NM	00151	Homo sapiens	1.483801	-1.797312	1.655086	-0.274076	1.775689	0.799842	1.115437	-5.97015	1.196717	-5.398965	1.072868	-5.456641	615.4926	0.000719	0.009143
5870180	EPPB9		17.60919	NM	01568	Homo sapiens	1.179154	-5.41951	1.833302	1.702907	1.775598	1.151656	1.55476	-0.606288	1.505823	-1.345351	-1.032498	-5.617708	1390.055	0.000117	0.002326
5390102	RPRM		10.91215	NM	01984	Homo sapiens	1.417271	-2.442524	1.494269	-1.840439	1.775302	1.004758	1.054328	-6.376223	1.25262	-4.642533	1.188074	-4.480297	3930.651	0.001009	0.011777
4260053	PHF10		12.19757	NM	13332	Homo sapiens	1.258666	-4.46962	1.579627	-0.857835	1.775229	1.020052	1.255001	-4.358502	1.410405	-2.597003	1.123828	-5.084018	5020.194	0.000625	0.00824
2600220	FLJ46072		11.92456	NM	19848	Homo sapiens	1.507444	-1.710115	1.723695	0.155938	1.774468	0.554472	1.143455	-5.751034	1.177137	-5.675479	1.029456	-5.630768	3571.478	0.00069	0.008888
7560021	RABL2B		19.41439	NM	00708	Homo sapiens	2.255612	3.791294	1.824736	0.749807	1.772083	0.259702	-1.236131	-4.852832	-1.272859	-4.68598	-1.029713	-5.632292	289.9128	7.33E-05	0.001642
1230615	SERPINH1		11.0589	NM	00123	Homo sapiens	1.560632	-1.554747	1.759687	-0.024126	1.771302	0.020296	1.127548	-5.960259	1.13499	-6.165539	1.006601	-5.663464	8843.571	0.000953	0.011288
1440435	CARF		16.64623	NM	01763	Homo sapiens	2.054169	2.024416	2.072558	1.908268	1.770841	-0.308867	1.008952	-6.511494	-1.159996	-6.007433	-1.17038	-4.895806	782.3483	0.000153	0.00282
4150722	PKD2		17.71158	NM	00029	Homo sapiens	2.094965	1.367139	2.463932	2.99786	1.76908	-1.127258	1.176121	-5.760688	-1.184212	-5.962377	-1.392776	-3.315492	1760.089	0.000114	0.002277

4640504	TIMM44		11.40098	NM	00635	Homo sapiens	1.175033	-5.547187	1.480809	-2.145121	1.768945	0.759072	1.260227	-4.375253	1.505443	-1.611148	1.194581	-4.458662	1220.42	0.000837	0.010241
2900240	PDHX		21.56421	NM	00347	Homo sapiens	1.519374	-0.08849	1.927628	3.79194	1.76887	2.355209	1.268698	-3.549757	1.164209	-5.382137	-1.089751	-5.234753	2626.399	4.39E-05	0.001111
5870551	RFFL		14.01958	NM	00101	Homo sapiens	1.401867	-2.724619	1.822121	1.319688	1.767112	0.793288	1.299781	-3.857916	1.260542	-4.597785	-1.031129	-5.624157	545.7924	0.000337	0.005191
2230008	SLC37A3		17.50068	NM	20711	Homo sapiens	2.087667	1.773481	2.287146	2.6463	1.766846	-0.74856	1.095551	-6.239953	-1.181579	-5.903283	-1.29448	-3.967967	473.114	0.00012	0.002371
3120053	GOLPH3L		16.03484	NM	01817	Homo sapiens	1.159597	-5.455768	1.561461	-0.214924	1.766716	1.919247	1.346556	-2.611912	1.523561	-0.445767	1.13145	-4.879414	2366.659	0.000182	0.003211
70471	RBM13		14.84158	NM	03250	Homo sapiens	1.217084	-4.884324	1.679726	0.496001	1.76608	1.273307	1.380124	-2.547587	1.451075	-1.850155	1.051409	-5.54424	2415.197	0.00026	0.004255
4860735	FLJ11016		21.24862	NM	01830	Homo sapiens	1.710031	1.618041	1.971178	3.646615	1.763877	1.848821	1.152714	-5.387169	1.031488	-6.725869	-1.117526	-5.025312	316.7738	4.72E-05	0.001172
4480523	SRXN1		13.45932	NM	08072	Homo sapiens	1.296731	-3.930114	1.697088	0.437404	1.76339	1.00787	1.308744	-3.620478	1.359874	-3.183831	1.039068	-5.597706	1160.047	0.000405	0.005939
2360358	VDP		12.38854	NM	00371	Homo sapiens	1.470027	-1.848055	1.691066	0.215765	1.763133	0.834903	1.150364	-5.616688	1.199388	-5.326031	1.042617	-5.588228	7074.418	0.000584	0.007821
1780056	CDC37L1		10.4046	NM	01791	Homo sapiens	1.358439	-3.59437	1.655182	-0.737185	1.76155	0.173961	1.218444	-5.04547	1.296745	-4.405157	1.064264	-5.518387	2718.677	0.001232	0.013737
3190477	LOC651423		12.03229	XM	94057	PREDICTED:	-1.248859	-5.391833	1.116117	-6.731866	1.76021	-1.379934	1.393872	-3.94098	2.198254	1.663029	1.577084	-2.036285	1154.374	0.000663	0.008622
5870014	MGC5352		12.73985	XM	94204	PREDICTED:	1.379947	-2.659285	1.618118	-0.101048	1.757889	1.244344	1.172595	-5.268874	1.273881	-4.188303	1.086378	-5.338556	1514.145	0.000516	0.0071
3520192	DFFA		11.48827	NM	00440	Homo sapiens	1.245324	-4.611282	1.516427	-1.506883	1.756607	0.927973	1.217697	-4.801374	1.410562	-2.543893	1.158385	-4.758846	2142.463	0.00081	0.010002
60594		0	13.20793	AK123319		Homo sapiens	-1.413434	-4.240568	1.102388	-6.841887	1.756356	-1.964062	1.558154	-2.919505	2.482493	2.281789	1.593227	-2.299622	85.29385	0.00044	0.006296
4200079		0	15.75212	BU928949		AGENCOURT	2.837622	3.005777	2.06259	-0.487763	1.752277	-2.431065	-1.375756	-4.612517	-1.619391	-3.038984	-1.177091	-5.165102	59.04511	0.000197	0.003414
4880243	C8orf61		14.42317	NM	00103	Homo sapiens	1.590715	-0.972551	1.96693	1.941106	1.751148	0.183411	1.236507	-4.812903	1.100856	-6.39199	-1.123223	-5.161062	193.6997	0.000296	0.004713
510634	HSPC111		18.26442	NM	01639	Homo sapiens	-1.068557	-6.452519	1.440232	-2.201695	1.749868	1.174455	1.53897	-0.570103	1.869834	2.450116	1.214991	-4.082125	2466.521	9.83E-05	0.002039
1410079	MAPK9		15.30847	NM	00275	Homo sapiens	1.519628	-0.89566	1.809733	1.777459	1.749332	1.181211	1.190906	-5.03843	1.151158	-5.777278	-1.034528	-5.608523	1213.277	0.000225	0.003794
2230639	FAM18B		18.71677	NM	01607	Homo sapiens	1.908798	1.645628	2.171882	3.147926	1.748905	0.057352	1.137827	-5.844398	-1.091425	-6.465325	-1.241853	-4.132055	1541.853	8.74E-05	0.001869
1780564	EIF1AX		15.05084	XM	94259	PREDICTED:	1.495171	-1.112551	1.776179	1.572358	1.748799	1.269134	1.187944	-5.049505	1.169632	-5.536092	-1.015656	-5.652904	10193.62	0.000244	0.004039
7570711	MRPS12		13.03412	NM	03336	Homo sapiens	-1.011604	-6.683281	1.391543	-3.379218	1.74703	0.330923	1.40769	-2.698418	1.767302	0.773339	1.255462	-3.901253	1897.04	0.000467	0.006578
1710491	HMG20A		13.31981	NM	01820	Homo sapiens	1.571791	-0.862208	1.806988	1.079327	1.746883	0.502064	1.149636	-5.658332	1.111396	-6.274518	-1.034407	-5.616507	1364.149	0.000424	0.00615
4070097	IDE		17.01093	NM	00496	Homo sapiens	1.383634	-2.75896	1.949422	2.648958	1.74606	0.904312	1.408915	-2.299363	1.261938	-4.452388	-1.116469	-5.12781	884.1956	0.000138	0.002623
6420477	PSMA6		12.1664	NM	00279	Homo sapiens	1.565068	-1.306598	1.822959	0.751557	1.745205	0.042656	1.164779	-5.594892	1.115098	-6.294052	-1.044553	-5.592491	5675.375	0.000632	0.008317
6510653	CD320		10.16307	NM	01657	Homo sapiens	-1.417952	-4.684729	1.138691	-6.756013	1.743393	-2.899966	1.614608	-3.188616	2.472047	1.077282	1.531051	-3.231239	276.9652	0.001359	0.014847
1050075	LOC653643		9.469428	XM	37087	PREDICTED:	2.220775	0.096805	2.05227	-1.027414	1.741721	-2.891226	-1.082107	-6.394778	-1.275047	-5.724202	-1.1783	-5.214704	40.3067	0.001815	0.018687
110324	GMPPA		11.74759	NM	01333	Homo sapiens	1.100759	-6.221943	1.320978	-3.910418	1.741595	0.908704	1.200061	-4.982754	1.582177	-0.427096	1.318413	-2.967634	2297.712	0.000736	0.009283
1690673	C1orf108		11.78055	NM	02459	Homo sapiens	1.185322	-5.325403	1.482179	-1.812635	1.741337	0.91528	1.250444	-4.335006	1.469083	-1.724055	1.174849	-4.56743	5722.246	0.000727	0.009217
5340608		0	8.458051	CB955871		AGENCOURT	1.800272	-3.248267	2.726702	0.127203	1.740701	-3.964205	1.514605	-4.57567	-1.034223	-6.769921	-1.566439	-3.70714	76.13134	0.002844	0.026417
4060537	G3BP2		17.1068	NM	01229	Homo sapiens	1.874454	1.670664	1.992562	2.302158	1.740461	0.251523	1.06301	-6.347921	-1.076987	-6.538951	-1.144848	-4.969583	2892.046	0.000134	0.002576
650424	NKAP		10.46552	NM	02452	Homo sapiens	1.627355	-1.282068	1.816416	0.005007	1.737796	-0.685028	1.116177	-6.090536	1.067866	-6.628906	-1.045241	-5.601292	378.7477	0.001202	0.013502
3290343	LAPTM4B		15.53776	NM	01840	Homo sapiens	1.679603	0.58818	1.837275	1.753089	1.737068	0.814822	1.093875	-6.111446	1.034214	-6.726216	-1.057687	-5.520371	31061.11	0.00021	0.003593
5270465	LOC646849		13.55128	XM	93397	PREDICTED:	1.575645	-1.20533	1.937821	1.626966	1.736706	-0.039079	1.229859	-4.919336	1.102219	-6.390731	-1.115803	-5.223981	17810.73	0.000392	0.005806
7550603	RYK		13.16618	XM	94026	PREDICTED:	1.754834	0.705085	1.739381	0.296072	1.735689	0.209924	-1.008884	-6.510915	-1.01103	-6.779543	-1.002127	-5.664851	1421.936	0.000446	0.006361
2710064	UBA2		12.63245	NM	00549	Homo sapiens	1.802092	-1.080062	2.339046	1.709141	1.735376	-1.932076	1.297961	-4.947275	-1.038445	-6.747423	-1.347861	-3.907924	801.4698	0.000536	0.00732
1510097	TMEM17		11.88266	NM	19827	Homo sapiens	2.745247	1.445037	2.217657	-0.829306	1.734924	-3.388704	-1.237904	-5.800017	-1.582345	-3.985463	-1.278245	-4.833988	84.72667	0.0007	0.008966
1260328	NFATC1		9.342632	NM	17239	Homo sapiens	1.490018	-3.789608	2.177464	0.339779	1.734866	-2.374912	1.461368	-3.859735	1.164326	-6.267111	-1.255119	-4.690375	33.56769	0.001917	0.019518
5860341	ENTPD7		11.49253	NM	02035	Homo sapiens	1.090105	-6.327663	1.37498	-3.333326	1.734733	0.615012	1.261328	-4.293108	1.591345	-0.530612	1.261642	-3.695983	328.4087	0.000809	0.009999
2260767	CHMP2B		11.62464	NM	01404	Homo sapiens	1.429276	-2.379797	1.679727	0.04668	1.731363	0.483568	1.175229	-5.362956	1.211357	-5.20131	1.030741	-5.62502	3714.675	0.00077	0.009607
1340139	CEBPZ		15.23125	NM	00576	Homo sapiens	1.355629	-2.899687	1.784484	1.677963	1.72882	1.108399	1.316351	-3.295157	1.27529	-4.103557	-1.032197	-5.614422	11762.19	0.000231	0.00386
6650674	ATP6V0E		11.31137	NM	00394	Homo sapiens	1.429134	-2.369501	1.655796	-0.172823	1.725421	0.445478	1.158602	-5.539127	1.20732	-5.244458	1.042049	-5.590943	2312.982	0.000866	0.010498
520450	GNG4		15.14746	NM	00448	Homo sapiens	1.019466	-6.666476	1.312013	-3.631847	1.725207	1.475153	1.28696	-3.500059	1.692265	1.392398	1.314932	-2.672931	2449.694	0.000237	0.003948
60605	TTLL12		11.24415	NM	01514	Homo sapiens	1.112795	-6.149083	1.435033	-2.613435	1.724467	0.464673	1.289576	-3.958337	1.549672	-1.027261	1.201691	-4.358364	4699.09	0.000888	0.01071
7050475	TIPRL		15.14739	NM	00103	Homo sapiens	1.570418	-0.309573	1.806999	1.761646	1.722941	0.938287	1.150649	-5.520825	1.097123	-6.327203	-1.048788	-5.554161	1029.079	0.000237	0.003948
5390326	RBM7		11.68761	NM	01609	Homo sapiens	1.249578	-4.906039	1.773277	0.206568	1.722376	-0.287902	1.419101	-2.829864	1.378367	-3.518033	-1.029553	-5.633497	2749.423	0.000752	0.009442
5310754	KIAA0859		11.0818	NM	01593	Homo sapiens	1.181511	-5.436721	1.511922	-1.660764	1.72038	0.468493	1.279651	-4.065129	1.456084	-2.070834	1.137876	-4.969784	1151.315	0.000945	0.011209
3370041	RNF135		12.55383	NM	03232	Homo sapiens	1.616869	-3.191661	2.694746	1.935715	1.718465	-2.911229	1.666644	-2.713141	1.062835	-6.709591	-1.568112	-2.917017	19.56031	0.000551	0.007459
6840414	NAPA		9.499405	NM	00382	Homo sapiens	-1.271838	-5.50687	-1.052358	-7.018947	1.718171	-2.597092	1.20856	-5.75968	2.185236	0.45723	1.808132	-1.343471	123.7075	0.001792	0.018495
5360703	CNIH		13.31854	NM	00100	Homo sapiens	1.549378	-0.843551	1.767672	1.051079	1.716845	0.530074	1.140891	-5.692119	1.108086	-6.268487	-1.029605	-5.626301	4526.126	0.000424	0.006151
540437	HNRPAB		10.44098	NM	03126	Homo sapiens	1.572669	-1.554098	1.780884	0.009163	1.716657	-0.590032	1.132395	-5.940768	1.091556	-6.490776	-1.037414	-5.617927	4532.895	0.001214	0.013598
5700192		0	11.76242	CA952337		iq18d03.y1 HF	-1.038853	-6.634982	-1.071052	-6.901712	1.715668	-0.762453	-1.030995	-6.479748	1.782326	0.07342	1.83757	0.31908	10.54057	0.000732	0.009252
70653	NUTF2		10.34123	NM	00579	Homo sapiens	1.389935	-2.52493	1.425755	-2.390525	1.714688	0.83751	1.025771	-6.479246	1.233646	-4.735927	1.202653	-4.222388	2261.218	0.001264	0.01403
1240487	PLA2G3		17.88636	NM	01571	Homo sapiens	1.040837	-6.594416	1.607491	0.14238	1.714527	1.219369	1.544421	-0.187792	1.647257	0.788018	1.066586	-5.447455	1010.316	0.000109	0.002193
360678	TTC1		12.36727	NM	00331	Homo sapiens	1.013414	-6.680647	1.372052	-3.422086	1.713114	0.332283	1.353891	-3.157426	1.690439	0.387086	1.248578	-3.867136	1344.202	0.000588	0.007863
240594	CRKRS		10.65221	NM	01650	Homo sapiens	1.070623	-6.461815	1.264239	-4.789426	1.710212	0.361212	1.180845	-5.281184	1.597399	-0.48789	1.35276	-2.711448	1105.366	0.001117	0.012742
6130088	STXBP3		17.7655	NM	00726	Homo sapiens	2.084762	2.291387	2.134848	2.373149	1.710088	-0.750695	1.024025	-6.493117	-1.219096	-5.439429	-1.248385	-4.216151	872.4103	0.000112	0.002254
4210465	CNOT7		17.98356	NM	01335	Homo sapiens	1.90428	2.437959	1.88337	2.05492	1.710004	0.487599	-1.011102	-6.508247	-1.113612	-6.216658	-1.101384	-5.254105	3687.092	0.000106	0.002155
2000528	SDCBP		20.71212	NM	00100	Homo sapiens	2.134287	3.037604	2.179954	3.113992	1.709858	-0.358023	1.021397	-6.495753	-1.248225	-4.995634	-1.274933	-3.83041	3024.322	5.35E-05	0.001288
1010544	HLA-E		11.35947	NM	00551	Homo sapiens	1.292386	-3.992962	1.609249	-0.465944	1.708433	0.48345	1.245176	-4.451488	1.321921	-3.689077	1.061634	-5.503006	1112.813	0.00085	0.010345
1980253	KLHL23		11.534	NM	14471	Homo sapiens	1.53605	-1.460945	1.766641	0.462564	1.707068	-0.123742	1.15012	-5.70113	1.111336	-6.304019	-1.034898	-5.618075	1846.762	0.000796	0.009886
50097	FLJ20582		12.82111	NM	01410	Homo sapiens	1.036585	-6.627016	1.493252	-1.976182	1.705997	0.212124	1.44055	-2.134533	1.645785	-0.095454	1.14247	-4.947572	3585.983	0.000502	0.00696
3370465		0	13.55455	R38630		yc88d04.s1 So	-1.801164	-2.531446	-1.084838	-6.964212	1.705521	-3.498408	1.660306	-3.215962	3.071922	2.47038	1.850214	-1.864838	60.97967	0.000392	0.005802
6290475	METTL5		12.88368	NM	01416	Homo sapiens	1.644724	-0.559211	1.892128	1.254933	1.704231	-0.360097	1.150423	-5.735927	1.036181	-6.731368	-1.110253	-5.264402	6740.41	0.000491	0.006855
5340450	ATP6V1C1		11.79023	NM	00169	Homo sapiens	1.097193	-6.265627	1.468533	-2.100883	1.704049	0.40637	1.338445	-3.260232	1.553099	-0.873418	1.160375	-4.7449	950.668	0.000724	0.009191
6450538	BECN1		10.74802	NM	00376	Homo sapiens	1.436177	-2.168735	1.5931	-0.674159	1.703783	0.400403	1.109265	-5.993134	1.186332	-5.448228	1.069476	-5.463257	1219.611	0.001075	0.012377
1710095	CHRNA5		10.20436	NM	00074	Homo sapiens	1.577747	-2.285735	1.998068	0.559858	1.702327	-1.622325	1.266406	-5.003413	1.078961	-6.608432	-1.173728	-5.000122	618.4125	0.001336	0.014628
1510431	C11orf48		11.13147	NM	02409	Homo sapiens	1.110797	-6.13528	1.39697	-2.907889	1.702251	0.517885	1.257628	-4.251158	1.532459	-0.995116	1.218531	-4.102703	5198.206	0.000927	0.011046
6840192	EIF3S9		9.144324	NM	00375	Homo sapiens	1.234081	-4.848723	1.417186	-2.895351	1.702132	0.168244	1.148374	-5.656134	1.379272	-3.104294	1.201065	-4.387274	8540.034	0.002089	0.020899
5360204	KIAA1970		11.88271	NM	13345	Homo sapiens	1.515126	-1.390227	1.723593	0.446958	1.701188	0.183391	1.137591	-5.758572	1.122803	-6.16176	-1.01317	-5.657567	2354.554	0.0007	0.008966
6250673	EIF5A		11.3394	NM	00197	Homo sapiens	1.296037	-3.860812	1.585341	-0.572982	1.700977	0.576555	1.223222	-4.671231	1.312445	-3.724746	1.072941	-5.433549	9849.959	0.000857	0.010416
270722	CAPZB		9.486927	NM	00493	Homo sapiens	1.272049	-4.341754	1.475709	-2.128069	1.699558	0.219206	1.160104	-5.519405	1.336079	-3.605892	1.151689	-4.853439	4898.331	0.001801	0.018574
6250577	FLJ38482		10.5174	NM	15268	Homo sapiens	1.667856	-1.521586	2.005635	0.612633	1.698222	-1.651571	1.202523	-5.548502	1.018207	-6.774933	-1.18102	-4.950564	187.0424	0.001178	0.013293
1850327	STAU		13.97204	NM	01745	Homo sapiens	1.757389	0.530381	1.900925	1.417124	1.697261	-0.337829	1.081676	-6.253979	-1.035427	-6.732456	-1.119996	-5.188466	3505.128	0.000342	0.005242
5890288	PMM2		10.73473	NM	00030	Homo sapiens	1.043489	-6.60171	1.300087	-4.370043	1.697028	0.142503	1.245904	-4.533987	1.626302	-0.275466	1.305319	-3.265732	1069.197	0.001081	0.012424
2680010	EPC1		16.14773	NM	02520	Homo sapiens	1.869903	1.647775	1.934076	1.893622	1.696927	-0.136584	1.034319	-6.463052	-1.101934	-6.368213	-1.139752	-5.010246	423.1729	0.000176	0.003133
3120632	NOMO2		8.872273	NM	17361	Homo sapiens	1.27811	-4.340639	1.452019	-2.538601	1.696422	0.024004	1.136068	-5.791661	1.32729	-3.810974	1.16832	-4.729673	1521.093	0.002357	0.022869
5910202	VPRBP		11.38228	NM	01470	Homo sapiens	1.834626	0.736102	1.671094	-0.918579	1.696291	-0.746873	-1.097859	-6.182556	-1.081551	-6.546947	1.015078	-5.657139	144.6978	0.000843	0.010285

3060408	ZNF485		14.43005	NM	14531	Homo sapiens	1.006972	-6.687131	1.610537	-0.87257	1.694831	-0.098506	1.599386	-0.571257	1.683097	0.067644	1.052339	-5.558313	310.3627	0.000295	0.004706
620292	ZNF675		15.6747	NM	13833	Homo sapiens	2.054645	-0.360097	2.880462	2.79432	1.693773	-2.930239	1.401927	-4.472368	-1.213058	-6.032381	-1.700619	-2.051047	159.0115	0.000202	0.003479
2850543	ATE1		11.73893	NM	00704	Homo sapiens	1.32825	-3.516812	1.668655	0.153593	1.692903	0.34023	1.256281	-4.305642	1.274536	-4.311982	1.014531	-5.655447	212.5678	0.000738	0.009308
2690019	RNU70		9.788554	NR	00001	Homo sapiens	1.219003	-4.956177	1.452999	-2.289383	1.690481	0.276983	1.191957	-5.123173	1.386774	-2.864241	1.163443	-4.714493	1477.364	0.001586	0.016817
6960575	LOC56902		10.55684	NM	02014	Homo sapiens	1.148461	-5.745215	1.415509	-2.623714	1.686934	0.428149	1.232526	-4.55441	1.468865	-1.698057	1.191751	-4.379323	1532.61	0.00116	0.013135
5340722	ZNF480		13.23729	NM	14468	Homo sapiens	1.214114	-4.955677	1.689179	0.496722	1.681982	0.372833	1.391285	-2.465687	1.385358	-2.761552	-1.004278	-5.664167	2305.756	0.000436	0.006246
4290537	PSPC1		14.47107	NM	01828	Homo sapiens	1.514367	-0.654835	1.738917	1.503072	1.678194	0.846452	1.14828	-5.474761	1.108182	-6.183726	-1.036183	-5.59795	1558.834	0.000292	0.004661
1570639	ZNF195		12.55789	NM	00715	Homo sapiens	1.62575	-1.239568	2.022262	1.544508	1.677169	-1.145797	1.243895	-4.969002	1.031628	-6.749014	-1.20576	-4.617701	3398.035	0.00055	0.007456
3290202	ZNF28		11.93674	NM	00696	Homo sapiens	1.277002	-3.901671	1.567821	-0.367272	1.676122	0.76856	1.227735	-4.444637	1.312544	-3.476141	1.069078	-5.43486	411.6116	0.000687	0.008858
3310435	ADAMTS20		12.93349	NM	02500	Homo sapiens	3.117972	1.124968	2.891425	0.26253	1.675159	-4.489524	-1.078351	-6.445439	-1.861299	-3.256397	-1.72606	-3.158653	40.09803	0.000483	0.006759
7560180	LOC283377		12.81946	NM	20734	Homo sapiens	-1.084552	-6.380571	1.247155	-5.070425	1.674313	-0.134559	1.352604	-3.229864	1.81588	1.400303	1.342507	-2.895072	370.2374	0.000502	0.006962
1570132	AUH		18.98524	NM	00169	Homo sapiens	1.560673	-0.471304	2.066512	3.69137	1.673847	0.392331	1.324116	-3.285816	1.072516	-6.523281	-1.234588	-3.870648	1909.387	8.16E-05	0.001778
110192	CTTN		10.50137	NM	13856	Homo sapiens	1.275772	-4.061905	1.515034	-1.250326	1.673135	0.448752	1.187543	-5.077277	1.311468	-3.655067	1.104355	-5.200019	3382.579	0.001185	0.013363
4390682	ATP5H		11.82795	NM	00635	Homo sapiens	1.305284	-3.812296	1.684907	0.327778	1.672429	0.151081	1.290836	-3.849774	1.281276	-4.216608	-1.007461	-5.66248	26874.29	0.000714	0.009099
3180685	KCNA5		17.07735	NM	00223	Homo sapiens	2.909515	3.570666	1.86251	-1.394819	1.670829	-2.774022	-1.562147	-3.041422	-1.74136	-1.956947	-1.114722	-5.420624	298.4409	0.000135	0.002589
6520762		0	9.527327	BF511167		UI-H-BI4-aoi-a	1.339215	-3.368413	1.131625	-6.329181	1.670064	0.122003	-1.183444	-5.210753	1.247047	-4.669236	1.47581	-1.376283	14.5477	0.001771	0.018307
5890022	MPHOSPH10		9.858262	NM	00579	Homo sapiens	1.308316	-3.816744	1.555672	-1.107984	1.668841	0.032771	1.189064	-5.166107	1.275564	-4.331512	1.072746	-5.446159	2788.385	0.00154	0.016467
1780647	TRIM56		13.7031	NM	03096	Homo sapiens	-1.006078	-6.687547	1.513051	-1.678507	1.668188	-0.072093	1.522248	-1.136016	1.678328	0.300831	1.102532	-5.259519	642.4998	0.000373	0.005607
1660376	BAT2D1		11.96399	NM	01517	Homo sapiens	1.730443	0.215951	1.779317	0.354378	1.667434	-0.698813	1.028244	-6.481725	-1.037788	-6.726576	-1.067099	-5.506669	4644.715	0.00068	0.008794
5420451	GFPT1		15.86189	NM	00205	Homo sapiens	1.595522	-0.007498	1.875155	2.371665	1.665223	0.393475	1.175261	-5.222521	1.043685	-6.68408	-1.126068	-5.008732	1967.71	0.000191	0.003337
240075	AEBP2		12.27962	NM	15320	Homo sapiens	1.44572	-2.038983	1.751974	0.922086	1.664835	0.034706	1.211835	-4.878744	1.151561	-5.839395	-1.052341	-5.54718	1242.875	0.000607	0.008048
6250121	H2AFJ		15.85759	NM	17792	Homo sapiens	-1.273155	-4.597158	1.178851	-6.008616	1.663322	-0.72771	1.50086	-1.867155	2.117666	2.975374	1.410969	-2.470862	752.5725	0.000191	0.003339
4220278	INADL		13.93851	NM	17687	Homo sapiens	-1.259528	-4.933718	1.213573	-5.780487	1.66103	-1.1921	1.528529	-1.947027	2.092114	2.288239	1.36871	-3.106837	463.3918	0.000346	0.005285
7560202	C1QBP		13.28545	NM	00121	Homo sapiens	1.404955	-2.36096	1.769779	1.357859	1.660575	0.249745	1.25967	-4.158764	1.181942	-5.423616	-1.065763	-5.471431	20203.81	0.000429	0.006199
6270373	RNUXA		10.73534	NM	03217	Homo sapiens	1.310739	-3.534423	1.551821	-0.742019	1.659757	0.392863	1.183928	-5.099042	1.266276	-4.241233	1.069554	-5.442236	3057.871	0.001081	0.012424
3460215	NFKBIB		14.68942	NM	00250	Homo sapiens	-1.084871	-6.385425	1.483304	-2.206329	1.659203	-0.380364	1.609194	-0.418442	1.800022	1.164522	1.118586	-5.162839	616.6408	0.000272	0.004419
1340026	LOC650803		10.72414	XM	93989	PREDICTED:	-1.103077	-6.31442	1.235184	-5.489259	1.657467	-1.023755	1.362503	-3.551051	1.828314	0.71314	1.341879	-3.257836	1120.659	0.001085	0.012467
1770148	AATF		10.96429	NM	01213	Homo sapiens	1.412522	-1.864284	1.489807	-1.157483	1.656131	0.744558	1.054714	-6.343685	1.172464	-5.393508	1.111642	-5.083642	2173.16	0.000989	0.011594
3710424		0	9.115863	BM129209		if19a01.y1 Me	1.549255	-4.043013	2.504558	0.472863	1.654711	-3.797558	1.616621	-3.469941	1.068069	-6.710542	-1.513593	-3.544478	42.93406	0.002116	0.02105
1990136	IDH3A		11.48828	NM	00553	Homo sapiens	-1.047199	-6.585924	1.224428	-5.320072	1.654265	-0.247847	1.28222	-4.06741	1.732344	0.767294	1.351051	-2.761684	337.2078	0.00081	0.010002
3440519	RNF10		9.308302	NM	01486	Homo sapiens	1.221717	-4.851172	1.435371	-2.359402	1.651551	0.081594	1.174881	-5.272356	1.351828	-3.193602	1.150608	-4.802857	1044.659	0.001945	0.019744
5860154	EP300		27.21538	NM	00142	Homo sapiens	2.126552	4.044771	2.272283	4.757561	1.647968	-0.034748	1.068529	-6.289972	-1.290409	-4.063572	-1.378839	-2.316253	1574.066	1.36E-05	0.000464
7510307	AMMECR1		11.32939	NM	00102	Homo sapiens	1.324645	-3.688024	1.722327	0.44974	1.647871	-0.324983	1.300218	-3.846404	1.24401	-4.800712	-1.045183	-5.580214	2145.426	0.00086	0.010448
1570561	RTTN		13.06787	NM	17363	Homo sapiens	2.246059	0.787162	2.359163	1.042248	1.646816	-3.119307	1.050357	-6.460929	-1.36388	-4.927192	-1.432561	-3.581823	95.31849	0.000461	0.006521
4060044	LEO1		13.55449	NM	13879	Homo sapiens	1.32254	-3.050744	1.662309	1.006752	1.645659	0.775797	1.256907	-3.880324	1.244317	-4.310215	-1.010118	-5.659278	1893.216	0.000392	0.005802
6110373	ACA10		14.93338	NR	00232	Homo sapiens	-1.137756	-5.896134	1.310764	-4.134844	1.645115	-0.192876	1.49133	-1.392494	1.87174	2.10353	1.255081	-3.744277	1151.879	0.000253	0.004162
5700477	DDT		11.81095	NM	00135	Homo sapiens	1.224495	-4.752335	1.615256	-0.086586	1.64479	0.17518	1.31912	-3.305801	1.34324	-3.205841	1.018285	-5.648752	21162.97	0.000719	0.009141
3520402	HERC4		11.08184	NM	01560	Homo sapiens	1.516474	-1.20762	1.664268	0.076447	1.644252	-0.180235	1.097459	-6.090671	1.08426	-6.458639	-1.012174	-5.658341	1462.372	0.000945	0.011209
6860162	VPS33A		11.40024	NM	02291	Homo sapiens	1.24513	-4.316681	1.540391	-0.607548	1.642666	0.508099	1.237132	-4.274338	1.319272	-3.324499	1.066395	-5.447069	639.8495	0.000837	0.010242
5860689	POP7		9.480843	NM	00583	Homo sapiens	1.14632	-5.746282	1.365681	-3.206228	1.642637	0.08822	1.19136	-5.042917	1.432965	-2.059705	1.202797	-4.226865	1171.136	0.001806	0.018618
4560497	HSPA9B		11.41881	NM	00413	Homo sapiens	1.351946	-2.708672	1.558856	-0.295158	1.64216	0.600771	1.153046	-5.385776	1.214663	-4.80378	1.053439	-5.517831	15979.29	0.000831	0.010194
3180068	PIGK		11.73214	NM	00548	Homo sapiens	1.389171	-2.080711	1.555165	-0.20926	1.641583	0.73429	1.119491	-5.759396	1.1817	-5.223786	1.055568	-5.501384	1168.466	0.00074	0.009323
1110279	BTBD1		14.13163	NM	02523	Homo sapiens	1.698363	-0.1013	2.000142	1.99411	1.639968	-0.996012	1.177688	-5.484649	-1.035608	-6.733448	-1.219623	-4.361694	991.9237	0.000325	0.005056
5690382	METTL7A		15.74582	NM	01403	Homo sapiens	-2.887258	-0.3198	-1.10687	-6.975081	1.637316	-5.075802	2.608488	-0.966267	4.727353	2.979036	1.812296	-3.230511	340.7447	0.000198	0.003419
4230504	TMED7		18.0327	NM	18183	Homo sapiens	1.850368	1.732622	2.038759	2.890099	1.635988	-0.511997	1.101812	-6.080907	-1.13104	-6.093516	-1.246194	-3.924095	2419.622	0.000104	0.002134
5810026	ZNF271		10.7685	NM	00662	Homo sapiens	1.251368	-4.143571	1.475683	-1.266653	1.633387	0.584801	1.179255	-5.020783	1.305281	-3.424438	1.106869	-5.117445	1652.038	0.001067	0.012301
4010600	FLJ11712		12.93136	NM	02457	Homo sapiens	1.280824	-4.245791	1.813863	1.265543	1.632621	-0.477384	1.416169	-2.401725	1.274665	-4.412076	-1.111013	-5.201404	4223.519	0.000483	0.006761
360300	HAGHL		11.10122	NM	03230	Homo sapiens	-1.277222	-5.054237	1.173465	-6.318992	1.632482	-2.168024	1.498775	-2.820606	2.085043	1.340202	1.391164	-3.294012	315.6313	0.000938	0.011142
4780709	GPR89A		9.056871	NM	01633	Homo sapiens	1.486804	-3.489101	2.060769	0.292816	1.629956	-2.714953	1.386039	-4.194887	1.096282	-6.563175	-1.264309	-4.499312	218.0361	0.002171	0.021463
4920474	LOC649801		11.39747	XM	93887	PREDICTED:	2.207547	-0.540001	2.638747	0.870912	1.629683	-4.005177	1.19533	-6.002421	-1.354587	-5.393256	-1.619178	-3.020808	321.4464	0.000838	0.010249
2260255	WNK3		11.51931	NM	00100	Homo sapiens	1.985383	0.614969	1.956704	0.156561	1.629314	-2.338114	-1.014657	-6.508152	-1.218539	-5.709617	-1.200937	-4.837441	890.1325	0.000801	0.009922
6200280	ETF1		10.93211	NM	00473	Homo sapiens	1.296379	-3.478924	1.516314	-0.752957	1.629005	0.525607	1.169653	-5.153232	1.25658	-4.158221	1.074319	-5.385242	3346.485	0.001001	0.011703
5910717	PGEA1		8.804638	NM	00100	Homo sapiens	1.944698	-2.26617	2.600664	0.112952	1.628722	-4.349577	1.33731	-5.395366	-1.194003	-6.340101	-1.596751	-3.406201	49.90964	0.002429	0.023363
1990471	OCIAD2		11.92221	NM	15239	Homo sapiens	-1.170069	-5.735683	1.186443	-5.952805	1.627936	-1.150327	1.38822	-3.165144	1.904797	1.464975	1.372115	-2.888436	3589.916	0.00069	0.008891
3800450	CSE1L		10.7404	NM	00131	Homo sapiens	1.431603	-1.731223	1.547624	-0.584876	1.626966	0.267846	1.081042	-6.16723	1.136464	-5.882096	1.051267	-5.534144	14867.51	0.001079	0.012408
2850369	CDV3		10.9373	NM	01754	Homo sapiens	1.659819	0.145037	1.600533	-0.748309	1.624168	-0.556734	-1.037042	-6.450372	-1.02195	-6.761909	1.014767	-5.655608	3837.941	0.000999	0.011685
1850646	RNU66		11.19528	NR	00244	Homo sapiens	1.973284	-1.724014	2.767377	1.20916	1.623464	-4.094697	1.402422	-4.876096	-1.215478	-6.183653	-1.704613	-2.635467	25.08623	0.000905	0.010852
580008	SMURF2		8.797277	NM	02273	Homo sapiens	1.649001	-3.056259	2.310978	0.274516	1.623384	-3.638439	1.401442	-4.607553	-1.01578	-6.780173	-1.423556	-3.839205	46.12566	0.002437	0.023418
780274	WNT8A		18.9288	NM	03193	Homo sapiens	4.12536	3.076265	3.207036	1.032472	1.622306	-4.778944	-1.286347	-5.794134	-2.542899	-0.614724	-1.976838	-2.216612	35.52808	8.28E-05	0.001796
1260600	FAM63B		14.52815	NM	01909	Homo sapiens	1.137312	-5.908389	1.786419	1.153433	1.621754	-0.477938	1.570738	-0.553552	1.425954	-2.414417	-1.101535	-5.260552	668.213	0.000286	0.00461
6840471	CRI2		11.91606	NM	15323	Homo sapiens	1.580882	-0.433893	1.70516	0.546847	1.619716	-0.369764	1.078613	-6.226145	1.024564	-6.754473	-1.052753	-5.543666	1297.649	0.000692	0.008896
1770593	MRFAP1		9.422224	NM	03329	Homo sapiens	1.407845	-2.169266	1.471738	-1.665068	1.619454	0.024325	1.045384	-6.403837	1.150307	-5.753941	1.100368	-5.218113	11078.66	0.001852	0.018987
7160202	CCNT2		12.11419	NM	00124	Homo sapiens	1.950042	0.883792	1.902985	0.292203	1.618385	-2.034828	-1.024728	-6.49453	-1.204931	-5.710287	-1.175854	-4.930306	1313.409	0.000644	0.008426
3800170	WDSOF1		12.14245	NM	01542	Homo sapiens	1.211228	-4.967086	1.67327	0.41253	1.614764	-0.2513	1.381466	-2.542414	1.333163	-3.410189	-1.036231	-5.604048	4543.998	0.000637	0.008363
6450041	PSMC6		12.99065	NM	00280	Homo sapiens	1.451888	-1.47831	1.719651	1.264828	1.613314	0.102443	1.184424	-5.030548	1.111184	-6.15982	-1.065912	-5.454114	6876.267	0.000474	0.00666
150240		0	10.06942	XM	37388	PREDICTED:	2.459224	0.016754	2.401837	-0.490412	1.612512	-4.378049	-1.023893	-6.506117	-1.525089	-4.549809	-1.4895	-3.886474	74.68845	0.001412	0.015323
580347	LOC650832		9.07314	XM	93991	PREDICTED:	-1.298238	-5.130163	1.081259	-6.91967	1.609613	-2.952043	1.403731	-4.092353	2.08966	0.568875	1.488647	-2.919856	1112.529	0.002156	0.021345
2680722	ZNF588		13.43787	NM	01622	Homo sapiens	1.770101	-2.633829	3.008483	2.238929	1.609402	-4.031462	1.699611	-2.9115	-1.09985	-6.627232	-1.869318	-1.712606	44.14555	0.000407	0.00597
3830192	TMF1		17.70845	NM	00711	Homo sapiens	1.971167	1.650913	2.201445	2.844976	1.607891	-1.619744	1.116823	-6.06062	-1.225934	-5.348227	-1.369151	-3.066018	252.51	0.000114	0.002278
10358	FHOD1		14.42497	NM	01324	Homo sapiens	-1.199666	-5.303417	1.279026	-4.718163	1.607805	-0.904976	1.534404	-1.194704	1.928828	2.163581	1.257054	-3.847761	1719.393	0.000296	0.004712
1500390	LOC255620		16.45913	XM	17313	PREDICTED:	2.27866	-1.210774	3.964785	2.957478	1.607285	-4.712601	1.739964	-3.455466	-1.417708	-5.366406	-2.46676	-0.496945	30.20177	0.000161	0.002934
830673	SERTAD3		11.61037	NM	20334	Homo sapiens	1.609103	0.150815	1.592673	-0.316162	1.607051	-0.211624	-1.010316	-6.508626	-1.001277	-6.784942	1.009028	-5.661006	1101.279	0.000774	0.009648
2510451	IER2		12.08981	NM	00490	Homo sapiens	-1.162515	-5.7435	1.053127	-6.957254	1.606651	-1.070647	1.224276	-4.911311	1.867756	1.525369	1.525601	-1.296856	314.0721	0.00065	0.00849
5360538	C6orf166		10.59748	NM	01806	Homo sapiens	1.423439	-2.327906	1.687811	0.295487	1.603193	-0.623576	1.185727	-5.19902	1.126282	-6.102048	-1.052781	-5.545814	4475.412	0.001141	0.012971
650441	PDE9A		9.909828	NM	00100	Homo sapiens	1.398285	-5.122638	2.673431	0.711534	1.602017	-4.322723	1.911936	-2.041375	1.145701	-6.497532	-1.668791	-2.899868	24.18546	0.001508	0.016204
2140156	CD46		14.91087	NM	15382	Homo sapiens	1.72849	0.663082	1.927396	2.044143	1.601948	-0.904746	1.115076	-5.977621	-1.078992	-6.515096	-1.203158	-4.386031	2308.437	0.000254	0.004185
6960717	ZNF537		11.63402	NM	02085	Homo sapiens	-1.529386	-3.502572	1.072933	-6.96343	1.600691	-3.371906	1.640929	-2.531357	2.448075	1.759288	1.491883	-3.137519	1039.88	0.000767	0.009586

110474	DDX59		11.03725	NM	00103	Homo sapiens	1.688606	-0.549968	1.864352	0.592374	1.599506	-1.734698	1.104078	-6.150725	-1.055705	-6.672684	-1.16558	-4.914917	805.9363	0.000961	0.011352
6660437	ACAA2		11.48001	NM	00611	Homo sapiens	1.400378	-2.183327	1.648809	0.509387	1.597611	-0.103739	1.177403	-5.131057	1.140843	-5.840397	-1.032046	-5.613078	4772.823	0.000813	0.010026
940246	FAM101B		8.828233	NM	18270	Homo sapiens	-1.541636	-4.071123	1.035272	-7.056792	1.597057	-4.137694	1.596013	-3.575189	2.462079	0.565635	1.542644	-3.369416	255.7334	0.002403	0.023195
1260053	CAMK2D		11.18743	NM	17211	Homo sapiens	2.30907	0.771568	2.129957	-0.359048	1.595415	-3.688478	-1.084092	-6.380664	-1.447316	-4.430416	-1.335049	-4.306833	149.5177	0.000907	0.010866
1780672	PELI1		13.67306	NM	02065	Homo sapiens	1.718001	-0.110145	2.014154	1.875267	1.59487	-1.607614	1.172383	-5.5765	-1.077204	-6.565264	-1.262895	-4.007973	1472.215	0.000377	0.005646
2470463	GPBP1		10.6032	NM	02291	Homo sapiens	1.669177	-0.355187	1.746627	0.046481	1.593651	-1.443343	1.0464	-6.428597	-1.047392	-6.694752	-1.095991	-5.356297	3867.292	0.001138	0.012956
5490671	HIATL1		10.8729	NM	03255	Homo sapiens	1.639668	-0.646653	1.795561	0.442766	1.593219	-1.464466	1.095076	-6.178683	-1.029154	-6.750258	-1.127002	-5.152746	1057.844	0.001024	0.011918
580039	FLJ16360		19.37919	NM	00102	Homo sapiens	1.525132	-1.644492	2.314934	4.077504	1.59147	-1.326717	1.517858	-1.595509	1.043497	-6.70576	-1.454588	-1.996557	25.92043	7.39E-05	0.00165
2360102	DPM1		10.58034	NM	00385	Homo sapiens	1.359052	-3.171053	1.702062	0.389665	1.591406	-0.790739	1.252389	-4.393961	1.170968	-5.638635	-1.069534	-5.465058	5406.719	0.001149	0.013039
2640114	IARS		12.31655	NM	00216	Homo sapiens	1.519682	-1.002785	1.753606	1.138613	1.59073	-0.558961	1.153929	-5.512206	1.046752	-6.674094	-1.102391	-5.229261	7000.651	0.000599	0.007967
1090181		0	16.02771	AA287314		zs52c11.s1 N	-1.336959	-4.350793	-1.337933	-4.705856	1.590608	-2.282532	-1.000729	-6.514434	2.126577	1.92036	2.128127	1.326391	20.11081	0.000182	0.003215
160465	TMEM55A		10.20962	NM	01871	Homo sapiens	1.629089	-2.466506	2.21041	0.906802	1.589892	-3.174047	1.356838	-4.521693	-1.024654	-6.769902	-1.390289	-3.65873	838.6242	0.001333	0.014611
6370187	ENTH		9.977255	NM	01466	Homo sapiens	1.444546	-2.611189	1.780154	0.378188	1.589271	-1.449069	1.232328	-4.89588	1.100187	-6.40609	-1.120108	-5.195081	4311.345	0.001466	0.015817
5220053	YWHAG		12.68788	NM	01247	Homo sapiens	1.798172	0.337399	1.94305	1.146844	1.588373	-1.858883	1.080569	-6.290126	-1.132084	-6.220324	-1.223296	-4.436287	4415.699	0.000526	0.007205
1430026	FKBP3		12.26972	NM	00201	Homo sapiens	1.574517	-1.414836	1.964449	1.558207	1.587539	-1.662073	1.247651	-4.805627	1.00827	-6.782276	-1.237417	-4.242088	1820.262	0.000609	0.00807
6940167	DHX36		14.34046	NM	02086	Homo sapiens	1.47536	-1.455885	1.867419	2.227066	1.586817	-0.517882	1.265738	-4.066784	1.075545	-6.501268	-1.176833	-4.512643	2401.601	0.000304	0.004801
3170286	JMJD1C		13.82973	NM	00424	Homo sapiens	1.518411	-1.756062	2.01857	2.251006	1.586248	-1.42448	1.329396	-3.752999	1.044676	-6.702494	-1.272544	-3.806605	4168.308	0.000358	0.005436
4490368	HSPA12B		10.84716	NM	05297	Homo sapiens	1.648269	-1.648585	2.066446	1.031886	1.584149	-2.588146	1.253707	-5.102354	-1.040476	-6.735858	-1.304452	-4.000478	361.4936	0.001034	0.012012
7050598	BBS7		13.15714	NM	01819	Homo sapiens	1.777735	0.583838	1.891938	1.222083	1.58334	-1.551241	1.06424	-6.353809	-1.122776	-6.241172	-1.194903	-4.589415	211.6872	0.000448	0.006376
4250767	C20orf129		11.23686	NM	03091	Homo sapiens	1.5217	-1.134738	1.717678	0.612886	1.5827	-0.817627	1.128789	-5.809329	1.040087	-6.706146	-1.085283	-5.366513	3216.449	0.000891	0.010727
1340390	NUDT9		12.5625	NM	19803	Homo sapiens	1.353146	-3.069761	1.780223	1.411504	1.581736	-0.631588	1.315619	-3.425705	1.168932	-5.591838	-1.125487	-5.032686	2018.09	0.000549	0.007449
2470296	GALNT12		18.50117	NM	02464	Homo sapiens	2.37066	3.467509	1.981305	1.066003	1.580319	-2.200558	-1.196514	-5.472782	-1.500115	-2.661543	-1.253737	-4.258144	608.3585	9.24E-05	0.001949
6400255	SNX16		9.808179	NM	02213	Homo sapiens	1.487999	-2.387136	1.827267	0.410847	1.580057	-1.838114	1.228003	-5.052295	1.061867	-6.644235	-1.156457	-4.969537	1476.369	0.001573	0.016699
3360546	ANP32A		12.79254	NM	00630	Homo sapiens	1.205237	-4.902004	1.670324	0.800679	1.579228	-0.251571	1.385888	-2.199557	1.310305	-3.485016	-1.057684	-5.500222	1430.233	0.000507	0.007008
4480139	FSBP		16.29659	NM	00655	Homo sapiens	3.025161	2.352379	2.624362	0.859745	1.578928	-4.302774	-1.152722	-6.181789	-1.915959	-1.998134	-1.662117	-2.782259	63.02236	0.000169	0.003035
5220373		0	12.35994	BC030623		Homo sapiens	-1.063849	-6.514567	1.490105	-2.176422	1.578761	-1.257788	1.585247	-0.711579	1.679564	0.036245	1.059497	-5.528418	174.3622	0.00059	0.007875
7560390	DPM2		10.68646	NM	00386	Homo sapiens	-1.011657	-6.681338	1.193358	-5.429299	1.578618	-0.234921	1.207268	-4.703683	1.59702	0.25014	1.322838	-2.646196	811.0124	0.001102	0.012623
6590703	GARS		9.015683	NM	00204	Homo sapiens	1.175983	-5.195427	1.338014	-3.127229	1.577927	0.063023	1.137784	-5.533181	1.341794	-2.808689	1.179305	-4.303416	13893.18	0.002211	0.021778
1940767	DNM1L		11.83196	NM	01206	Homo sapiens	1.688496	0.347073	1.717861	0.343639	1.577667	-1.115718	1.017392	-6.500241	-1.070248	-6.566305	-1.088862	-5.361604	1047.409	0.000713	0.009091
7610494	HSPC142		8.807732	NM	01417	Homo sapiens	1.285316	-3.58095	1.385698	-2.429945	1.576056	0.029803	1.078099	-6.164365	1.226202	-4.560622	1.137374	-4.79369	5596.26	0.002426	0.023345
270750	USP8		12.12007	NM	00515	Homo sapiens	1.654924	-0.9272	1.995734	1.432129	1.575218	-2.045837	1.205937	-5.325633	-1.0506	-6.693687	-1.266957	-4.064902	1348.379	0.000643	0.008416
6760333	EIF1AX		10.51469	NM	00141	Homo sapiens	1.357218	-3.510174	1.79221	0.663538	1.574767	-1.441834	1.320503	-3.798385	1.16029	-5.868097	-1.13808	-5.037918	7769.745	0.001179	0.013304
1050470	STAG2		10.56046	NM	00660	Homo sapiens	1.78882	-0.547977	1.973552	0.446169	1.573566	-2.685899	1.10327	-6.221754	-1.136794	-6.290253	-1.254191	-4.403715	3405.817	0.001158	0.013119
2320435	KIAA1409		12.19571	NM	02081	Homo sapiens	3.1844	1.525843	2.33062	-1.348409	1.569584	-4.923601	-1.366332	-5.37307	-2.028818	-2.336719	-1.484865	-4.118742	75.84423	0.000625	0.008243
4560128	OCIAD2		10.05366	NM	00101	Homo sapiens	-1.117838	-6.168295	1.236542	-5.347455	1.567746	-1.579679	1.382254	-3.111864	1.752486	0.463159	1.267847	-3.838973	7746.672	0.001421	0.015402
1230575	LOC90624		10.99297	NM	18170	Homo sapiens	1.623102	-1.201247	1.919117	0.918285	1.567588	-2.107395	1.182376	-5.541794	-1.035414	-6.739288	-1.224248	-4.44195	571.06	0.000978	0.011506
1300327	LOC643493		8.597254	XM	93157	PREDICTED:	1.327312	-5.026167	2.102624	0.042114	1.56197	-3.586936	1.584122	-2.864798	1.176792	-6.182753	-1.346136	-4.069068	155.202	0.002668	0.025113
2750014		0	10.73892	CD657255		AGENCOURT	1.138929	-6.3359	2.269056	0.443318	1.561534	-3.858647	1.992272	-0.567761	1.371055	-4.940851	-1.453094	-3.521101	17.42787	0.001079	0.01241
4290543	LRP11		11.87598	NM	03283	Homo sapiens	1.405147	-3.175469	1.952233	1.515978	1.55927	-1.913425	1.389344	-3.206185	1.109684	-6.355697	-1.252017	-4.091839	941.0479	0.000702	0.008985
6110048	LOC645317		11.1327	XM	93046	PREDICTED:	1.612001	0.365073	1.538031	-0.766653	1.55663	-0.601099	-1.048094	-6.388113	-1.035571	-6.71411	1.012093	-5.657503	2046.755	0.000927	0.011046
5570477	MELK		12.67305	NM	01479	Homo sapiens	1.64123	0.794067	1.619589	0.28884	1.556169	-0.491882	-1.013362	-6.503977	-1.05466	-6.61723	-1.040754	-5.580138	2613.548	0.000528	0.007236
4670626	SLC25A19		8.924018	NM	02173	Homo sapiens	-1.245994	-5.398228	1.240723	-5.819895	1.552485	-3.032899	1.545933	-2.549723	1.934387	0.190778	1.251275	-4.481628	686.1721	0.002303	0.022458
610026	PSMB10		14.34114	NM	00280	Homo sapiens	-1.194995	-5.185332	1.238201	-4.9943	1.552087	-0.980996	1.479644	-1.342259	1.854737	2.219517	1.253502	-3.674878	5365.275	0.000304	0.004801
5720768	NUP54		12.60939	NM	01742	Homo sapiens	1.671975	-0.413485	1.942222	1.498709	1.54977	-1.969977	1.161633	-5.654355	-1.078854	-6.550343	-1.253233	-4.06435	3895.851	0.00054	0.007367
2260168	C8orf30A		14.52215	NM	01645	Homo sapiens	-1.148685	-5.593632	1.201852	-5.245629	1.549729	-0.402461	1.380548	-2.125103	1.780149	2.281525	1.289451	-2.964714	1279.365	0.000287	0.004614
6040601	CTH		9.740343	NM	15374	Homo sapiens	1.311952	-4.083336	1.755142	0.285578	1.54965	-1.757113	1.33781	-3.623707	1.181179	-5.668875	-1.132605	-5.088689	1053.992	0.001618	0.017085
3940523	C6orf62		11.31967	NM	03093	Homo sapiens	1.554118	-0.222773	1.608566	0.107447	1.548338	-0.641347	1.035035	-6.445255	-1.003733	-6.78421	-1.038899	-5.58855	7075.176	0.000863	0.010475
6650520	NEFH		8.914243	NM	02107	Homo sapiens	-1.272228	-5.244193	1.248289	-5.819692	1.547907	-3.236982	1.588108	-2.359609	1.969292	0.182797	1.240023	-4.618769	168.0515	0.002313	0.022525
7210400	AASDHPPT		16.23289	NM	01542	Homo sapiens	2.137757	2.100005	2.103674	1.66963	1.547028	-2.638707	-1.016202	-6.505675	-1.381848	-3.951387	-1.359816	-3.389884	764.2616	0.000172	0.003077
610372	PRDX3		10.75066	NM	01409	Homo sapiens	1.684855	-0.391909	1.811301	0.392922	1.54538	-2.099569	1.075048	-6.307684	-1.090253	-6.489543	-1.172075	-4.826565	8638.166	0.001074	0.012367
4180280	PPP1R12A		12.48718	NM	00248	Homo sapiens	1.482716	-1.116255	1.730597	1.333831	1.54525	-0.715477	1.16718	-5.262246	1.042175	-6.685687	-1.119947	-5.033657	1449.09	0.000564	0.007606
6650754	SDHD		15.77131	NM	00300	Homo sapiens	1.472384	-1.810522	2.02706	2.975739	1.543503	-1.368572	1.37672	-2.83849	1.048302	-6.675022	-1.313285	-3.139042	3620.402	0.000196	0.003402
780278	LOC649555		9.755812	XM	94196	PREDICTED:	1.457997	-3.037608	1.928293	0.644549	1.542414	-2.607364	1.322564	-4.264613	1.057899	-6.674717	-1.250179	-4.30468	20.66585	0.001608	0.016986
5720576	ST6GALNAC4		8.596678	NM	17504	Homo sapiens	-1.200631	-5.633251	1.109844	-6.711509	1.540997	-2.676425	1.332514	-4.196763	1.85017	0.228657	1.388481	-3.131086	620.2378	0.002669	0.025115
3420471	ST6GAL1		10.56047	NM	17321	Homo sapiens	1.707288	-0.326076	1.811158	0.248063	1.53941	-2.274699	1.060839	-6.380439	-1.109054	-6.378579	-1.176527	-4.815756	6630.821	0.001158	0.013119
5490360	AIG1		11.88261	NM	01610	Homo sapiens	-1.188106	-5.533679	1.330318	-4.286136	1.537991	-1.979987	1.580558	-1.045193	1.827296	0.977152	1.156108	-4.917552	330.5034	0.0007	0.008966
4260187	PTPRK		10.18027	NM	00284	Homo sapiens	1.2654	-4.346197	1.678767	0.249813	1.537754	-1.309105	1.326669	-3.391345	1.215231	-5.081726	-1.091701	-5.322401	1847.77	0.001349	0.014757
4640128	WDR4		18.52629	NM	03366	Homo sapiens	-1.35957	-3.013351	1.131274	-6.309483	1.53591	-1.195468	1.538046	-0.693539	2.088176	3.866091	1.357681	-2.506788	256.1048	9.18E-05	0.00194
1450358	OPN3		13.9863	NM	00103	Homo sapiens	2.884391	2.043008	2.259578	-0.492761	1.534063	-4.545287	-1.276518	-5.56284	-1.880231	-2.124206	-1.472937	-3.753348	508.1918	0.00034	0.00523
60692	CDC42BPA		8.649081	NM	01482	Homo sapiens	-1.281718	-5.078154	1.027746	-7.055319	1.528421	-3.227972	1.317281	-4.59631	1.959006	0.376394	1.487159	-2.634841	398.8973	0.002606	0.024651
130750		0	11.17299	CD558852		AGENCOURT	-1.051522	-6.550486	-1.059923	-6.890618	1.52823	-1.117438	-1.007989	-6.510952	1.606967	0.061763	1.619806	0.096176	12.71552	0.000912	0.010907
5960113	UCHL5		12.55047	NM	01598	Homo sapiens	1.445252	-1.551537	1.743604	1.513947	1.525575	-0.905954	1.206436	-4.730005	1.055577	-6.613679	-1.142916	-4.797939	1551.542	0.000552	0.007466
2070152	CMTM6		11.3857	NM	01780	Homo sapiens	1.693115	-0.246477	1.8471	0.758332	1.520215	-2.299775	1.090948	-6.212121	-1.113734	-6.323581	-1.215026	-4.43225	3002.584	0.000842	0.010275
4050768	DSCR2		10.60041	NM	00372	Homo sapiens	1.442611	-1.461318	1.608742	0.274247	1.519813	-0.835313	1.11516	-5.829602	1.053516	-6.619948	-1.058513	-5.490255	4744.184	0.00114	0.012965
2340445	GNA13		13.23973	NM	00657	Homo sapiens	1.638347	0.516336	1.7384	1.245813	1.517224	-1.192487	1.06107	-6.320374	-1.079832	-6.459592	-1.145776	-4.80616	2815.584	0.000435	0.006246
6770647	C20orf6		10.22509	NM	01664	Homo sapiens	1.448081	-2.550702	1.824815	0.778548	1.514435	-2.210726	1.260161	-4.572347	1.045822	-6.699	-1.204947	-4.478234	2093.647	0.001325	0.014539
1230026	PRMT6		10.23467	NM	01813	Homo sapiens	1.399635	-2.484821	1.692223	0.542681	1.511008	-1.4835	1.209046	-4.852577	1.079573	-6.479102	-1.11993	-5.086578	3513.113	0.00132	0.014496
6980537		0	10.164	CR627122		Homo sapiens	2.070274	-0.820709	2.360787	0.220481	1.509974	-4.56514	1.140326	-6.204245	-1.371066	-5.161432	-1.563462	-3.131245	797.4253	0.001358	0.014845
2710204	RNF11		12.43326	NM	01437	Homo sapiens	1.672126	0.558676	1.721554	0.765795	1.508465	-1.564848	1.02956	-6.470249	-1.108495	-6.249013	-1.141262	-4.90308	362.0987	0.000575	0.007722
2710722	ARL6IP6		12.05071	NM	15252	Homo sapiens	1.876583	0.624019	1.90536	0.558487	1.506211	-2.908682	1.015335	-6.506255	-1.245896	-5.260164	-1.265003	-4.155565	3296.221	0.000659	0.008584
60382	CSPP1		10.95942	NM	02479	Homo sapiens	1.813297	-1.45076	2.313901	1.019251	1.504234	-4.089102	1.276074	-5.271343	-1.205462	-6.016999	-1.538258	-2.81553	138.5793	0.00099	0.011611
870221	PGM1		11.97676	NM	00263	Homo sapiens	1.721425	0.71051	1.711927	0.350106	1.503298	-1.89365	-1.005548	-6.512966	-1.145099	-5.944828	-1.138781	-4.975403	14275.37	0.000677	0.008763
1690273	ADD3		13.24923	NM	01682	Homo sapiens	1.992417	1.34967	1.919513	0.59956	1.501622	-2.995636	-1.03798	-6.46609	-1.326843	-4.459015	-1.278293	-4.057626	1736.769	0.000434	0.006231
5260673	WSB1		19.76833	NM	13426	Homo sapiens	4.019637	3.352756	2.902192	0.656946	1.500702	-5.240688	-1.385035	-5.248323	-2.678504	0.20228	-1.933889	-2.144681	534.8798	6.71E-05	0.001531
4070224	MAD2L1		10.50438	NM	00235	Homo sapiens	1.781231	0.054998	1.8064	-0.032449	1.499405	-2.868186	1.01413	-6.507233	-1.187958	-5.773303	-1.204744	-4.631953	8035.228	0.001184	0.013353
6020743		0	10.44523	BX110374		BX110374 So	1.671917	-1.864448	-1.162406	-6.474297	1.494143	-3.738105	-1.943446	0.133025	-1.118981	-6.443132	1.736801	-1.15795	17.19936	0.001212	0.013587
6180296	KIAA2026		12.37271	NM	00101	Homo sapiens	1.98443	1.517487	1.760587	-0.397998	1.491245	-2.949215	-1.127141	-6.009827	-1.330721	-4.319609	-1.180616	-4.826607	406.6998	0.000587	0.00785

7610292	LETM1		8.979734	NM	01231	Homo sapiens	-1.123316	-6.067031	1.16611	-6.019108	1.490035	-2.08079	1.30991	-3.745805	1.67378	0.166725	1.277782	-3.574663	577.1655	0.002246	0.022029
6770403	ANXA7		11.21536	NM	00403	Homo sapiens	1.282331	-3.520675	1.622168	0.806407	1.48502	-0.942267	1.265015	-3.63845	1.158062	-5.484872	-1.092355	-5.217505	3736.291	0.000898	0.010785
4250136	DUT		9.316011	NM	00102	Homo sapiens	-1.12543	-6.03327	1.204162	-5.55304	1.483309	-2.078352	1.3552	-3.118358	1.66936	0.221542	1.231819	-4.035533	3001.616	0.001939	0.019698
6900164	DDX3X		11.12241	NM	00135	Homo sapiens	1.544074	-0.232936	1.61896	0.339076	1.481953	-1.361045	1.048499	-6.38068	-1.041918	-6.683258	-1.09245	-5.256933	6828.57	0.00093	0.011068
6520008	HSP90AA1		11.64908	NM	00534	Homo sapiens	1.519266	-0.530554	1.669537	0.883388	1.479423	-1.394419	1.09891	-6.000312	-1.026931	-6.742211	-1.128505	-4.927562	23947.93	0.000763	0.00954
5890204	GPC4		9.614575	NM	00144	Homo sapiens	1.394123	-2.72896	1.704755	0.409331	1.479278	-2.07395	1.222816	-4.767145	1.061082	-6.610158	-1.152423	-4.831286	11590.44	0.001706	0.017771
70554	STK38L		9.367076	NM	01500	Homo sapiens	-1.154666	-5.752543	1.056813	-6.92906	1.477832	-2.189979	1.220267	-4.840915	1.706403	0.516775	1.398385	-2.27592	262.3635	0.001897	0.019358
3930189	SLC22A3		9.807489	NM	02197	Homo sapiens	1.312026	-5.592933	2.595302	0.551521	1.473639	-5.059798	1.978088	-1.629052	1.123178	-6.568801	-1.761152	-2.403203	20.69168	0.001573	0.016699
4060494	HIAT1		12.36035	NM	03305	Homo sapiens	1.959186	0.66426	1.998271	0.644952	1.472261	-3.595944	1.01995	-6.502243	-1.330733	-4.626033	-1.357281	-3.55545	621.8033	0.00059	0.007875
3440243	ATP6V1H		11.26365	NM	21362	Homo sapiens	1.990763	1.56172	1.562659	-2.172875	1.468303	-3.18447	-1.273958	-4.669342	-1.355826	-4.049237	-1.064262	-5.538117	22.83039	0.000882	0.010647
1070424	VBP1		12.07965	NM	00337	Homo sapiens	1.710527	0.258964	1.814397	0.881993	1.467674	-2.592671	1.060724	-6.361346	-1.165468	-5.809842	-1.23624	-4.123013	5418.517	0.000652	0.008511
840224	PPM1B		9.007371	NM	00103	Homo sapiens	1.552439	-3.096273	2.123161	0.400359	1.463607	-4.236648	1.367629	-4.451453	-1.060694	-6.697901	-1.450636	-3.249657	24.76716	0.002219	0.02184
1440487	PPAN		9.356751	NM	02023	Homo sapiens	-1.087999	-6.274597	1.163782	-5.814441	1.461808	-1.648078	1.266193	-3.853183	1.590446	0.211527	1.256084	-3.445138	1344.959	0.001905	0.019436
270446	SFRS3		10.2104	NM	00301	Homo sapiens	1.472534	-1.330494	1.650875	0.421094	1.46085	-1.856143	1.121112	-5.814288	-1.007998	-6.781399	-1.130078	-4.952823	5847.881	0.001333	0.01461
4590241	EPIM		9.712825	NM	19435	Homo sapiens	2.327049	-1.979539	3.194356	0.117676	1.458264	-5.857893	1.372707	-5.597702	-1.595767	-4.921504	-2.19052	-2.022696	39.88114	0.001637	0.017228
1030379	IARS2		9.324368	NM	01806	Homo sapiens	1.425579	-2.117355	1.648071	0.144673	1.457784	-2.097061	1.156072	-5.479853	1.022591	-6.758098	-1.130531	-4.985806	6039.314	0.001932	0.019636
5860392	NEDD4		16.27322	NM	00615	Homo sapiens	1.2904	-4.699857	2.292118	2.975897	1.455605	-3.3862	1.776285	0.025026	1.128027	-6.280688	-1.574684	-1.506836	16.51292	0.00017	0.003048
240600	LOC389599		10.01107	XM	37200	PREDICTED:	1.517609	-1.385272	1.728913	0.467992	1.454123	-2.506832	1.139235	-5.747249	-1.04366	-6.697013	-1.188974	-4.51045	1410.333	0.001446	0.015633
2450022	ATP11C		10.30453	NM	17369	Homo sapiens	1.243312	-4.729314	1.75231	0.699888	1.453934	-2.495181	1.409388	-2.501833	1.169404	-5.692693	-1.20522	-4.340759	1624.324	0.001283	0.014188
2690437	LOC643357		9.817207	XM	92668	PREDICTED:	1.75788	0.381844	1.628994	-1.074971	1.453487	-2.972141	-1.07912	-6.277787	-1.209423	-5.426114	-1.120749	-5.199605	16160.42	0.001567	0.016656
620246	NTHL1		9.194359	NM	00252	Homo sapiens	-1.10953	-6.094572	1.143031	-6.114059	1.452692	-1.921199	1.268227	-3.915207	1.611805	0.269976	1.270912	-3.340968	2493.076	0.002044	0.020532
7610164	PPFIBP1		10.54529	NM	00362	Homo sapiens	1.928282	0.197949	1.908176	-0.221113	1.451634	-3.956783	-1.010537	-6.511226	-1.328353	-4.762268	-1.314502	-3.999449	442.8444	0.001165	0.013186
4610382	C7orf28A		12.43444	NM	01562	Homo sapiens	1.384319	-2.316577	1.761348	1.759585	1.448385	-1.831305	1.272358	-3.767487	1.04628	-6.662664	-1.216077	-3.940139	2141.246	0.000575	0.007722
5090356	WDR46		8.583382	NM	00545	Homo sapiens	-1.132564	-5.933661	1.112569	-6.509205	1.447309	-2.362491	1.260056	-4.242301	1.63917	0.109138	1.300871	-3.195704	1775.177	0.002685	0.025248
2600184	GJA1		12.20097	NM	00016	Homo sapiens	1.538872	-0.295333	1.700855	1.205124	1.447228	-1.819402	1.105261	-5.938554	-1.063324	-6.559787	-1.17525	-4.417869	40222.61	0.000624	0.008234
6980402	AGL		9.412549	NM	00064	Homo sapiens	1.995169	0.349541	1.756092	-1.550298	1.444425	-4.178866	-1.136141	-6.081439	-1.381289	-4.404367	-1.215772	-4.796962	594.8368	0.00186	0.019053
1010341	YARS2		10.33288	NM	01593	Homo sapiens	1.429645	-1.830685	1.670366	0.685646	1.444387	-2.021228	1.168378	-5.258811	1.010312	-6.778923	-1.156453	-4.673744	4751.126	0.001268	0.014071
7330403	KIAA1033		10.55574	NM	01527	Homo sapiens	1.179586	-5.579241	1.788166	0.628861	1.44263	-2.906227	1.515927	-1.564847	1.222997	-5.195401	-1.239518	-4.099964	1300.86	0.00116	0.013138
4890220	FLJ90396		10.67403	NM	15335	Homo sapiens	1.652896	0.027027	1.691168	0.112158	1.437908	-2.704041	1.023154	-6.489308	-1.149515	-5.907525	-1.176131	-4.637353	1361.295	0.001107	0.01267
6380598	IMP4		8.817748	NM	03341	Homo sapiens	-1.203734	-5.298437	1.071859	-6.861381	1.437761	-2.909431	1.290232	-4.124611	1.730681	0.419303	1.341372	-3.025688	2405.968	0.002415	0.023267
1260538	HSD17B4		9.836979	NM	00041	Homo sapiens	1.388727	-2.098257	1.607346	0.38844	1.433727	-1.853947	1.157424	-5.300015	1.032404	-6.720857	-1.121096	-4.974361	8764.767	0.001554	0.016574
4570110	C11orf55		16.16482	NM	20742	Homo sapiens	-1.075622	-6.474374	-1.53951	-2.03889	1.431077	-3.275649	-1.431274	-2.727081	1.539298	-1.79096	2.203157	2.226871	18.68358	0.000175	0.003121
2850273	KIAA0776		12.60255	NM	01532	Homo sapiens	2.343804	0.681445	2.32638	0.332887	1.430692	-4.997358	-1.00749	-6.51336	-1.638231	-3.217035	-1.626052	-2.654169	251.407	0.000542	0.007379
4540731	CHRNB4		9.100811	NM	00075	Homo sapiens	2.392808	0.350013	1.867211	-2.452999	1.427137	-5.241357	-1.281488	-5.535159	-1.676649	-3.312001	-1.308362	-4.650875	40.97121	0.00213	0.021134
5890368	CLTC		8.725412	NM	00485	Homo sapiens	1.393645	-3.092038	1.739616	0.183732	1.421893	-3.165559	1.248249	-4.661118	1.020269	-6.767162	-1.223451	-4.266816	2737.668	0.002517	0.024056
2690333	ELOVL5		10.5385	NM	02181	Homo sapiens	1.647111	-1.091358	1.907377	0.702604	1.416721	-3.748385	1.158014	-5.775848	-1.162622	-6.000773	-1.346333	-3.382331	6911.615	0.001168	0.013213
5360689	HRC		12.51242	NM	00215	Homo sapiens	1.968491	-0.770651	2.453636	1.3076	1.414796	-4.93676	1.246455	-5.547322	-1.39136	-4.771928	-1.734268	-1.827806	142.257	0.000559	0.007548
5090019	ERBB3		12.49325	NM	00100	Homo sapiens	-1.664427	-1.987994	-1.115855	-6.756708	1.405681	-4.56606	1.491616	-3.251099	2.339652	2.029049	1.568536	-2.270989	168.9924	0.000563	0.007594
1190273	CDC14A		10.47194	NM	00367	Homo sapiens	2.03536	0.789028	1.7622	-1.327664	1.396665	-4.504415	-1.155011	-5.942147	-1.457301	-3.615713	-1.261721	-4.425478	271.22	0.001199	0.01348
3060438	CA2		8.811769	NM	00006	Homo sapiens	2.567256	0.062323	1.983909	-2.582725	1.390172	-5.781279	-1.294039	-5.661142	-1.846718	-2.989747	-1.427096	-4.299626	2934.947	0.002421	0.023312
6450523		0	14.89331	AK024852		Homo sapiens	-1.777071	-0.950204	-1.07672	-6.921478	1.383708	-4.648426	1.650448	-1.786589	2.458947	2.820559	1.489866	-2.700111	452.4139	0.000256	0.0042
4060725	ASCIZ		11.90239	NM	01525	Homo sapiens	1.662051	0.501855	1.695462	0.558428	1.382733	-3.117581	1.020102	-6.49357	-1.202004	-5.200653	-1.226167	-4.000299	881.6253	0.000695	0.008929
3840326	LOC643834		15.98444	XM	93219	PREDICTED:	2.508459	3.162796	1.497275	-3.498465	1.373787	-4.697187	-1.67535	-1.521469	-1.825945	-0.589805	-1.089889	-5.473932	27.86301	0.000184	0.003243
4010463	P4HA1		16.62133	NM	00101	Homo sapiens	3.067909	2.274853	2.509768	0.259552	1.36867	-5.697658	-1.222387	-5.892769	-2.241526	-0.588546	-1.833728	-2.061316	41.88143	0.000154	0.002834
4880717	ACSL1		11.05603	NM	00199	Homo sapiens	-1.38884	-3.58764	1.221023	-5.730953	1.367396	-4.237002	1.695806	-0.492886	1.899094	0.926645	1.119877	-5.25349	185.4466	0.000954	0.011296
6330333		0	10.47556	AI539442		te51e10.x1 Sc	2.153288	1.179072	1.285928	-5.539316	1.344808	-5.093798	-1.674501	-1.765035	-1.601186	-2.551813	1.045788	-5.616205	22.77546	0.001198	0.013464
670170	DNAJC10		8.864302	NM	01898	Homo sapiens	1.466659	-2.095892	1.709135	0.115266	1.340784	-4.020271	1.165326	-5.517697	-1.093881	-6.420143	-1.274728	-3.67251	948.6423	0.002365	0.022921
3850022	ANAPC10		9.071863	NM	01488	Homo sapiens	1.426845	-1.762341	1.605818	0.127374	1.328308	-3.537871	1.125433	-5.736714	-1.074182	-6.485426	-1.20892	-4.037269	1660.966	0.002157	0.021352
3140639	ATP8B2		17.92914	NM	00100	Homo sapiens	1.328481	-4.686454	2.589646	3.305258	1.324498	-5.154575	1.949329	0.391327	-1.003007	-6.784768	-1.955191	0.150962	22.83116	0.000107	0.002177
3360494	HPS4		10.60092	NM	15284	Homo sapiens	1.56121	-1.575893	1.866759	0.804618	1.320636	-4.595316	1.195713	-5.355521	-1.182165	-5.747554	-1.41353	-2.583179	21.96027	0.001139	0.012965
2070241	C18orf17		10.50295	NM	15321	Homo sapiens	-1.353669	-3.447497	-1.173994	-5.973928	1.309933	-4.409583	1.153046	-5.642747	1.773216	0.896323	1.537853	-1.105263	198.6785	0.001185	0.013358
7150537	BTBD3		10.28138	NM	01496	Homo sapiens	2.032036	0.627604	1.719898	-1.771739	1.299215	-5.447523	-1.181486	-5.782965	-1.564049	-2.773114	-1.323799	-3.998494	2367.434	0.001295	0.014298
2340093	AFF4		10.14559	NM	01442	Homo sapiens	1.785848	0.68372	1.569298	-1.608658	1.29445	-4.781259	-1.137991	-5.845215	-1.379619	-3.444809	-1.212328	-4.419995	1193.046	0.001368	0.014916
620474	MANEA		11.50416	NM	02464	Homo sapiens	3.289922	0.864336	2.256617	-2.354961	1.278896	-6.529694	-1.4579	-5.182535	-2.572469	-1.056688	-1.764503	-3.298431	90.43125	0.000805	0.009968
4290132	C14orf122		11.46807	NM	01604	Homo sapiens	-1.371153	-2.807645	1.107955	-6.527573	1.276066	-4.518078	1.519176	-0.839326	1.749681	1.368225	1.15173	-4.778485	678.8969	0.000816	0.010063
2940176		0	10.16513	BC030088		Homo sapiens	1.341763	-5.511009	2.691054	0.52897	1.276032	-6.300814	2.005611	-1.759876	-1.051512	-6.747162	-2.108924	-1.188325	17.13636	0.001357	0.014841
1300326	SFXN2		8.825337	NM	17885	Homo sapiens	-1.3874	-3.357087	1.074943	-6.86449	1.275446	-5.0639	1.491376	-2.078998	1.769554	0.350128	1.186524	-4.687482	246.259	0.002407	0.023221
4480204	SENP6		10.44321	NM	01557	Homo sapiens	1.433045	-1.758886	1.679094	0.811462	1.269211	-4.455358	1.171697	-5.207696	-1.129083	-5.976617	-1.322944	-2.696831	1580.178	0.001213	0.013589
830162	SCOTIN		8.623396	NM	01647	Homo sapiens	-1.479916	-2.951794	-1.151405	-6.440907	1.265016	-5.508383	1.285312	-4.714618	1.872117	0.264188	1.456546	-2.623271	154.1298	0.002637	0.02489
7550070	ATP5G1		8.135839	NM	00100	Homo sapiens	-1.250824	-4.190293	1.060153	-6.86978	1.260552	-4.470437	1.326065	-2.940831	1.576729	0.135766	1.189028	-4.239818	7041.224	0.003306	0.029671
4010110	MAN1A1		26.68588	NM	00590	Homo sapiens	4.594678	4.719235	2.591056	0.106011	1.258919	-6.400362	-1.773284	-2.977624	-3.6497	3.101887	-2.058158	-1.425735	74.60562	1.51E-05	0.0005
1170328	LAMB1		9.248854	NM	00229	Homo sapiens	1.457758	-2.1107	1.70191	0.164655	1.244638	-5.172256	1.167485	-5.46929	-1.17123	-5.688973	-1.367393	-2.644631	2854.898	0.001996	0.020146
2650661	LOC153561		10.79351	XM	93605	PREDICTED:	2.283622	0.198758	2.019263	-1.367619	1.238478	-6.333447	-1.130919	-6.239521	-1.843894	-2.091722	-1.630439	-2.747815	252.8549	0.001056	0.012202
1450315	LOC647970		11.66706	XM	94342	PREDICTED:	2.846039	1.341033	1.609056	-4.308298	1.238259	-6.47625	-1.768763	-2.906916	-2.29842	-0.618201	-1.299451	-4.806015	78.2406	0.000758	0.009492
5310240	HHEX		12.36405	NM	00272	Homo sapiens	2.469668	1.205014	1.873893	-1.97627	1.22404	-6.375202	-1.317935	-5.174618	-2.017636	-0.924361	-1.530907	-3.1959	299.0178	0.000589	0.00787
5220762	FLJ20010		8.773707	NM	01902	Homo sapiens	-1.318181	-3.236322	1.026957	-7.034725	1.220025	-5.09376	1.353716	-2.585969	1.608214	0.432531	1.187999	-4.26906	525.3442	0.002463	0.02363
5690736		0	15.42921	BI046034		MR3-FN0206-	1.705179	-3.008947	2.998052	2.229769	1.203393	-6.558974	1.758204	-2.551711	-1.416976	-4.936145	-2.491332	0.493915	32.80394	0.000217	0.003687
60364	MTG1		9.463484	NM	13838	Homo sapiens	-1.425866	-2.252163	-1.073312	-6.822419	1.193749	-5.688745	1.328473	-3.356814	1.702125	0.709895	1.281265	-3.423806	675.6099	0.00182	0.018726
6040553	PCSK1		11.02599	NM	00043	Homo sapiens	1.966462	0.975602	1.142307	-6.505901	1.191478	-6.180753	-1.721483	-0.684635	-1.65044	-1.460462	1.043045	-5.611945	24.94507	0.000965	0.01139
7160201		0	14.48387	AK124143		Homo sapiens	3.268336	2.080006	1.643967	-4.297779	1.173734	-6.789459	-1.988078	-2.060447	-2.784563	0.738567	-1.400631	-4.411092	29.93951	0.00029	0.004647
5130066	ATP5D		9.440098	NM	00168	Homo sapiens	-1.379306	-2.259927	-1.086784	-6.654456	1.172606	-5.712843	1.269163	-3.72412	1.617383	0.676216	1.27437	-3.143656	1086.237	0.001838	0.01889
4210273	CADPS2		9.603722	NM	01795	Homo sapiens	-1.505212	-1.841845	-1.174798	-6.017827	1.168326	-6.147774	1.281251	-4.284694	1.758578	0.544839	1.372548	-2.769661	434.1663	0.001714	0.017828
110091	THAP2		12.45093	NM	03143	Homo sapiens	2.257324	1.598577	1.363624	-4.910488	1.167214	-6.529852	-1.655386	-1.988467	-1.933942	-0.247508	-1.168273	-5.118036	27.19215	0.000571	0.007685
6420280	MANEA		23.43243	XM	93898	PREDICTED:	3.515323	4.1768	1.805243	-2.458336	1.138658	-6.821014	-1.947285	-1.186679	-3.08725	3.095026	-1.585412	-2.921018	25.78372	2.90E-05	0.000814
5220170	OPN3		37.28419	NM	01432	Homo sapiens	3.025531	3.898468	3.500924	5.01316	1.135411	-6.769605	1.157127	-6.034247	-2.664702	2.699569	-3.083399	2.69113	39.86708	2.65E-06	0.000146
6980129	PGK1		9.196977	NM	00029	Homo sapiens	1.757889	0.133018	1.424565	-3.410031	1.134082	-6.542131	-1.233983	-4.989938	-1.550055	-1.828826	-1.25614	-4.102866	14530.42	0.002042	0.02052
6420520	RFXAP		15.50595	NM	00053	Homo sapiens	1.897956	2.058333	1.558515	-1.305561	1.123226	-6.524131	-1.217797	-4.906666	-1.689737	0.254863	-1.387535	-2.445669	405.9405	0.000212	0.003617

6860494	F2R		11.48902	NM 00199	Homo sapiens	1.797681	0.703454	1.517026	-2.220584	1.116002	-6.653419	-1.185004	-5.420624	-1.610822	-1.017054	-1.359339	-3.009623	1142.9	0.00081	0.010002
2100445	MMAA		18.18743	NM 17225	Homo sapiens	-2.113322	1.091035	-1.753084	-1.518235	1.103382	-6.873593	1.205488	-5.608651	2.331801	2.150275	1.934321	-0.072852	98.56752	0.0001	0.002068
2480471	OLFML2A		13.313	NM 18248	Homo sapiens	1.953274	0.932031	1.651203	-1.664068	1.08327	-6.927598	-1.18294	-5.631451	-1.803127	-0.188894	-1.524276	-2.055781	1281.309	0.000425	0.006156
5910736		0	20.00273	AI354654	qv15e02.x1 N	-1.24155	-5.33455	-2.284612	2.40169	1.068329	-7.001555	-1.84013	0.06303	1.326383	-4.621035	2.440717	2.23548	51.15281	6.34E-05	0.001462
430376	CLDN18		26.02376	NM 01636	Homo sapiens	5.629202	4.315725	1.424589	-5.86837	1.054075	-7.113104	-3.951457	2.194456	-5.340418	3.993202	-1.351506	-4.872952	26.74831	1.71E-05	0.000553
5290646		0	9.657304	DB304419	DB304419 BR	-1.666297	-1.96854	1.218981	-6.08382	1.046441	-7.086209	2.031183	0.578237	1.743681	-1.461224	-1.164883	-5.126699	75.97871	0.001676	0.017522
1780671	OR7E156P		21.60021	NR 00217	Homo sapiens	3.376911	3.339528	1.837521	-2.618137	1.026196	-7.13086	-1.837754	-2.147194	-3.290709	3.102323	-1.790615	-2.103898	92.75709	4.35E-05	0.001105
2360452	LOC440925		14.90542	NM 00101	Homo sapiens	2.460775	1.586512	1.626597	-3.293578	1.026098	-7.128038	-1.512837	-3.622222	-2.398189	1.276874	-1.585227	-2.677219	191.4709	0.000255	0.00419
4810543	LOC648998		19.2682	XM 93807	PREDICTED:	-1.059447	-6.499558	1.783691	1.773985	1.013144	-7.132646	1.889726	2.920517	1.073373	-6.501052	-1.76055	1.204981	12.73007	7.60E-05	0.001685
6960382		0	8.769816	CR748468	CR748468 So	1.52199	-2.084377	-1.166225	-6.213816	1.011492	-7.13747	-1.774983	0.305935	-1.504698	-2.338841	1.179627	-4.795668	14.63512	0.002467	0.023655
830139	PRSS3		17.53952	NM 00277	Homo sapiens	5.044512	2.262038	2.258655	-3.27032	1.003876	-7.142467	-2.233414	-2.859306	-5.025033	2.196679	-2.249933	-2.43467	41.45297	0.000119	0.002356
