# Supplementary material for: A Continuum of Cell States Spans Pluripotency and Lineage Commitment in Human Embryonic Stem Cells
Source: PLoS One. 2009 Nov 5;4(11):e7708. doi: 10.1371/journal.pone.0007708 (PMC2768791; doi:10.1371/journal.pone.0007708)
Supplement: Table S3 — (0.05 MB DOC) [file pone.0007708.s003.doc]

Table S3. Primer / probe sets used for analysis of gene expression by qPCR

| **Gene Symbol** | **Accession** | **ABI assay ID** |
| --- | --- | --- |
| CRIPTO1 | NM_003212 | Hs02339499_g1 |
| CYCLOPHILIN A | NM_021130 | Hs99999904_m1 |
| DNMT3b | NM_006892 var 1 | Hs00171876_m1 |
|  | NM_175848 var 2 |  |
|  | NM_175849 var 3 |  |
|  | NM_175850 var 6 |  |
| GATA4 | NM_002052 | Hs00171403_m1 |
| GATA6 | NM_005257 | Hs00232018_m1 |
| GDF3 | NM_020634 | Hs00220998_m1 |
| GSC | NM_173849 | Hs00418279_m1 |
| LHX2 | NM_004789 | Hs00180351_m1 |
| MIXL1 | NM_031944 | Hs00430824_g1 |
| NANOG | NM_024865 | Hs02387400_g1 |
| OCT4 | NM_002701 | Hs01895061_u1 |
| PAX6 | NM_000280 var 1 | Hs00240871_m1 |
|  | NM_001604 var 2 |  |
| SOX17 | NM_022454 | Hs00751752_s1 |
| T | NM_003181 | Hs00610080_m1 |
