# Supplementary figures and images for: A Continuum of Cell States Spans Pluripotency and Lineage Commitment in Human Embryonic Stem Cells
Source: PLoS One. 2009 Nov 5;4(11):e7708. doi: 10.1371/journal.pone.0007708 (PMC2768791; doi:10.1371/journal.pone.0007708)

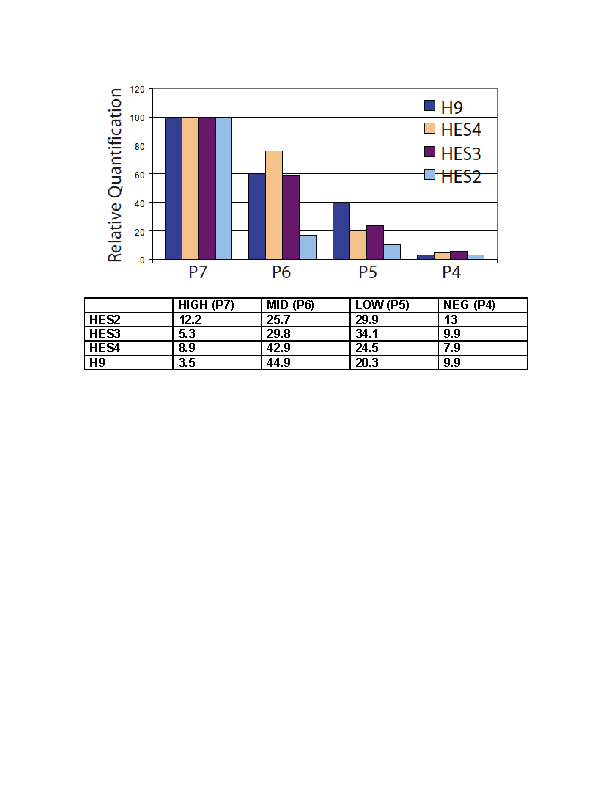

Supplement: Figure S1 — QRT-PCR analysis of GDF-3 expression in immunologically defined subpopulations of four different human ES cell lines. Table below the Figure indicates the proportion of cells in each subpopulation for the different cell lines. (0.05 MB TIF) [file pone.0007708.s004.tif]

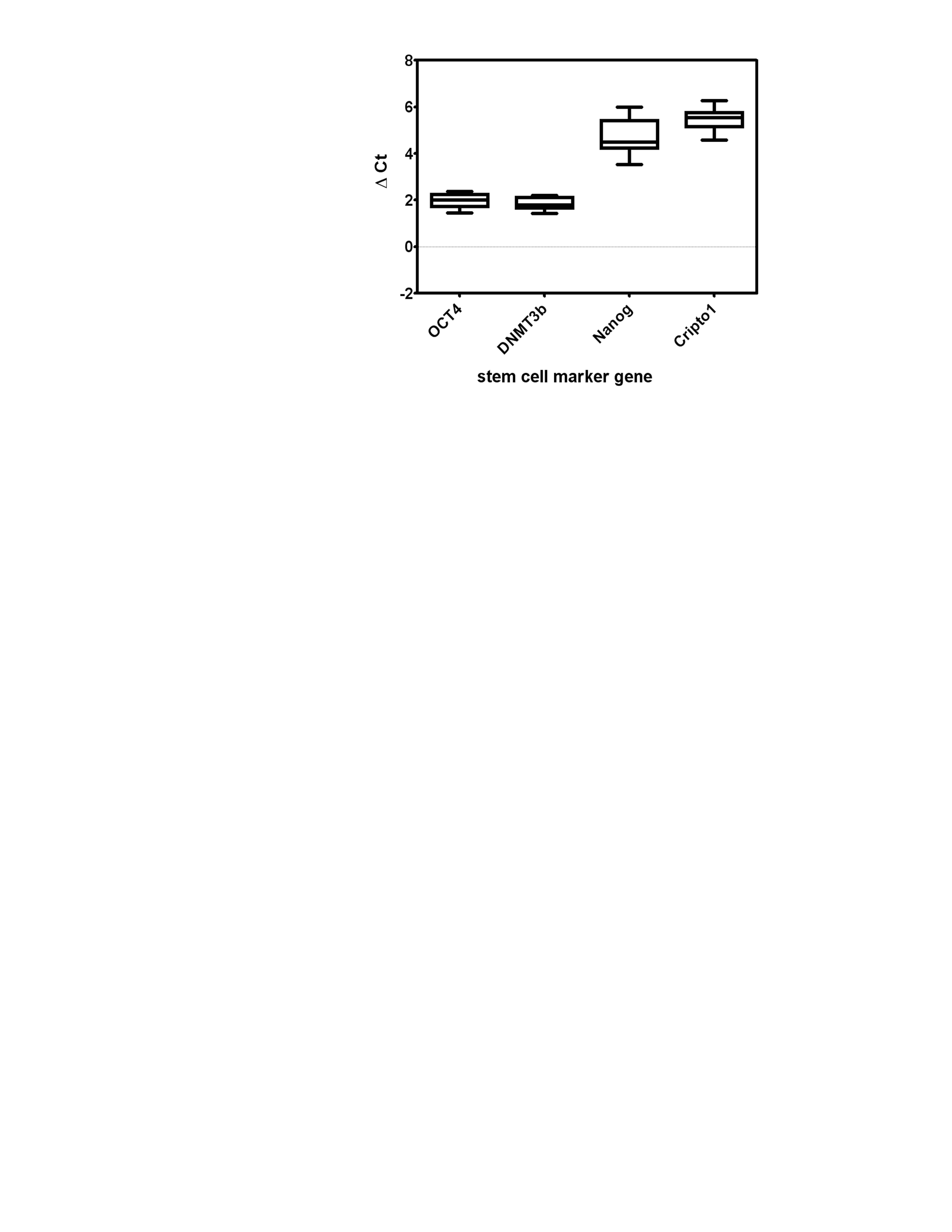

Supplement: Figure S2 — Reproducibility of unbiased global amplification of human embryonic stem cells. Expression of four stem cell marker genes normalized to cyclophilin in nine single cell equivalents prepared from a pool of nine lysed HES3 cells. Each single cell equivalent was separately subjected to mRNA isolation, reverse transcription and global cDNA amplification. Boxes in the box plots indicate the interquartile range (IQR) with the median; the whiskers indicate highest and lowest points. (1.59 MB TIF) [file pone.0007708.s005.tif]

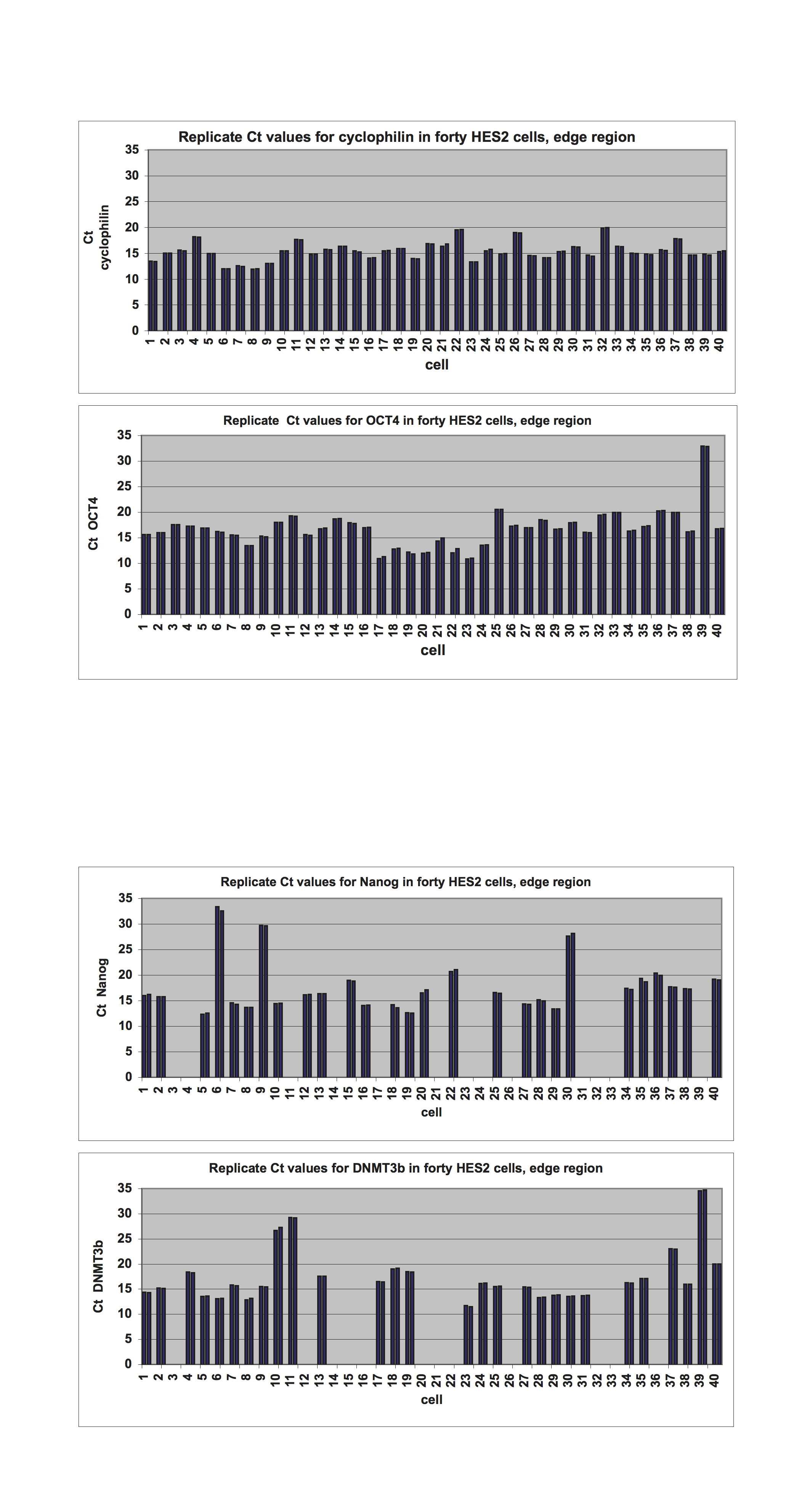

Supplement: Figure S3 — Reproducibility of single cell Ct measurements. Duplicate values for Ct measurements for four genes on forty single cells isolated by manual dissection are shown. (0.60 MB TIF) [file pone.0007708.s006.tif]

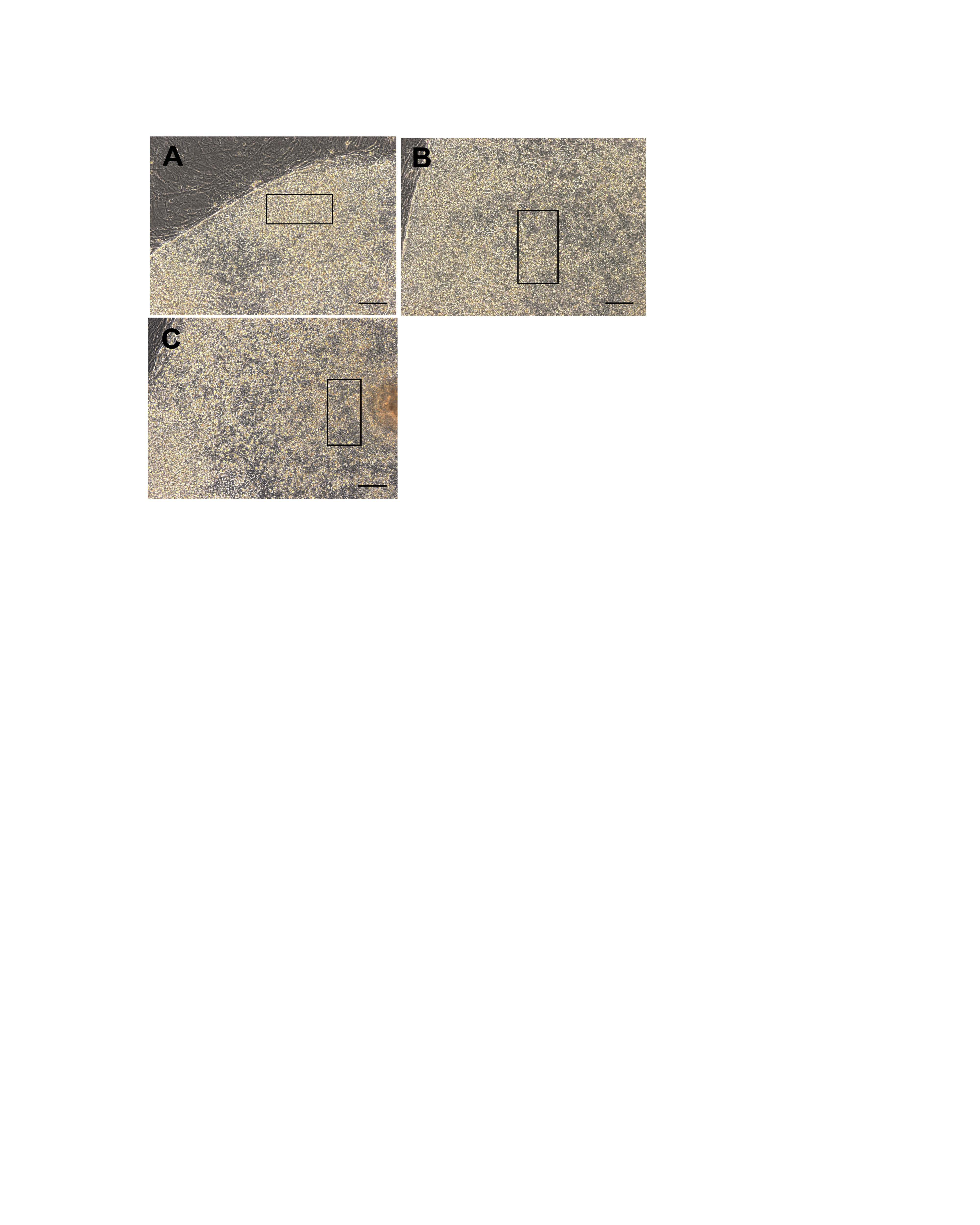

Supplement: Figure S4 — Isolation of single ES cells from three colony regions Small sections were excised from the edge (A), mid (B), and adjacent center (C) regions of HES2 colonies. Single cells were isolated for global RT-PCR analysis from each section as described in the materials and methods. Scale bars equal 100 µM. (8.71 MB TIF) [file pone.0007708.s007.tif]
